# Quantitative Phosphoproteomic Profiling of Mouse Sperm Maturation in Epididymis Revealed Kinases Important for Sperm Motility

**DOI:** 10.1016/j.mcpro.2024.100810

**Published:** 2024-07-06

**Authors:** Xiangzheng Zhang, Haixia Tu, Xin Zhou, Bing Wang, Yueshuai Guo, Chenghao Situ, Yaling Qi, Yan Li, Xuejiang Guo

**Affiliations:** 1 State Key Laboratory of Reproductive Medicine and Offspring Health, Department of Histology and Embryology, Nanjing Medical University, Nanjing, China; 2 Department of Clinical Laboratory, Sir Run Run Hospital, Nanjing Medical University, Nanjing, China; 3 School of Medicine, Southeast University, Nanjing, China

**Keywords:** phosphoproteomics, epididymis, sperm maturation, sperm, phosphorylation, motility

## Abstract

Transcriptionally and translationally silent sperm undergo functional maturation during epididymis traverse, which provides sperm ability to move and is crucial for successful fertilization. However, the molecular mechanisms governing sperm maturation remain poorly understood, especially at the protein post-translational modification level. In this study, we conducted a comprehensive quantitative phosphoproteomic analysis of mouse epididymal sperm from different regions (caput, corpus, and cauda) to unveil the dynamics of protein phosphorylation during sperm maturation. We identified 6447 phosphorylation sites in 1407 phosphoproteins, and 345 phosphoproteins were differentially phosphorylated between caput and cauda sperm. Gene ontology and KEGG pathway analyses showed enrichment of differentially phosphorylated proteins in energy metabolism, sperm motility, and fertilization. Kinase substrate network analysis followed by inhibition assay and quantitative phosphoproteomics analysis showed that TSSK2 kinase is important for sperm motility and progressive motility. This study systemically characterized the intricate phosphorylation regulation during sperm maturation in the mouse epididymis, which can be a basis to elucidate sperm motility acquisition, and to offer potential targets for male contraception and the treatment of male infertility.

## Highlights

• Characterization of the phosphoproteome of mouse sperm maturation in epididymis.
• Differentially phosphorylated proteins are enriched in sperm motility.
• TSSK2 is important for sperm motility and progressive motility.

## Graphical Abstract

**Figure:**
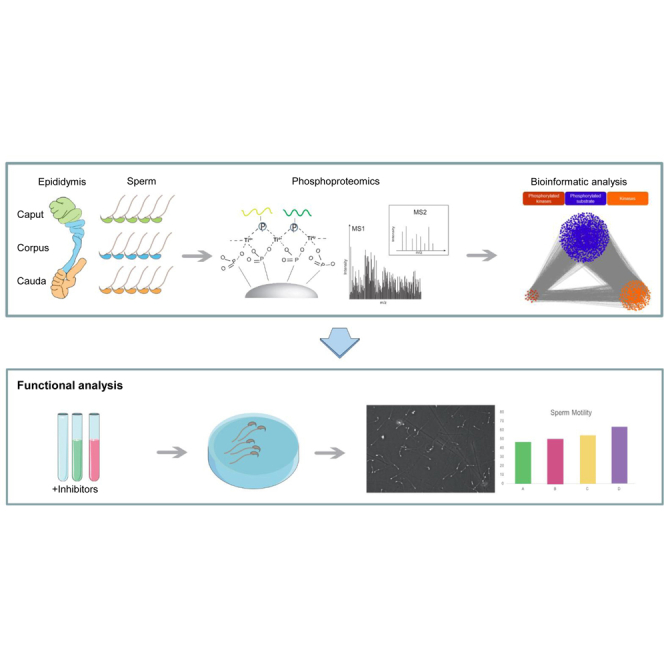

## In Brief

In this study, we conducted a comprehensive quantitative phosphoproteomic analysis of mouse epididymal sperm from different regions (caput, corpus, and cauda). Phosphoproteomics analyses and inhibition assay showed that TSSK2 kinase is important for sperm motility and progressive motility. Our systemic characterization of the dynamic phosphoproteome of sperm maturation in epididymis can be a basis to elucidate sperm motility acquisition and to offer potential targets for male contraception and the treatment of male infertility.

Infertility affects approximately 15% of men and usually manifests as impaired spermatogenesis, presenting as the absence of sperm (azoospermia) or various degrees of sperm motility decrease (asthenozoospermia) and number decrease (oligozoospermia) (1). As the only cell to perform functions outside the male body in mammals, functional motile sperm fertilize the egg in the female fallopian tube to form the embryo. The sperm is formed in the testis by spermatogenesis. The tadpole-like sperm released from the testis is mature in structure but not functionally mature because it lacks motility and the ability to recognize and fertilize the egg (2, 3). After release from the testis, sperm gradually acquire progressive motility and fertilization potential during the transportation from the proximal to the distal region of the epididymis (4). Only mature sperm from cauda epididymis can undergo capacitation and acrosome reaction, events required before successful fertilization (5). The capacity of sperm to bind to zona-free oocytes and the early embryo development ability of sperm from higher segments of the epididymis are reduced (6). The sperm from different parts of the epididymis have different maturation states.

Epididymis is a crescent-shaped tube-like organ that connects the testis to the vas deferens and is mainly divided into three anatomical regions: caput (head), corpus (body), and cauda (tail) (7). The constantly changing luminal environment created by the epididymal epithelium can regulate sperm maturation (8). For example, serine (or cysteine) peptidase inhibitor (HongrES1) involved in the regulation of sperm capacitation and male fertility is specifically expressed in the caudal segment of the rat epididymis and binds to the head of sperm as it passes through the caudal segment of the epididymis (9, 10). Skerget *et al.* performed the first quantitative proteomics analysis of sperm protein expression changes during epididymal maturation (11). David *et al.* performed quantitative proteomic profiling of sperm maturation and identified RHOA as an important protein for sperm maturation (12).

Post-translational modifications, including phosphorylation, glycosylation, ubiquitination, and acetylation, are also important for protein regulation, especially in transcriptionally and translationally silent sperm (13, 14). Tourzani *et al.* found that caput sperm have higher levels of O-GlcNAcylation in comparison to mature cauda sperm (15). In recent years, with the development of mass spectrometry technology, it has become possible to systemically analyze protein phosphorylation in tissues or cells (16, 17), which can help us to gain a deeper understanding of the molecular mechanism of the maturation of sperm. Baker *et al.* have performed an MS-based quantification of sperm phosphorylation from different regions of rat epididymis and identified 22 regulated phosphoproteins in rats (18). However, the molecular mechanisms underlying sperm maturation are still largely unknown. Due to the significantly lower number of sperm in mouse epididymis than in rats (19, 20), sperm phosphoproteome from different regions of epididymis has not been explored in mice.

In this study, using the sensitive phosphoproteomics method, we identified 6447 phosphorylation sites of sperm proteins from mouse caput, corpus and cauda epididymis, and found 345 proteins showing differentially regulated phosphorylation levels, which are enriched in the processes of sperm motility and energy metabolism. Kinase-substrate relationship analysis followed by inhibition assay revealed important roles of TSSK2 kinase-mediated pathway in the regulation of motility acquisition in sperm maturation.

## Experimental Procedures

### Experimental Design and Statistical Rationale

All animal experiments were approved by the Animal Care and Use Committee of Nanjing Medical University (approval number: IACUC-1707017). Adult male ICR mice, aged 8 to 10 weeks, were procured from the experimental animal center of Nanjing Medical University. For the proteomic and phosphoproteomic analysis of mouse epididymal sperm from different regions, proteins were extracted from sperm collected from the caput, corpus, and cauda regions of the epididymis of 12 ICR mice in each of the five biological replicates. For the proteomic and phosphoproteomic analysis of kinase inhibitor-treated and control sperm, proteins were extracted from sperm collected from cauda epididymis of one ICR mouse in each of the five biological replicates. The protein from each sample was digested, and labeled by TMTpro (sample information corresponding to the channels of the TMTpro reagents is provided in the Supplemental Table S1) and mixed. 40 μg of TMTpro-labeled mixed peptides were subjected to quantitative proteomics analysis, and the rest were subjected to phosphorylation enrichment for quantitative phosphoproteomics assay. The false discovery rate (FDR) cut-off value for proteins, peptides, and phosphorylation sites was 0.01. After normalization against the protein expression levels, differences in phosphorylation sites between sperm from caput and cauda epididymis were analyzed using an independent Student's *t* test. *p* value <0.05 and fold change >1.5 were considered significant. Fisher's precise test is used for kinase enrichment analysis. And an independent Student's *t* test was used in comparison between the two groups. *p* value <0.05 was used as the cut-off value for both statistical analyses.

### Isolation of Epididymal Sperm

The epididymis was swiftly removed from adult mice and divided into caput, corpus, and cauda sections. The divided tissue was incised and placed in PBS, allowing the sperm to be released into the PBS through gentle blowing using a dropper. The resulting sperm suspensions were filtered through a 40 μm filter and subsequently subjected to two rounds of centrifugation at 400*g* for 15 min on a 28% Percoll/PBS gradient. The sperm located at the bottom of the centrifuge tubes were resuspended in red blood cell lysis buffer (Invitrogen), incubated at room temperature for 5 min, and then centrifuged at 1000*g* for 10 min to collect the sperm. Following three washes with PBS, the purity of the sperm was assessed using a smear microscope.

### Protein Sample Preparation and TMT Labeling

Protein extraction and digestion were performed according to a previously established protocol as described (21). Sperm samples were lysed using sonication in a lysis buffer composed of 8 M Urea, 1 mM DTT, 75 mM NaCl, and 50 mM Tris (pH 8.2), supplemented with a protease and phosphatase cocktail. After incubation on ice for 1 h, the lysates were centrifuged at 40,000*g* for 60 min at 4 °C. The resulting supernatant was quantified using the Bradford assay, and then subjected to reduction, alkylation, and trypsin digestion. The digested peptides were subsequently desalted using an OASIS HLB Vac cartridge (Waters). For TMT labeling, the purified peptides were reconstituted in 200 mM HEPES (pH 8.5), and labeled using the first 15 channels of the TMT18-plex according to the manufacturer’s instructions. For the phosphoproteomic analysis of sperm from caput, corpus and cauda regions of epididymis, peptide digest from 50 μg protein in each replicate was labeled with 200 μg of TMTpro reagent. And for the phosphoproteomic analysis of sperm incubated with DMSO or ALK inhibitor2 compound 18, peptide digest from 90 μg protein in each replicate was labeled with 400 μg of TMTpro reagent. All labeled peptide samples in each experiment were combined, purified, and lyophilized.

### High-pH Reverse Phase Fractionation

For proteomics quantification, the TMT-labeled peptide mixture was fractionated on a nanoEase M/Z Peptide BEH C18 column (300 μm × 150 mm, 1.7 μm; 130 Å, Waters) as previously described (22) with a 128-min gradient (3% buffer B for 14 min, 3% to 8% B for 1 min, 8% to 29% B for 71 min, 29% to 41% B for 12 min, 41% to 100% B for 1 min, 100% B for 8 min, and 100% to 3% B for 1 min followed by 20 min at 3% B). Buffer A (20 mM ammonium formate, pH 10) and buffer B (100% acetonitrile) were used as the mobile phase. A total of thirty fractions were collected using a non-adjacent pooling scheme and subsequently lyophilized.

For phosphoproteomic quantification, the TMT-labeled peptide mixture was fractionated on an XBridge Peptide BEH C18 column (2.1 mm × 150 mm, 3.5 μm; 130 Å, Waters) using a 34-min gradient (0%–10% buffer B for 3 min, 10%–27% B for 16 min, 27%–31% B for 1 min, 31%–39% B for 4 min, and 39%–60% B for 4 min, followed by 6 min at 60% B). Buffer A (5 mM ammonium formate, pH 10) and buffer B (100% acetonitrile) were used as mobile phase. A total of 10 fractions were collected using a non-adjacent pooling scheme and then lyophilized for subsequent phosphopeptide enrichment.

### Phosphopeptide Enrichment

Phosphopeptides from each fraction were enriched using immobilized metal affinity chromatography (Ti4+-IMAC, JK Chemical) following a previously described method with adaptations (23). In brief, the peptides were resuspended in loading buffer (80% acetonitrile (ACN), 3% trifluoroacetic acid (TFA)) and then incubated with Ti4+-IMAC beads. After a 20-min incubation at room temperature, the beads were washed sequentially with Wash Buffer1 (50% ACN, 3% TFA, 200 mM NaCl) and Wash Buffer2 (30% ACN, 0.1% TFA). The phosphopeptides were subsequently eluted twice with Elution Buffer1 (10% NH4OH) and once with Elution Buffer2 (80% ACN). The eluates were pooled, dried and subjected to MS analysis.

### LC-MS/MS Analysis and Data Processing

For LC-MS/MS analysis, peptides or phosphopeptides were dissolved in a solution of 0.1% formic acid (FA) and subjected to analysis using an Orbitrap Fusion Lumos mass spectrometry system (ThermoFisher) coupled with the Easy-nLC 1200 (ThermoFisher). Solvent A consisted of water with 0.1% FA, while solvent B comprised 80% ACN and 0.1% FA. The peptides were separated using an analytical column (75 μm × 25 cm, Acclaim PepMap RSLC C18 column, 2 μm, 100 Å; DIONEX) with a linear 95 min gradient (3%–5% B for 5 s, 5%–15% B for 40 min, 15%–28% B for 34 min and 50 s, 28%–38% B for 12 min, 30%–100% B for 5 s, and 100% B for 8 min) in data-dependent acquisition mode. The Orbitrap Fusion Lumos was configured with a resolution of 60K for MS1 and 50K for MS2. The quadrupole isolation window was set at 0.8Th and 1.6Th for proteomic and phosphoproteomic quantification, respectively.

The raw files were analyzed using MaxQuant software (version 2.2.0.0, https://www.maxquant.org) and searched against the mouse protein sequences obtained from the Universal Protein Resource (UniProt, 2022.09; 55,315 entries) database. Trypsin was selected as the enzyme of choice, and a maximum of two missed cleavages was permitted. The mass tolerance for both precursor and fragment ions was set to 20 ppm. Carbamidomethylation (Cys) was set as a fixed modification. Variable modifications considered in the search included oxidation (M) and acetylation (protein N-terminus) with phosphorylation (STY) for phosphopeptides. False discovery rate (FDR) cut-offs were set to 0.01 for proteins, peptides, and phosphorylation sites to control the rate of false identification. Only class I phosphorylation sites were further analyzed in downstream analysis. For each protein, its expression levels in each sample were normalized by dividing the average expression level of the protein across samples. For phosphorylation site quantification, the levels of each phosphorylation site were normalized by dividing its average level across samples. To avoid the effect of protein expression changes, the level of each phosphorylation site was further calibrated by dividing the normalized expression level of the corresponding protein. If the corresponding proteins could not be quantified by proteomic analysis, the normalized level of the phosphorylation site was used directly for statistical analysis (24). Statistical analysis of differences in phosphorylation sites was conducted using Student’s *t* test.

### Bioinformatics Analysis

The regulatory relationships between kinases and phosphorylation sites were annotated using iGPS 1.0 (25) and visualized through Cytoscape 3.10.0 (26). Kinase enrichment analysis was executed using Fisher’s exact test, contrasting significantly upregulated or downregulated phosphorylation sites against the remainder of phosphorylation sites (23). Gene ontology (GO) and Kyoto Encyclopedia of Genes and Genomes (KEGG) pathway enrichment analyses were performed utilizing the clusterProfiler 4.6.2 package (27) with an adjusted *p* value less than 0.05 as a cut-off.

### Immunoblotting

Protein lysates were boiled at 100 °C for 10 min in SDS-PAGE loading buffer (FDBIO), separated by 10% SDS-PAGE, and transferred onto polyvinylidene difluoride (PVDF) membranes. The membranes were blocked with 5% BSA (bull serum albumin) in TBST (Tris-buffered saline with Tween 20) and incubated overnight at 4 °C with primary antibodies, including Anti-Phospho-Tyrosine (PTM BIO) and Anti-Alpha-Tubulin (Proteintech). Subsequently, the membranes were washed three times with TBST, and incubated with secondary antibodies (Goat Anti-Rabbit IgG H&L (HRP); Abcam) for 2 h at room temperature. Protein bands were visualized using the High-sig ECL Western Blotting Substrate (Tanon).

### Kinase Inhibition Assays

The incision made in the epididymal caudal region was incubated in serum-free modified HTF medium (Irvine Scientific) at 37 °C for 10 min. Subsequently, the resulting sperm suspension was divided into four equal portions in replicate. Each portion was supplemented with a final concentration of 10 μM ML281, 1 μM ALK inhibitor2 compound 18, or 0.02% dimethyl sulfoxide (DMSO) as a control, and incubated at 37 °C for 1 h. Mouse sperm motility was then assessed using a computer-assisted semen analysis system (Hamilton Thorne Research).

### ATP Measurements

After the incubation with the kinase inhibitors or DMSO, the sperm were collected and washed twice with PBS (phosphate-buffered saline). The ATP content of the sperm was measured according to the instructions provided by the manufacturer of the ATP assay kit (Beyotime). The ATP measurements were corrected for the protein concentration determined by the BCA (bicinchoninic acid) method.

## Results

### Quantitative Phosphoproteomic Profiling of Maturing Sperm From Mouse Caput, Corpus and Cauda Epididymis

**Fig. 1:**
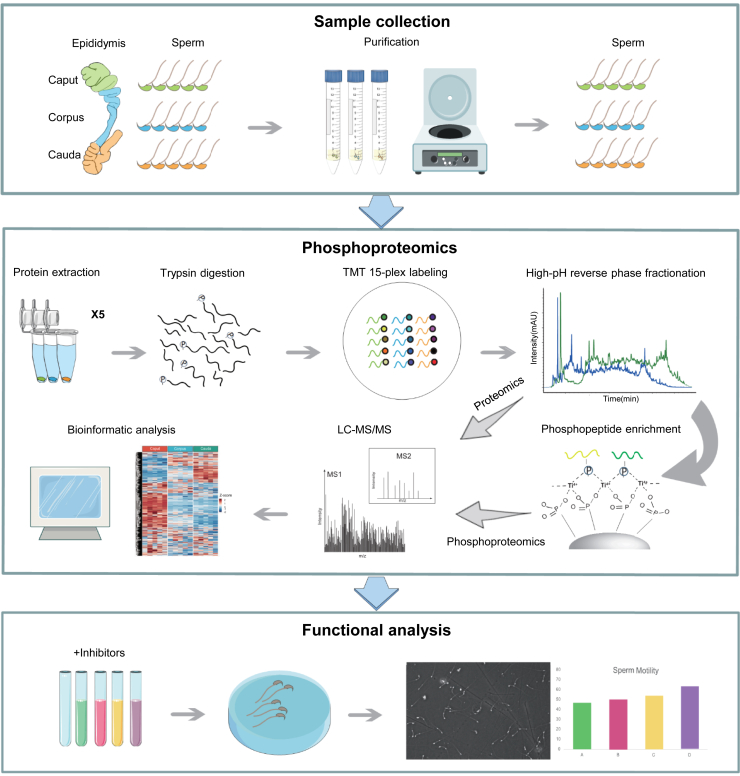
**Workflow of phosphoproteomic analysis of maturing mouse sperm from different epididymal regions.** Workflow depicting the experimental setup for quantitative phosphoproteomic analysis of mouse sperm during epididymal maturation. Sperm from the caput, corpus, and cauda regions of the epididymis were isolated and subjected to trypsin digestion, TMT labeling, high-pH reverse phase fractionation, Ti4+-IMAC enrichment, LC-MS/MS analysis and bioinformatic analysis.

**Fig. 2:**
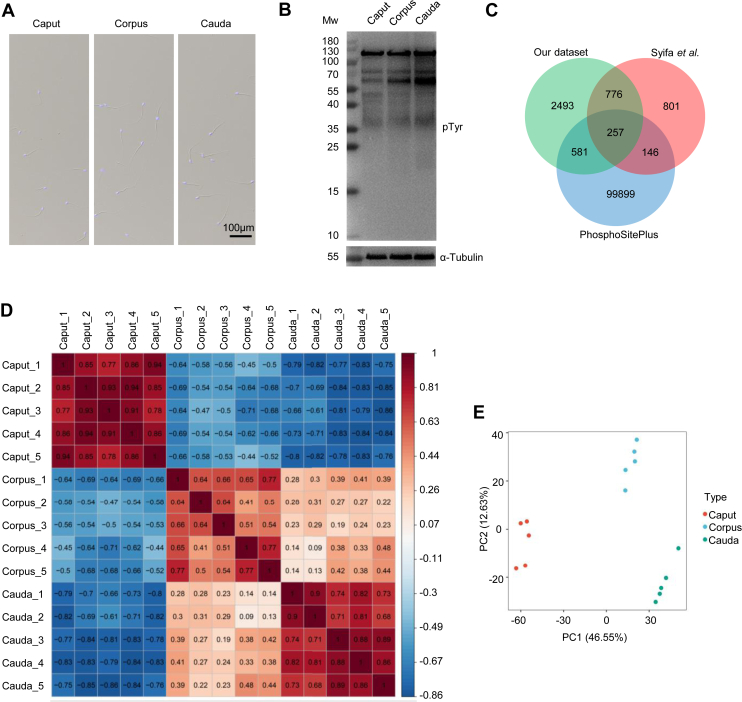
**Quantitative phosphoproteomic characterization of sperm from different epididymal regions.***A*, images were taken to assess sperm purity after isolation and purification of epididymal sperm, bar =100 μm. *B*, immunoblot analysis of protein tyrosine phosphorylation levels in sperm from different epididymal regions. *C*, comparison of class I phosphorylation sites with the PhosphositePlus database and Syifa’s data. *D*, correlation analysis of sperm protein phosphorylation levels. *E*, principal component analysis (PCA) of sperm samples from the caput, corpus, and cauda epididymal regions based on quantitative phosphoproteomic data.

To gain an overall understanding of the dynamics of protein phosphorylation in mouse sperm during epididymal maturation, we isolated sperm with high-purity (>99%) from the caput, corpus and cauda regions of the mouse epididymis (Figs. 1 and 2*A*), and performed quantitative proteomics and phosphoproteomic analysis of sperm proteins in five replicates *via* multiplexed tandem mass tag (TMT) 15-plex labeling, high-pH reverse phase fractionation, Ti^4+^-IMAC enrichment, and liquid chromatography-tandem mass spectrometry (LC-MS/MS) analysis (Fig. 1). Additionally, we evaluated the tyrosine phosphorylation levels of proteins by immunoblotting. We found that the cauda sperm protein showed higher levels of protein tyrosine phosphorylation (Fig. 2*B* and Supplemental Fig. S1), consistent with previous reports (28).

In the quantitative proteomics analysis, a total of 4756 proteins were identified in the mouse sperm during epididymal maturation, and 548 were down-regulated and 408 were up-regulated in cauda sperm (Supplemental Table S2), consistent with Skerget *et al.*’s study revealing significant reduction in proteome content in cauda sperm compared with caput sperm (11). For quantitative phosphoproteomics analysis, a total of 6447 phosphorylation sites corresponding to 6341 phosphopeptides and 1407 phosphoproteins were identified in mouse epididymal sperm (Supplemental Tables S3 and S4). Among the identified phosphorylation sites, 4121 were class I phosphorylation sites with localization probability above 0.75, corresponding to 1205 phosphorylated proteins (Supplemental Table S4). The 4121 Class I sites comprised 89.1% phosphoserine (pS), 10.24% phosphothreonine (pT), and 0.66% phosphotyrosine (pY) with a distribution similar to previous studies of phosphoproteins in germ cells (23). Compared with the PhosphositePlus database (https://www.phosphosite.org) and previous mouse sperm phosphoproteomic studies (29), we identified 2493 new phosphorylation sites that have not been reported before (Fig. 2*C*). Based on the quantification of protein expression and phosphorylation site levels, we performed correlation analysis, and found higher correlation coefficients within groups compared with those between groups at both levels. Sperm from corpus epididymis are more similar to those from cauda epididymis than those from caput epididymis both at the protein expression and phosphorylation levels (Fig. 2*D* and Supplemental Fig. S1). We further performed the principal component analysis (PCA), and found high reproducibility between replicates within each group, and distinct clusters of samples from caput, corpus, and cauda epididymis (Fig. 2*E*), indicating different phosphorylation states among sperm from different regions of the epididymis.

### Functional Analysis of Differentially Regulated Phosphoproteins During Epididymal Sperm Maturation

**Fig. 3:**
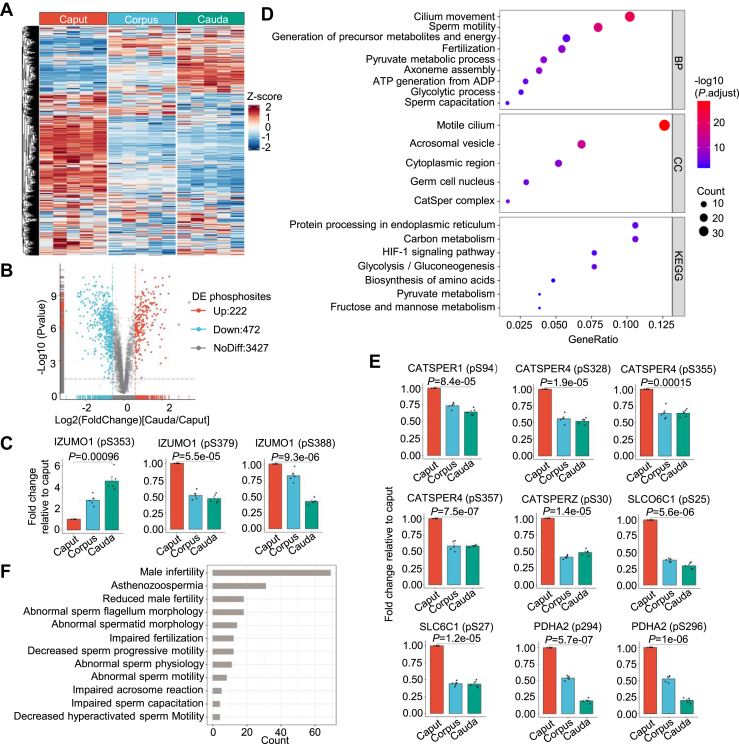
**Functional analysis of differentially regulated phosphoproteins during sperm maturation.***A*, heatmap analysis of Class I phosphorylation sites in sperm from caput, corpus, and cauda epididymal regions. *B*, volcano analysis of differential phosphorylation sites between caput and cauda sperm. *C*, the quantification of differentially regulated phosphorylation sites of Izumo1 during sperm maturation. *D*, gene ontology enrichment analysis and KEGG pathway analysis of differentially phosphorylated proteins. *E*, the quantification of differentially regulated phosphorylation sites of CatSper complex proteins and PDHA2 during sperm maturation. *F*, the distribution of the number of differentially phosphorylated proteins with MGI male reproductive phenotypes.

To evaluate the phosphorylation dynamics during sperm maturation, we performed a heatmap analysis of sperm class I sites protein phosphorylation levels from caput, corpus, and cauda of the mouse epididymis. The results showed that the phosphorylation levels of corpus epididymal sperm were between those of caput and cauda epididymal sperm (Fig. 3*A*). Therefore, we subsequently focused on the analysis of differential phosphorylation sites between caput and cauda epididymal sperm. We found that a total of 2620 class I sites are statistically significant (*p* value < 0.05), of which 870 were up-regulated and 1750 were down-regulated. And 694 class I sites showed changes greater than 1.5 fold (FC ≥1.5, *p* value < 0.05), with 222 upregulated corresponding to 128 proteins and 472 downregulated corresponding to 256 proteins (Fig. 3*B*), and are used for subsequent analysis (Supplemental Table S4). We compared proteins with differential phosphorylation sites with proteins with differential expression levels (Supplemental Tables S2 and S4) and found that only 2 of the 128 proteins with upregulated phosphorylation sites were upregulated at the protein expression level, and only 7 of the 256 proteins with downregulated phosphorylation sites were also downregulated at the protein expression level (Supplemental Fig. S3), suggesting that phosphorylation regulation is largely independent of their abundance during sperm maturation. Interestingly, 39 proteins had both up- and downregulated phosphorylation sites, suggesting that these proteins undergo complex phosphorylation regulation at different locations in the epididymis. For example, Izumo1 has two downregulated phosphorylation sites (pS379 and pS388) and one upregulated phosphorylation site (pS353) (Fig. 3*C*). Previous studies have shown that the phosphorylation of IZUMO1 in sperm is altered during the maturation of the sperm in rat and mouse (18, 30), indicating high confidence of our quantitative phosphoproteomics data.

To further characterize the functions of differentially phosphorylated sperm proteins, we performed gene ontology enrichment analysis (Supplemental Table S6). We observed the enrichment of terms related to sperm motility and energy metabolism, including cilium movement, sperm motility, generation of precursor metabolites and energy, pyruvate metabolic process, ATP generation from ADP, and glycolytic process (Fig. 3*D*). These proteins may be involved in sperm motility acquisition in cauda epididymis. Besides, we also observed enrichment of terms of sperm capacitation and fertilization. Matured sperm from cauda epididymis but not those from the testis can fertilize the egg. Proteins composing the cation channel of sperm (CatSper) showed downregulated phosphorylation levels at pS94 of CATSPER1, pS328, pS355, and pS357 of CATSPER4, and pS30 of CATSPERZ (Fig. 3*E*). CatSper proteins are responsible for multiple Ca^2+^-dependent physiological responses during fertilization (31). Recently, SLCO6C1 was identified as a novel component of the CatSper (32). Our data showed significantly down-regulated levels of pS25 and pS27 of SLCO6C1. These regulated phosphorylation sites may regulate the activity of CatSper in sperm and deserve further functional studies.

We further performed KEGG pathway analysis of differentially phosphorylated proteins during sperm maturation (Supplemental Table S7), and found enrichment in the glycolysis/gluconeogenesis pathway and pyruvate metabolism pathway (Fig. 3*D*). Glycolysis plays an important role in providing ATP for sperm motility along the entire length of the sperm flagellum (33), and pyruvate has been reported to enhance glycolytic ATP production, motility, hyperactivation, and capacitation in sperm (34). It seems that during sperm maturation glycolytic enzymes and pyruvate metabolism-related enzymes are subjected to phosphorylation regulation.

To analyze the *in vivo* functions of proteins in mice, we annotated phenotypes of the differentially phosphorylated proteins according to the MGI database. The results showed that proteins with altered levels of phosphorylation during sperm maturation in the epididymis were closely related to male infertility and sperm abnormalities. In total, 69 proteins were annotated to male infertility, 31 proteins were annotated to asthenozoospermia, and 18 proteins were annotated to abnormal sperm flagellum morphology (Fig. 3*F*). The phosphorylation changes of these proteins essential for male fertility indicated potential important regulations of phosphorylation in sperm maturation.

### Kinase Analysis of Sperm Phosphoproteome During Epididymal Sperm Maturation

**Fig. 4:**
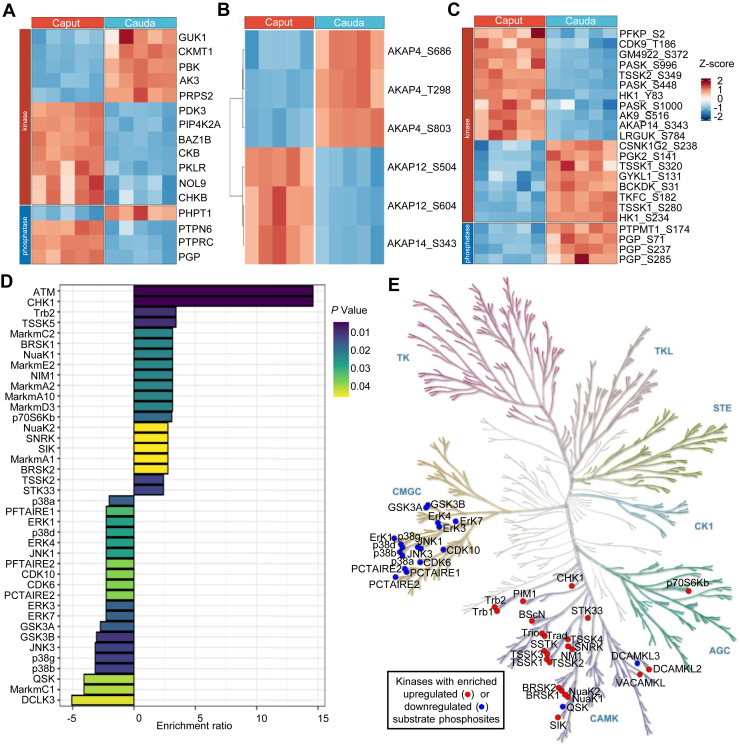
**Kinase analysis of sperm phosphoproteome during epididymal maturation.***A*, differentially regulated expression of kinases and phosphatases in sperm during maturation. *B*, differentially regulated phosphorylation sites of AKAPs in sperm during maturation. *C*, differentially regulated phosphorylation sites of kinases and phosphatases during sperm maturation. *D*, the top 20 kinases with enriched phosphorylation substrates in sperm from cauda epididymis (*p* < 0.05). *E*, enrichment of CAMK family kinases with upregulated phosphorylation sites (*red*) and CMGC family kinases with downregulated sites (*blue*) during sperm maturation.

Protein phosphorylation is a reversible dynamic process that is regulated by the competing activities of protein kinases and phosphatases (35). With the quantitative sperm proteome and phosphoproteome, we analyzed the expression and phosphorylation levels of kinases and phosphatases in sperm. In our quantitative proteomic dataset of sperm epididymal maturation, we found that among the quantified 124 kinases and 44 phosphatases in sperm, only 12 kinases and 4 phosphatases showed differential expression during sperm maturation from caput to cauda epididymis (Fig. 4*A*, Supplemental Table S2). Notably, pyruvate dehydrogenase kinase isoform 3 (PDK3) was found to be downregulation in cauda sperm (Fig. 4*A*). It was reported to be able to inhibit pyruvate dehydrogenase activity by phosphorylating the S294, S296, and S301 of PDHA2 and inhibit glucose metabolism and aerobic respiration (36). PDHA2 is a testis-specific subunit of pyruvate dehydrogenase and was found to be downregulated at pS294 and pS296 during sperm maturation (Fig. 3*E*).

It is well known that temporal and spatial regulation of cAMP-dependent protein kinase A (PKA) activity is essential for sperm motility (37). AKAPs are a class of kinase anchoring proteins that bind to regulatory subunits of the PKA and target kinase holoenzymes to specific subcellular compartments (38). During sperm epididymal maturation, we found that pT298, pS803, and pS686 of AKAP4 were upregulated, whereas pS343 of AKAP14 and pS504 and pS604 of AKAP12 were downregulated. These phosphorylation sites may be involved in regulating the functions of AKAPs or the interaction between AKAPs and PKA (Fig. 4*B*).

Kinases and phosphatases may also be regulated by phosphorylation (39, 40), we analyzed phosphorylation changes of kinases and phosphatases in our quantitative phosphoproteomic dataset during sperm maturation from caput to cauda epididymis. The results showed that among 117 phosphorylation sites from 49 kinases and 24 phosphorylation sites from 17 phosphatases, 19 sites from 15 kinases and 4 sites from 2 phosphatases showed significant changes (Fig. 4*C*, Supplemental Table S4). Phosphoglycerate kinase 2 (PGK2) catalyzes the first step in the glycolytic pathway to produce ATP, which is essential for sperm motility. It has been reported that PGK2 deficiency in mice does not affect the process of spermatogenesis, but reduces sperm ATP levels and motility (41). Although the expression of PGK2 showed no change, we found a significant increase in the phosphorylation level of S141 of PGK2, suggesting a potential role of PGK2 phosphorylation in the upregulation of glycolysis in epididymal sperm maturation.

Kinases often recognize certain motifs to phosphorylate their specific substrate proteins (25). With the sequences surrounding phosphorylation sites, it is possible to annotate the kinase-substrate phosphorylation relationship. We used iGPS to predict the kinase-substrate relationship and to identify kinases with enriched phosphorylation substrates (Fig. 4*D* and Supplemental Table S8). The results showed that most kinases with enriched phosphorylation sites upregulated during sperm maturation belonged to the CAMK family, whereas most kinases with enriched phosphorylation sites down-regulated during sperm maturation belonged to the CMGC family (Fig. 4*E*). GSK3 is a kinase from the CMGC family, and it is the top 10 kinases with enriched phosphorylation sites down-regulated during sperm maturation. Consistently, GSK3 activity was reported to decrease in bovine sperm from caput to cauda epididymis (42). In humans, GSK3 activity was negatively correlated with sperm motility (43). These kinases with enriched regulated phosphorylation substrates may play important roles in sperm functional maturation.

### Inhibition of TSSK2 Suppresses Sperm Motility but not Energy Production

As the kinases with enriched regulated phosphorylation substrates in the kinase-substrate analysis may regulate sperm functions, we further analyzed the expression and available inhibitors of the predicted kinases. We found that STK33 and TSSK2 were identified in our sperm proteome, and have specific inhibitors for functional experiments. Deletion or mutations of both STK33 and TSSK2 in mice result in abnormal sperm structure leading to male sterility (21, 44, 45); however, it is still not known whether they regulate motility acquisition during sperm maturation in epididymis.

We collected sperm from the cauda epididymis, and incubated the sperm with ML281 or ALK inhibitor 2 compound 18, inhibitor of STK33, and TSSK2 kinase, respectively. ML281 is a potent and selective inhibitor of STK33 kinase with an IC50 of 14 nM (46). ALK inhibitor 2 compound 18 is a potent inhibitor of TSSK2 with an IC50 of 37 nM and FAK with an IC50 of 5 nM (47), because FAK was not identified in the mouse sperm proteome or phosphoproteome, ALK inhibitor 2 compound 18 was used as specific inhibitor of TSSK2 in sperm.

**Fig. 5:**
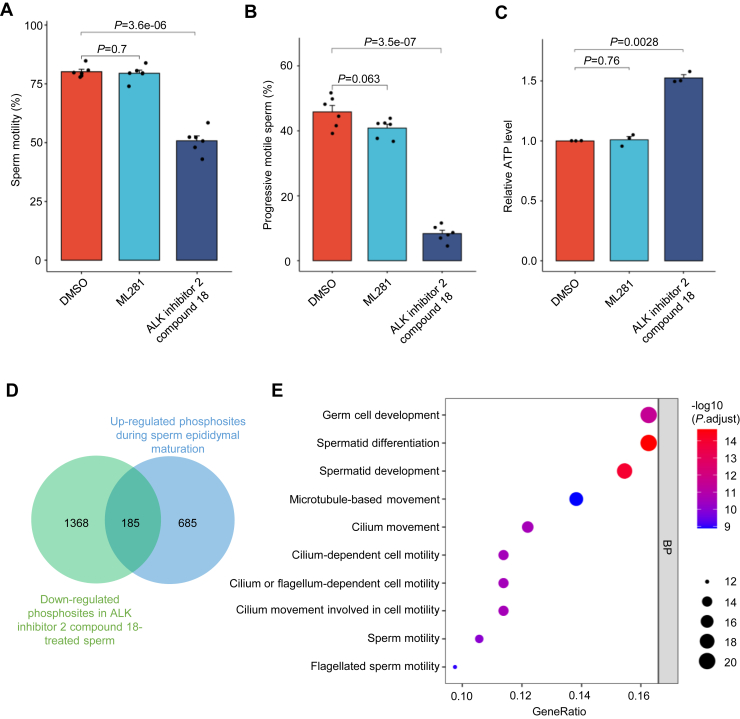
**Inhibition of TSSK2 kinase affects sperm motility or progressive motility but not energy production.***A* and *B*, Sperm motility (*A*) and progressive motility (*B*) assay using ML281 or ALK inhibitor 2 compound 18 to inhibit STK33 or TSSK2, respectively, with DMSO as a control. Sperm from cauda epididymis were used. *C*, sperm ATP levels after treatment with ML281 and ALK inhibitor 2 compound 18. *D*, comparison of down-regulated phosphorylation sites (*p* value < 0.05) in ALK inhibitor 2 compounds 18-treated sperm with up-regulated phosphorylation sites (*p* value < 0.05) during sperm epididymal maturation in the epididymis. *E*, gene ontology enrichment analysis of proteins with phosphorylation sites up-regulated during sperm epididymal maturation and down-regulated in ALK inhibitor 2 compound 18-treated sperm

We observed that ML281 treatment did not affect sperm motility or progressive motility (*p* > 0.05). Although STK33 is essential for sperm tail structure (21), it sees that its kinase activity may not be involved in sperm motility acquisition in epididymis. ALK inhibitor 2 compound 18 treatment significantly reduced both sperm motility (50.8% *versus* 80.18%, *p* < 0.001) and progressive motility (8.28% *versus* 45.83%, *p* < 0.001) (Fig. 5, *A* and *B*), indicating important roles of TSSK2 kinase in sperm motility acquisition.

ATP is essential for sperm motility or progressive motility. We measured the levels of ATP, and found that ATP in ALK inhibitor 2 compound 18-treated sperm (*p* < 0.01) did not decrease but increased to varying degrees (Fig. 5*C*). The suppression of sperm motility or progressive motility is not caused by ATP deficiency. TSSK2 may regulate the functions of components of flagellar structure during sperm maturation in epididymis.

To gain further insights into the potential mechanisms by which TSSK2 regulates sperm motility, we performed phosphoproteomic analysis of ALK inhibitor 2 compound 18-treated and control sperm. As a result, we observed 1553 down-regulated and 325 up-regulated phosphorylation sites (*p* value＜0.05) after 1 h incubation with ALK inhibitor 2 compound 18. Most of the differentially regulated phosphorylation sites were downregulated (1553/1878, 82.7%) indicated high efficacy of the TSSK2 kinase inhibitor (Supplemental Table S9). Among those 870 up-regulated phosphorylation sites (*p* value＜0.05) during sperm epididymal maturation, we found that 185 (21.3%, 185/870) were downregulated by TSSK2 inhibition, indicating important contribution of TSSK2 kinase activity in phosphorylation upregulation during sperm maturation (Fig. 5*D*). We performed gene ontology enrichment analysis and observed enrichment of terms related to sperm motility (Fig. 5*E*, Supplemental Table S10), which was also consistent with the motility inhibition phenotype after TSSK2 inhibition. These motility-related proteins might be involved in the signaling pathways regulated by TSSK2 during sperm maturation.

## Discussion

Sperm in epididymis is transcriptionally silent and translationally suppressive, the regulation of protein phosphorylation in sperm maturation in mouse epididymis is still not well known. We systemically characterized the phosphoproteomes of sperm, and identified 694 Class I differentially regulated phosphorylation sites corresponding to 345 phosphoproteins as the sperm moved from the caput to the cauda of the epididymis. These regulated phosphoproteins are enriched in sperm motility and energy metabolism. Kinase-substrate relationship analysis followed by functional studies confirmed the important regulatory roles of TSSK2 and kinase in sperm motility regulation.

Our gene ontology and KEGG pathway enrichment analysis of differentially phosphorylated proteins during sperm maturation showed significant enrichment of phosphoproteins in the glycolytic process, pyruvate metabolism, sperm motility, and motile cilium regulation. Glycolysis was shown to play a major role in ATP supplement in mouse sperm flagellar movement (33). Pyruvate, a product of glycolysis, promotes glycolytic ATP production, and capacitation (34, 48). The complete glycolytic pathway consists of multiple chemical reaction steps, and each step is catalyzed by specific enzymes, including hexokinase, phosphoglucose isomerase, and phosphofructokinase (49). Pyruvate metabolism is similarly regulated by multiple enzymes. We observed changes in the phosphorylation levels of 13 phosphorylation sites of 8 of these enzymes, including LDHA, LDHC, ALDOA, PGK2, HK1, PDHA1, PDHA2, and PFKP. The enzymatic activity of PDHA1/2 has been reported to be negatively correlated with the level of phosphorylation, However, the exact roles of these phosphorylation sites in sperm maturation still deserve further investigation (36).

The flagellum contains 9 + 2 microtubule axoneme and fibrous sheath structures, which are the structural basis of sperm motility. Mutations in a number of structural proteins, such as cilia and flagella-associated protein 44/70 (CFAP44/70) (50, 51), dynein axonemal heavy chain 8 (DNAH8) (52) and fibrous sheath-interacting protein 2 (FSIP2) (53), have been reported to cause impaired sperm motility. We observed 85 phosphorylation sites of 39 flagellum-related proteins with altered phosphorylation levels during sperm maturation, including pS1047 and pS1312 of CFAP44, pS403 of CFAP70, pT83, pS84, pS125 and pS141 of DNAH8, pS426, pS373, pT38, and pT473 of FSIP2. Further functional studies of these energy metabolism-related and structural proteins may help elucidate the molecular basis of flagellar motility acquisition in epididymis.

Kinases typically recognize specific sequence motifs surrounding phosphorylation sites, which enabled the identification of 50 kinases with enriched downstream regulated phosphorylation sites. Our inhibitor assay of STK33 and TSSK2 *in vitro* showed important roles of TSSK2 in sperm progressive motility, without down-regulation of ATP levels. Although sperm motility was not affected after STK33 inhibition, our recent study showed that sperm from STK33 knockout mice were unable to induce oocyte activation (21). These kinases may have important functions in sperm maturation in epididymis. Further phosphoproteomics analysis showed that a high proportion (21.3%) of the proteins with phosphorylation level upregulated during sperm epididymal maturation could be downregulated by TSSK2 kinase inhibitor, indicating important roles of TSSK2 kinase in phosphoproteome dynamics and sperm motility acquisition during sperm epididymis maturation. However, whether phosphorylation affects the function of these proteins deserves further investigation.

In conclusion, we performed in-depth phosphoproteomic analysis of mouse sperm epididymal maturation and identified complex dynamic phosphorylation regulation in energy metabolism and flagellar structure proteins. Kinase substrate phosphorylation network and functional assay revealed important roles of TSSK2 kinase in sperm motility regulation. Further functional studies of differentially regulated phosphorylation sites may help us elucidate sperm maturation, and provide new candidate targets for male contraception as well as new insights into the treatment of asthenozoospermia, an important cause of male infertility. Although our study is the most comprehensive phosphoproteome of sperm maturation to date, limited by the low number of sperm from caput and corpus and limited protein amount for phosphoproteomics analysis, the phosphoproteome coverage of sperm epididymal maturation is still relatively limited, and only 810 of the 4756 identified mouse sperm proteins have class I phosphorylation sites identified in the phosphoproteome. In the future, the development of more sensitive phosphoproteomics technologies is anticipated to enhance the comprehensiveness of phosphoproteome of sperm maturation, thereby to help better characterize phosphorylation regulation underlying the sperm epididymal maturation.

## Data Availability

The proteomics data have been deposited in the ProteomeXchange Consortium *via* the proteomics identifications (PRIDE) database (Identifier: PXD049067), reviewer account username: reviewer_pxd049067@ebi.ac.uk, password: ThljWQWf. Annotated spectra can be visualized by using MS-Viewer (https://msviewer.ucsf.edu/prospector/cgi-bin/msform.cgi?form=msviewer) with the search keys hzxhvat1ph and 73q98epndd for phosphoproteomic and proteomic datasets of sperm epididymal maturation, and the search keys of qgrqhou6te and rfmgexe6ug for phosphoproteomic and proteomic datasets of ALK inhibitor 2 compound 18- treated sperm.

## Supplemental data

This article contains supplemental data.

## Conflict of interest

The authors declare that they have no conflicts of interest with the contents of this article.
