# Supplementary material for: Quantitative Phosphoproteomic Profiling of Mouse Sperm Maturation in Epididymis Revealed Kinases Important for Sperm Motility
Source: Mol Cell Proteomics. 2024 Jul 6;23(8):100810. doi: 10.1016/j.mcpro.2024.100810 (PMC11338950; doi:10.1016/j.mcpro.2024.100810)
Supplement: Supplemental Table S5 [file mmc5.pdf]

Table S5. Annotated MS/MS spectra of peptides from proteins identified based on a single peptide.

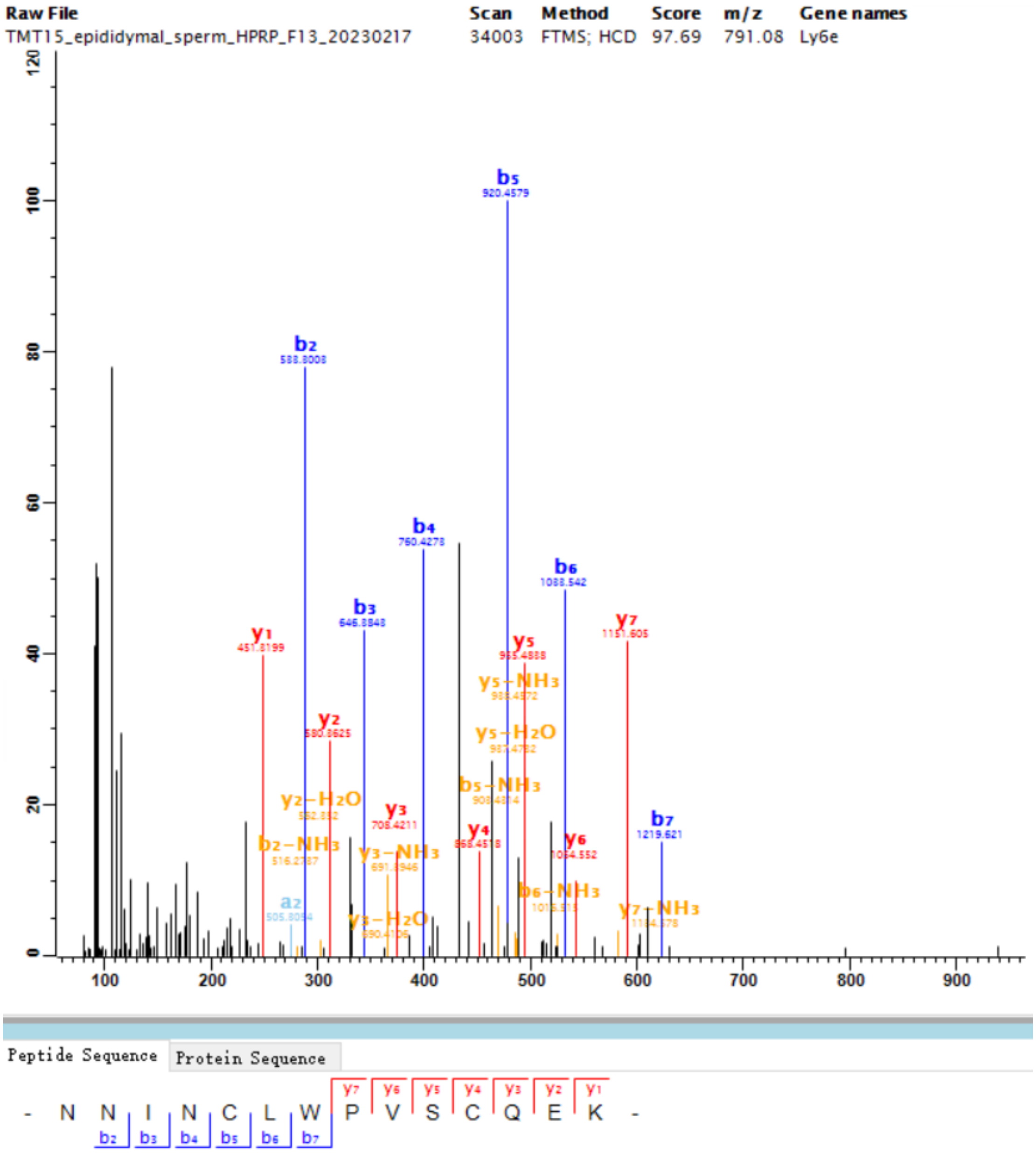

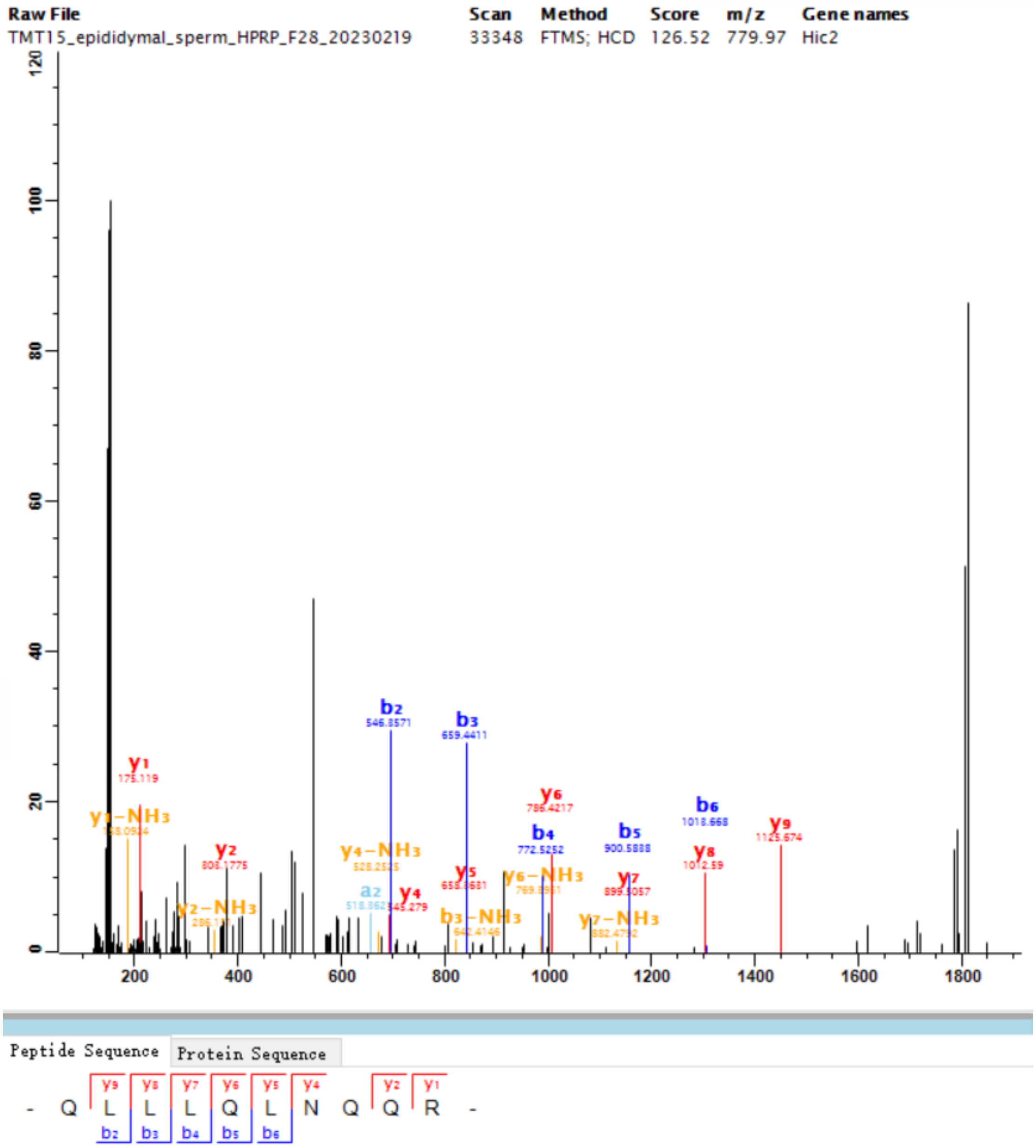
Raw File
TMT15_epididymal_sperm_HPRP_F28_20230219
Scan Method Score m/z Gene names
33348 FTMS; HCD 126.52 779.97 Hic2
b2 546.8571
b3 659.4411
y1 175.119
y6 786.4217
b6 1018.668
b4 772.5252
b5 900.5888
y9 1125.674
y2
y4
y5
y7
y8
a2
y1-NH3
y2-NH3
y4-NH3
y6-NH3
y7-NH3
b3-NH3
Peptide Sequence
Protein Sequence
- Q L L L Q L N Q Q R -

Raw File	Scan	Method	Score	m/z	Gene names
TMT15_epididymal_sperm_HPRP_F21_20230219	31572	FTMS; HCD	106.04	1193.88	Spink8

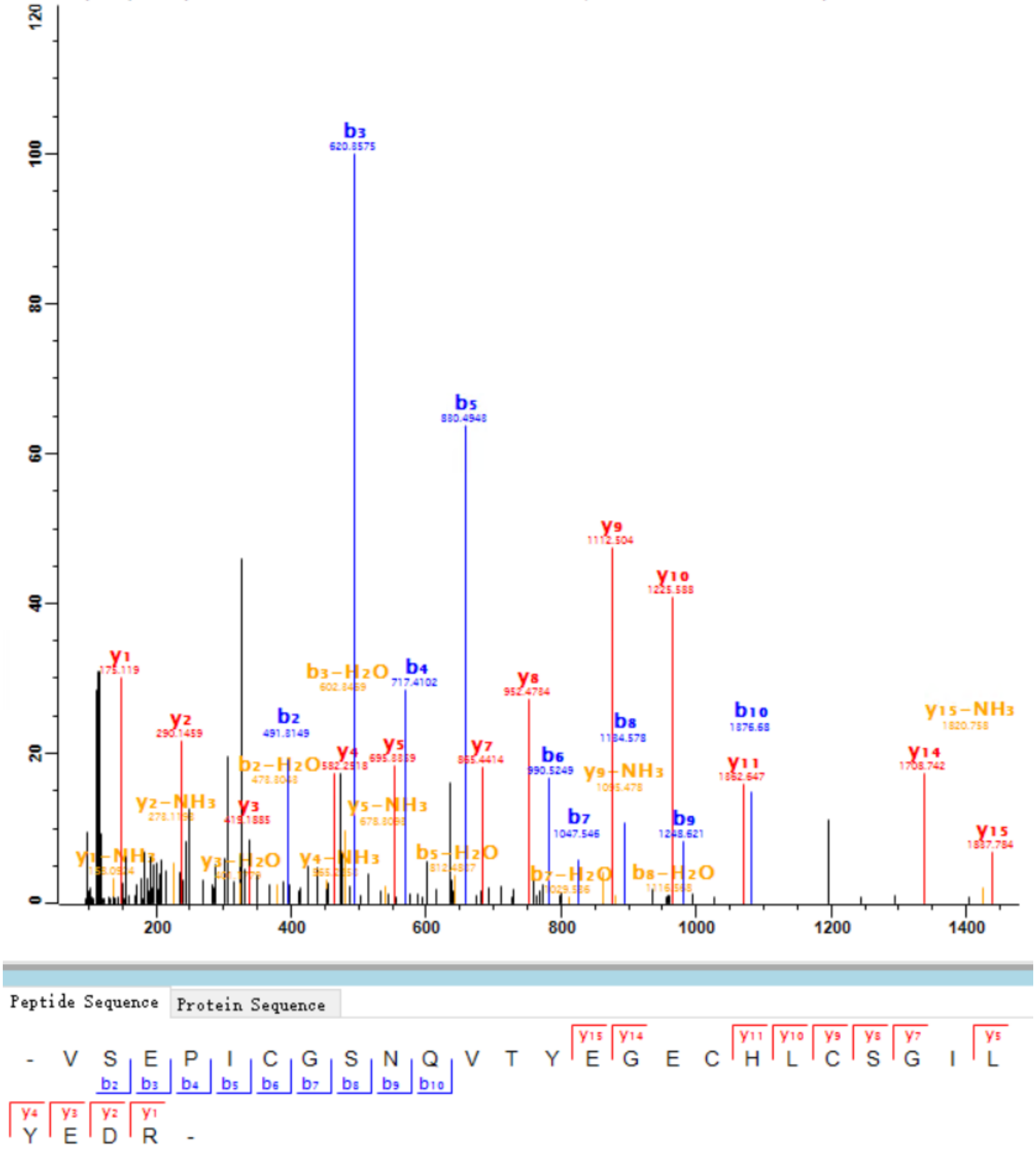

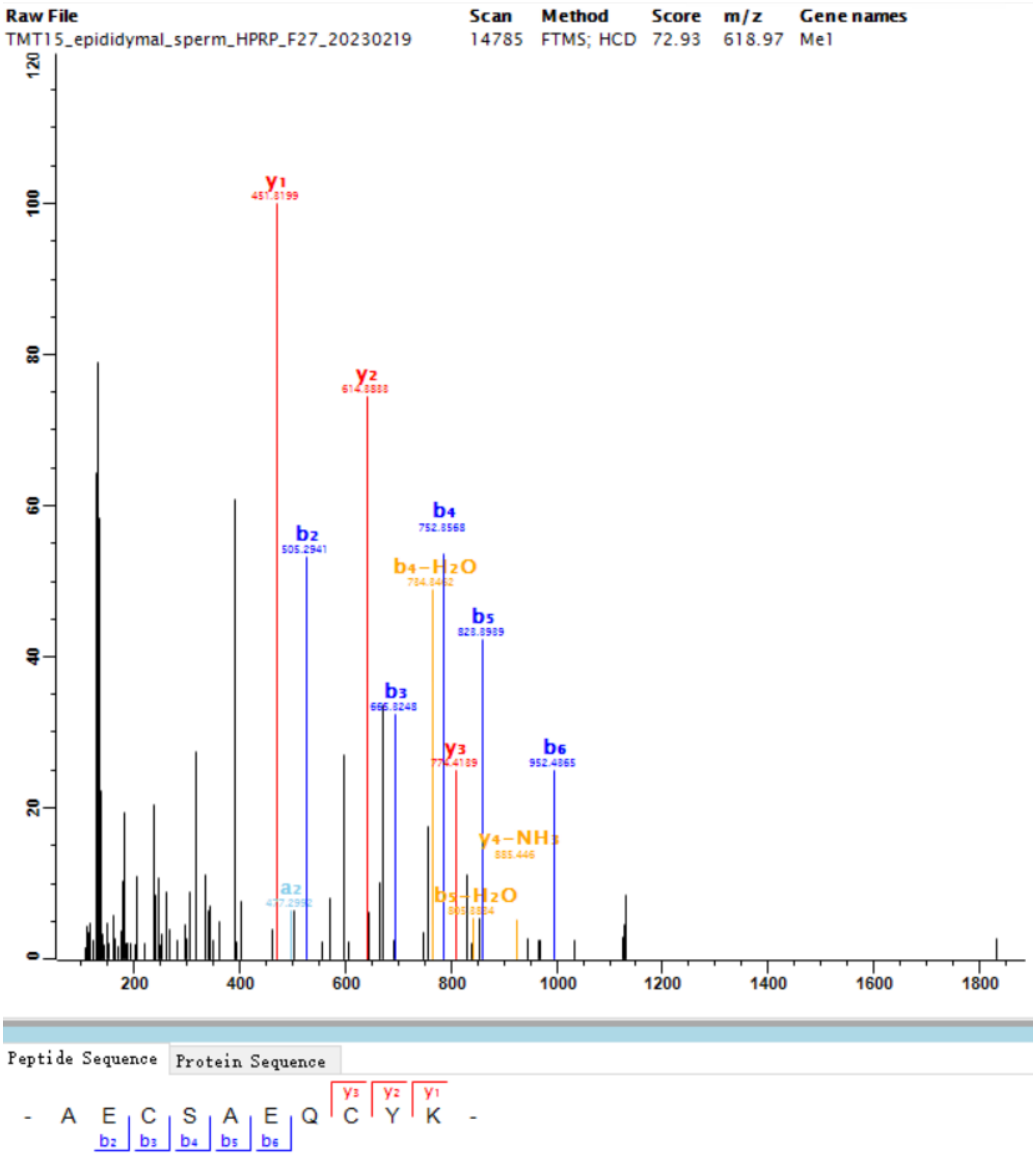
Raw File
TMT15_epididymal_sperm_HPRP_F27_20230219
Scan
14785
Method
FTMS; HCD
Score
72.93
m/z
618.97
Gene names
Me1
y1
451.8199
y2
614.8888
b2
505.2941
b4
752.8568
b4-H2O
784.8462
b5
828.8989
b3
y3
774.4189
b6
952.4865
y4-NH3
885.446
a2
b5-H2O
0
20
40
60
80
100
120
200
400
600
800
1000
1200
1400
1600
1800
Peptide Sequence
Protein Sequence
- A E C S A E Q C Y K -
y3 y2 y1
b2 b3 b4 b5 b6

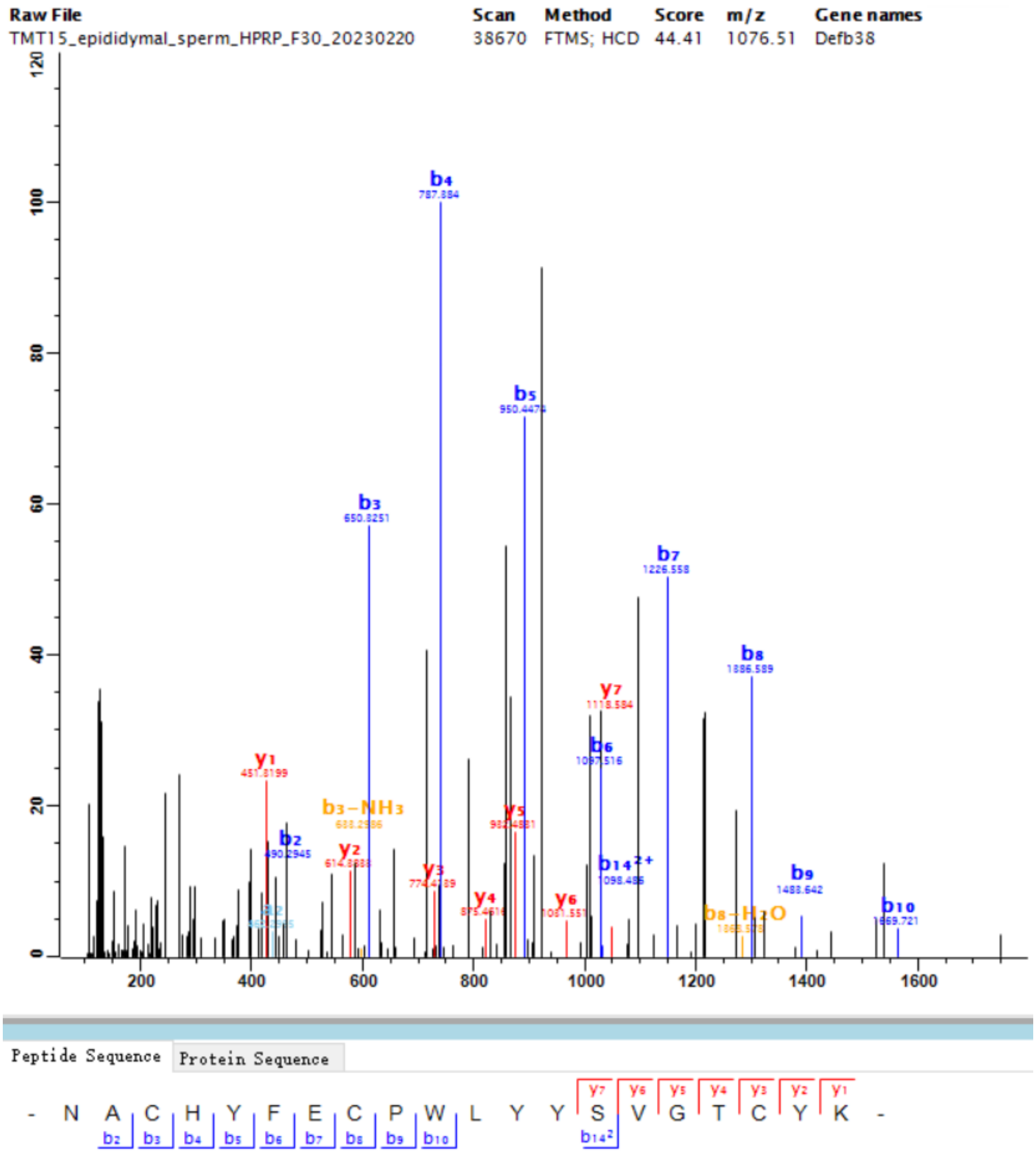
Raw File
TMT15_epididymal_sperm_HPRP_F30_20230220
Scan
38670
Method
FTMS; HCD
Score
44.41
m/z
1076.51
Gene names
Defb38
b4
787.884
b5
950.4474
b3
650.8251
b7
1226.558
b8
1386.589
y7
1118.584
b6
1097.516
y1
451.8199
b3-NH3
b2
490.2945
y2
y5
y3
y4
y6
b14 2+
1098.486
b9
1488.642
b10
b8-H2O
0
20
40
60
80
100
120
200
400
600
800
1000
1200
1400
1600
Peptide Sequence
Protein Sequence
- N A C H Y F E C P W L Y Y S V G T C Y K -
y7 y6 y5 y4 y3 y2 y1
b2 b3 b4 b5 b6 b7 b8 b9 b10
b14 2

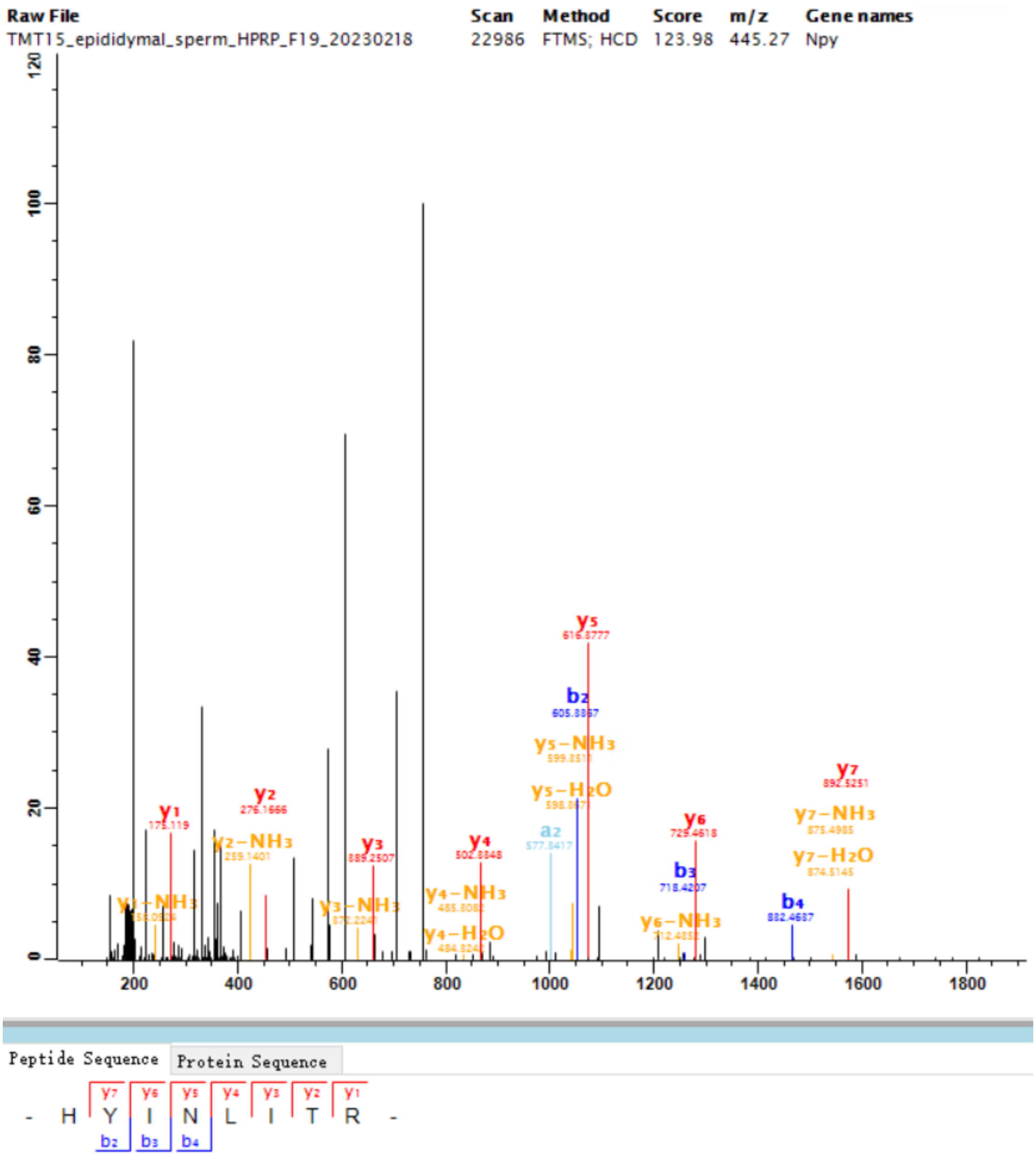

Raw File
TMT15_epididymal_sperm_HPRP_F19_20230218
Scan
22986
Method
FTMS; HCD
Score
123.98
m/z
445.27
Gene names
Npy
0
20
40
60
80
100
120
200
400
600
800
1000
1200
1400
1600
1800
y1
y2
y2-NH3
y3
y3-NH3
y4
y4-NH3
y4-H2O
a2
b2
y5
y5-NH3
y5-H2O
y6
y6-NH3
b3
b4
y7
y7-NH3
y7-H2O
Peptide Sequence
Protein Sequence
- H Y I N L I T R -

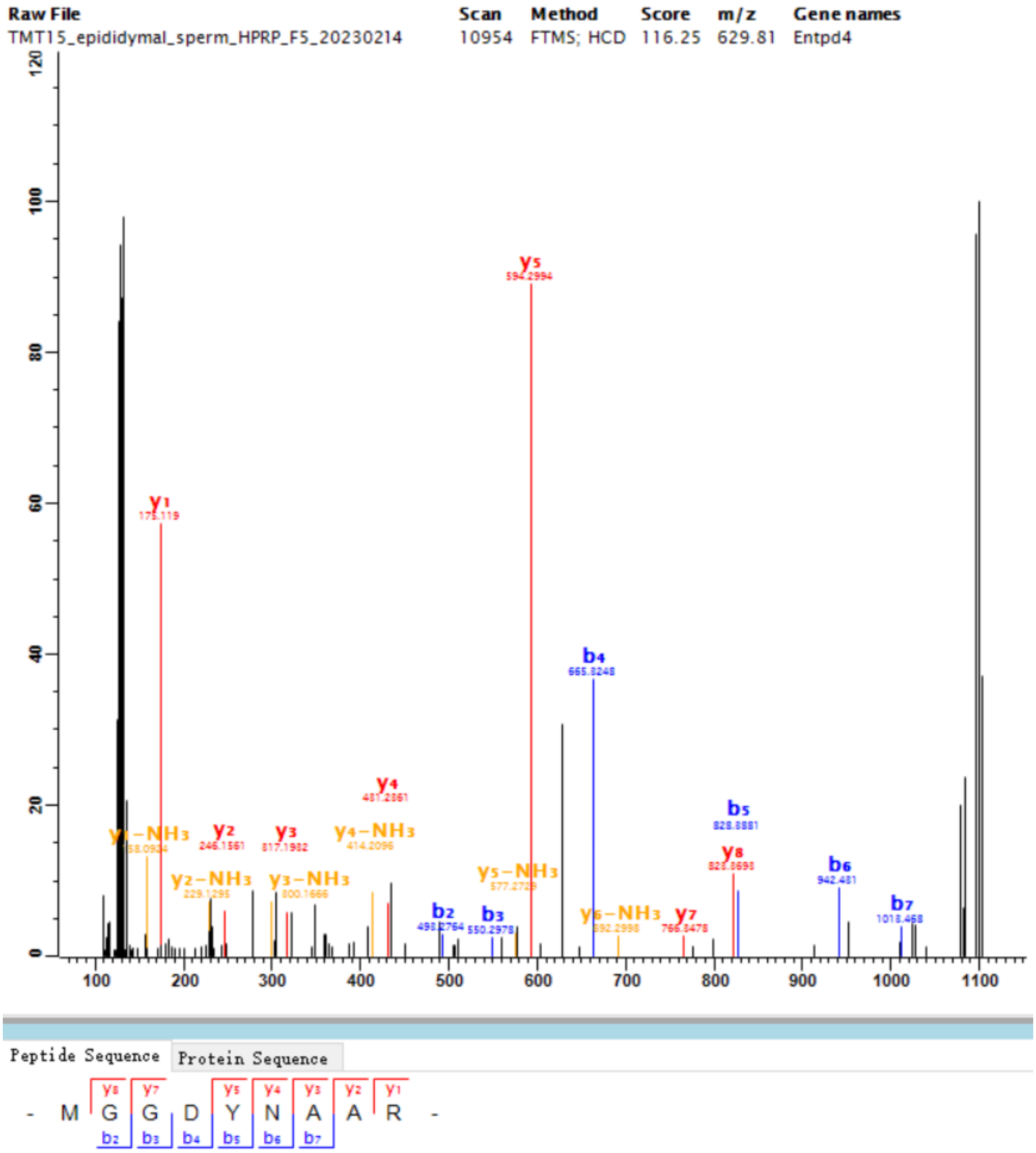

Raw File
TMT15_epididymal_sperm_HPRP_F5_20230214
Scan
10954
Method
FTMS; HCD
Score
116.25
m/z
629.81
Gene names
Entpd4
y5
594.2994
y1
175.119
b4
665.8248
y4
431.2861
y2
246.1561
y3
317.1982
y1-NH3
y4-NH3
414.2096
y2-NH3
229.1295
y3-NH3
300.1666
y5-NH3
577.2729
b5
828.8881
y8
y6-NH3
692.2998
y7
b6
942.481
b7
b2
b3
550.2978
Peptide Sequence
Protein Sequence
- M G G D Y N A A R -
y8 y7 y5 y4 y3 y2 y1
b2 b3 b4 b5 b6 b7

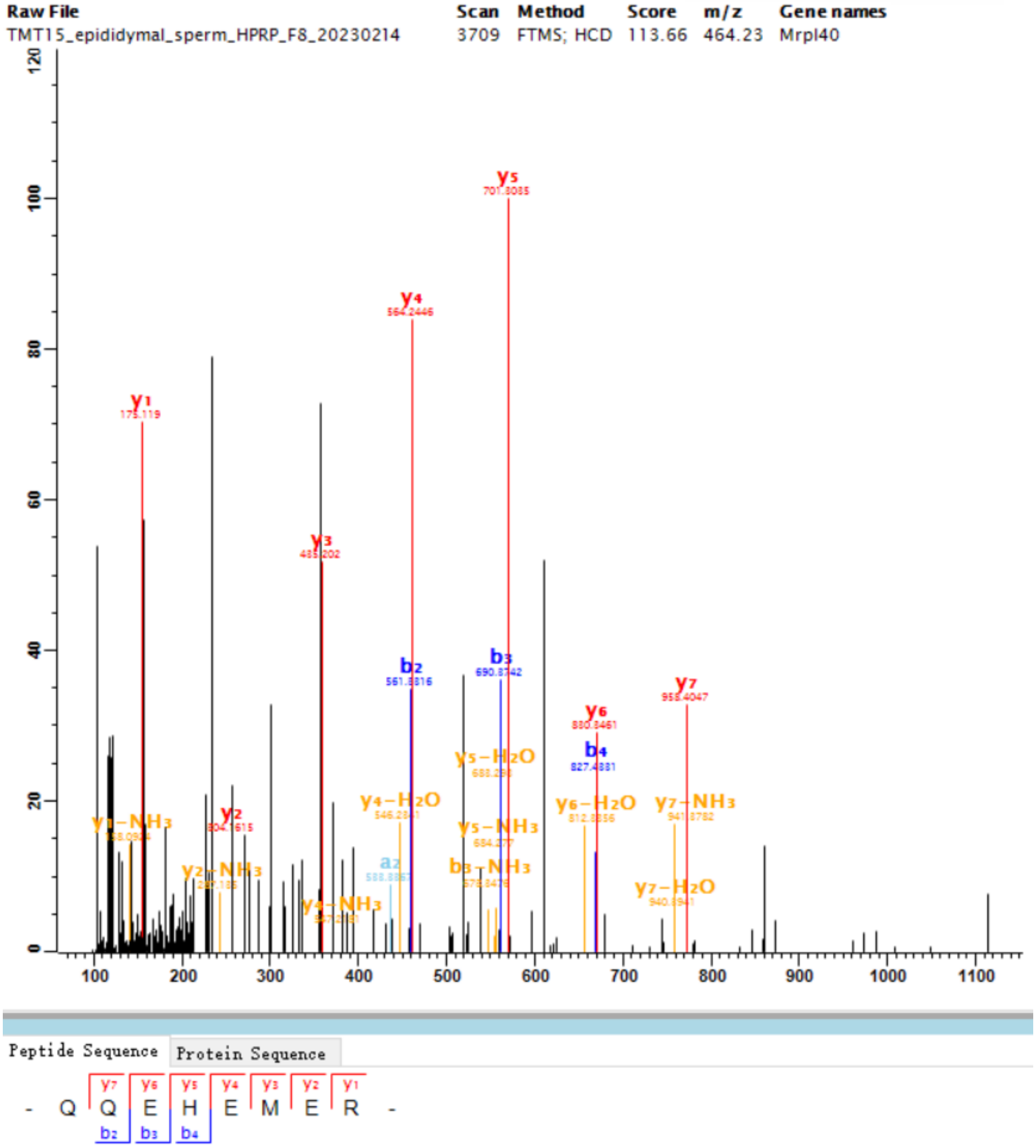

Raw File
TMT15_epididymal_sperm_HPRP_F8_20230214
Scan Method Score m/z Gene names
3709 FTMS; HCD 113.66 464.23 Mrpl40
y1
y2
y3
y4
y5
y6
y7
b2
b3
b4
a2
y1-NH3
y2-NH3
y4-NH3
y4-H2O
y5-H2O
y5-NH3
b3-NH3
y6-H2O
y7-NH3
y7-H2O
Peptide Sequence
Protein Sequence
- Q Q E H E M E R -

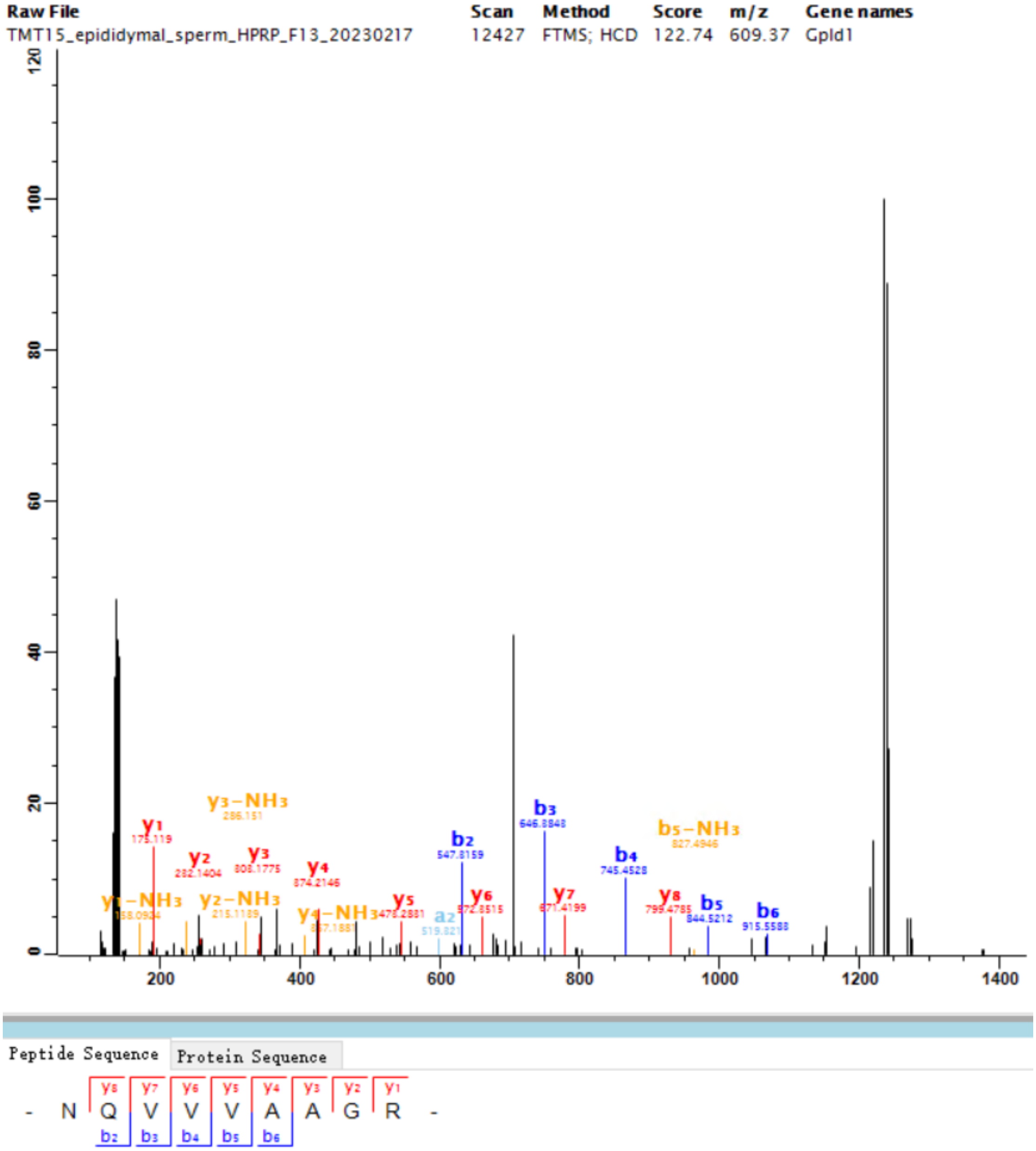

Raw File
TMT15_epididymal_sperm_HPRP_F13_20230217
Scan
12427
Method
FTMS; HCD
Score
122.74
m/z
609.37
Gene names
Gpld1
y1
175.119
y2
282.1404
y3
y4
y5
y6
y7
y8
y1-NH3
y2-NH3
y3-NH3
y4-NH3
a2
b2
547.8159
b3
b4
745.4528
b5
844.5212
b6
b5-NH3
827.4946
Peptide Sequence
Protein Sequence
- N Q V V V A A G R -

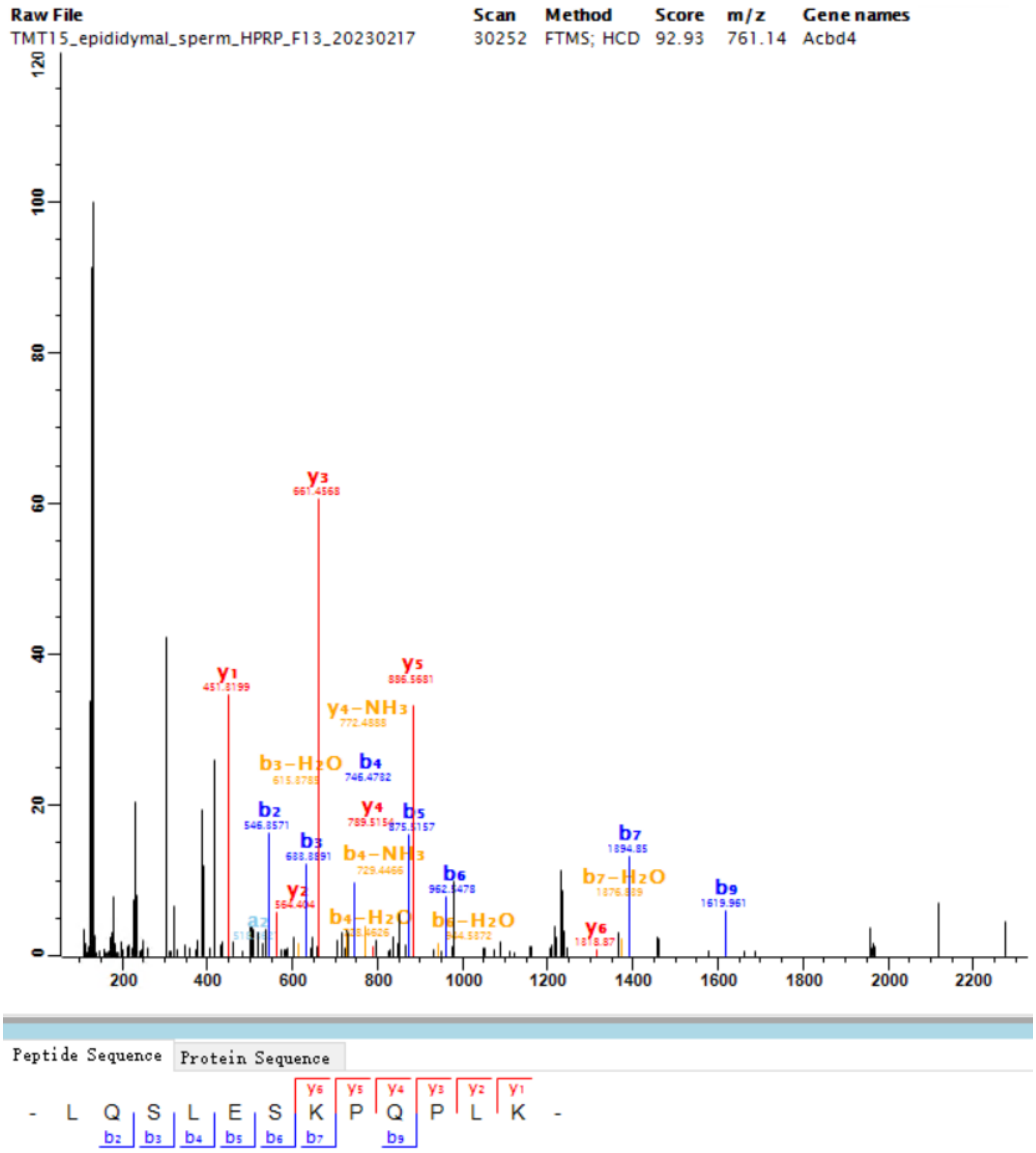

Raw File
TMT15_epididymal_sperm_HPRP_F13_20230217
Scan
30252
Method
FTMS; HCD
Score
92.93
m/z
761.14
Gene names
Acbd4
y3
y1
y5
y4-NH3
b3-H2O
b4
b2
y4
b5
b3
b4-NH3
b6
b7
b7-H2O
b9
y2
b4-H2O
b6-H2O
y6
a2
Peptide Sequence
Protein Sequence
- L Q S L E S K P Q P L K -

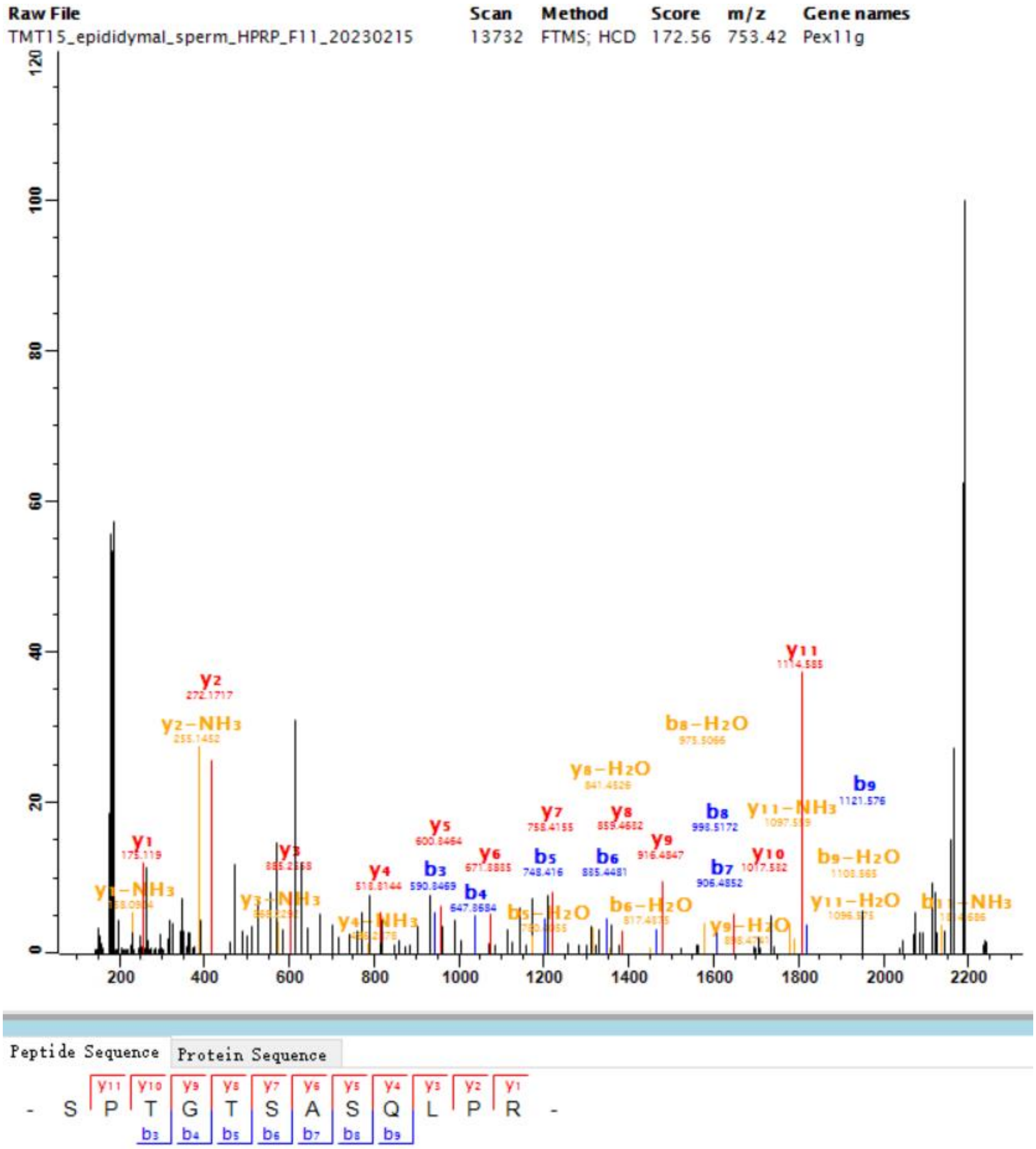
Raw File
TMT15_epididymal_sperm_HPRP_F11_20230215
Scan
13732
Method
FTMS; HCD
Score
172.56
m/z
753.42
Gene names
Pex11g
y2 272.1717
y2-NH3 255.1452
y11 1114.585
b8-H2O 975.5066
y8-H2O 841.4526
b9 1121.576
y11-NH3
b8 998.5172
y7 758.4155
y8 859.4682
y5 600.3464
y1 175.119
y3 385.2358
y4 518.8144
b3 590.3469
y6 671.3835
b5 748.416
b6 885.4481
y9 916.4847
b7 906.4852
y10 1017.532
b9-H2O 1103.565
b4 647.3684
y1-NH3
y3-NH3
y4-NH3
b5-H2O
b6-H2O
y9-H2O
y11-H2O 1096.575
b11-NH3
Peptide Sequence
Protein Sequence
- S P T G T S A S Q L P R -

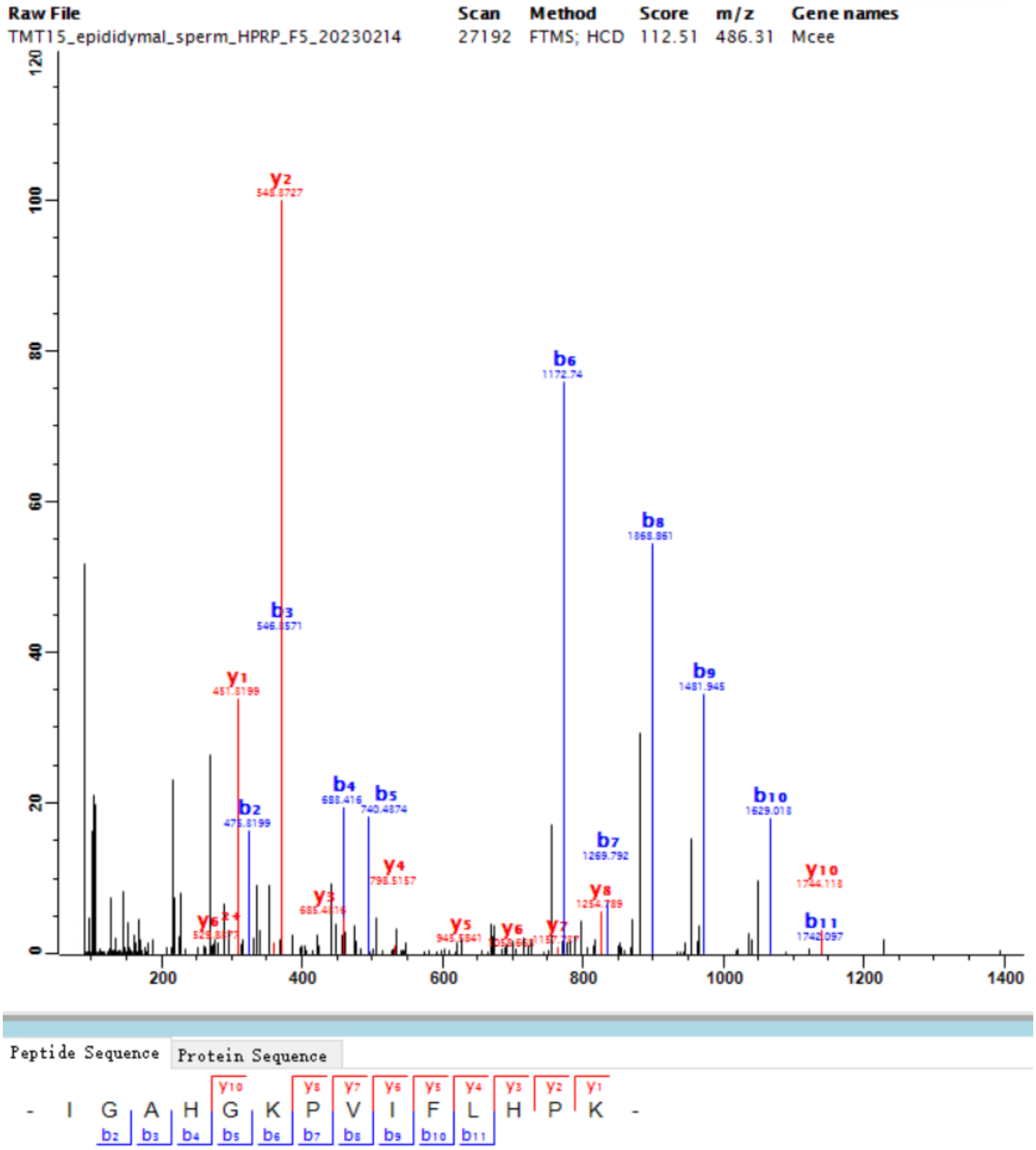

Raw File
TMT15_epididymal_sperm_HPRP_F5_20230214
Scan
27192
Method
FTMS; HCD
Score
112.51
m/z
486.31
Gene names
Mcee
y2
b6
1172.74
b8
1368.861
b3
b9
1481.945
y1
b4
b5
740.4874
b2
b10
1629.018
b7
1269.792
y4
798.5157
y10
1744.118
y3
y8
b11
y5
y6
y7
Peptide Sequence
Protein Sequence
- I G A H G K P V I F L H P K -

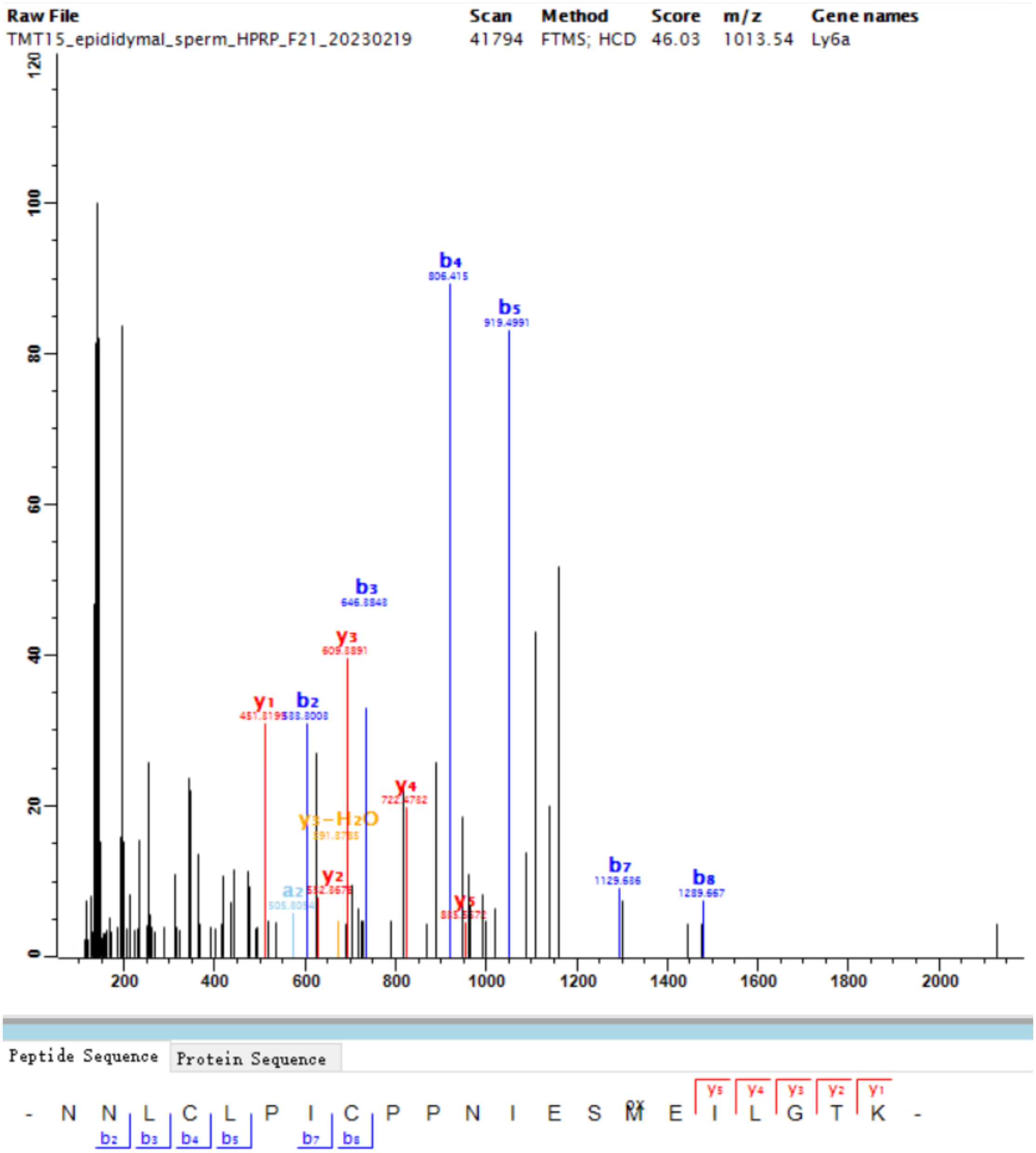
Raw File
TMT15_epididymal_sperm_HPRP_F21_20230219
Scan
41794
Method
FTMS; HCD
Score
46.03
m/z
1013.54
Gene names
Ly6a
b4
806.415
b5
919.4991
b3
646.8848
y3
609.3891
y1
b2
588.8008
y4
b7
1129.686
b8
1289.667
a2
y2
y5
Peptide Sequence
Protein Sequence
- N N L C L P I C P P N I E S M E I L G T K -

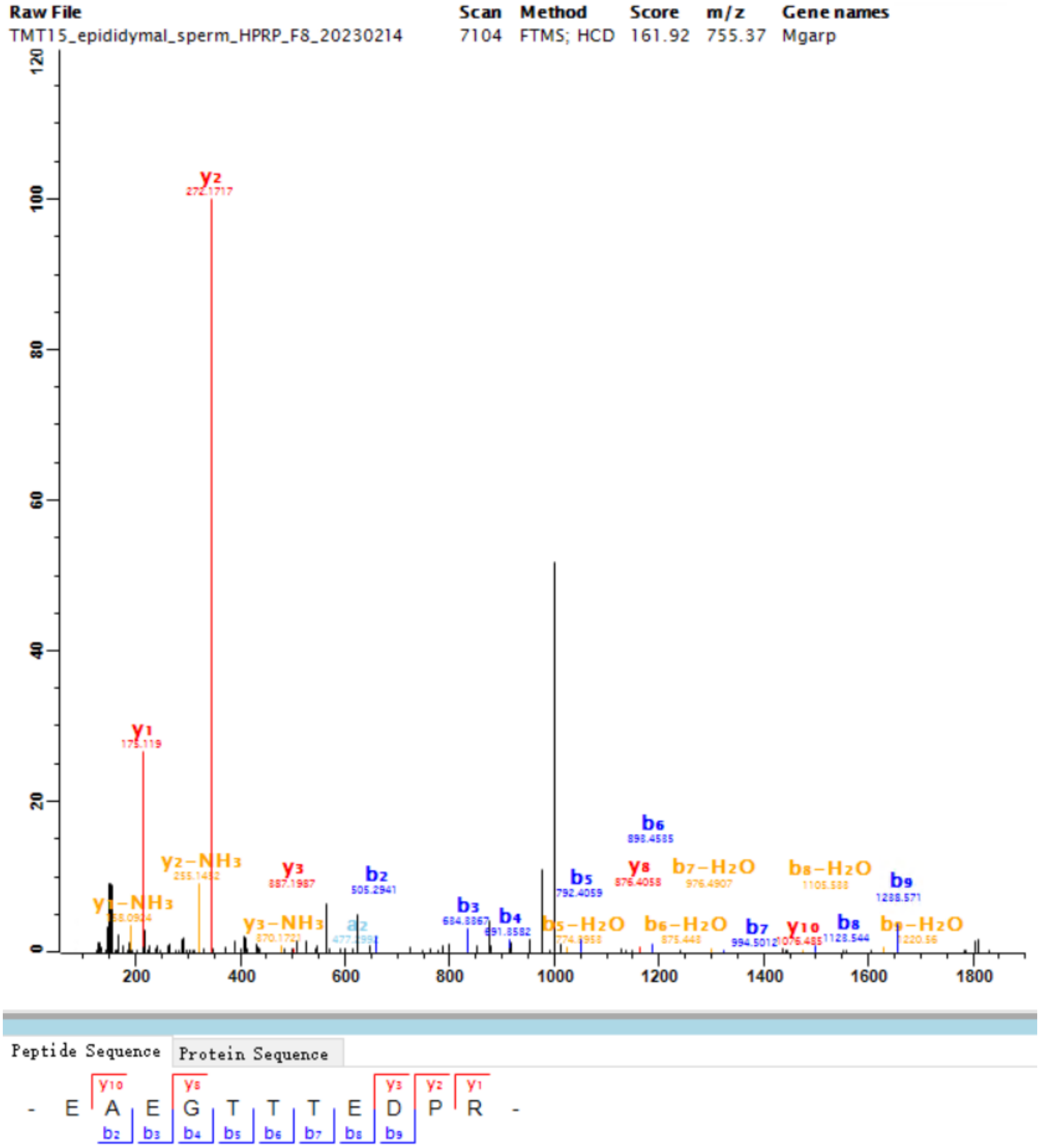
Raw File
TMT15_epididymal_sperm_HPRP_F8_20230214
Scan
7104
Method
FTMS; HCD
Score
161.92
m/z
755.37
Gene names
Mgarp
Peptide Sequence
Protein Sequence
- E A E G T T T E D P R -

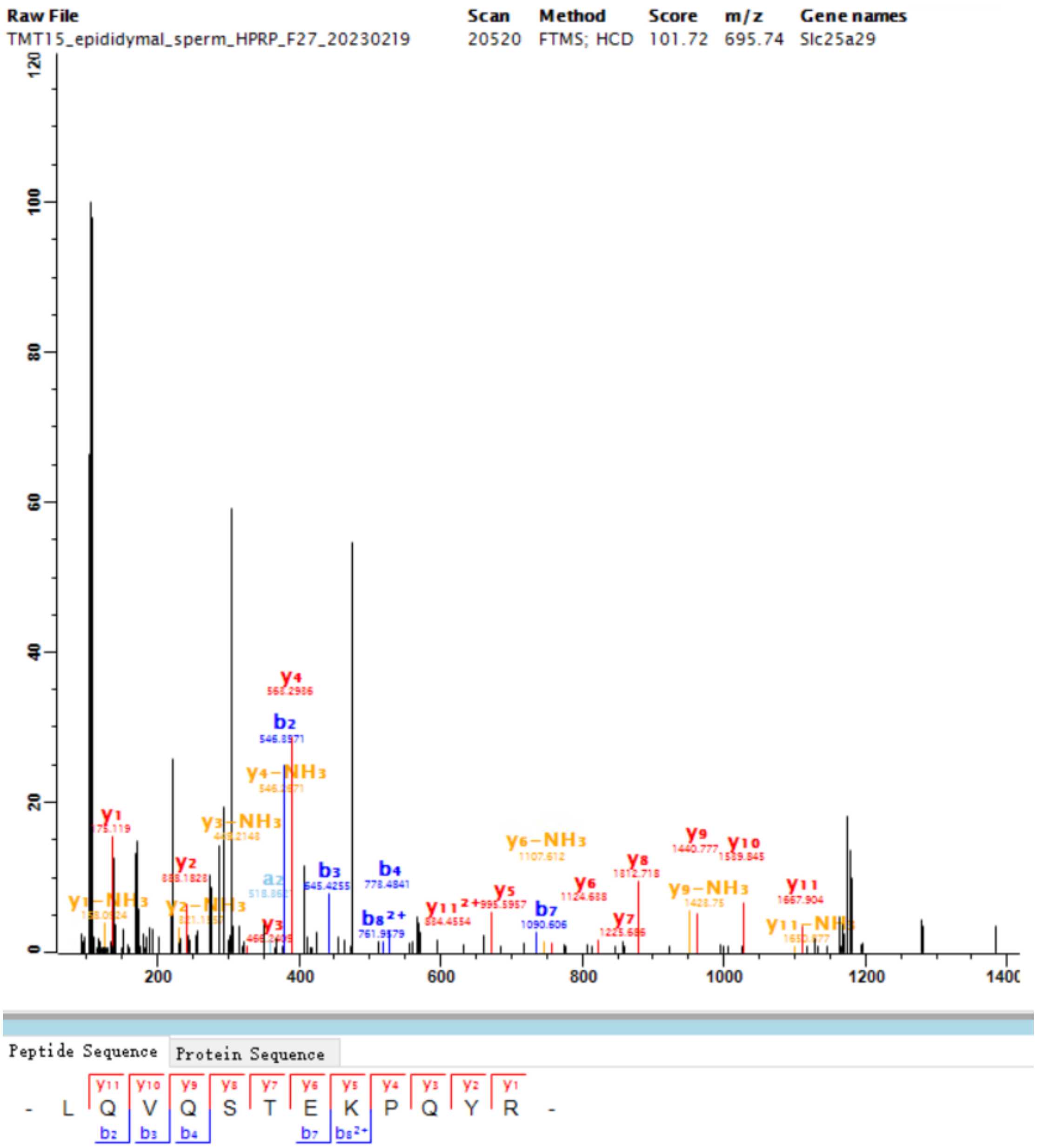

Raw File
TMT15_epididymal_sperm_HPRP_F27_20230219
Scan 20520
Method FTMS; HCD
Score 101.72
m/z 695.74
Gene names Slc25a29
y4
b2
y4−NH3
y1
y3−NH3
y2
a2
b3
b4
y6−NH3
y8
y9
y10
y6
y5
b7
y9−NH3
y11
y1−NH3
y2−NH3
y3
b8 2+
y11 2+
y7
y11−NH3
Peptide Sequence
Protein Sequence
- L Q V Q S T E K P Q Y R -

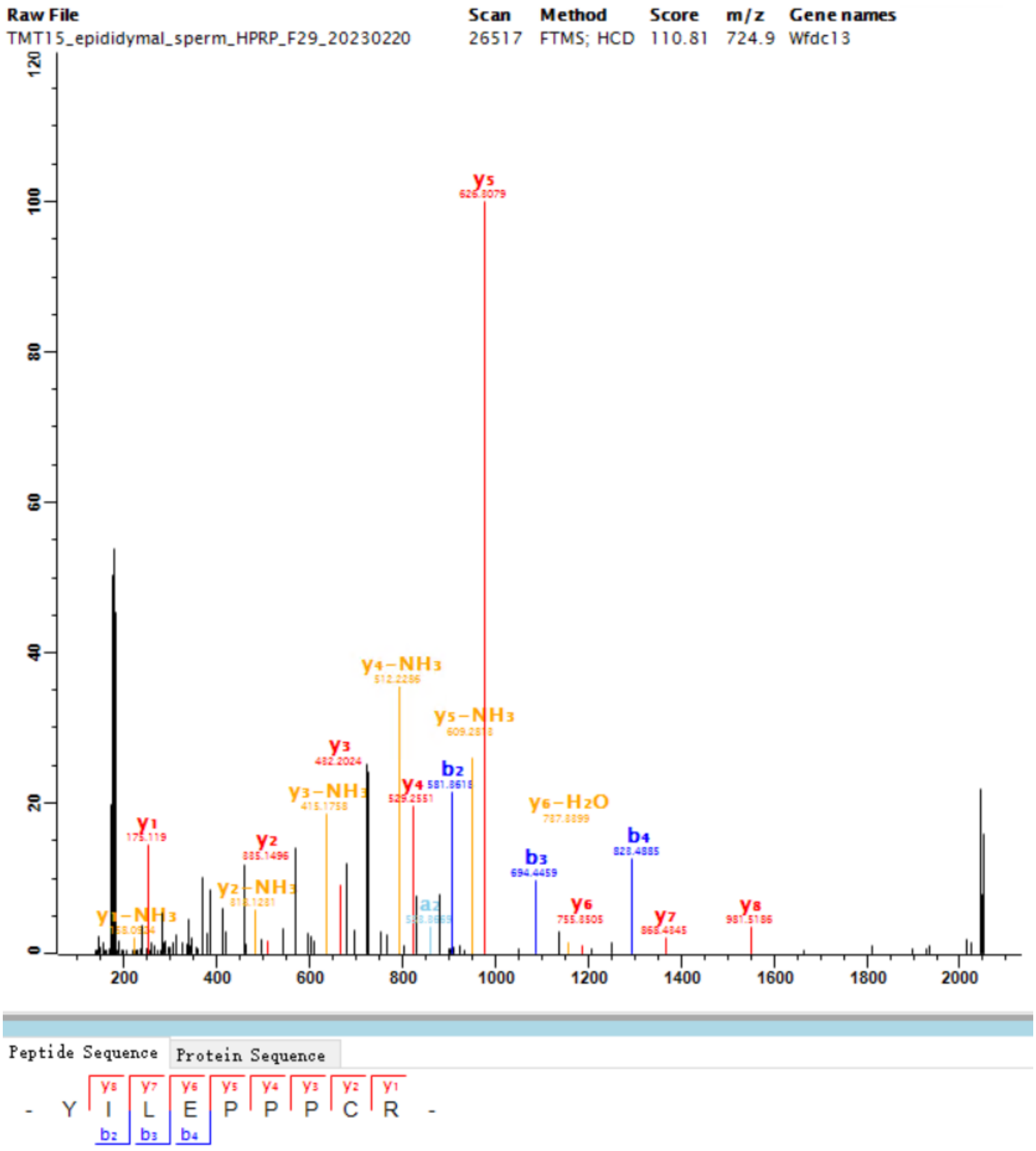

Raw File
TMT15_epididymal_sperm_HPRP_F29_20230220
Scan 26517
Method FTMS; HCD
Score 110.81
m/z 724.9
Gene names Wfdc13
Peptide Sequence
Protein Sequence
- Y I L E P P P C R -

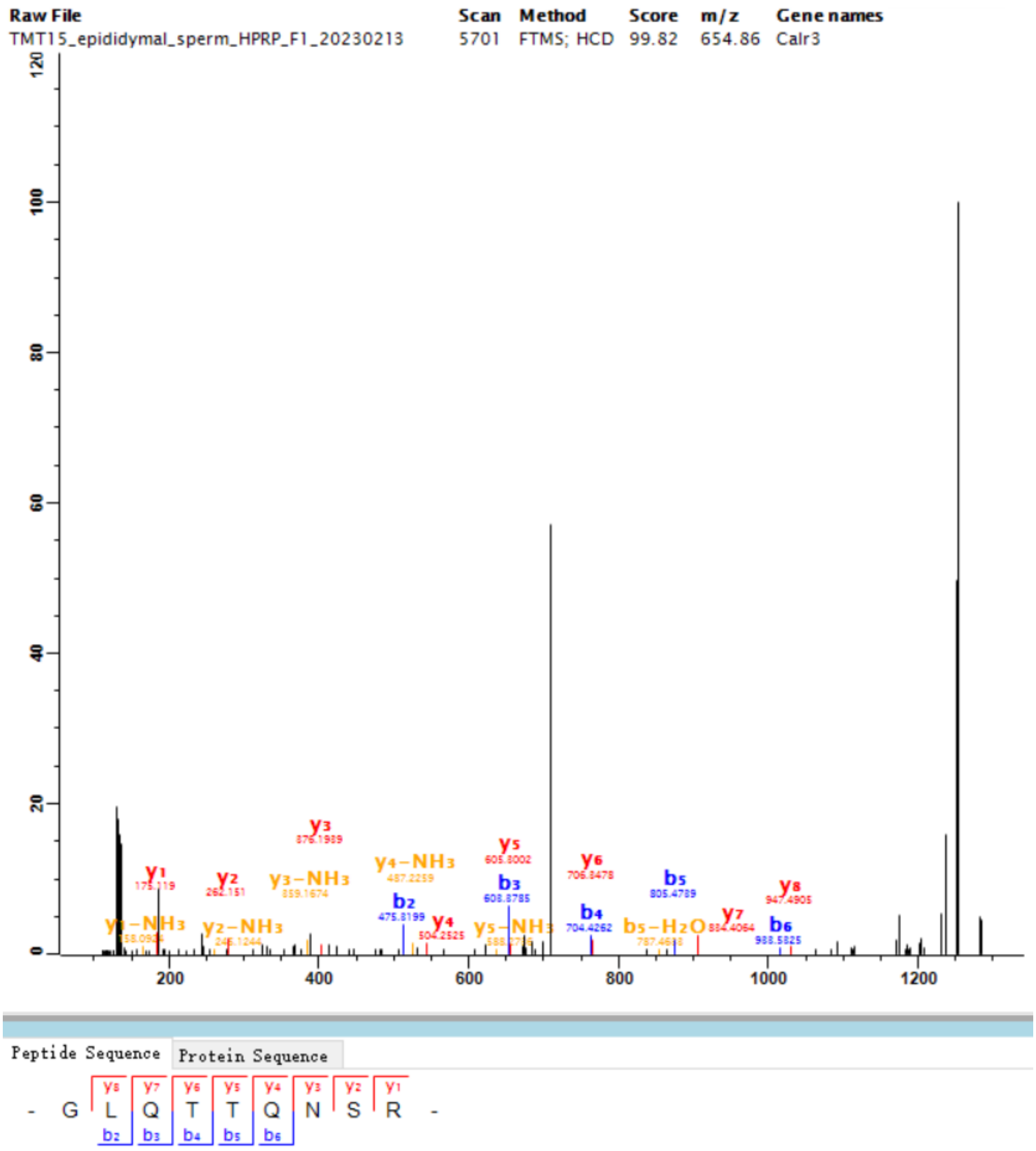

Raw File
TMT15_epididymal_sperm_HPRP_F1_20230213
Scan
5701
Method
FTMS; HCD
Score
99.82
m/z
654.86
Gene names
Calr3
y1
y2
y3
y4
y5
y6
y7
y8
b2
b3
b4
b5
b6
y2-NH3
y3-NH3
y4-NH3
y5-NH3
b5-H2O
175.119
262.151
487.2259
605.8002
706.8478
805.4789
947.4905
475.8199
704.4262
504.2525
988.5825
Peptide Sequence
Protein Sequence
- G L Q T T Q N S R -

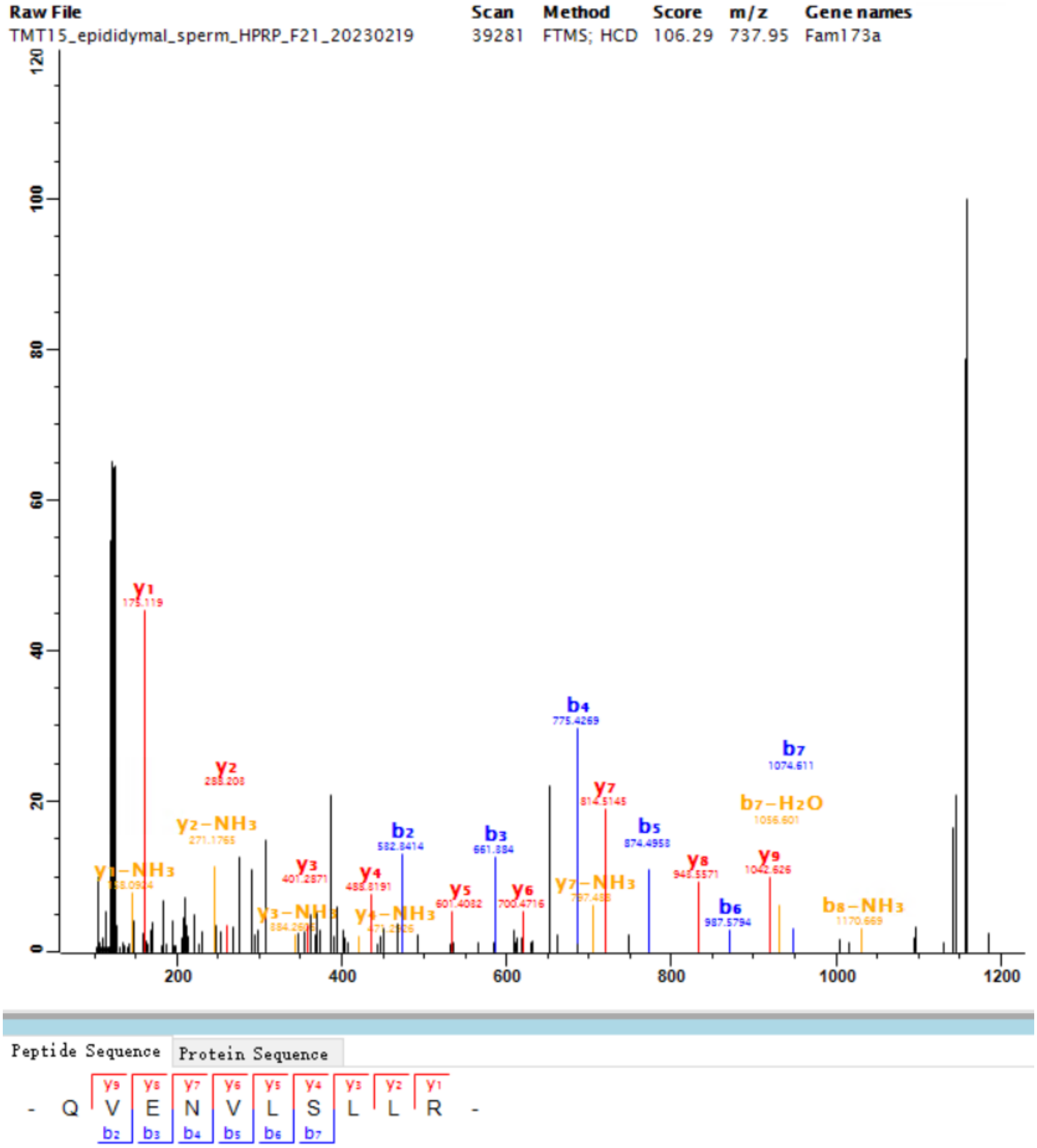
Raw File
TMT15_epididymal_sperm_HPRP_F21_20230219
Scan
39281
Method
FTMS; HCD
Score
106.29
m/z
737.95
Gene names
Fam173a
Peptide Sequence
Protein Sequence
- Q V E N V L S L L R -

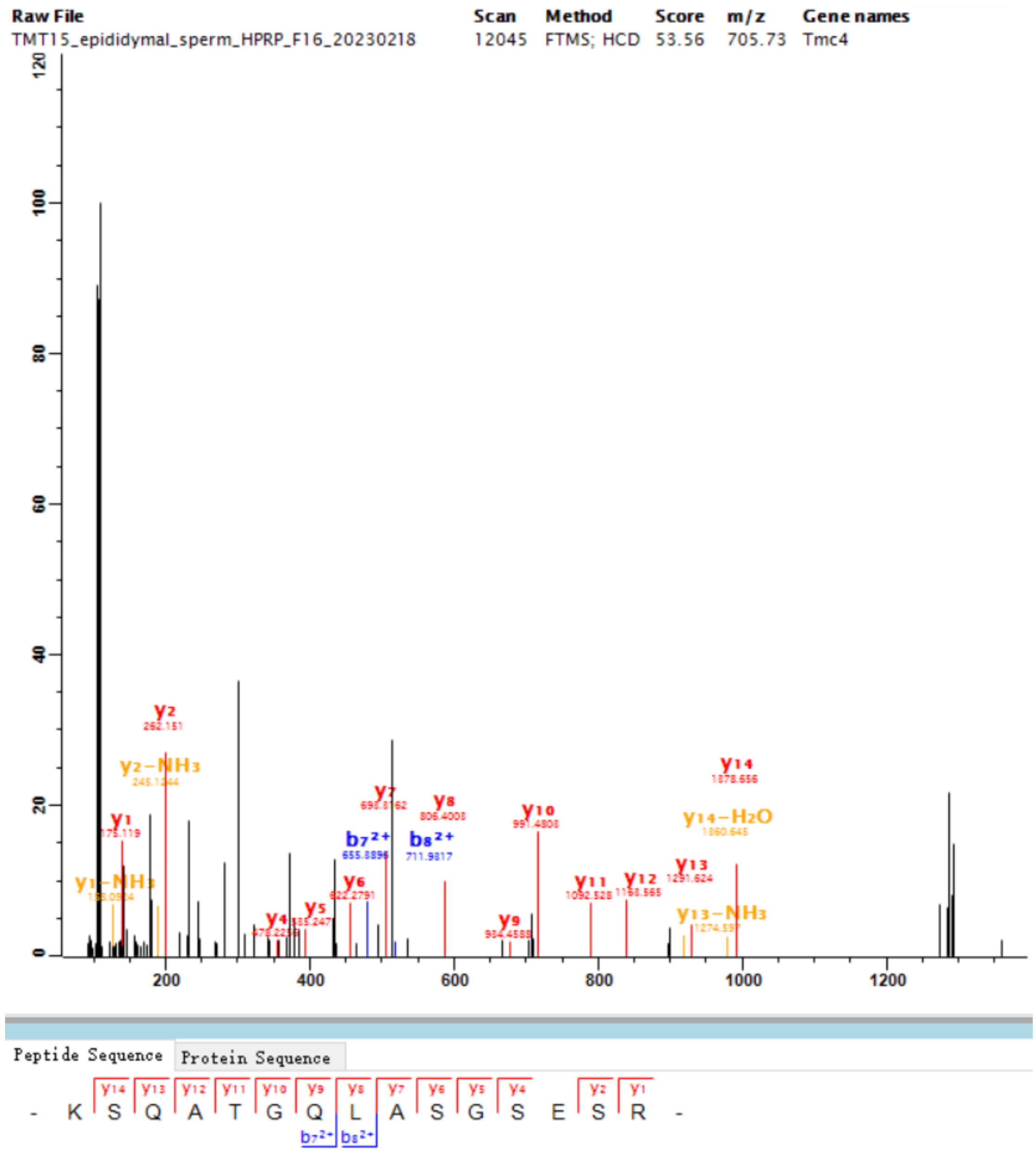

Raw File
TMT15_epididymal_sperm_HPRP_F16_20230218
Scan
12045
Method
FTMS; HCD
Score
53.56
m/z
705.73
Gene names
Tmc4
y2
262.151
y1
175.119
y6
622.2791
b7 2+
655.8896
y8
806.4008
b8 2+
711.9817
y10
991.4808
y11
1092.528
y13
1291.624
y14
1878.656
y14-H2O
1860.645
y13-NH3
0
20
40
60
80
100
120
200
400
600
800
1000
1200
Peptide Sequence
Protein Sequence
- K S Q A T G Q L A S G S E S R -

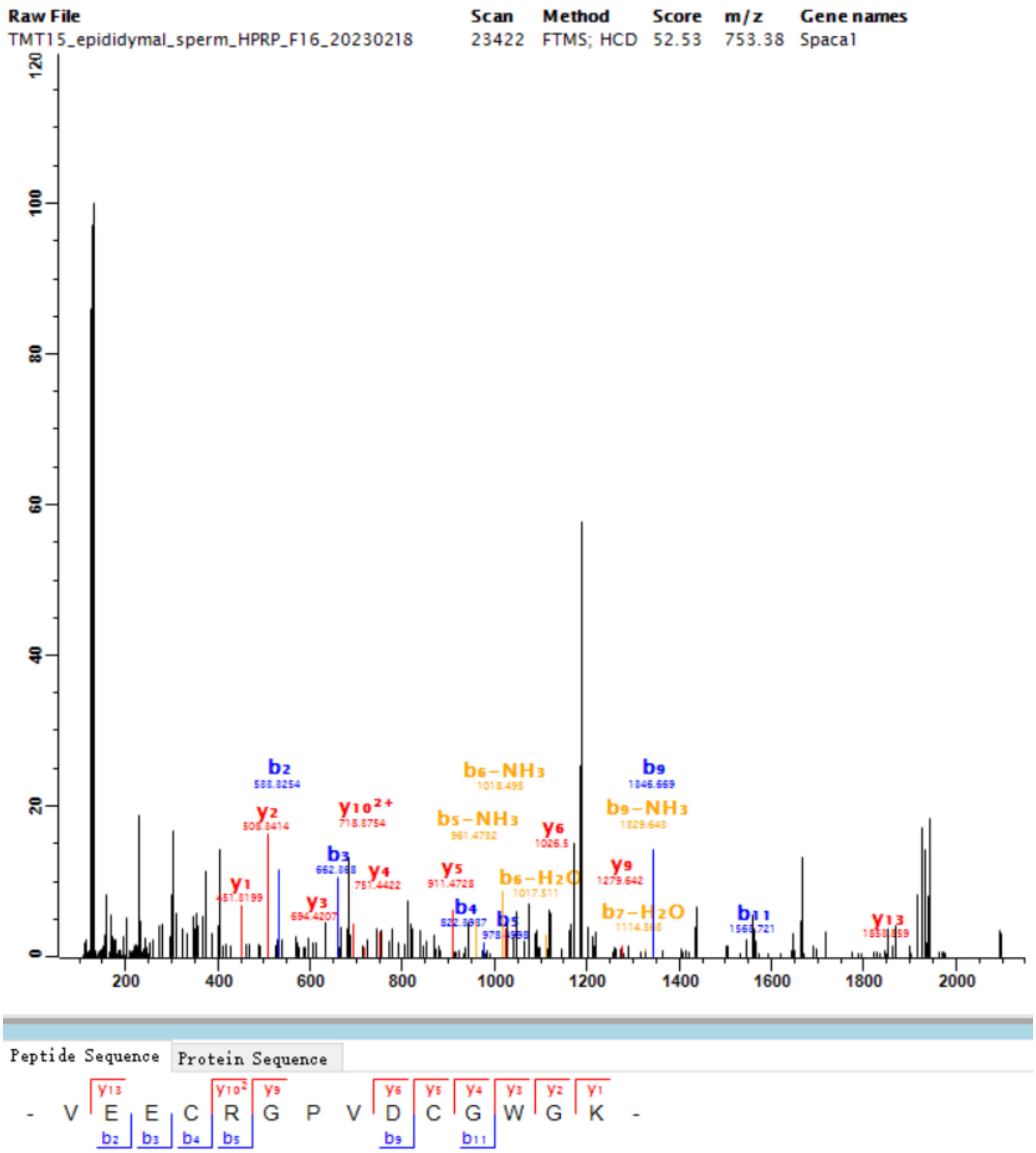

Raw File
TMT15_epididymal_sperm_HPRP_F16_20230218
Scan
23422
Method
FTMS; HCD
Score
52.53
m/z
753.38
Gene names
Spaca1
b2
y2
y10 2+
b3
y4
y1
y3
b6-NH3
b5-NH3
y6
y5
b6-H2O
b4
b5
b9
b9-NH3
y9
b7-H2O
b11
y13
Peptide Sequence
Protein Sequence
- V E E C R G P V D C G W G K -

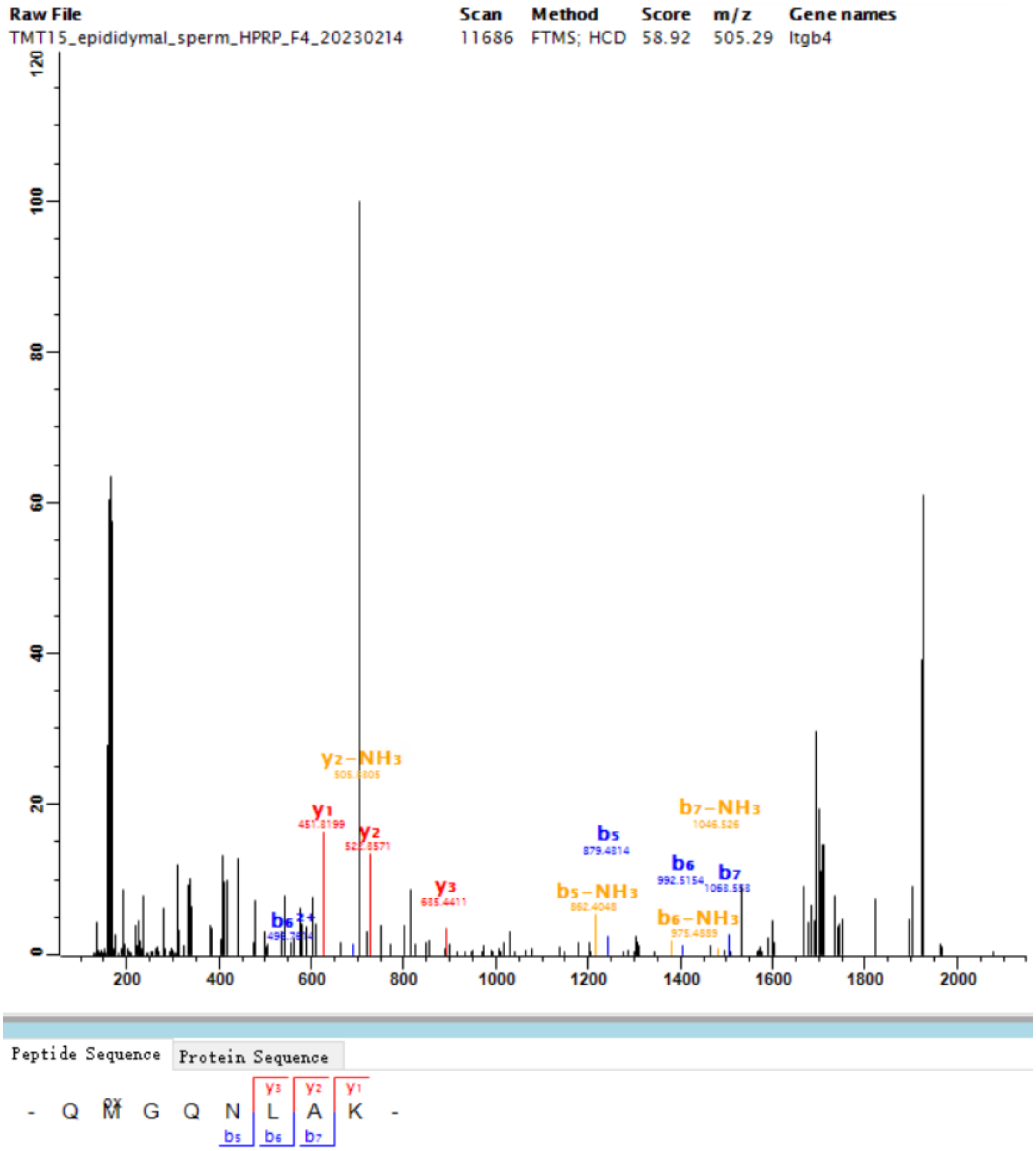

Raw File
TMT15_epididymal_sperm_HPRP_F4_20230214
Scan
11686
Method
FTMS; HCD
Score
58.92
m/z
505.29
Gene names
Itgb4
y2-NH3
y1
451.3199
y2
y3
b6 2+
b5
879.4814
b6
992.5154
b7
b5-NH3
862.4048
b6-NH3
975.4889
b7-NH3
1046.526
Peptide Sequence
Protein Sequence
- Q M G Q N L A K -
ox
y3 y2 y1
b5 b6 b7

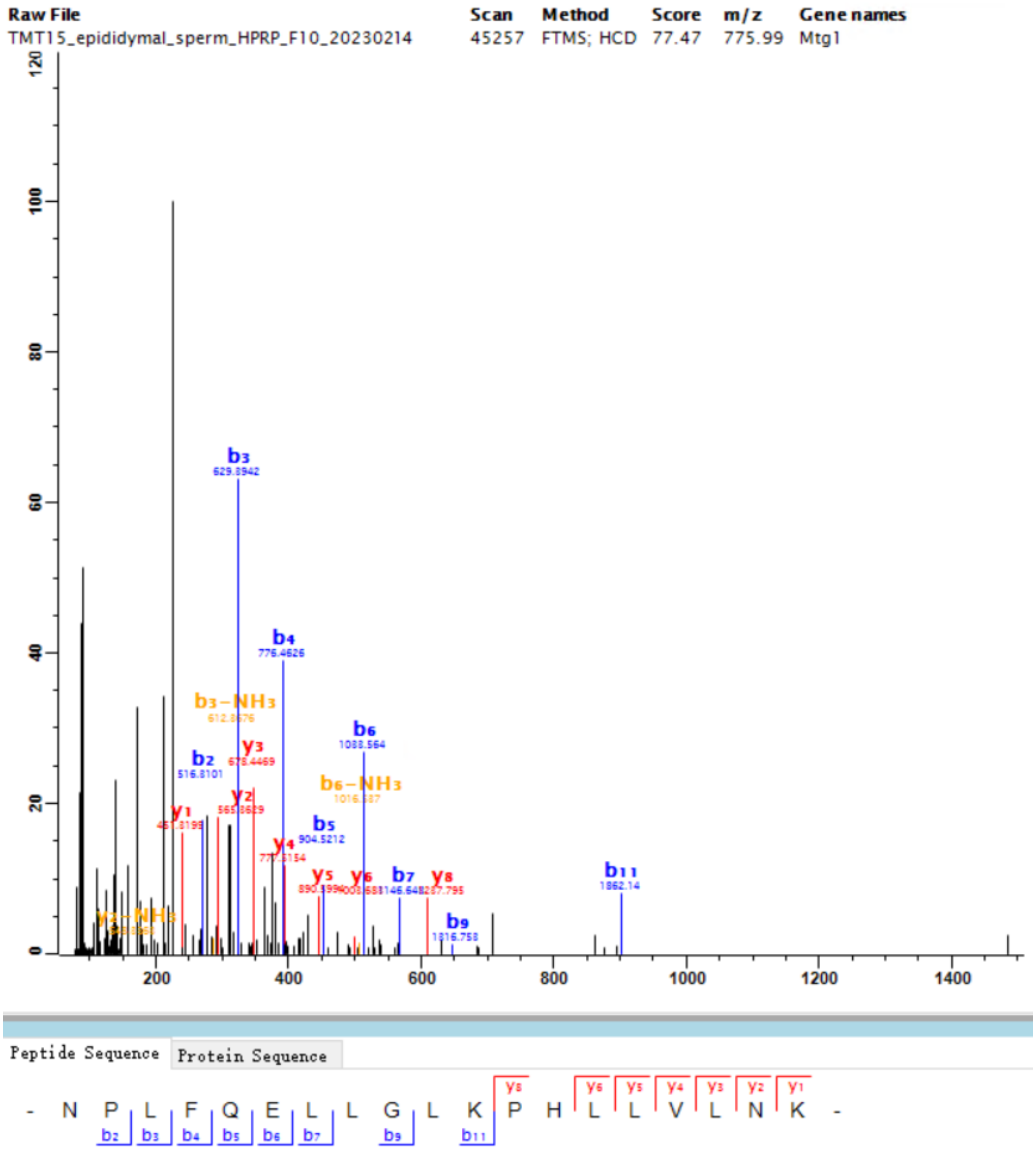
Raw File
TMT15_epididymal_sperm_HPRP_F10_20230214
Scan
45257
Method
FTMS; HCD
Score
77.47
m/z
775.99
Gene names
Mtg1
b3
629.8942
b4
776.4626
b6
1088.564
b2
516.8101
b5
904.5212
b7
b9
1316.758
b11
1862.14
y1
y2
y3
y4
y5
y6
y8
b3-NH3
b6-NH3
Peptide Sequence
Protein Sequence
- N P L F Q E L L G L K P H L L V L N K -

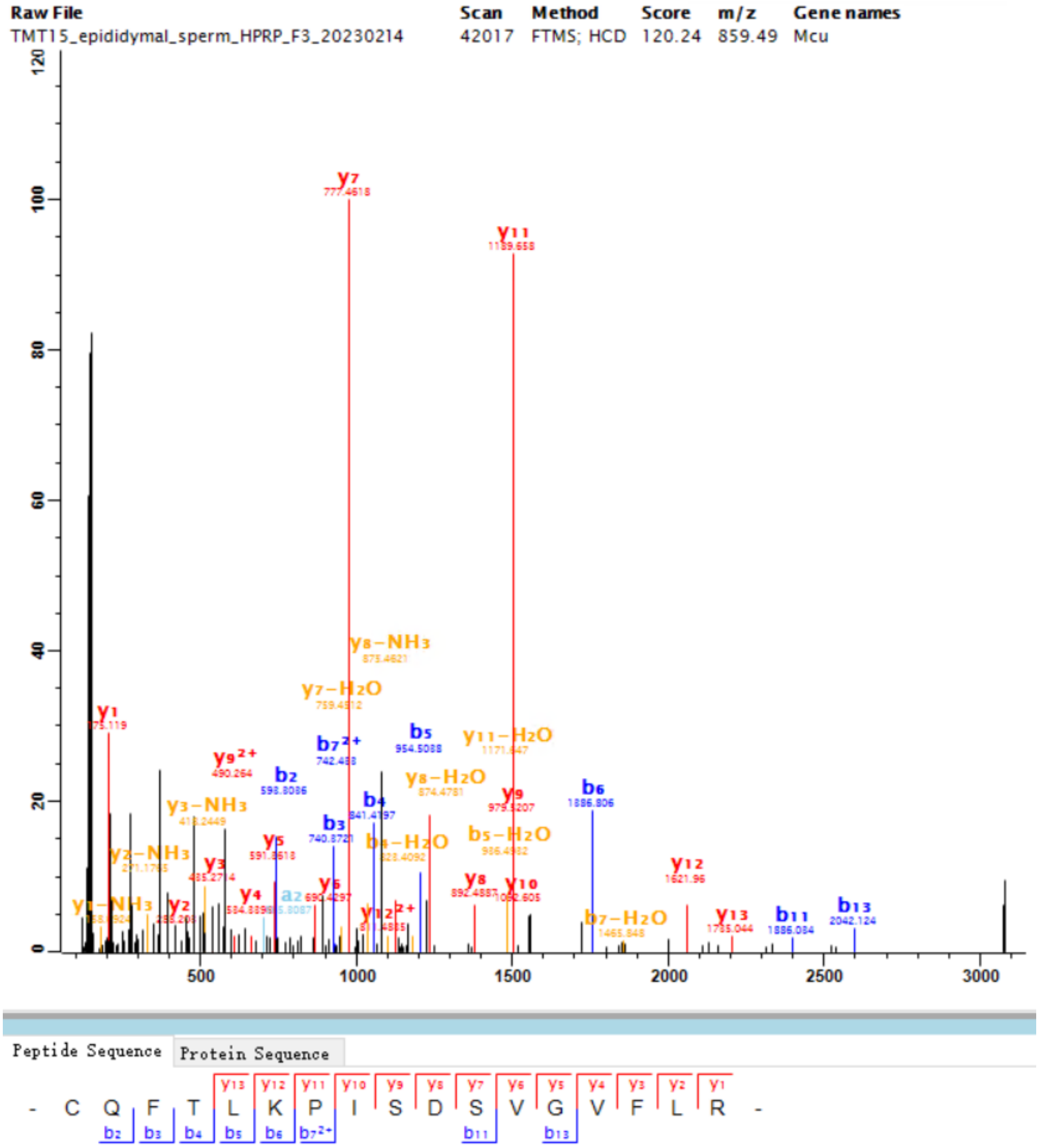
Raw File
TMT15_epididymal_sperm_HPRP_F3_20230214
Scan
42017
Method
FTMS; HCD
Score
120.24
m/z
859.49
Gene names
Mcu
y7
777.4618
y11
1139.658
y8-NH3
875.4621
y7-H2O
759.4512
y1
175.119
b7 2+
742.488
b5
954.5088
y11-H2O
1171.647
y9 2+
490.264
b2
598.8086
y8-H2O
874.4781
b6
1886.806
y9
979.5207
y3-NH3
413.2449
b4
841.4797
b3
740.8721
b5-H2O
936.4982
y2-NH3
271.1765
y3
485.2714
y5
591.3618
y6
690.4297
y8
892.4887
y10
1052.605
y12
1621.96
y4
584.8896
a2
y1-NH3
y2
y12 2+
b7-H2O
1465.848
y13
1735.044
b11
1896.084
b13
2042.124
500
1000
1500
2000
2500
3000
0
20
40
60
80
100
120
Peptide Sequence
Protein Sequence
- C Q F T L K P I S D S V G V F L R -

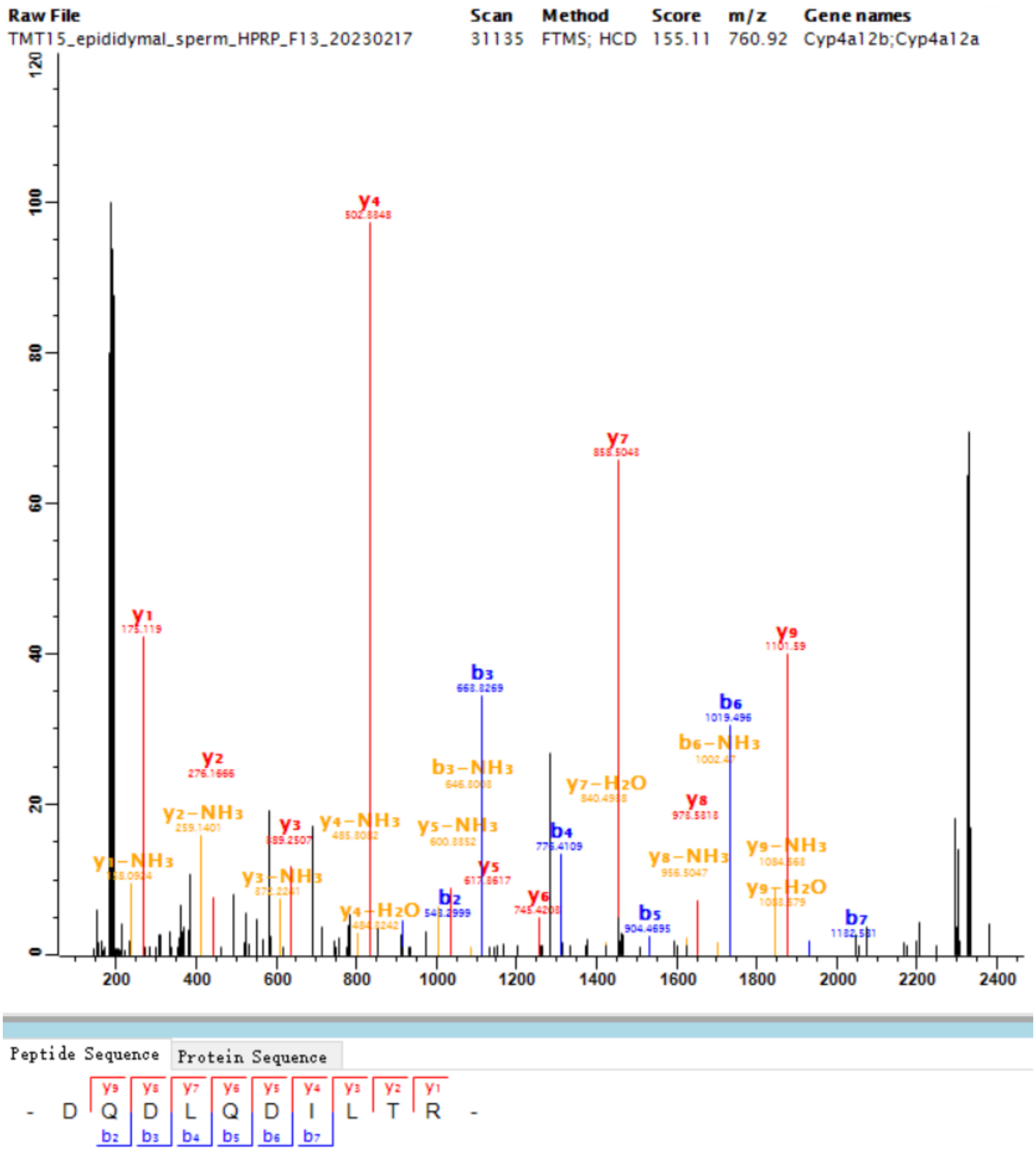
Raw File
TMT15_epididymal_sperm_HPRP_F13_20230217
Scan
31135
Method
FTMS; HCD
Score
155.11
m/z
760.92
Gene names
Cyp4a12b;Cyp4a12a
0
20
40
60
80
100
120
200
400
600
800
1000
1200
1400
1600
1800
2000
2200
2400
y1
175.119
y2
276.1666
y2-NH3
259.1401
y3
389.2507
y4
502.3348
y4-NH3
y4-H2O
y5-NH3
y5
y6
y7
858.5048
y7-H2O
y8
y8-NH3
y9
1101.59
y9-NH3
y9-H2O
b2
543.2999
b3
668.3269
b3-NH3
b4
776.4109
b5
904.4695
b6
1019.496
b6-NH3
1002.47
b7
Peptide Sequence
Protein Sequence
- D Q D L Q D I L T R -
y9 y8 y7 y6 y5 y4 y3 y2 y1
b2 b3 b4 b5 b6 b7

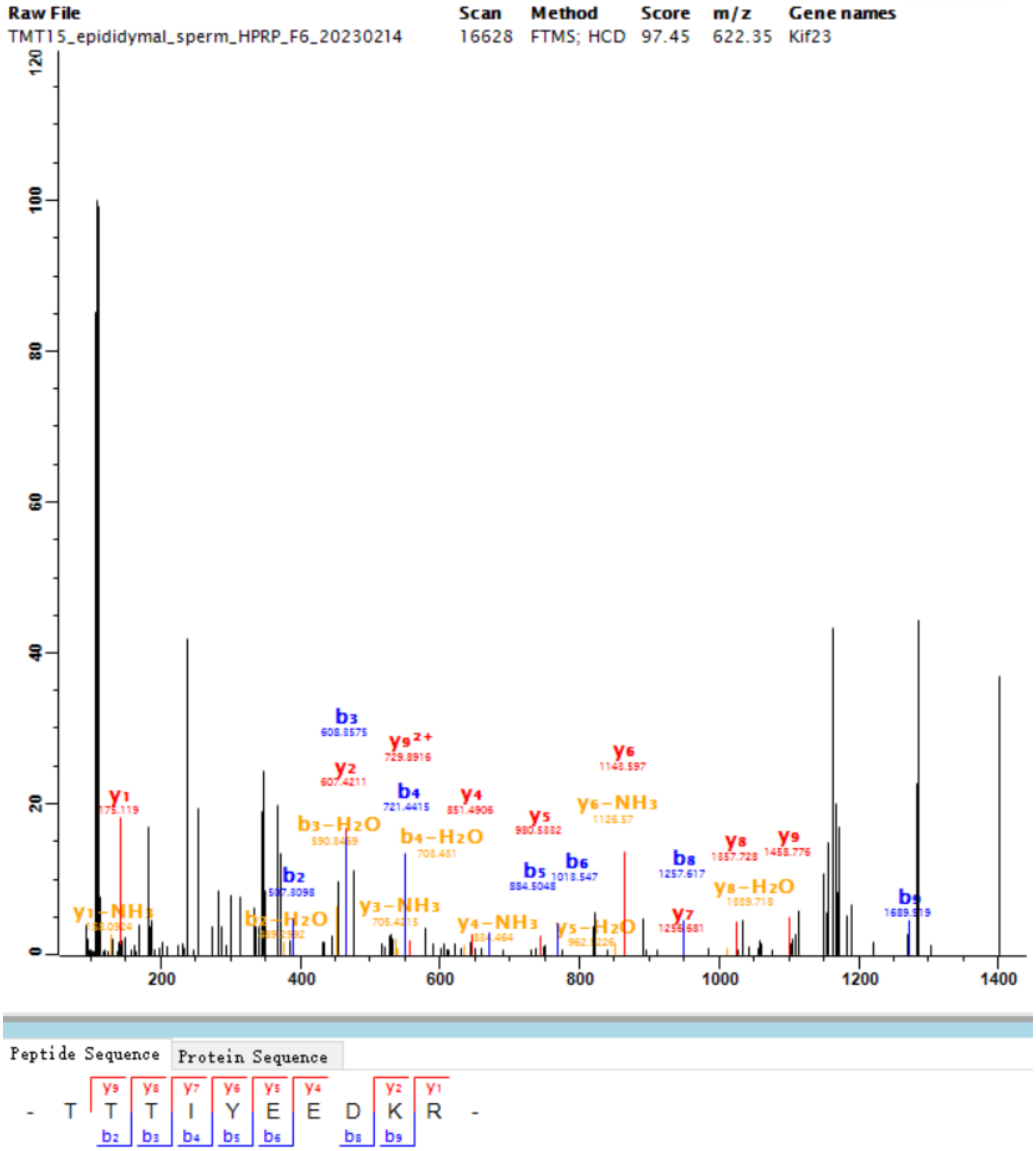
Raw File
TMT15_epididymal_sperm_HPRP_F6_20230214
Scan
16628
Method
FTMS; HCD
Score
97.45
m/z
622.35
Gene names
Kif23
Peptide Sequence
Protein Sequence
- T T T I Y E E D K R -

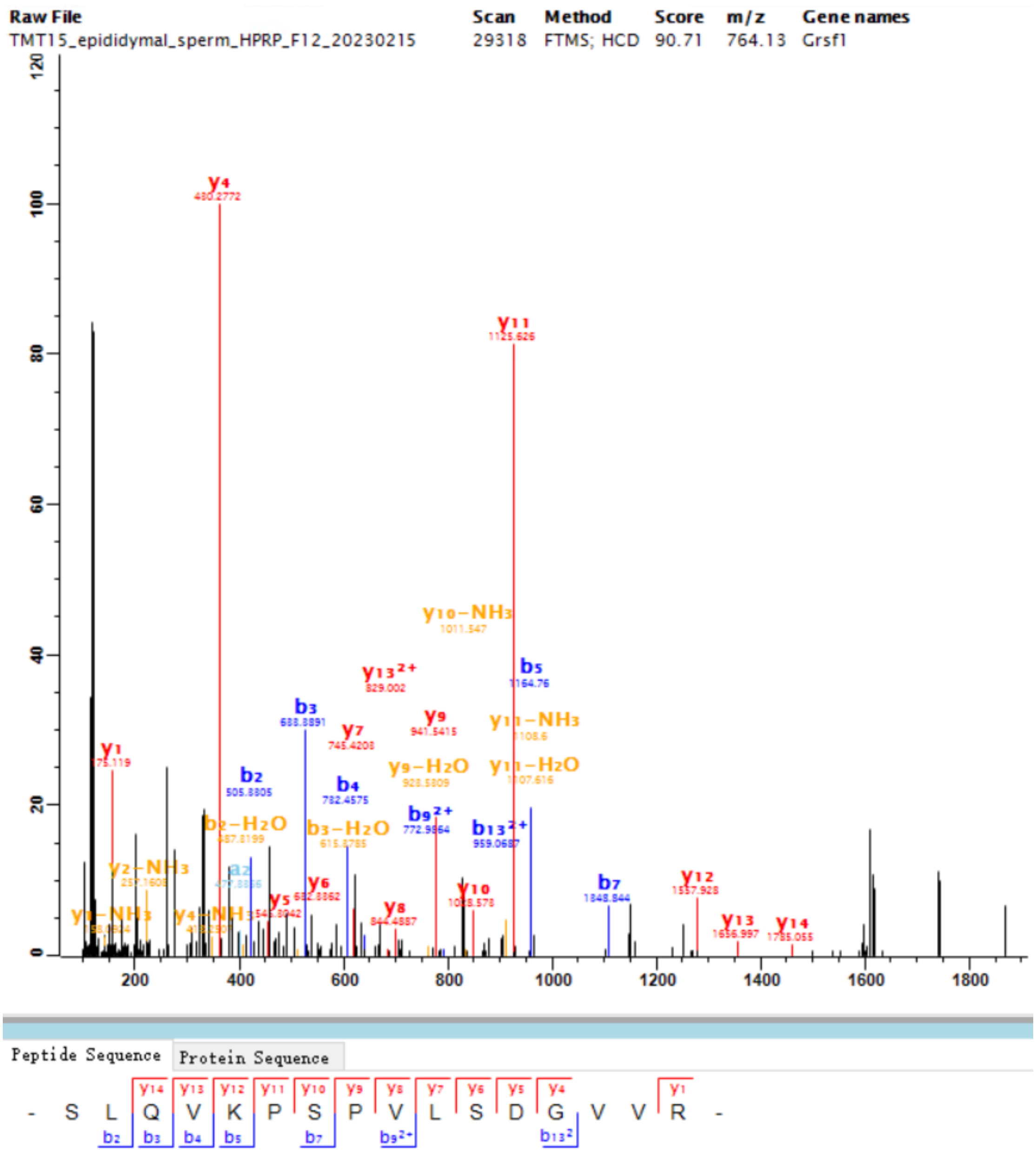

Raw File
TMT15_epididymal_sperm_HPRP_F12_20230215
Scan 29318
Method FTMS; HCD
Score 90.71
m/z 764.13
Gene names Grsf1
Peptide Sequence
Protein Sequence
- S L Q V K P S P V L S D G V V R -

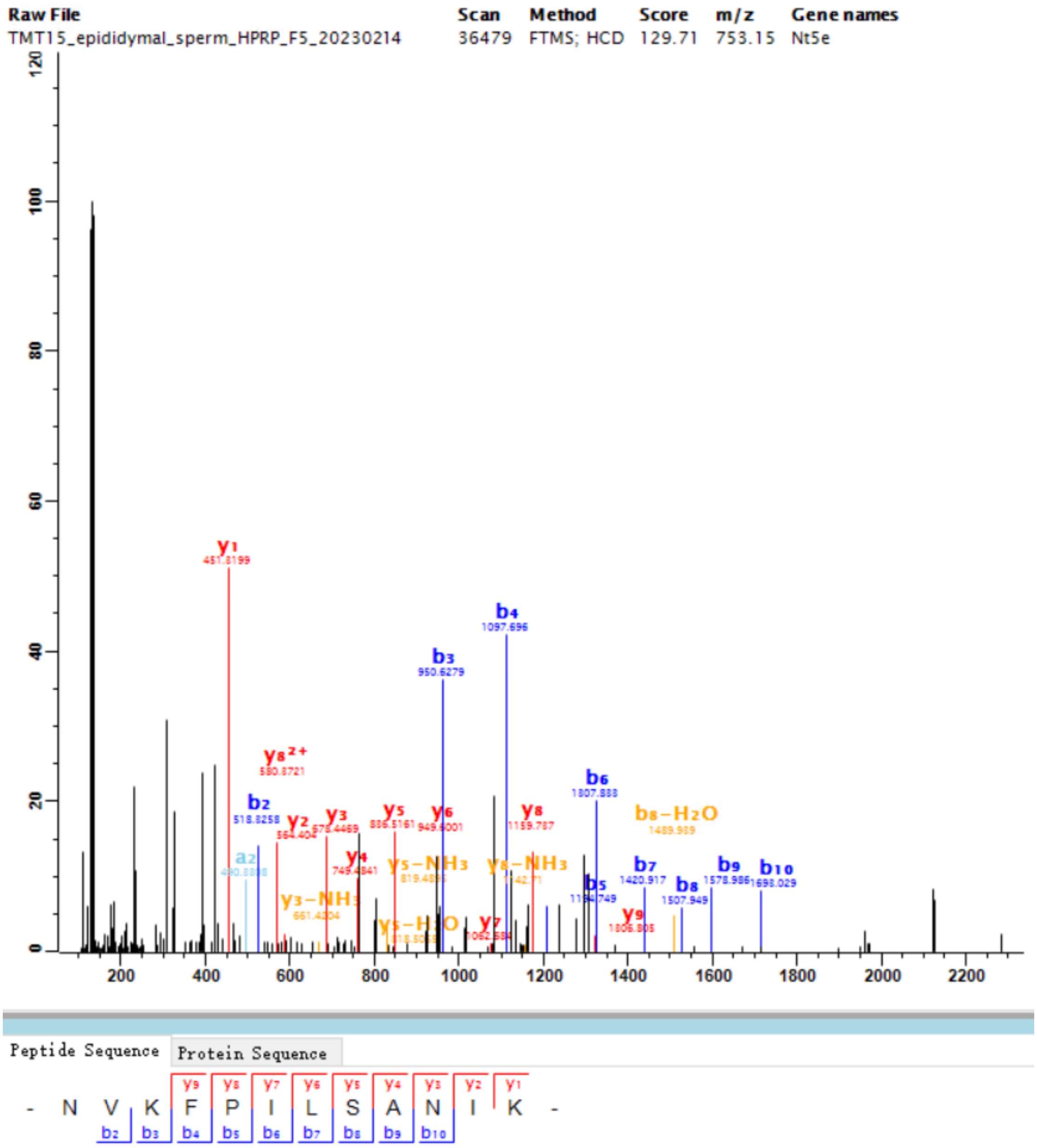
Raw File
TMT15_epididymal_sperm_HPRP_F5_20230214
Scan
36479
Method
FTMS; HCD
Score
129.71
m/z
753.15
Gene names
Nt5e
Peptide Sequence
Protein Sequence
- N V K F P I L S A N I K -

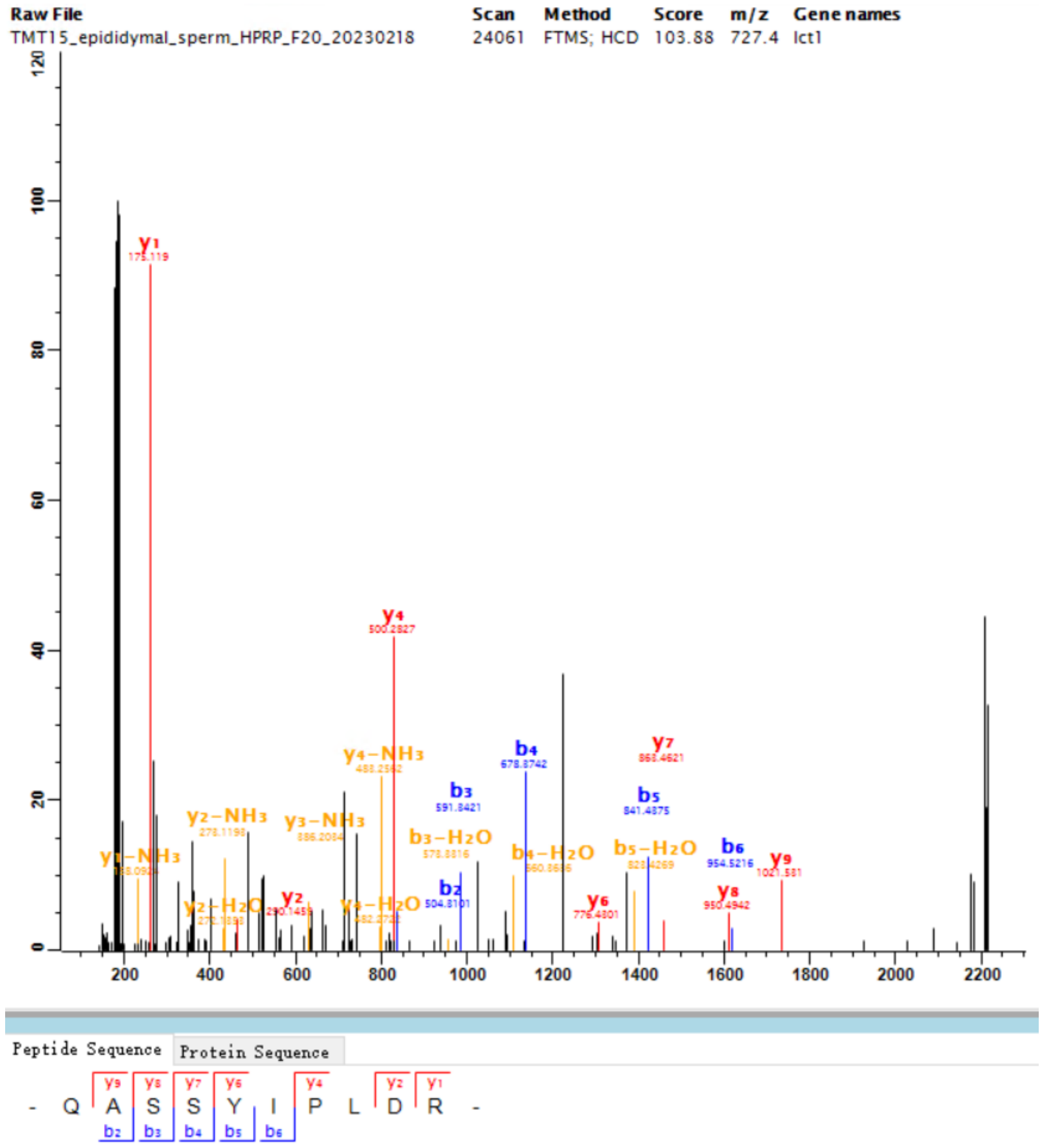

Raw File
TMT15_epididymal_sperm_HPRP_F20_20230218
Scan
24061
Method
FTMS; HCD
Score
103.88
m/z
727.4
Gene names
lct1
y1
175.119
y4
500.2827
y4-NH3
y2-NH3
y3-NH3
y1-NH3
y2-H2O
y2
y3-H2O
b2
b3
591.8421
b3-H2O
b4
678.8742
b4-H2O
y6
y7
b5
841.4875
b5-H2O
b6
954.5216
y8
y9
1021.581
Peptide Sequence
Protein Sequence
- Q A S S Y I P L D R -

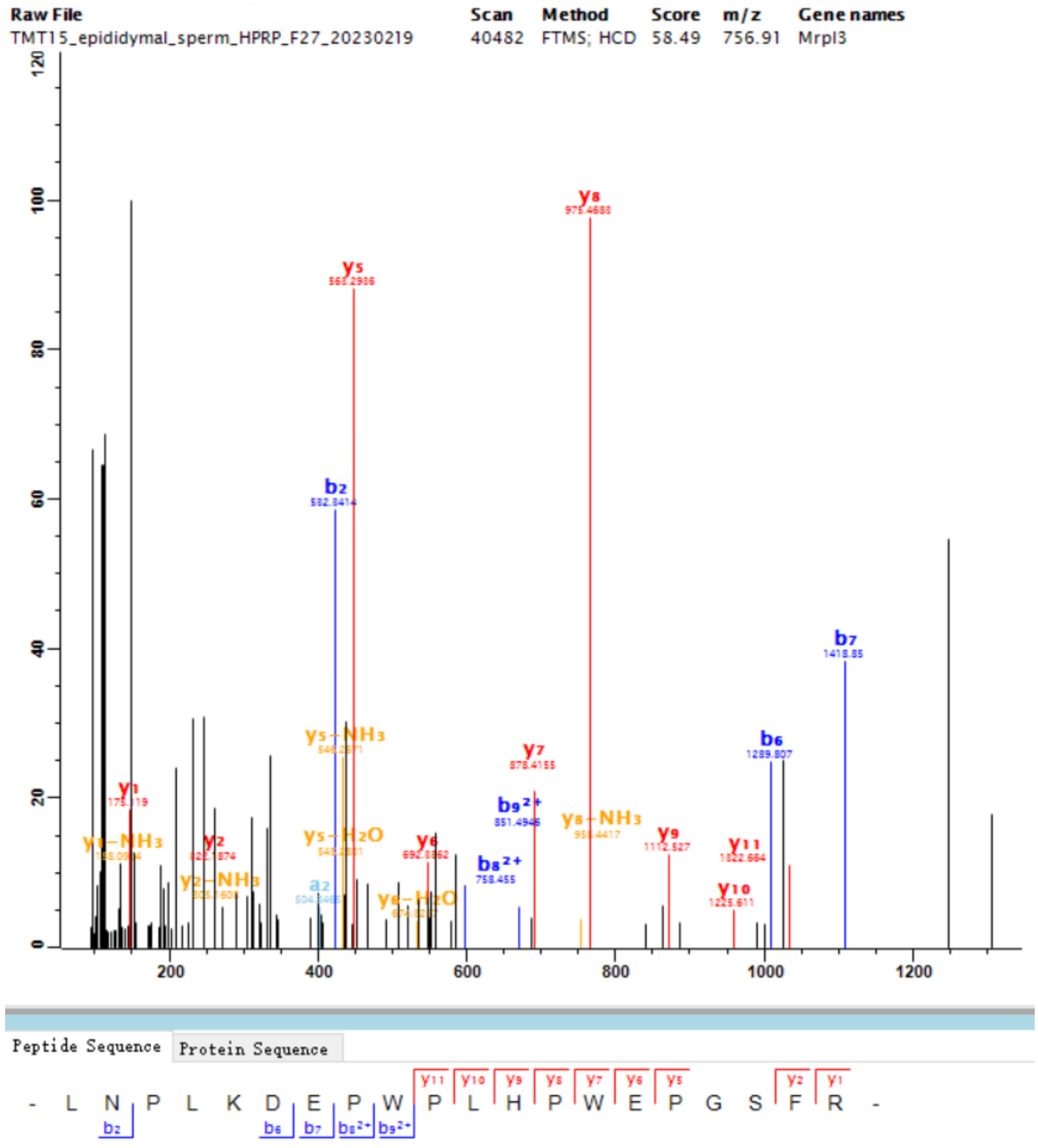

Raw File
TMT15_epididymal_sperm_HPRP_F27_20230219
Scan
40482
Method
FTMS; HCD
Score
58.49
m/z
756.91
Gene names
Mrpl3
y8
975.4688
y5
b2
582.8414
b7
1418.85
b6
1289.807
y7
878.4155
y5-NH3
b9 2+
851.4946
y8-NH3
y1
y1-NH3
y2
y5-H2O
y6
692.8862
b8 2+
758.455
y9
1112.527
y11
y10
1225.611
y2-NH3
a2
y6-H2O
0
20
40
60
80
100
120
200
400
600
800
1000
1200
Peptide Sequence
Protein Sequence
- L N P L K D E P W P L H P W E P G S F R -

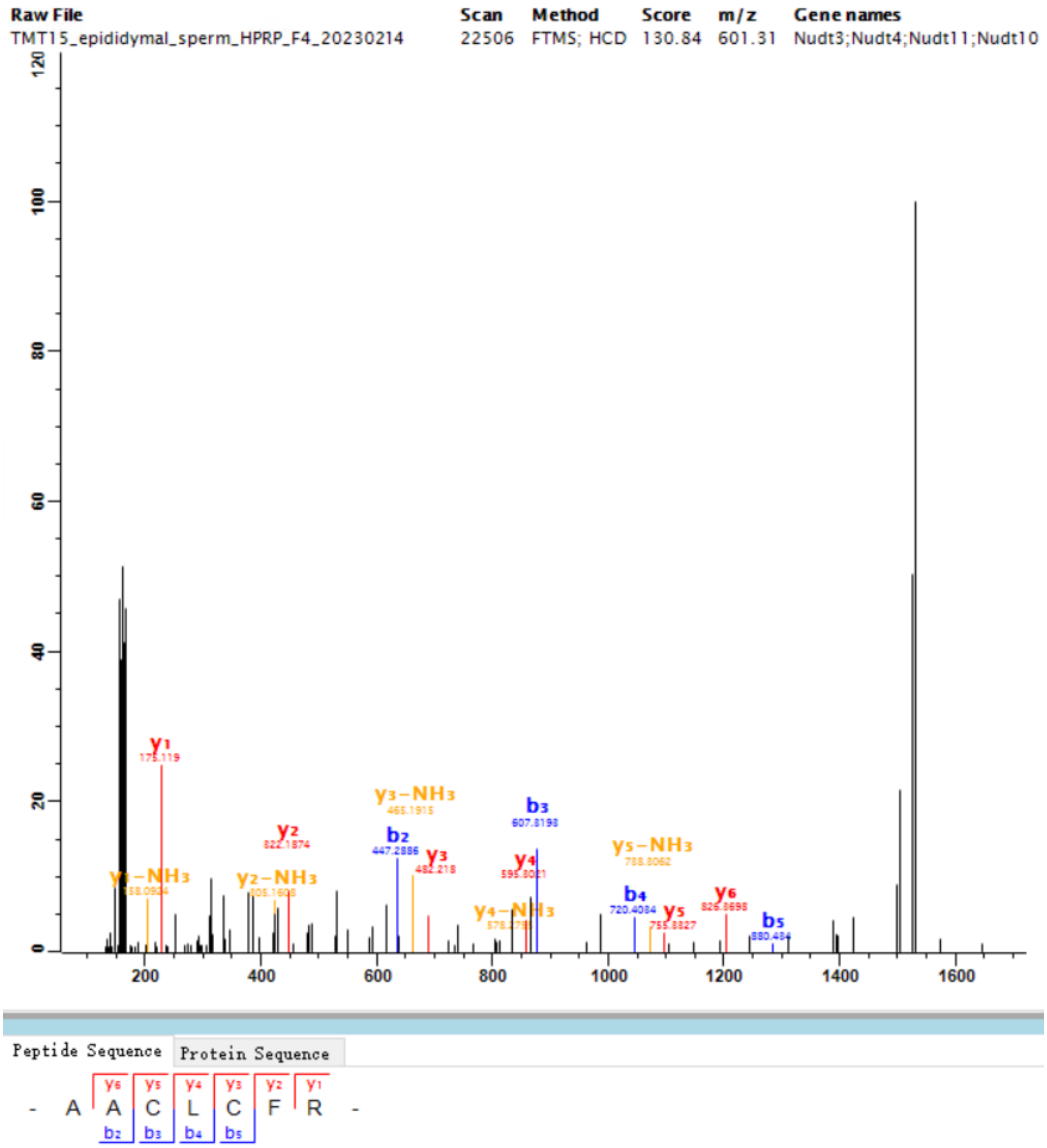
Raw File
TMT15_epididymal_sperm_HPRP_F4_20230214
Scan
22506
Method
FTMS; HCD
Score
130.84
m/z
601.31
Gene names
Nudt3;Nudt4;Nudt11;Nudt10
0
20
40
60
80
100
120
200
400
600
800
1000
1200
1400
1600
y1
175.119
y1-NH3
y2
322.1874
y2-NH3
y3-NH3
465.1915
b2
447.2886
y3
482.218
b3
607.8198
y4
595.8021
y4-NH3
y5-NH3
b4
720.4084
y5
755.8827
y6
826.8698
b5
880.484
Peptide Sequence
Protein Sequence
- A A C L C F R -
y6 y5 y4 y3 y2 y1
b2 b3 b4 b5

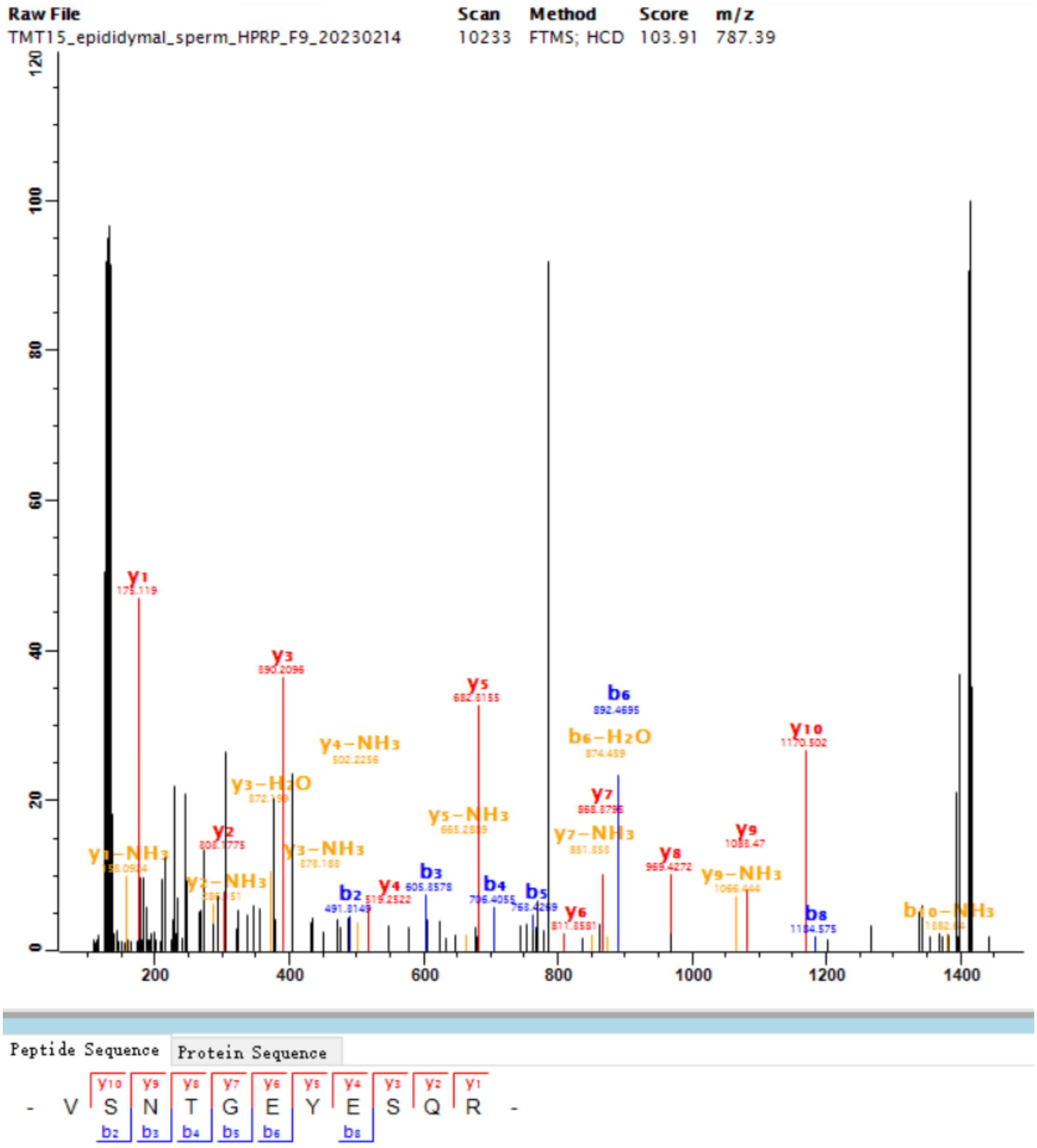
Raw File
TMT15_epididymal_sperm_HPRP_F9_20230214
Scan
10233
Method
FTMS; HCD
Score
103.91
m/z
787.39
0
20
40
60
80
100
120
200
400
600
800
1000
1200
1400
y1
175.119
y3
b6
892.4695
b6-H2O
874.459
y10
1170.502
y5
y4-NH3
y3-H2O
y5-NH3
y7
y7-NH3
y9
y9-NH3
y8
y2
y3-NH3
y1-NH3
y2-NH3
b2
b3
b4
b5
y4
y6
b8
b10-NH3
Peptide Sequence
Protein Sequence
- V S N T G E Y E S Q R -
y10 y9 y8 y7 y6 y5 y4 y3 y2 y1
b2 b3 b4 b5 b6 b8

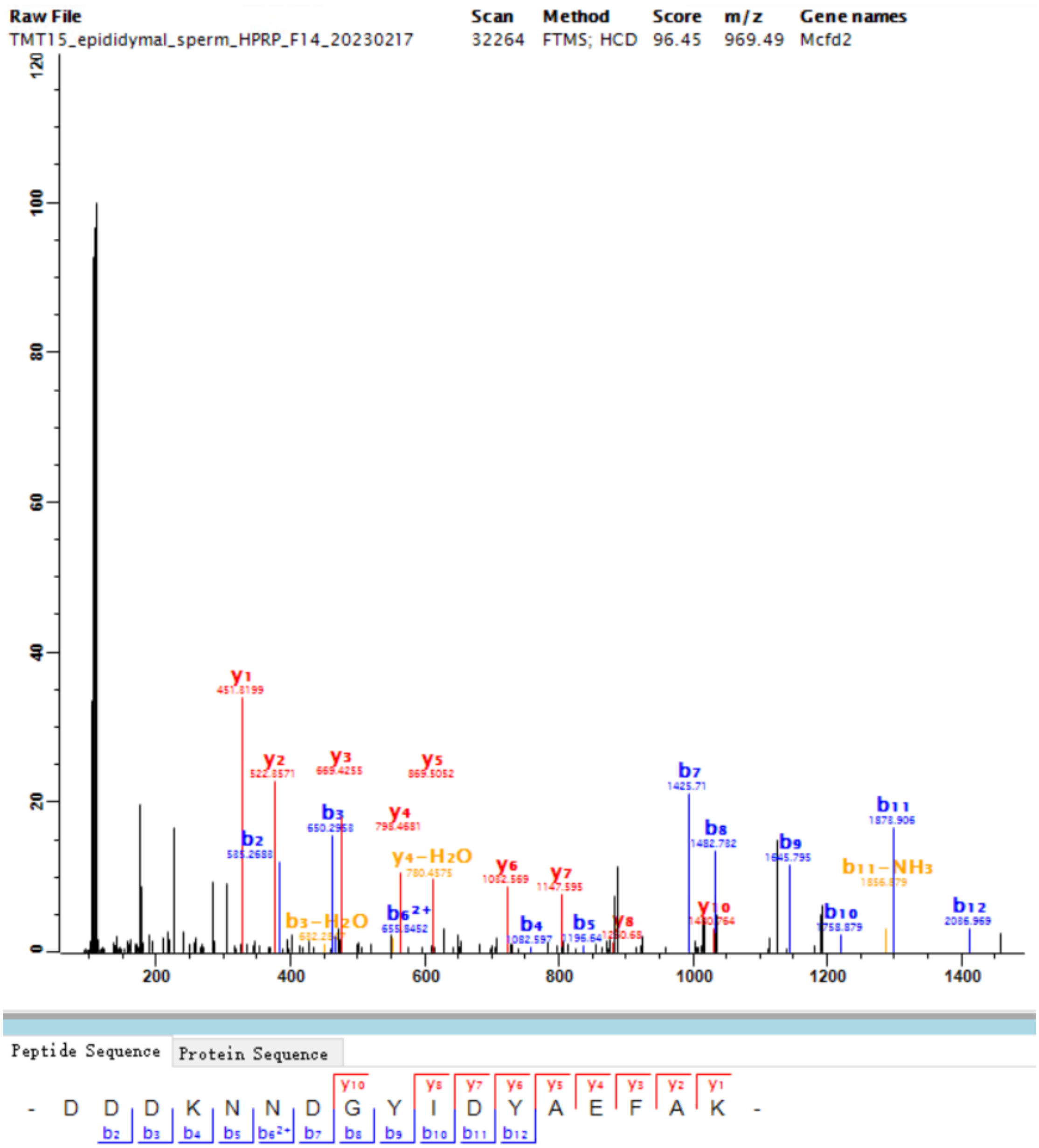
Raw File
TMT15_epididymal_sperm_HPRP_F14_20230217
Scan
32264
Method
FTMS; HCD
Score
96.45
m/z
969.49
Gene names
Mcfd2
y1
y2
y3
y4
y5
y6
y7
y8
y10
b2
b3
b4
b5
b6²⁺
b7
b8
b9
b10
b11
b12
b3−H2O
y4−H2O
b11−NH3
Peptide Sequence
Protein Sequence
- D D D K N N D G Y I D Y A E F A K -

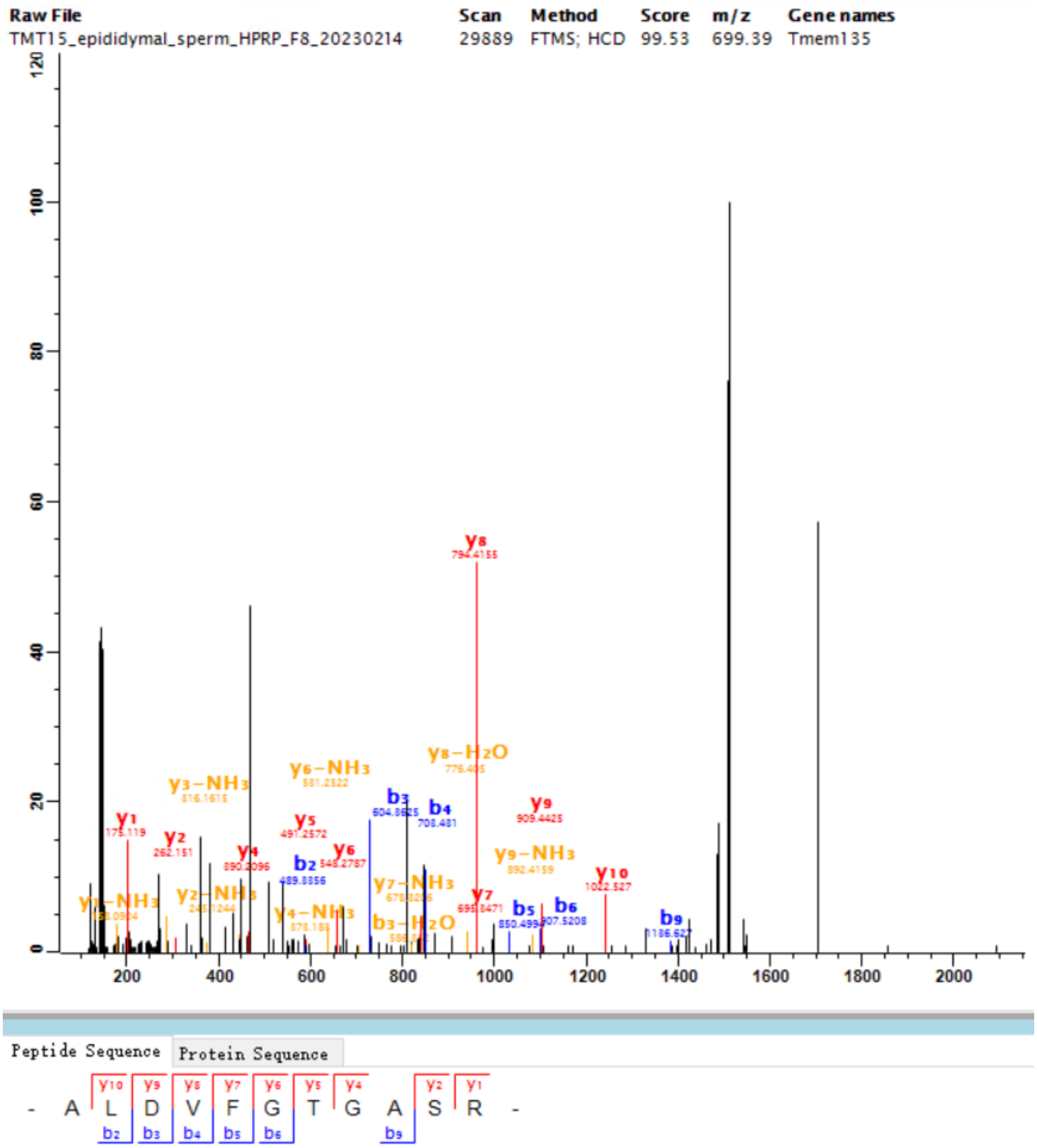

Raw File
TMT15_epididymal_sperm_HPRP_F8_20230214
Scan
29889
Method
FTMS; HCD
Score
99.53
m/z
699.39
Gene names
Tmem135
y8
794.4155
y8−H2O
y6−NH3
y3−NH3
b3
b4
y9
y1
y2
y5
y6
y4
b2
y9−NH3
y7−NH3
y10
y7
b5
b6
b9
y1−NH3
y2−NH3
y4−NH3
b3−H2O
Peptide Sequence
Protein Sequence
- A L D V F G T G A S R -

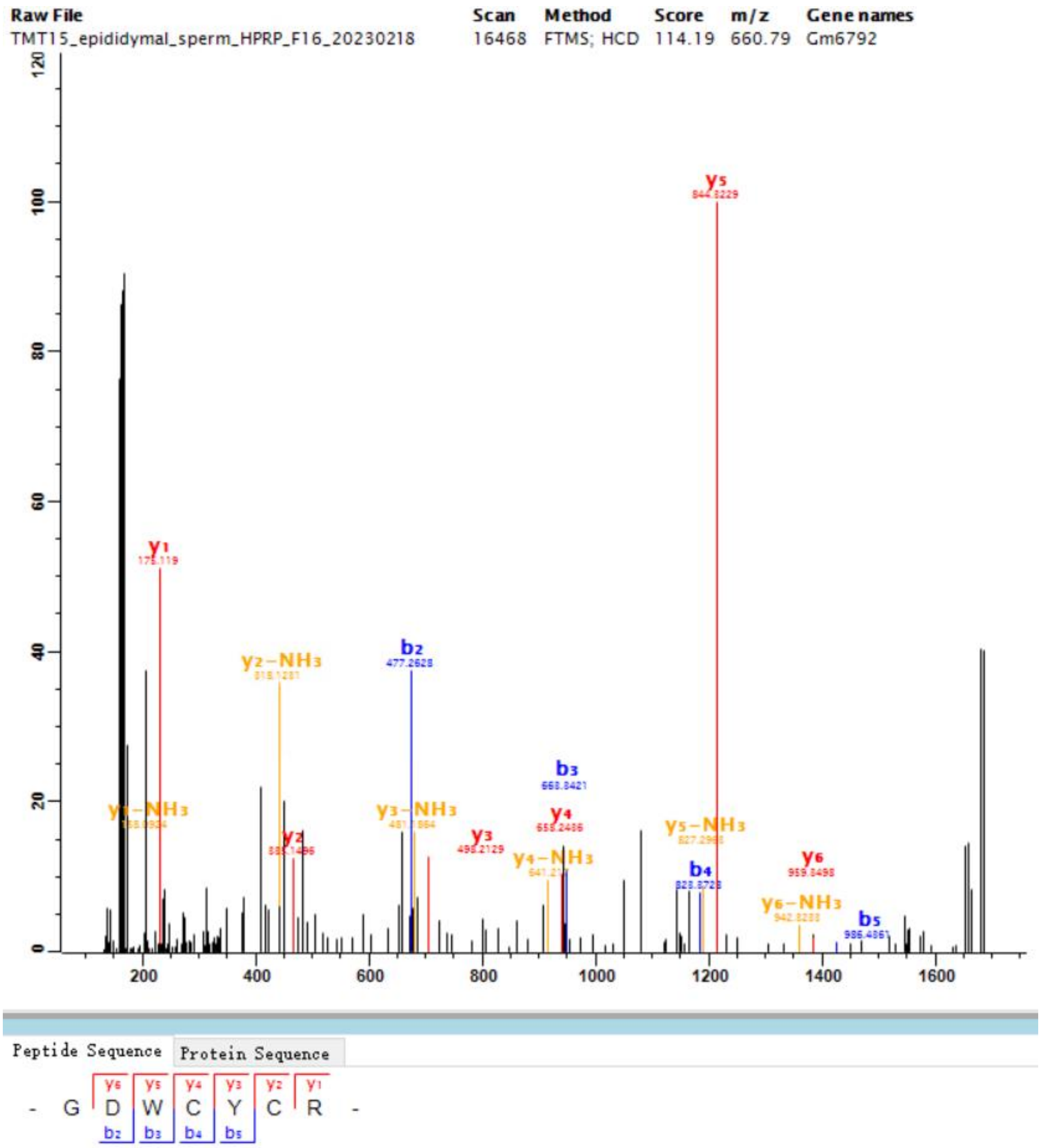

Raw File
TMT15_epididymal_sperm_HPRP_F16_20230218
Scan
16468
Method
FTMS; HCD
Score
114.19
m/z
660.79
Gene names
Gm6792
y1
y1-NH3
y2-NH3
y2
b2
477.2628
y3-NH3
y3
b3
y4
y4-NH3
y5
y5-NH3
b4
y6
y6-NH3
b5
Peptide Sequence
Protein Sequence
- G D W C Y C R -

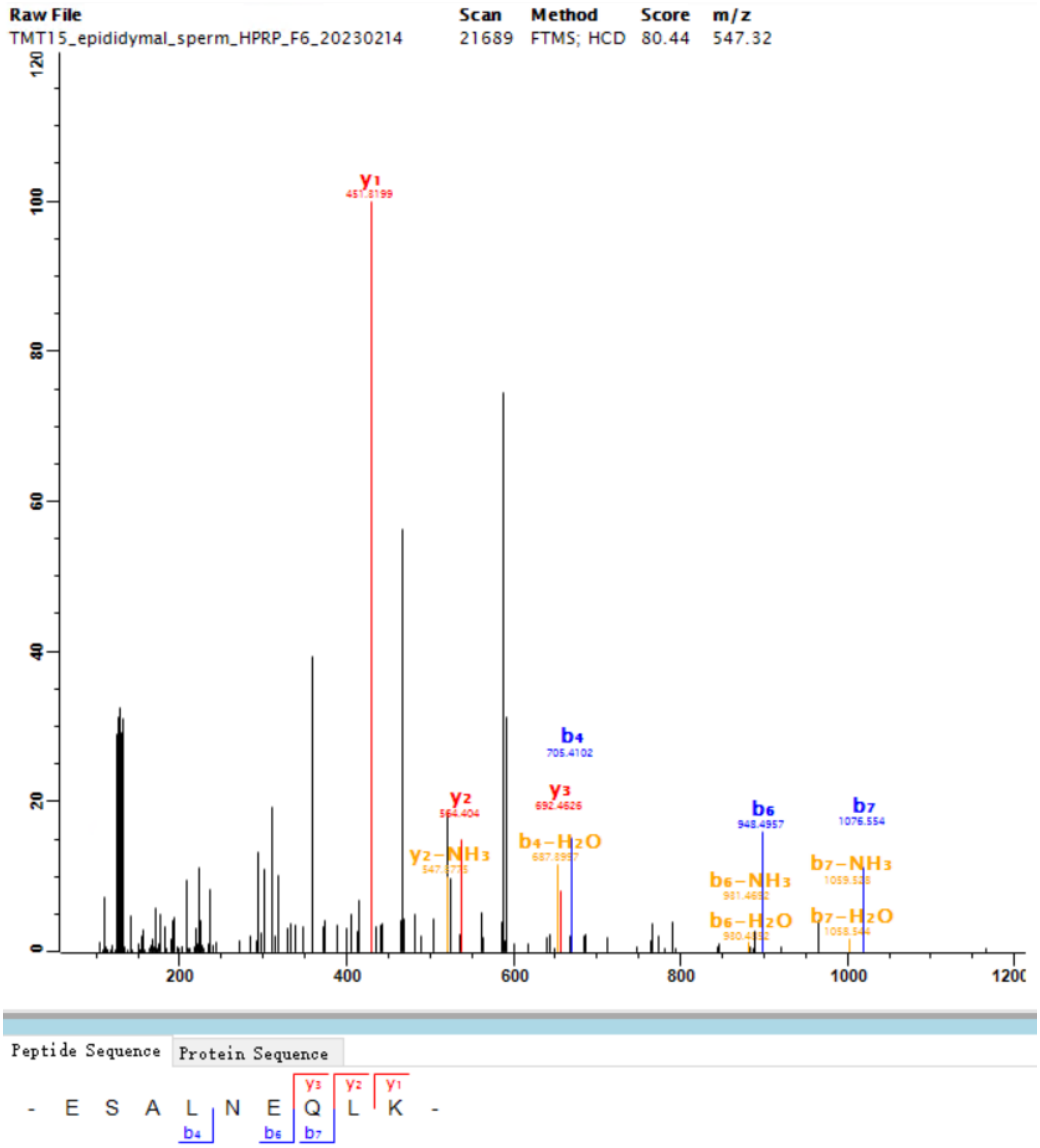

Raw File
TMT15_epididymal_sperm_HPRP_F6_20230214
Scan
21689
Method
FTMS; HCD
Score
80.44
m/z
547.32
0
20
40
60
80
100
120
200
400
600
800
1000
y1
451.8199
y2
y2-NH3
b4
705.4102
y3
692.4626
b4-H2O
b6
948.4957
b6-NH3
b6-H2O
b7
1076.554
b7-NH3
b7-H2O
1058.544
Peptide Sequence
Protein Sequence
- E S A L N E Q L K -
y3 y2 y1
b4 b6 b7

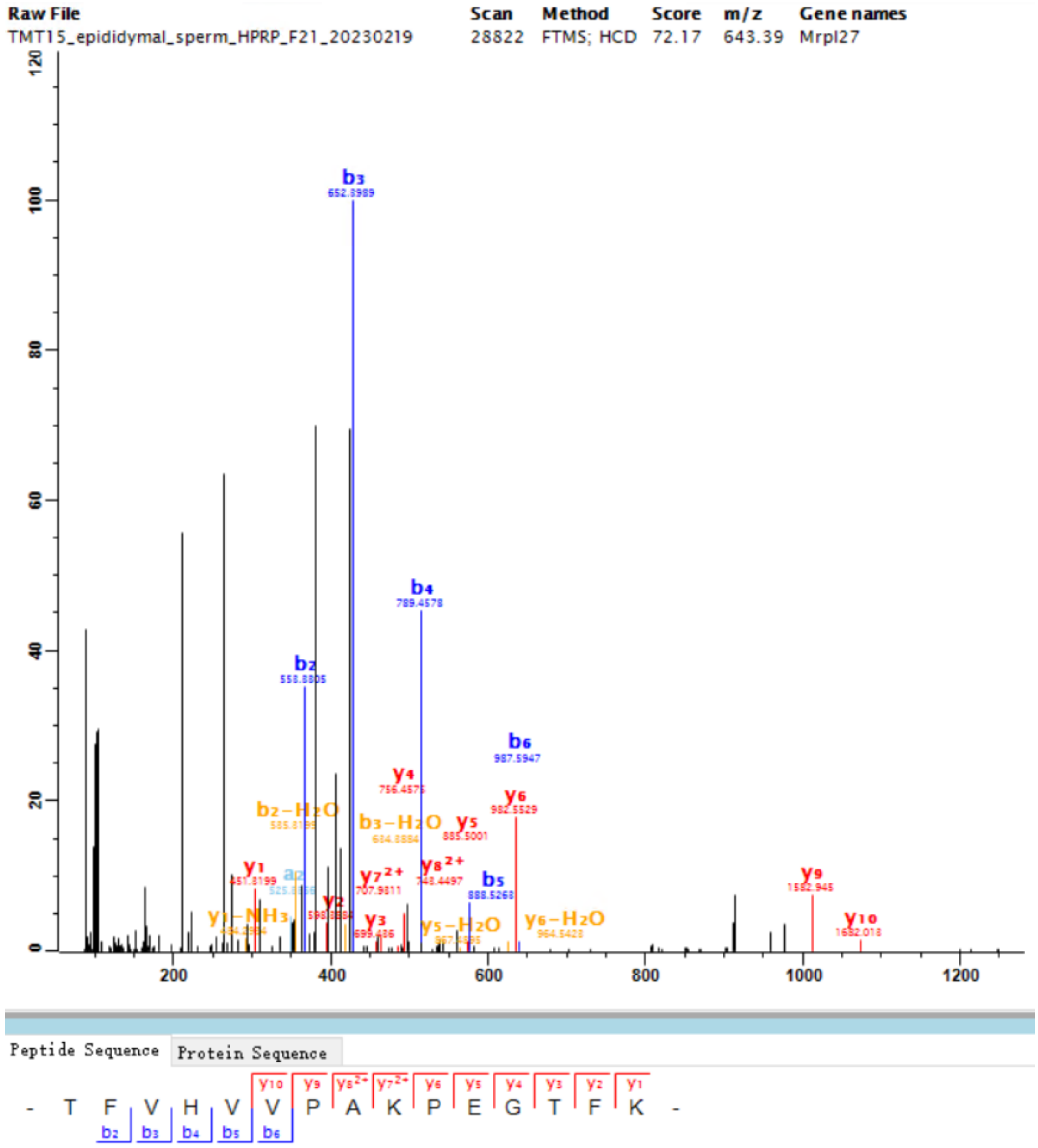
Raw File
TMT15_epididymal_sperm_HPRP_F21_20230219
Scan
28822
Method
FTMS; HCD
Score
72.17
m/z
643.39
Gene names
Mrpl27
b3
652.8989
b4
789.4578
b2
558.8805
b6
987.5947
y4
756.4575
y6
982.5529
b2-H2O
b3-H2O
y5
b5
888.5268
y1
y2
y3
y7 2+
y8 2+
y9
y10
y5-H2O
y6-H2O
964.5428
0
20
40
60
80
100
120
200
400
600
800
1000
1200
Peptide Sequence
Protein Sequence
- T F V H V V P A K P E G T F K -

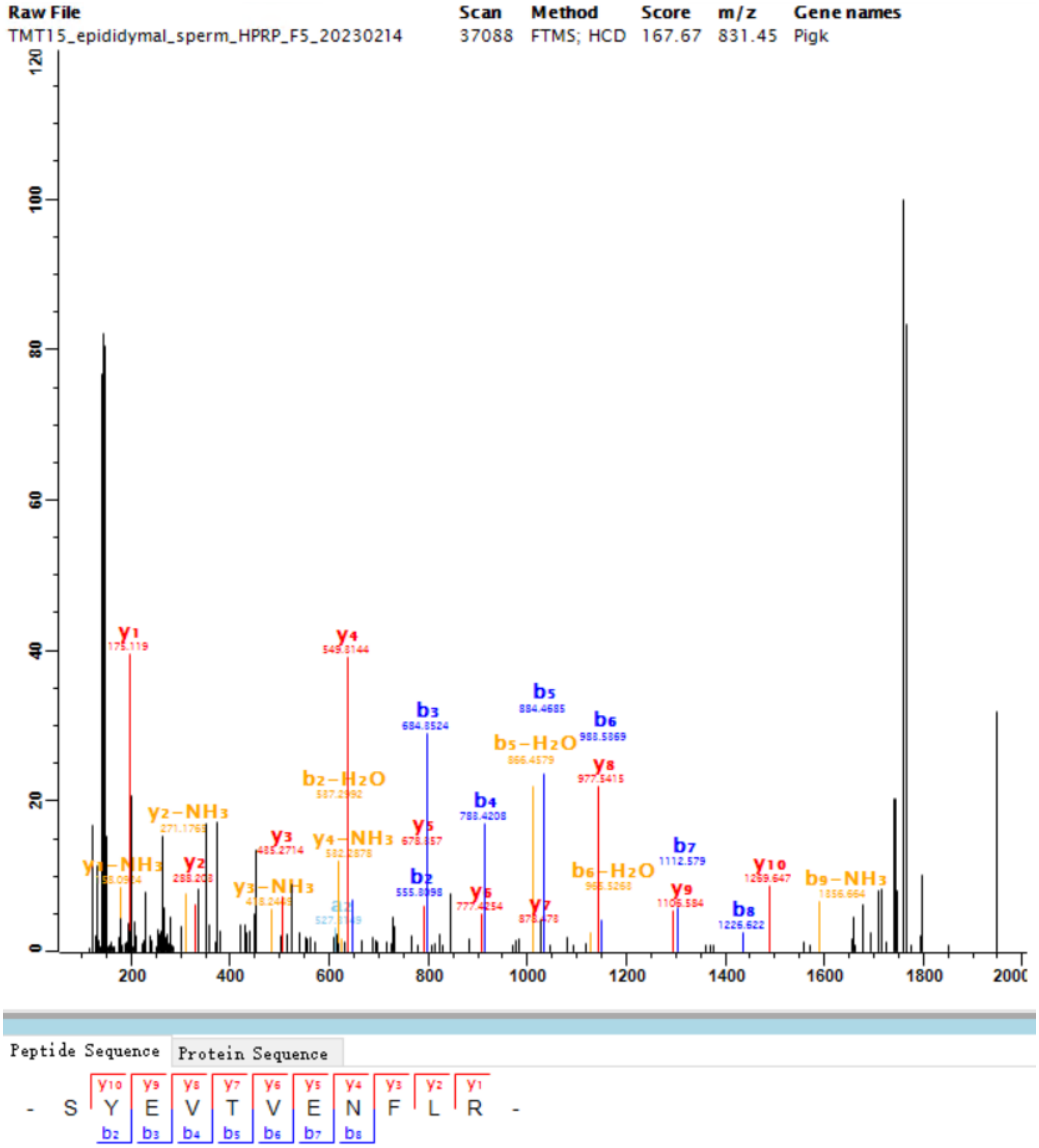

Raw File
TMT15_epididymal_sperm_HPRP_F5_20230214
Scan
37088
Method
FTMS; HCD
Score
167.67
m/z
831.45
Gene names
Pigk
0
20
40
60
80
100
120
200
400
600
800
1000
1200
1400
1600
1800
y1
175.119
y4
549.8144
b3
684.8524
b5
884.4685
b6
988.5869
b5-H2O
866.4579
b2-H2O
587.2992
y8
977.5415
y2-NH3
271.1769
b4
788.4208
y3
y4-NH3
y5
y2
b7
1112.579
y10
b6-H2O
b9-NH3
y3-NH3
b2
555.8098
y6
y9
b8
1226.622
y7
Peptide Sequence
Protein Sequence
- S Y E V T V E N F L R -
y10 y9 y8 y7 y6 y5 y4 y3 y2 y1
b2 b3 b4 b5 b6 b7 b8

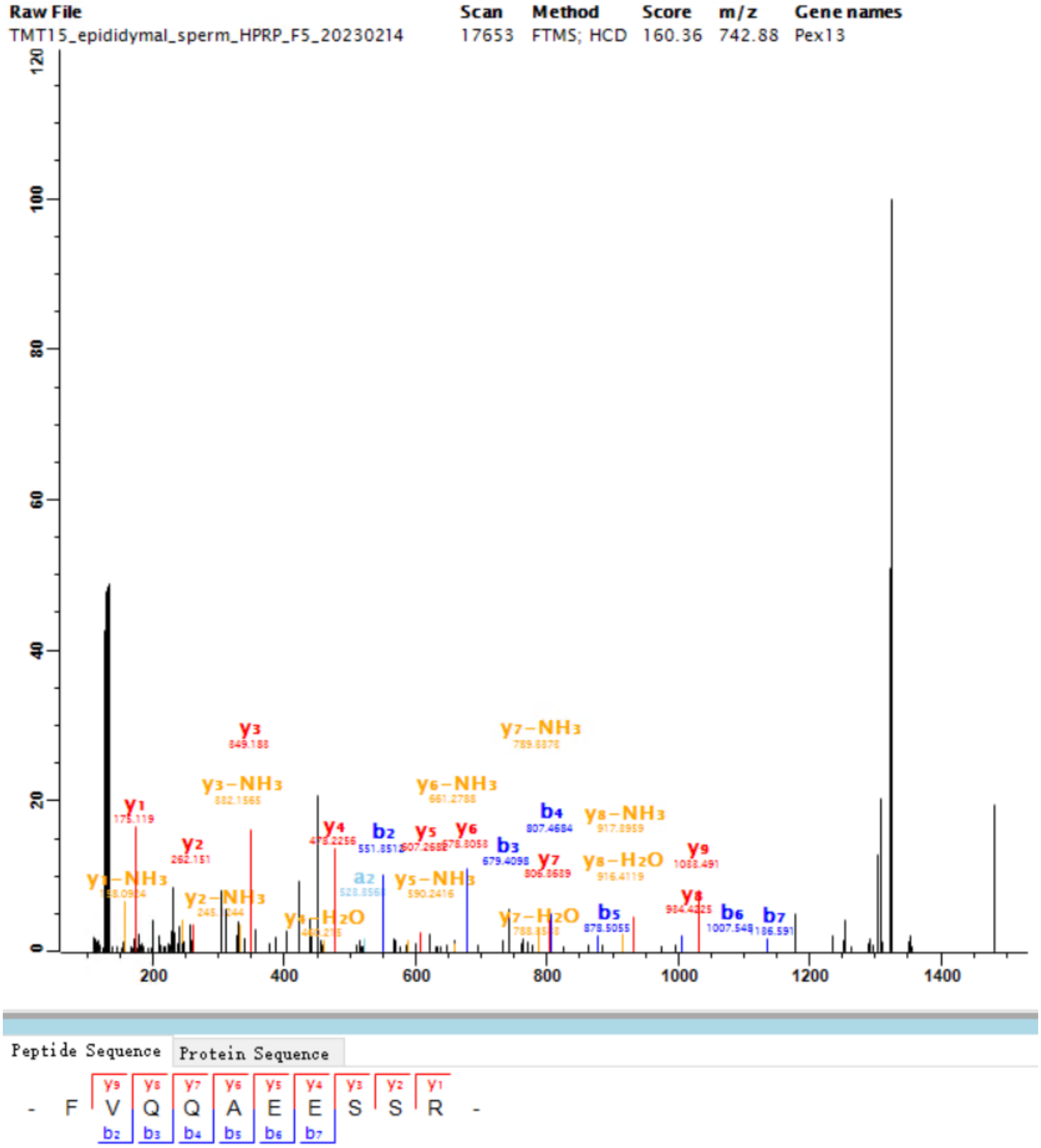

Raw File
TMT15_epididymal_sperm_HPRP_F5_20230214
Scan
17653
Method
FTMS; HCD
Score
160.36
m/z
742.88
Gene names
Pex13
Peptide Sequence
Protein Sequence
- F V Q Q A E E S S R -

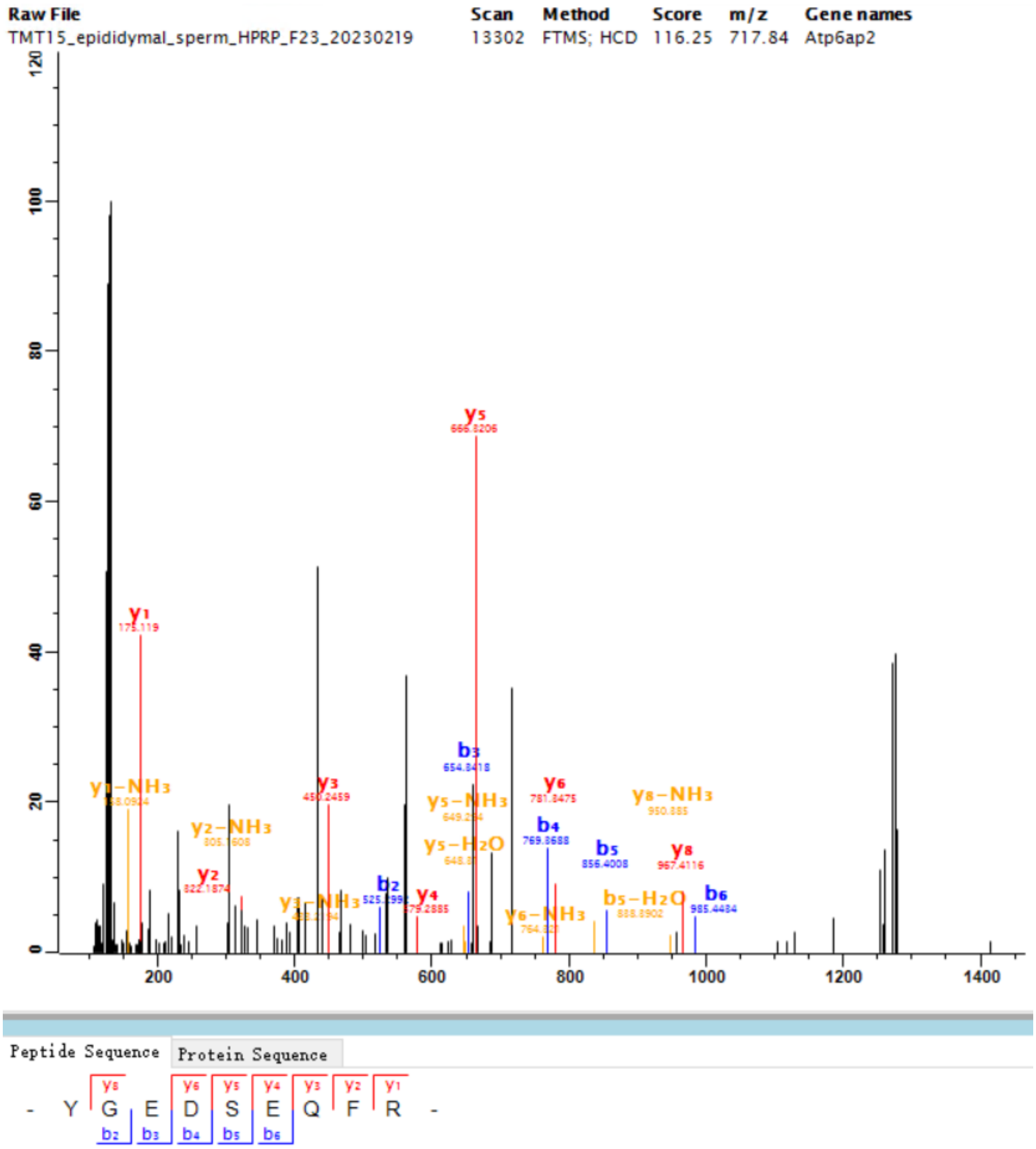
Raw File
TMT15_epididymal_sperm_HPRP_F23_20230219
Scan
13302
Method
FTMS; HCD
Score
116.25
m/z
717.84
Gene names
Atp6ap2
y5
y1
175.119
b3
654.8418
y3
y6
781.3475
y8-NH3
950.385
y5-NH3
y1-NH3
y2-NH3
b4
769.3688
y5-H2O
b5
856.4008
y8
967.4116
y2
b2
y4
b6
985.4484
y3-NH3
y6-NH3
b5-H2O
0
20
40
60
80
100
120
200
400
600
800
1000
1200
1400
Peptide Sequence
Protein Sequence
- Y G E D S E Q F R -

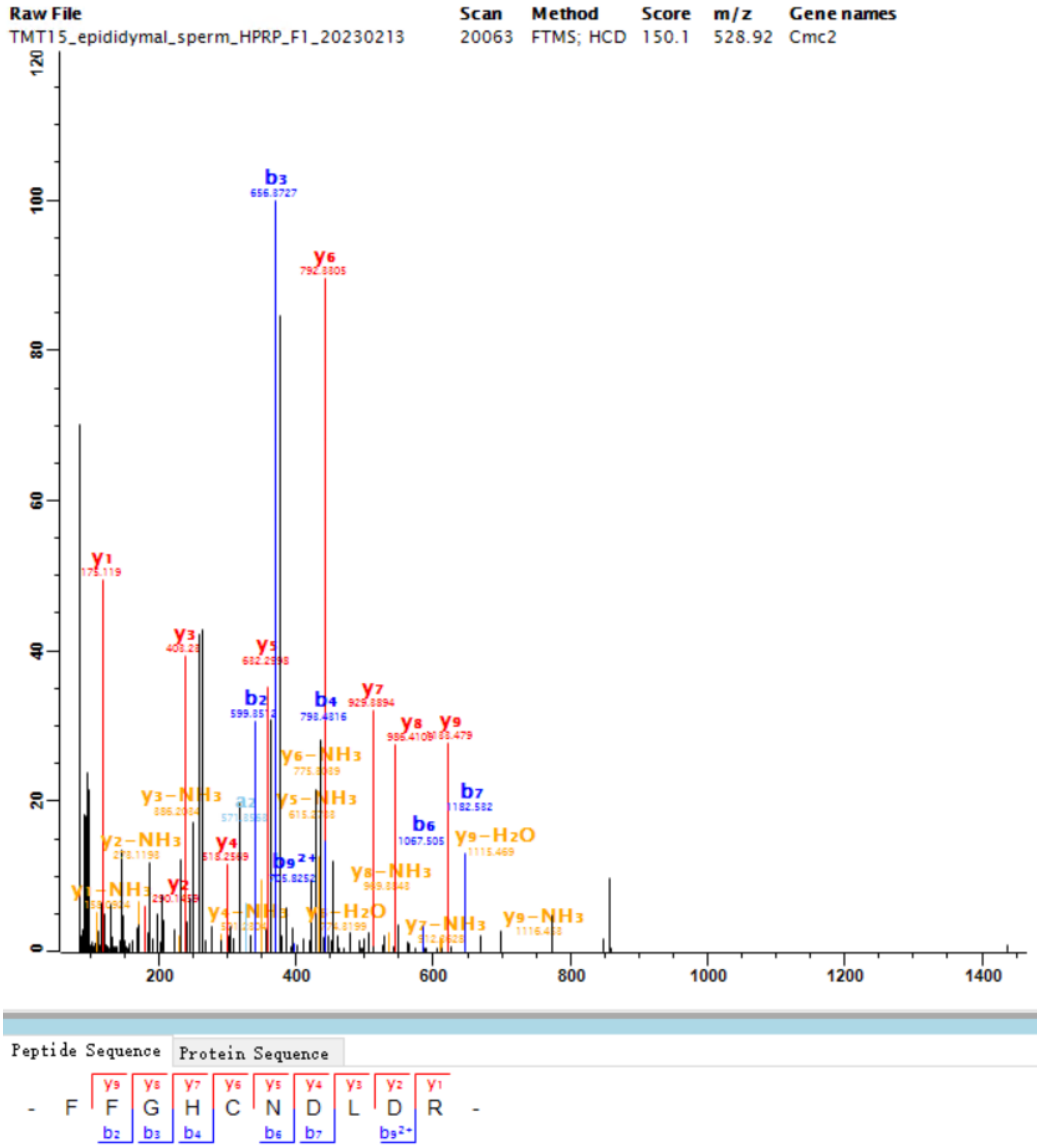
Raw File
TMT15_epididymal_sperm_HPRP_F1_20230213
Scan
20063
Method
FTMS; HCD
Score
150.1
m/z
528.92
Gene names
Cmc2
Peptide Sequence
Protein Sequence
- F F G H C N D L D R -

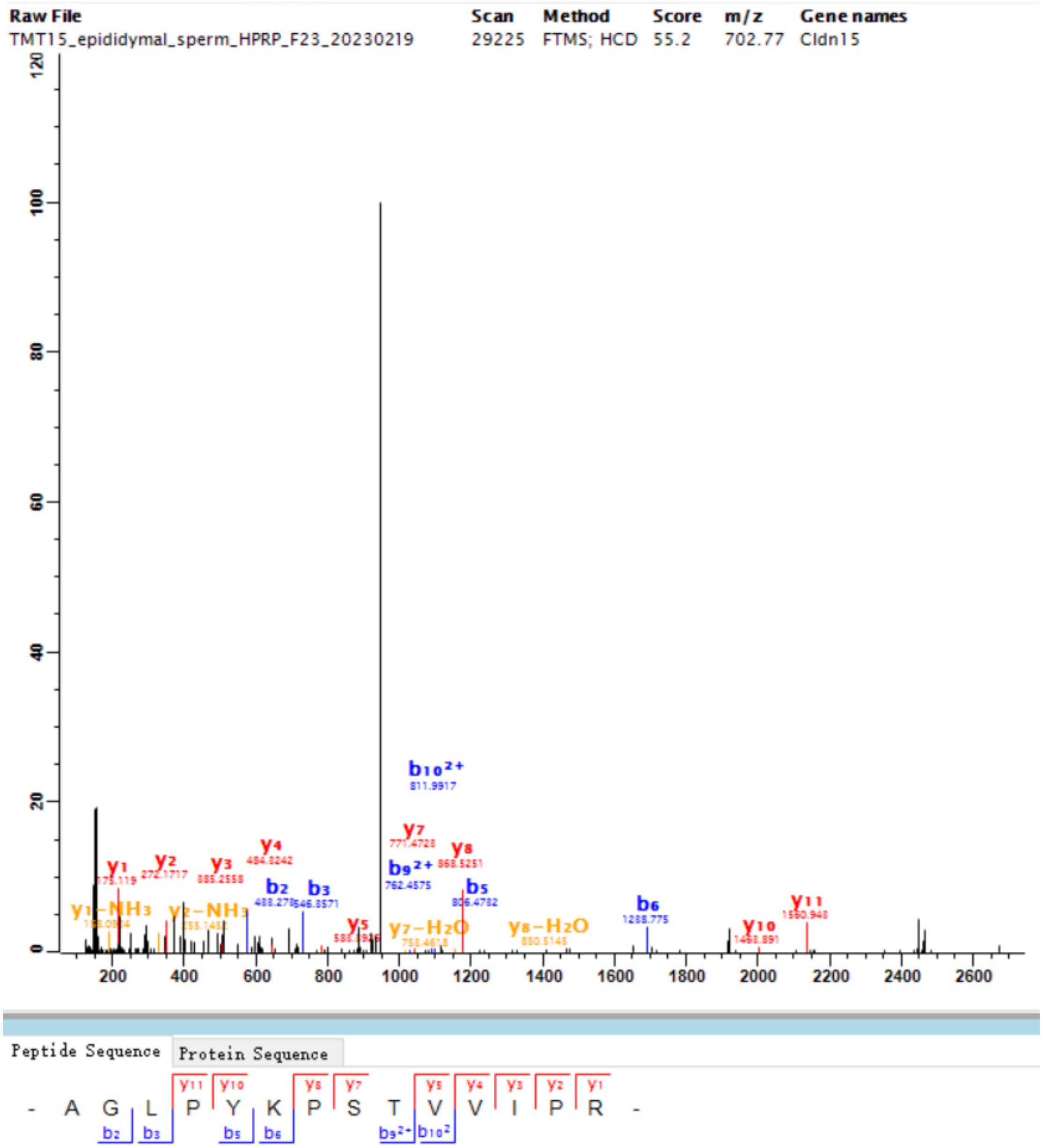
Raw File
TMT15_epididymal_sperm_HPRP_F23_20230219
Scan
29225
Method
FTMS; HCD
Score
55.2
m/z
702.77
Gene names
Cldn15
b10 2+
811.9917
y7
771.4728
y8
868.5251
b9 2+
762.4575
b5
806.4782
y1
175.119
y2
272.1717
y3
385.2558
y4
484.8242
b2
488.278
b3
546.8571
b6
1288.775
y11
1580.948
y1-NH3
y2-NH3
y5
y7-H2O
y8-H2O
850.5145
y10
1498.891
Peptide Sequence
Protein Sequence
- A G L P Y K P S T V V I P R -

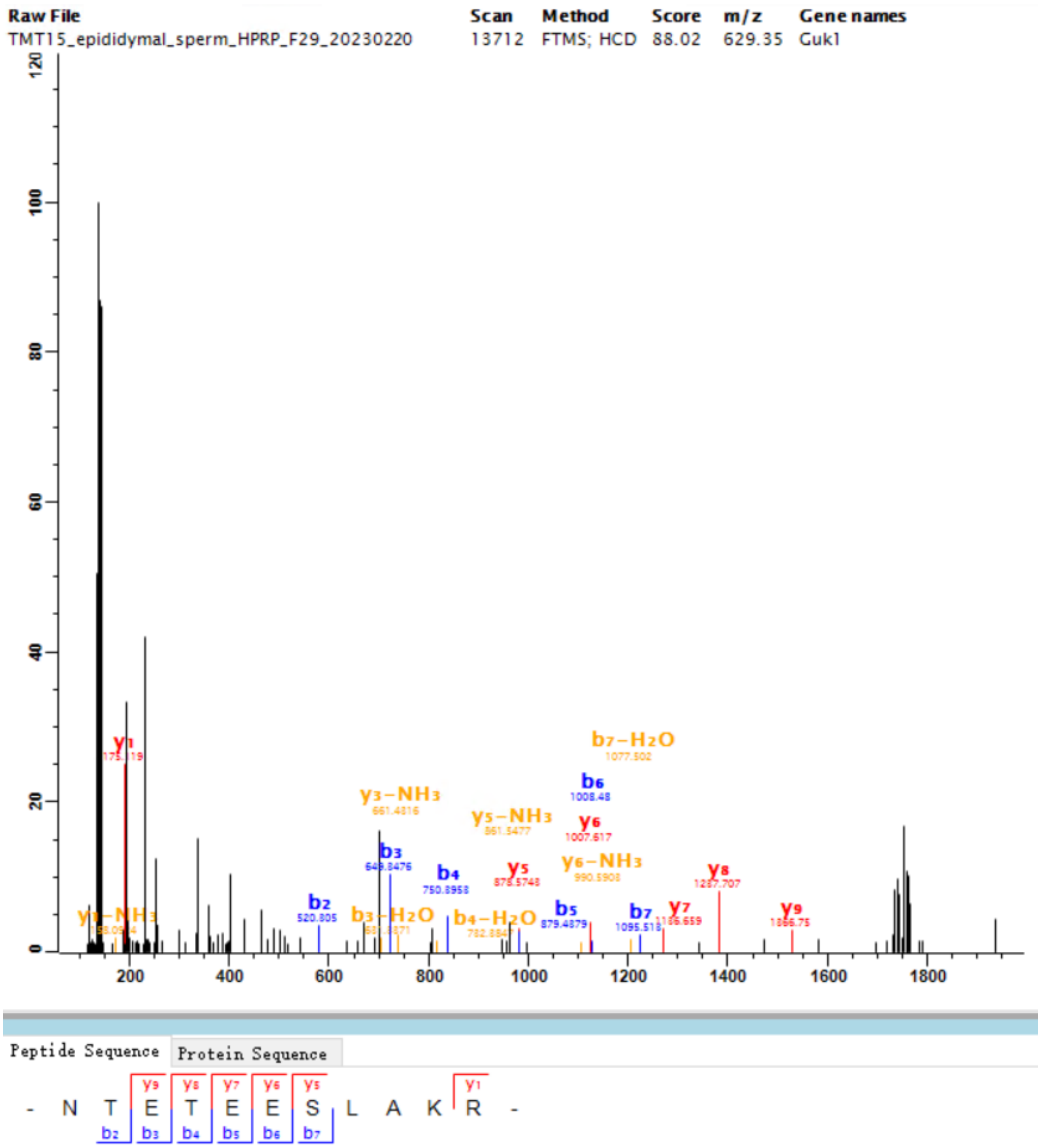

Raw File
TMT15_epididymal_sperm_HPRP_F29_20230220
Scan Method Score m/z Gene names
13712 FTMS; HCD 88.02 629.35 Guk1
b7-H2O
1077.502
b6
1008.48
y3-NH3
661.4816
y5-NH3
861.5477
y6
1007.617
y6-NH3
990.5908
b4
750.8958
b2
520.805
b3-H2O
b4-H2O
b5
879.4879
b7
1095.518
y7
y8
y9
Peptide Sequence
Protein Sequence
- N T E T E E S L A K R -

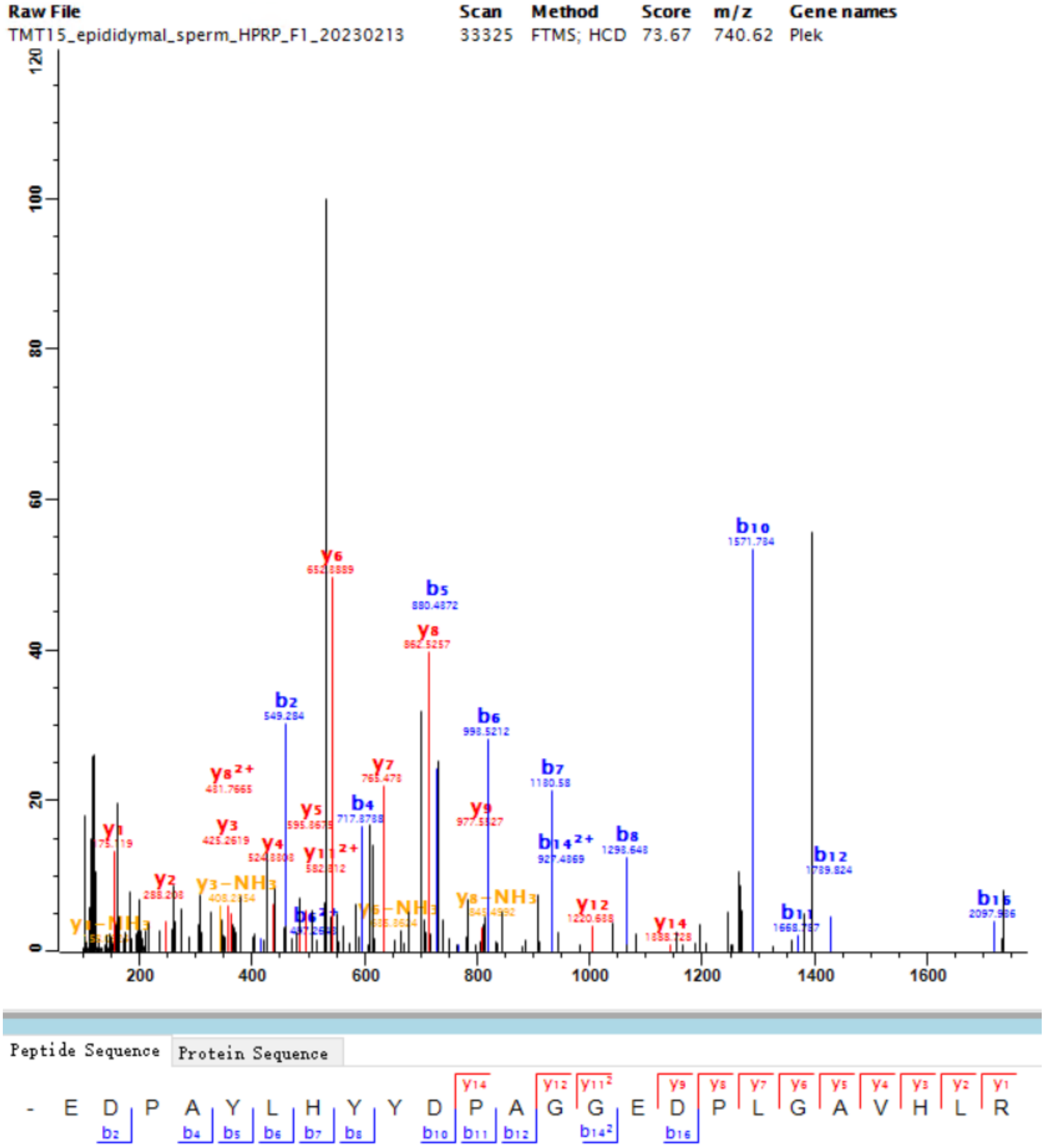

Raw File
TMT15_epididymal_sperm_HPRP_F1_20230213
Scan
33325
Method
FTMS; HCD
Score
73.67
m/z
740.62
Gene names
Plek
Peptide Sequence
Protein Sequence
- E D P A Y L H Y Y D P A G G E D P L G A V H L R

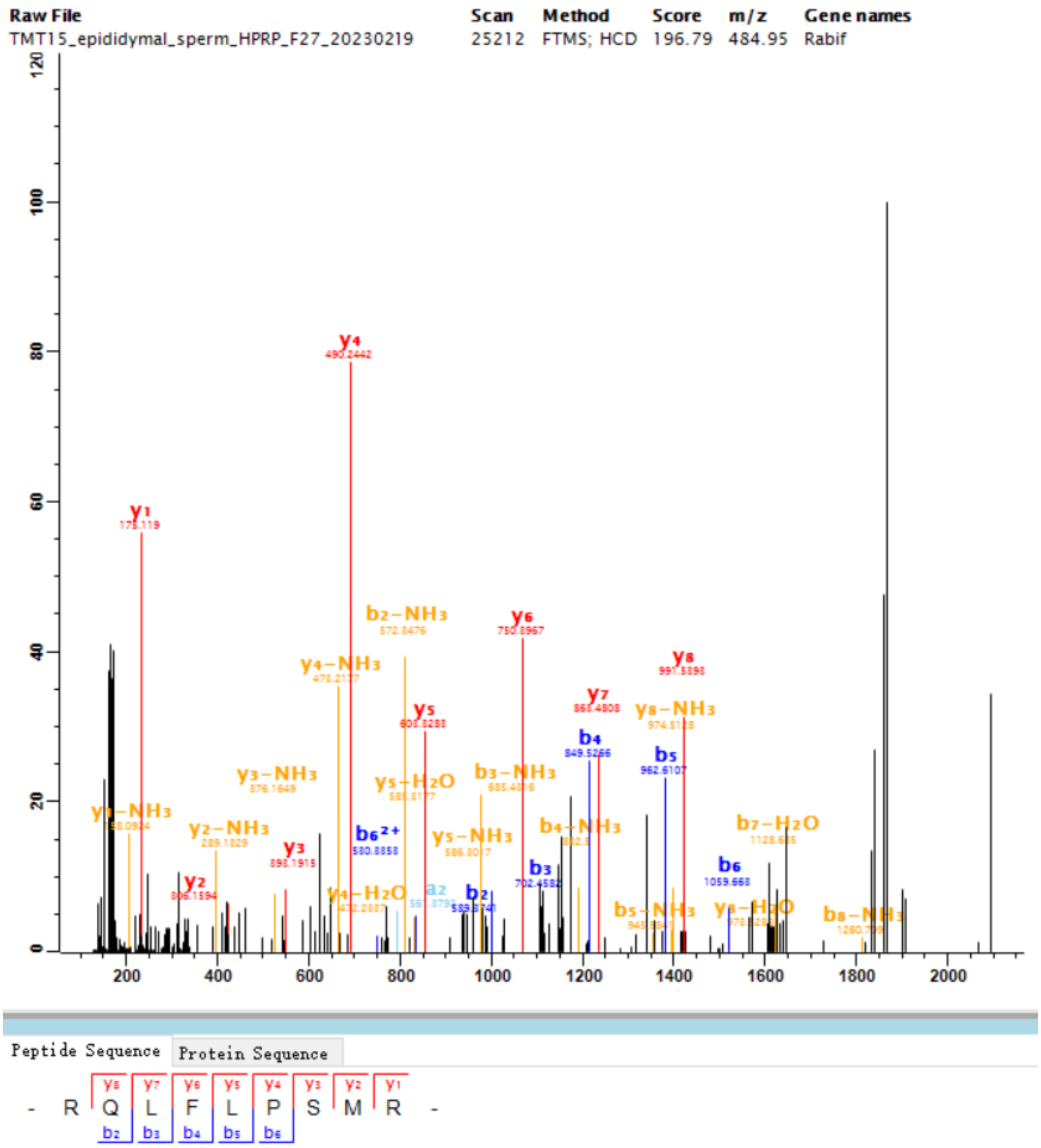

Raw File
TMT15_epididymal_sperm_HPRP_F27_20230219
Scan Method Score m/z Gene names
25212 FTMS; HCD 196.79 484.95 Rabif
y4 490.2442
y1 175.119
b2-NH3 572.8476
y6 750.8967
y4-NH3
y8 991.5898
y7
y8-NH3
y5
b4 849.5266
b5 962.6107
y3-NH3
y5-H2O
b3-NH3
b7-H2O
y2-NH3
y3
b6 2+ 580.8858
y5-NH3
b4-NH3
b3 702.4582
b6 1059.668
y2
a2
b2
b5-NH3
b8-NH3
Peptide Sequence
Protein Sequence
- R Q L F L P S M R -

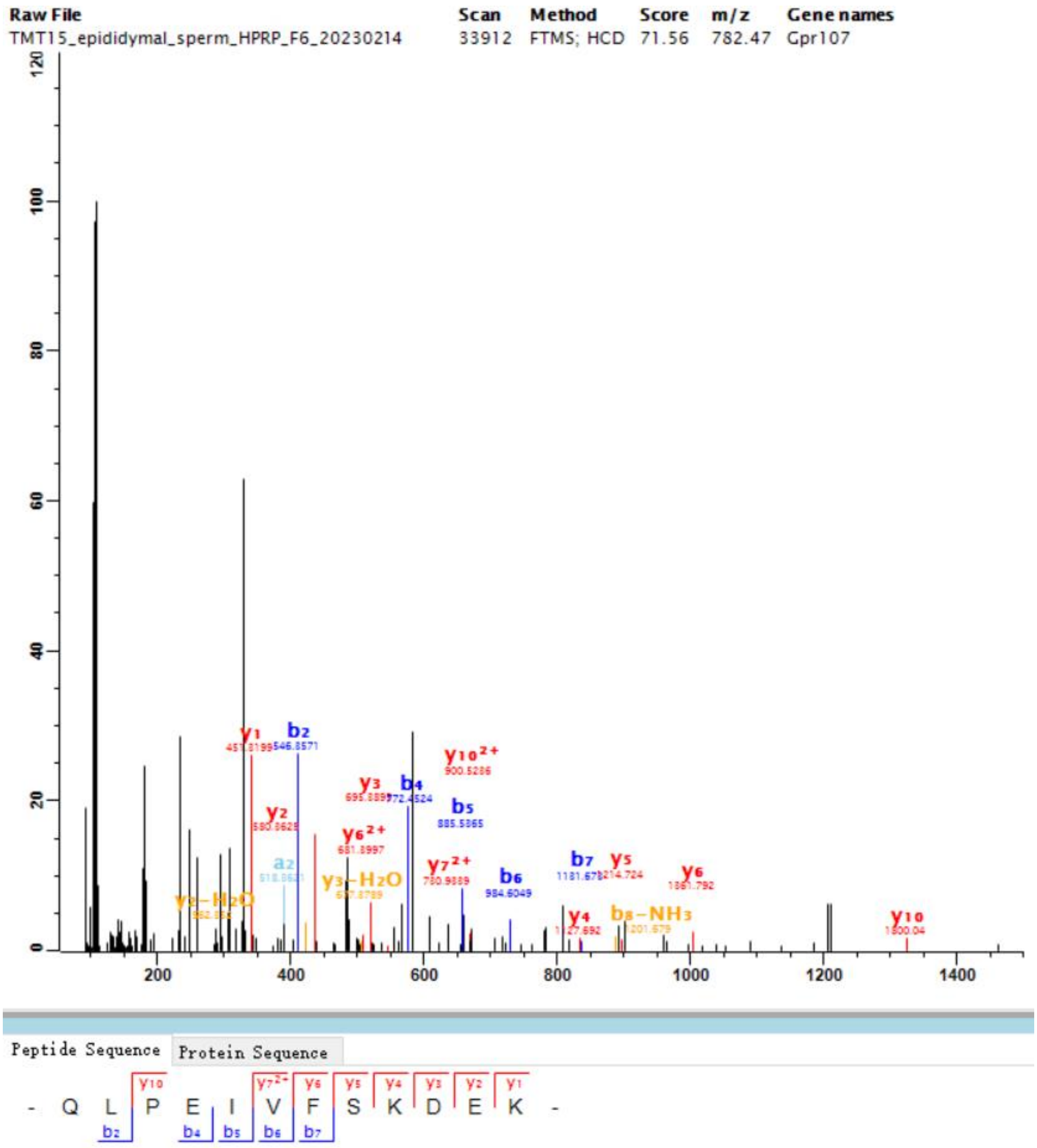
Raw File
TMT15_epididymal_sperm_HPRP_F6_20230214
Scan
33912
Method
FTMS; HCD
Score
71.56
m/z
782.47
Gene names
Gpr107
y1
b2
546.8571
y10 2+
900.5286
y3
b4
b5
885.5865
y2
y6 2+
y7 2+
b6
984.6049
b7
y5
y6
a2
y3-H2O
y4
b8-NH3
y10
200
400
600
800
1000
1200
1400
Peptide Sequence
Protein Sequence
- Q L P E I V F S K D E K -

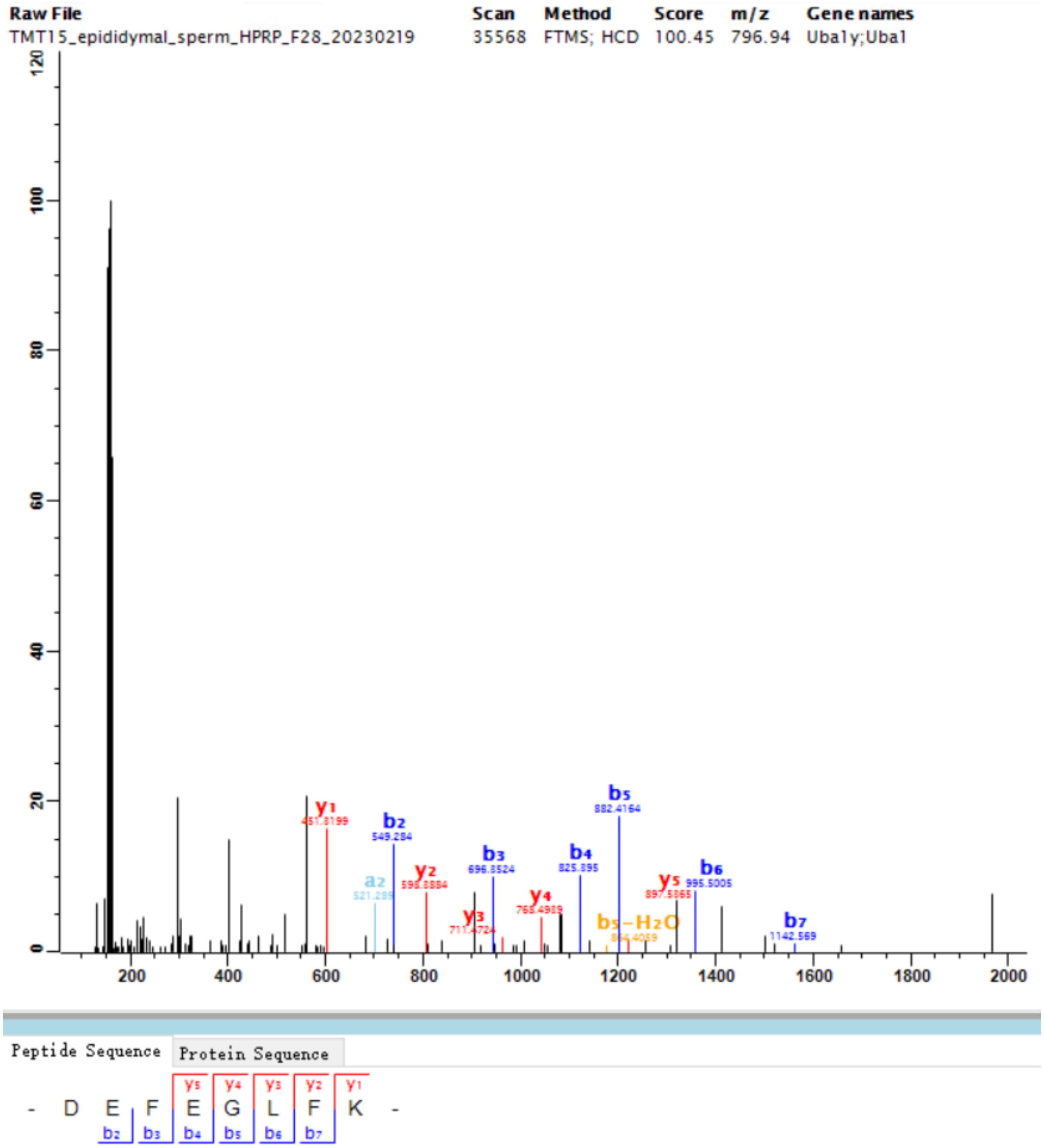

Raw File
TMT15_epididymal_sperm_HPRP_F28_20230219
Scan
35568
Method
FTMS; HCD
Score
100.45
m/z
796.94
Gene names
Uba1y;Uba1
0
20
40
60
80
100
120
200
400
600
800
1000
1200
1400
1600
1800
2000
y1
a2
521.289
b2
549.284
y2
y3
711.4724
b3
696.8524
y4
b4
825.895
b5
882.4164
b5−H2O
y5
b6
995.5005
b7
1142.569
Peptide Sequence
Protein Sequence
- D E F E G L F K -
y5 y4 y3 y2 y1
b2 b3 b4 b5 b6 b7

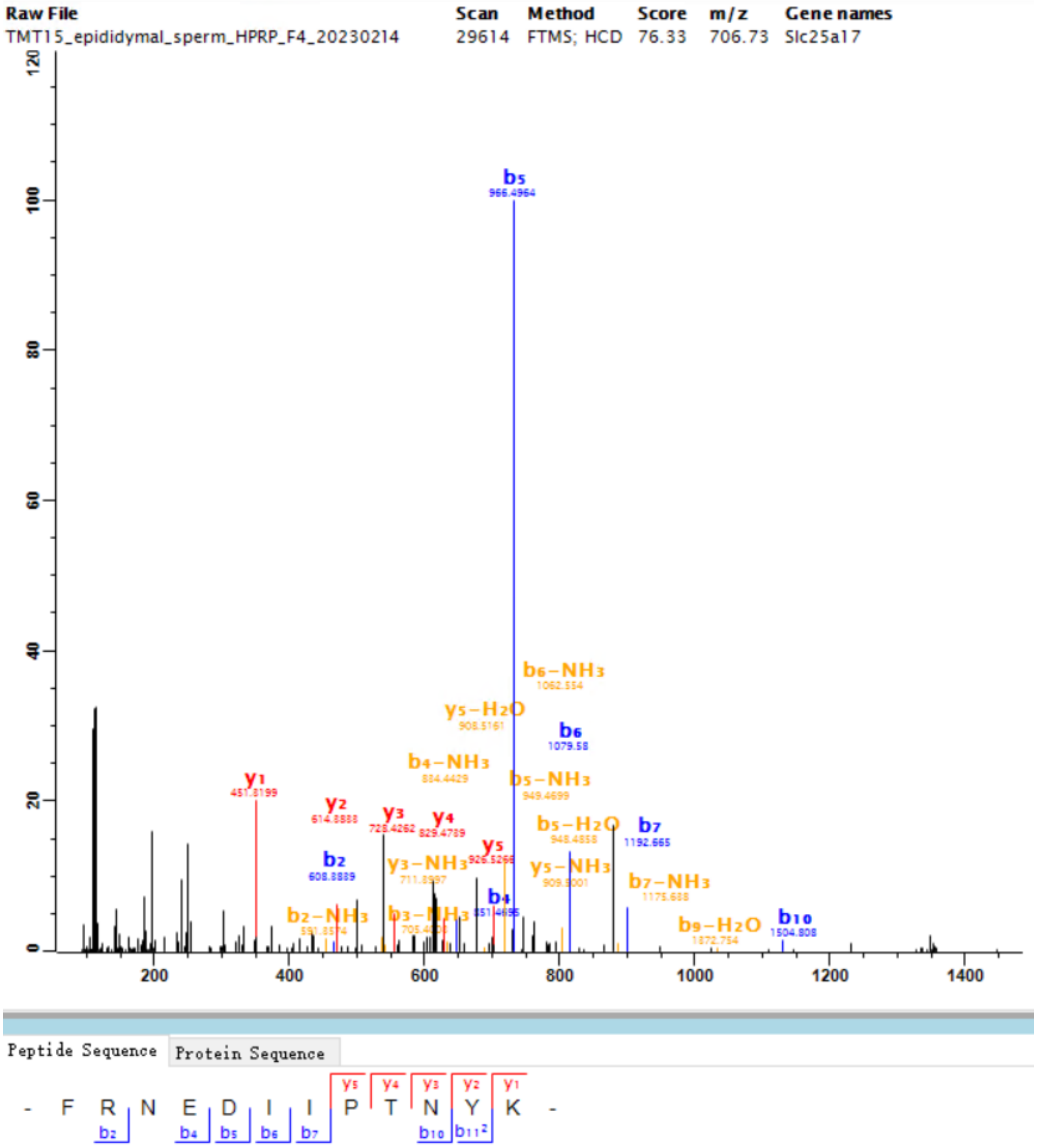
Raw File
TMT15_epididymal_sperm_HPRP_F4_20230214
Scan
29614
Method
FTMS; HCD
Score
76.33
m/z
706.73
Gene names
Slc25a17
b5
966.4964
b6-NH3
1062.554
y5-H2O
908.5161
b6
1079.58
b4-NH3
884.4429
b5-NH3
949.4699
y1
451.8199
y2
614.8888
y3
728.4262
y4
829.4789
b5-H2O
948.4858
b7
1192.665
b2
608.8889
y5
926.5266
y3-NH3
711.3997
y5-NH3
909.5001
b7-NH3
1175.638
b4
b2-NH3
b3-NH3
b9-H2O
1372.754
b10
1504.808
200
400
600
800
1000
1200
1400
0
20
40
60
80
100
120
Peptide Sequence
Protein Sequence
- F R N E D I I P T N Y K -
y5 y4 y3 y2 y1
b2 b4 b5 b6 b7 b10 b11²

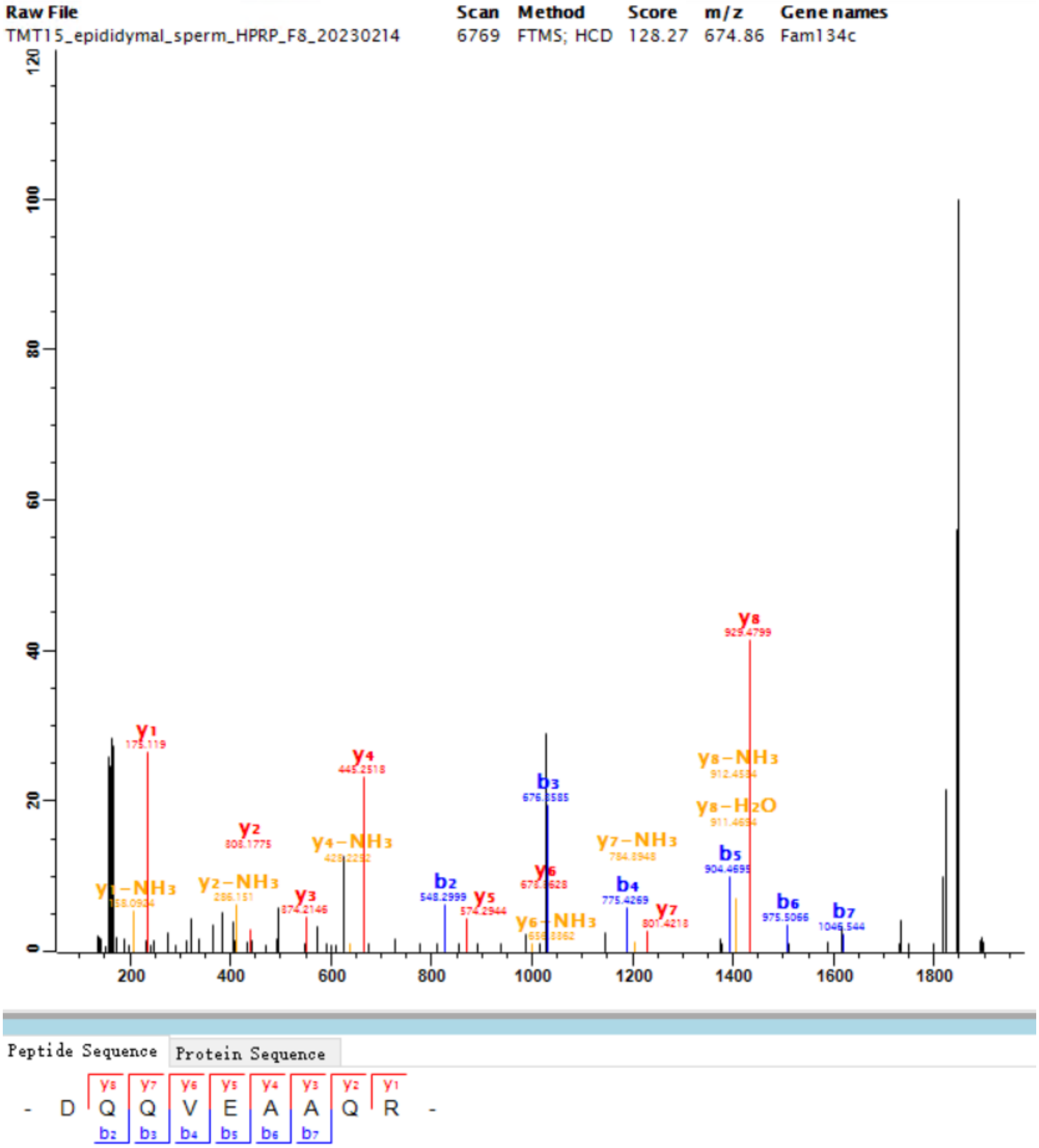
Raw File
TMT15_epididymal_sperm_HPRP_F8_20230214
Scan
6769
Method
FTMS; HCD
Score
128.27
m/z
674.86
Gene names
Fam134c
y8
929.4799
y1
175.119
y4
445.2518
b3
676.3585
y8-NH3
912.4534
y8-H2O
911.4694
y2
303.1775
y4-NH3
y7-NH3
b5
904.4695
b2
548.2999
y5
574.2944
b4
775.4269
y7
801.4218
b6
975.5066
b7
1046.544
y1-NH3
y2-NH3
286.151
y3
374.2146
y6-NH3
200
400
600
800
1000
1200
1400
1600
1800
0
20
40
60
80
100
120
Peptide Sequence
Protein Sequence
- D Q Q V E A A Q R -

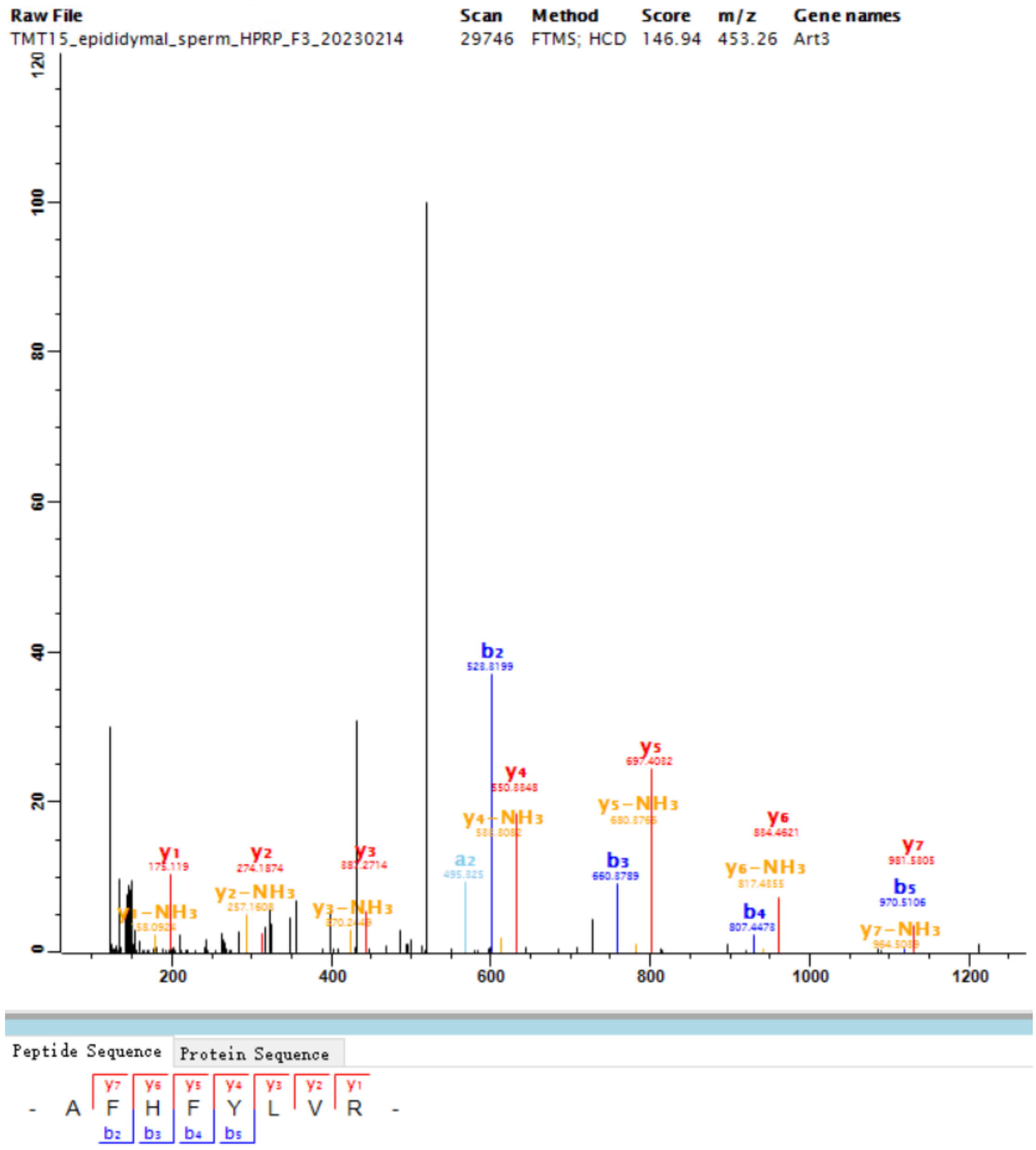

Raw File
TMT15_epididymal_sperm_HPRP_F3_20230214
Scan
29746
Method
FTMS; HCD
Score
146.94
m/z
453.26
Gene names
Art3
b2
y4
y5
y4-NH3
y5-NH3
y6
y7
a2
b3
b4
b5
y1
y2
y3
y1-NH3
y2-NH3
y3-NH3
y6-NH3
y7-NH3
Peptide Sequence
Protein Sequence
- A F H F Y L V R -

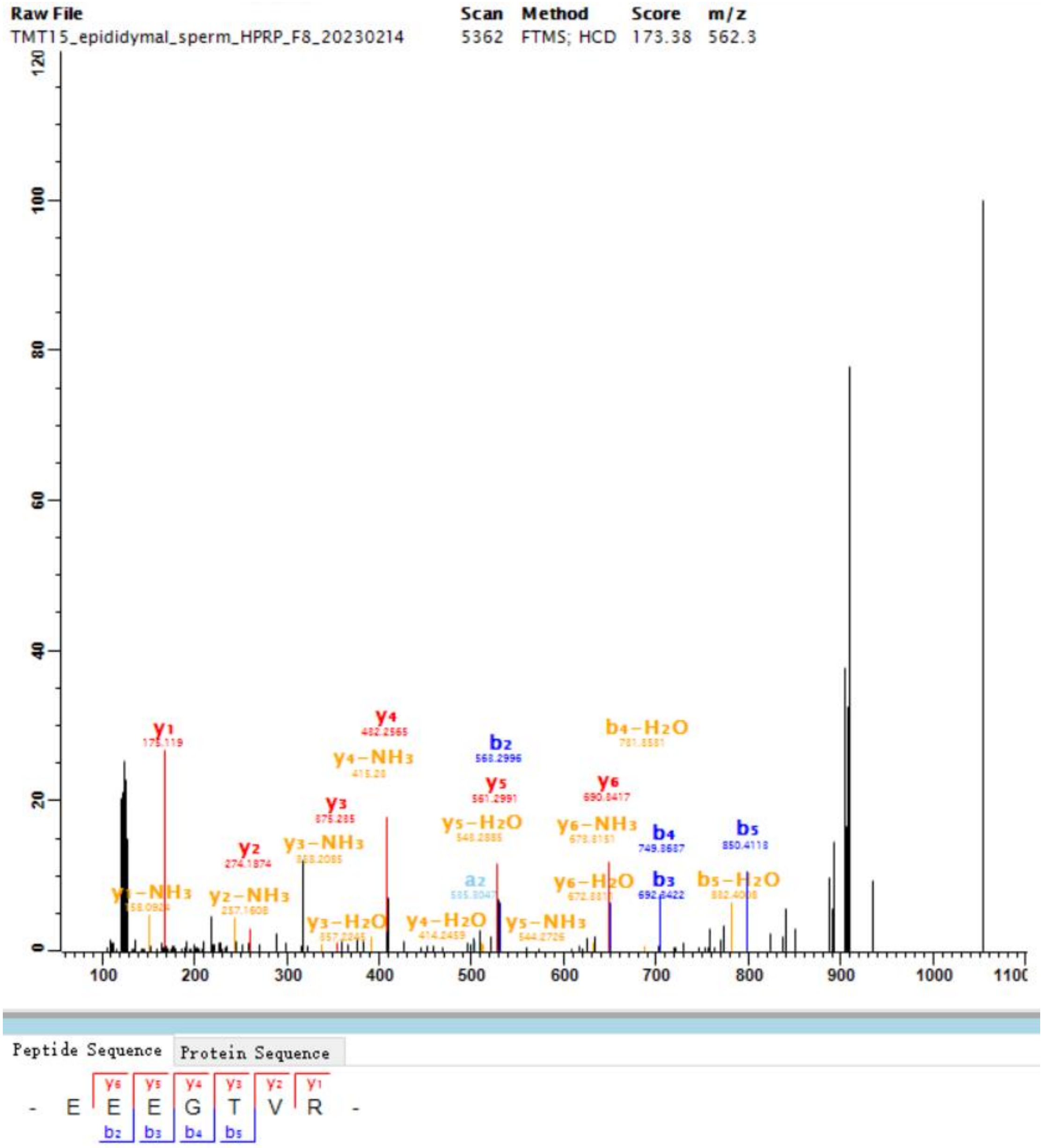

Raw File
TMT15_epididymal_sperm_HPRP_F8_20230214
Scan
5362
Method
FTMS; HCD
Score
173.38
m/z
562.3
y1
y2
y3
y4
y5
y6
y2-NH3
y3-NH3
y4-NH3
y5-NH3
y6-NH3
y3-H2O
y4-H2O
y5-H2O
y6-H2O
a2
b2
b3
b4
b5
b4-H2O
b5-H2O
Peptide Sequence
Protein Sequence
- E E E G T V R -

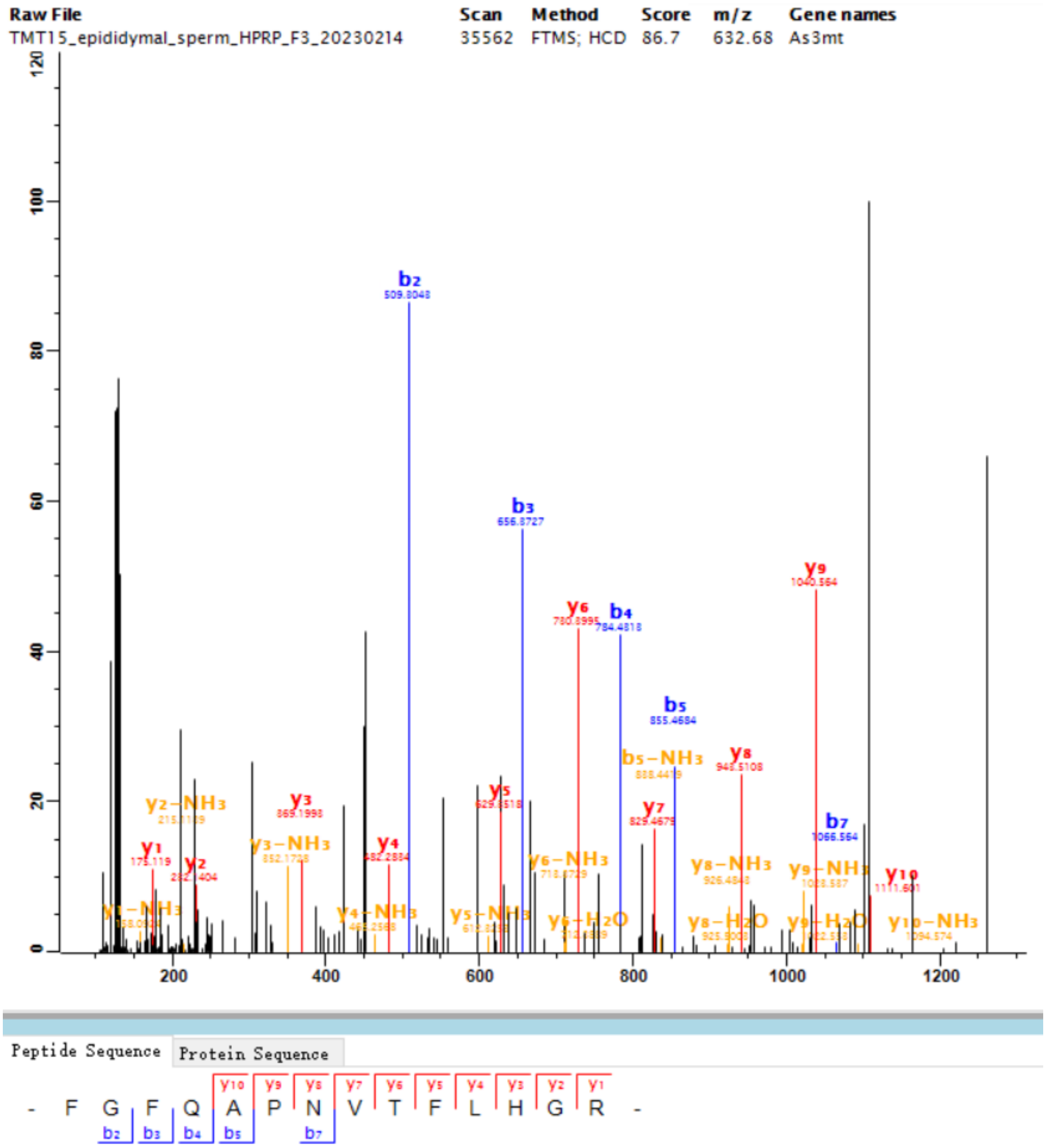
Raw File
TMT15_epididymal_sperm_HPRP_F3_20230214
Scan
35562
Method
FTMS; HCD
Score
86.7
m/z
632.68
Gene names
As3mt
b2
509.8048
b3
656.8727
y6
780.8995
b4
784.4818
y9
b5
855.4684
b5-NH3
y8
y7
b7
1066.564
y10
1111.601
y1
175.119
y2
y3
y4
y5
y2-NH3
y3-NH3
y4-NH3
y5-NH3
y6-NH3
y8-NH3
y9-NH3
y1-NH3
y6-H2O
y8-H2O
y9-H2O
y10-NH3
Peptide Sequence
Protein Sequence
- F G F Q A P N V T F L H G R -

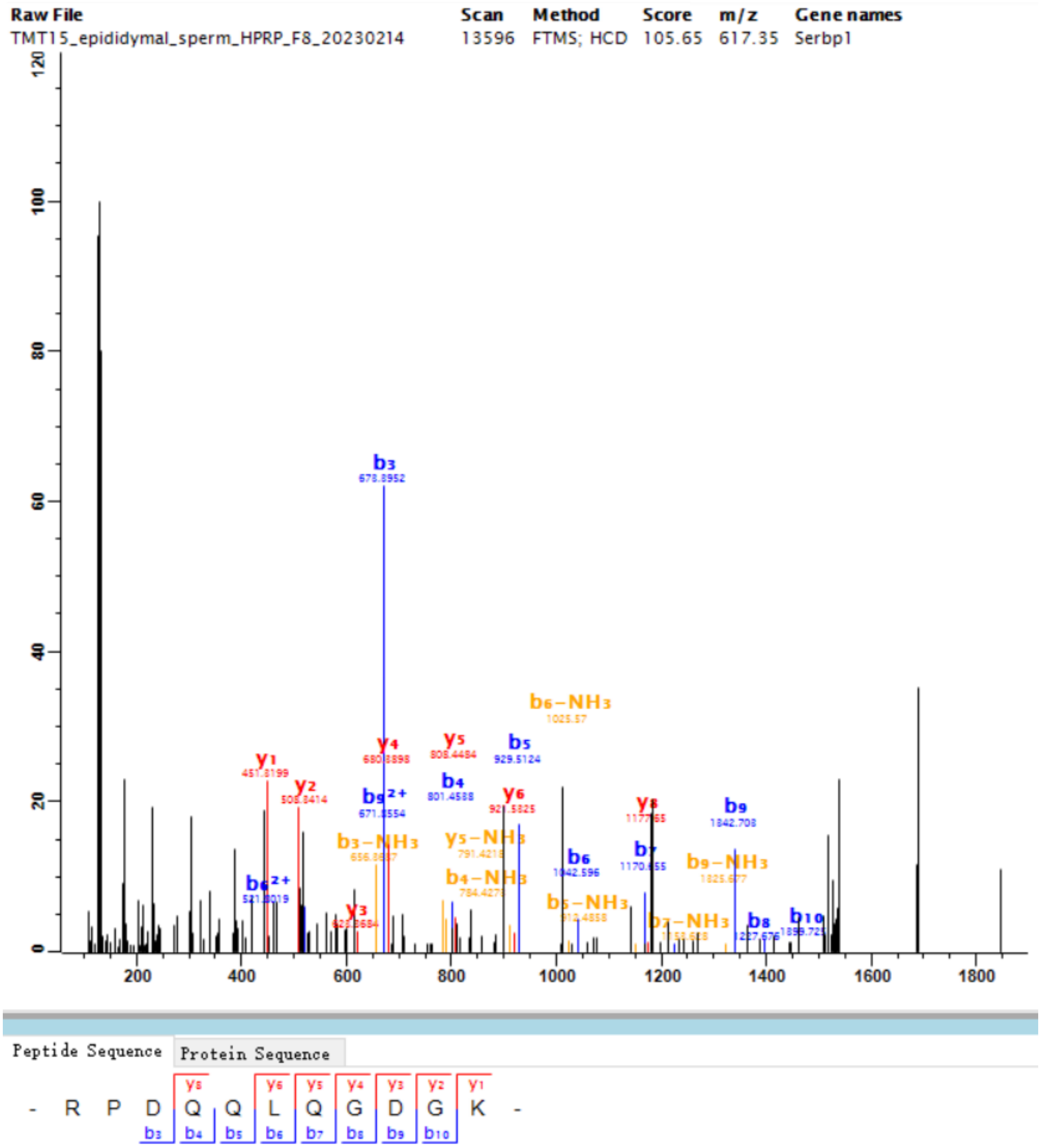
Raw File
TMT15_epididymal_sperm_HPRP_F8_20230214
Scan
13596
Method
FTMS; HCD
Score
105.65
m/z
617.35
Gene names
Serbp1
b3 678.8952
y1 451.8199
y2 508.8414
y4
y5 808.4494
b5 929.5124
b4 801.4588
b6-NH3 1025.57
y6
b3-NH3
y5-NH3 791.4218
b4-NH3
b6
b7
y8
b9 1842.708
b9-NH3
b5-NH3
b7-NH3
b8
b10
y3
Peptide Sequence
Protein Sequence
- R P D Q Q L Q G D G K -

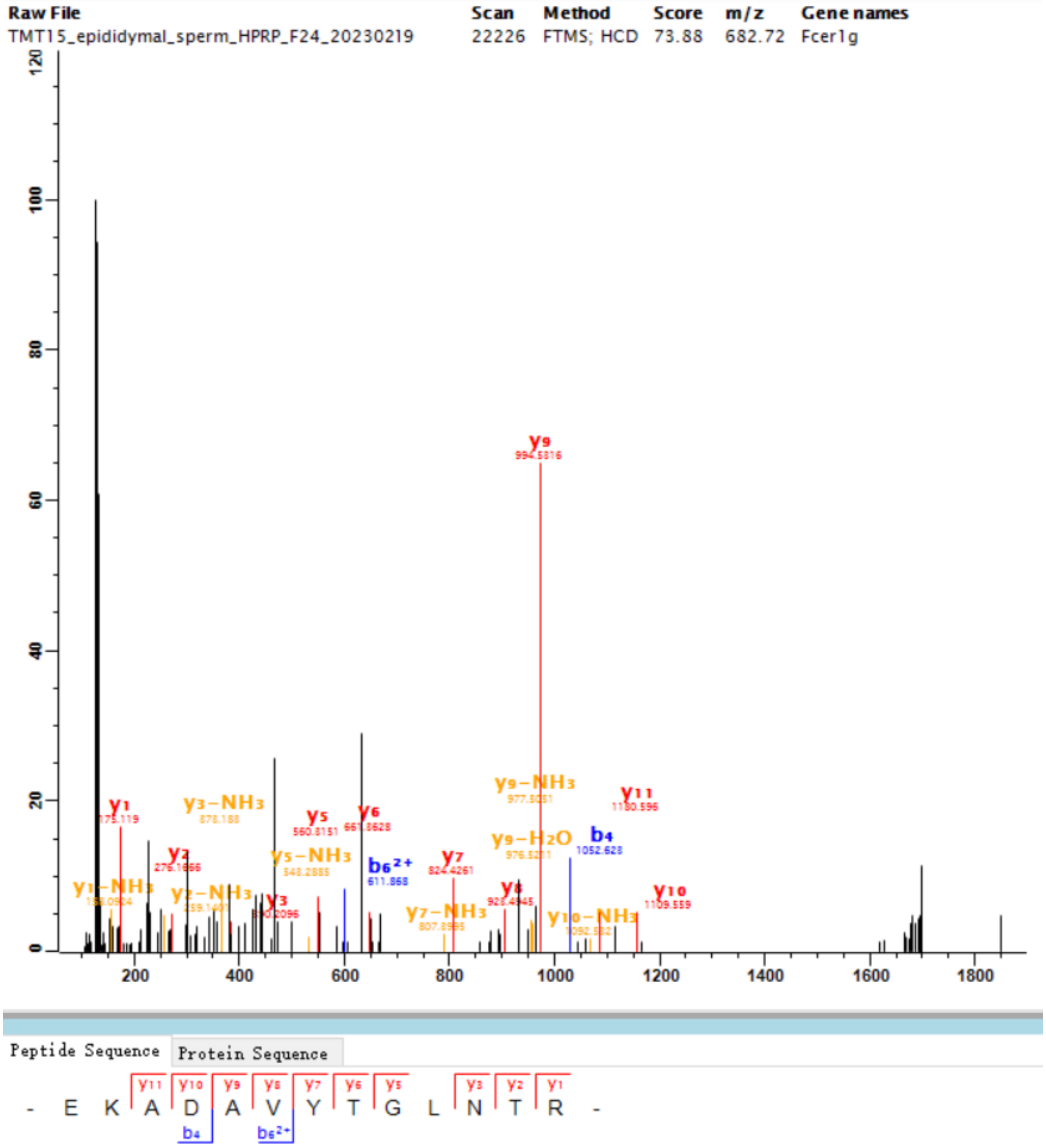
Raw File
TMT15_epididymal_sperm_HPRP_F24_20230219
Scan
22226
Method
FTMS; HCD
Score
73.88
m/z
682.72
Gene names
Fcer1g
y1
175.119
y2
276.1666
y3
y5
y6
y7
824.4261
y8
y9
994.5016
y10
1109.559
y11
b4
1052.628
b6 2+
611.858
y1-NH3
y2-NH3
y3-NH3
y5-NH3
y7-NH3
y9-NH3
y9-H2O
y10-NH3
Peptide Sequence
Protein Sequence
- E K A D A V Y T G L N T R -

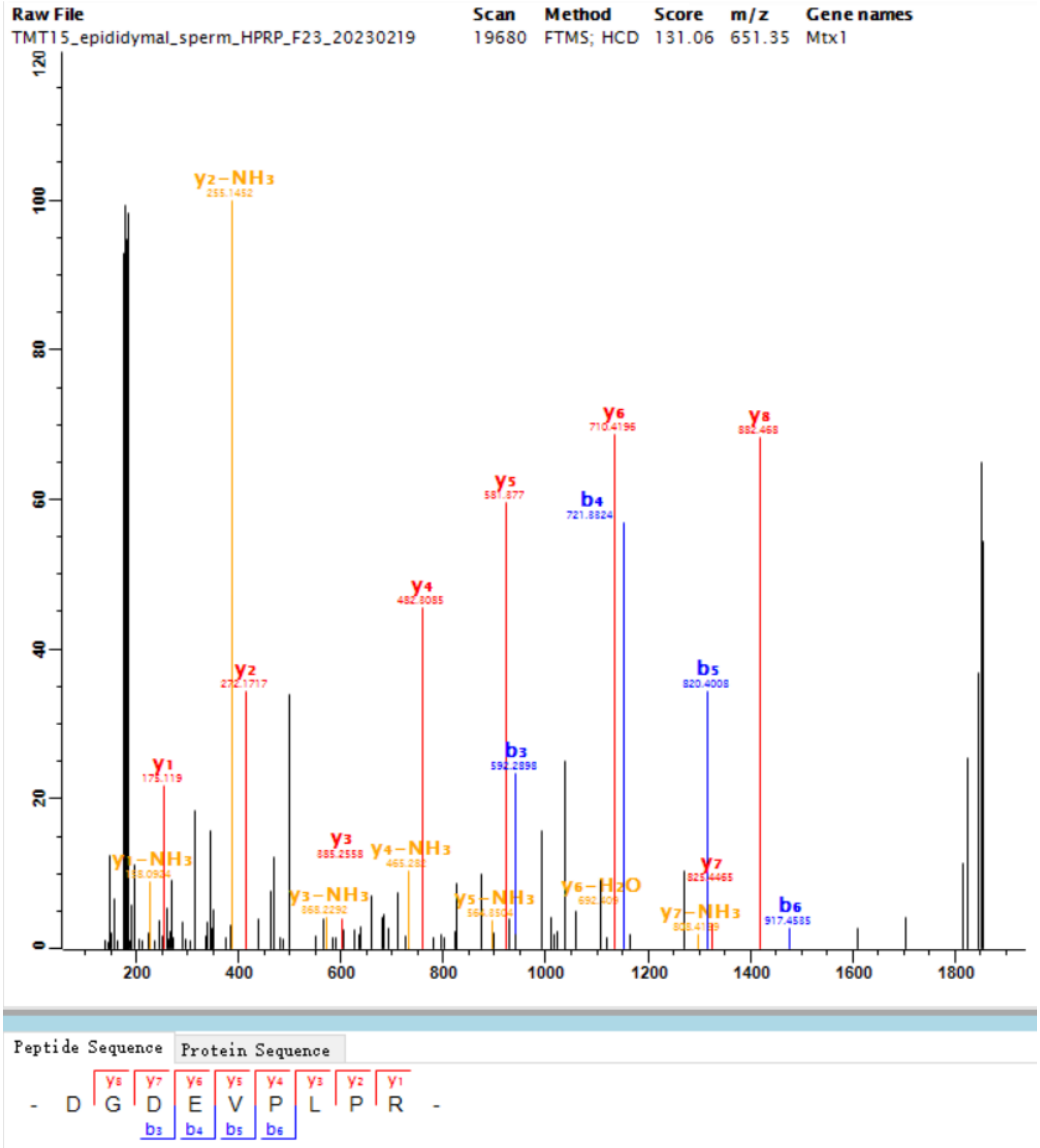

Raw File
TMT15_epididymal_sperm_HPRP_F23_20230219
Scan
19680
Method
FTMS; HCD
Score
131.06
m/z
651.35
Gene names
Mtx1
y2−NH3
255.1452
y6
710.4196
y8
882.468
y5
581.877
b4
721.8824
y4
482.8085
b5
820.4008
y2
272.1717
b3
592.2898
y1
175.119
y3
y4−NH3
y3−NH3
y5−NH3
y6−H2O
y7
y7−NH3
b6
917.4585
Peptide Sequence
Protein Sequence
- D G D E V P L P R -

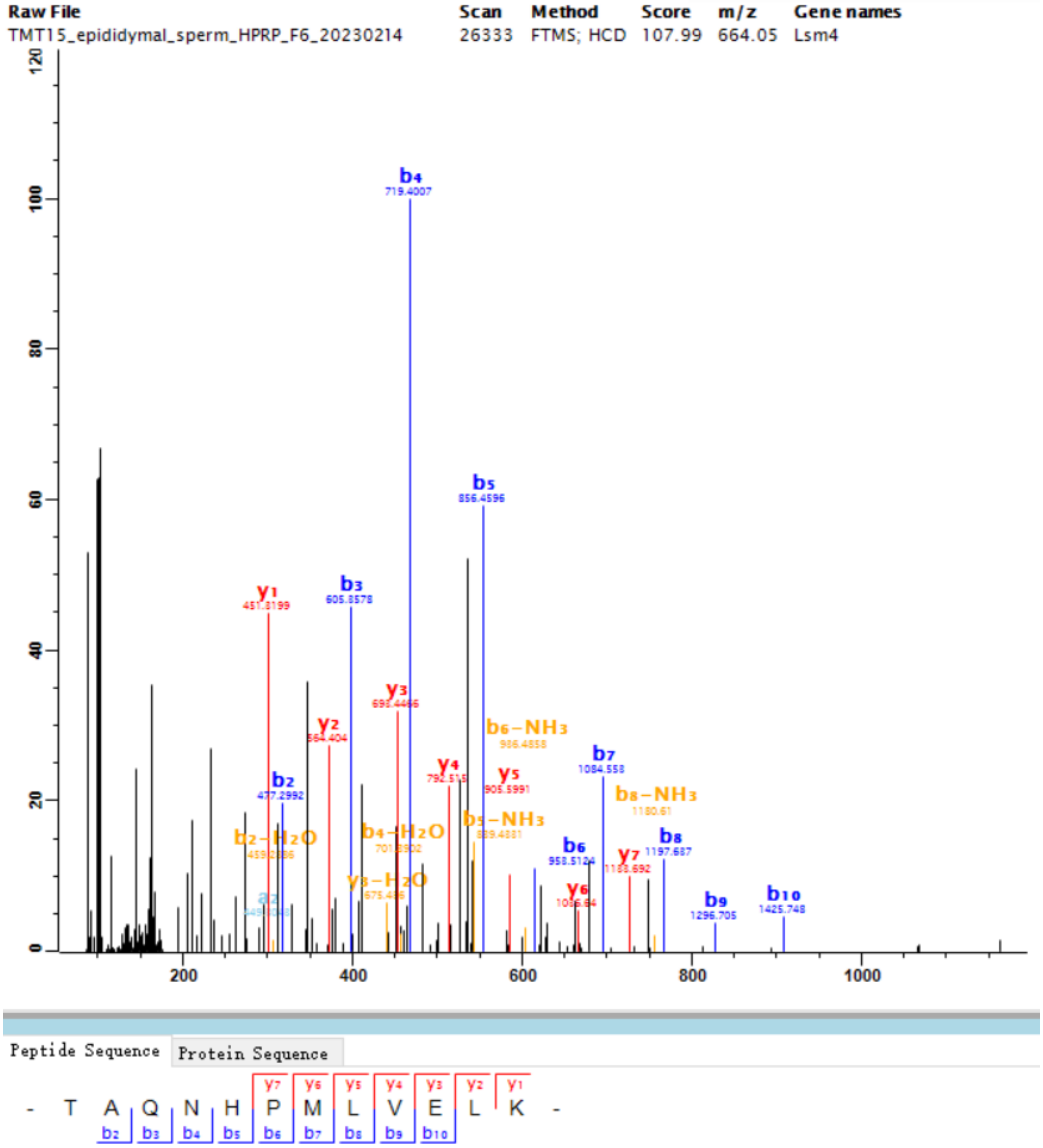
Raw File
TMT15_epididymal_sperm_HPRP_F6_20230214
Scan
26333
Method
FTMS; HCD
Score
107.99
m/z
664.05
Gene names
Lsm4
b4
719.4007
b5
856.4596
b3
605.3578
y1
451.3199
y3
y2
b6−NH3
b7
1084.558
b2
477.2992
y4
y5
905.5991
b8−NH3
b4−H2O
b8
1197.687
b6
958.5124
y7
b9
1296.705
b10
1425.748
200
400
600
800
1000
0
20
40
60
80
100
120
Peptide Sequence
Protein Sequence
- T A Q N H P M L V E L K -

Raw File	Scan	Method	Score	m/z	Gene names
TMT15_epididymal_sperm_HPRP_F16_20230218	28836	FTMS; HCD	243.52	1058.49	Zp3r

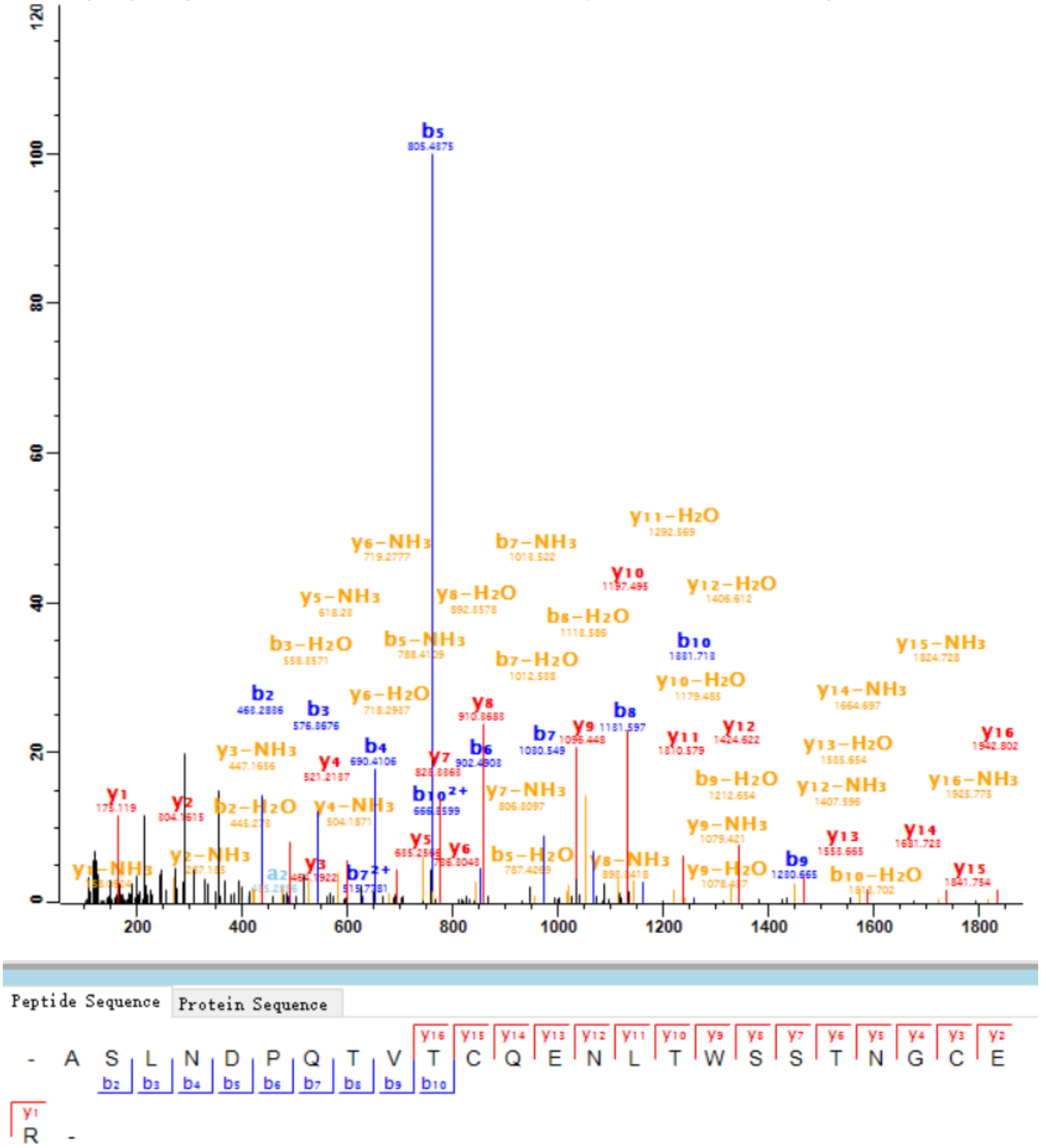

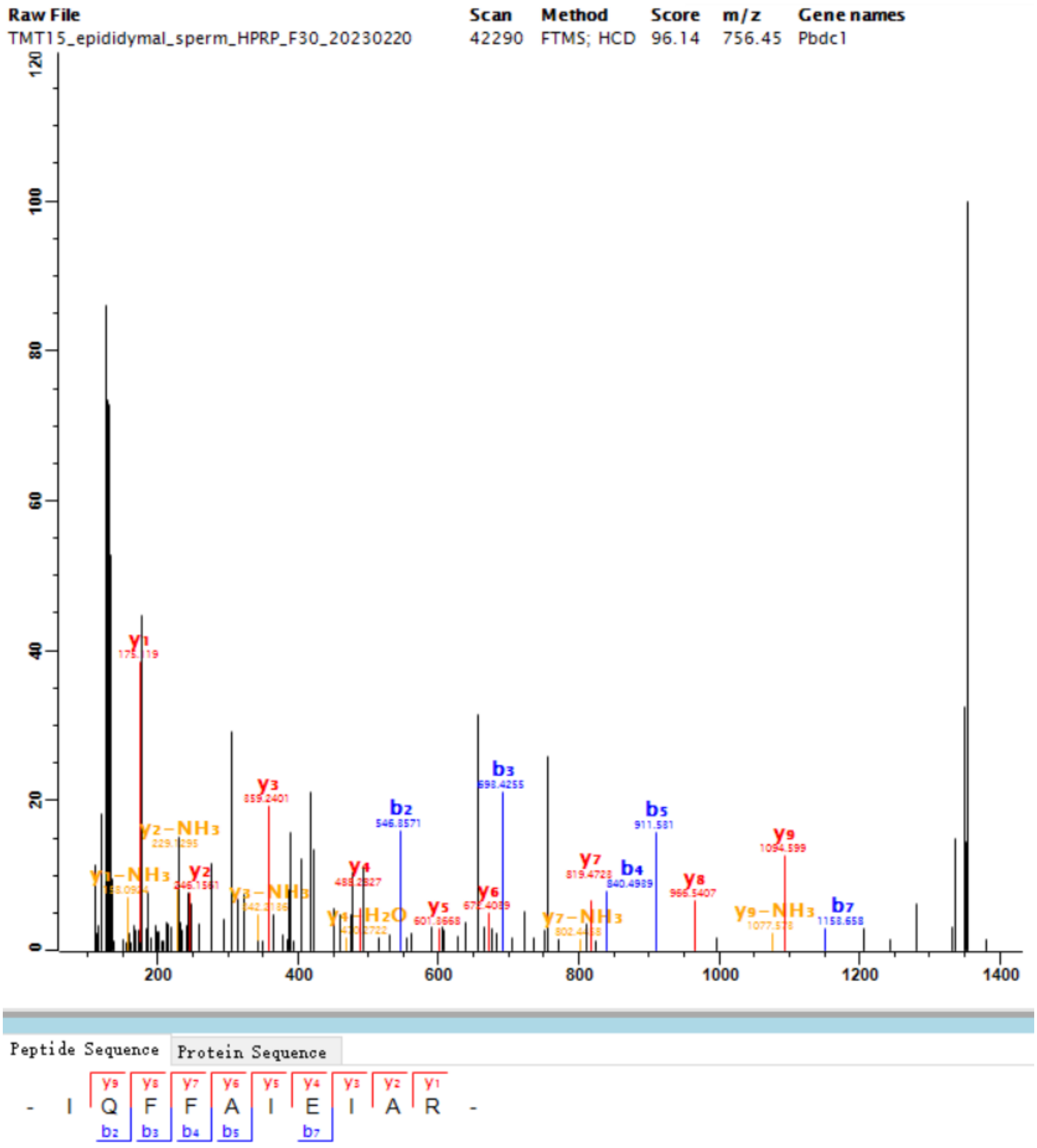
Raw File
TMT15_epididymal_sperm_HPRP_F30_20230220
Scan
42290
Method
FTMS; HCD
Score
96.14
m/z
756.45
Gene names
Pbdc1
b2
546.8571
b3
b4
840.4989
b5
911.581
b7
1158.658
y9
1094.599
y8
966.5407
Peptide Sequence
Protein Sequence
- I Q F F A I E I A R -

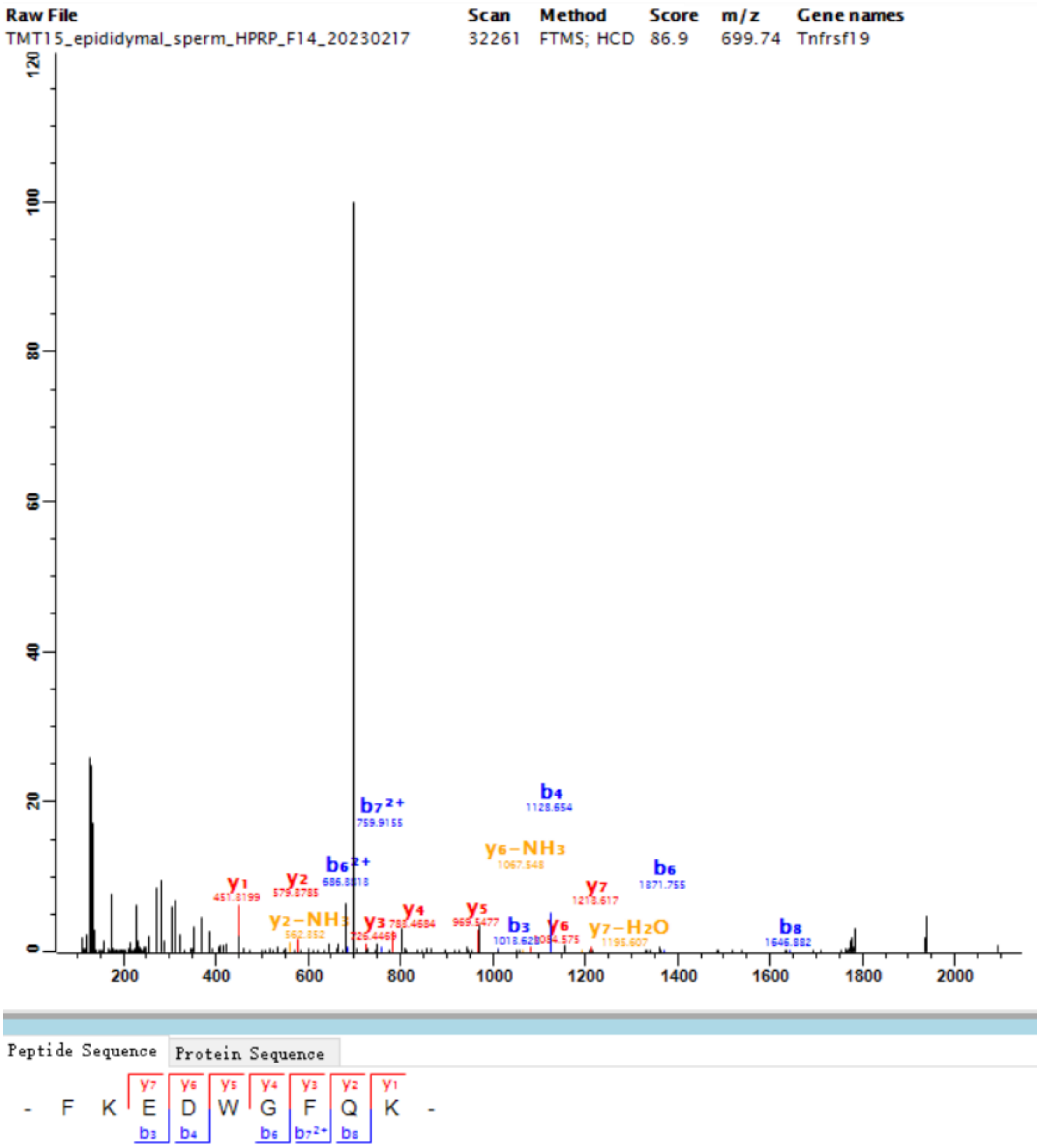
Raw File
TMT15_epididymal_sperm_HPRP_F14_20230217
Scan
32261
Method
FTMS; HCD
Score
86.9
m/z
699.74
Gene names
Tnfrsf19
y1
y2
y2-NH3
y3
y4
y5
y6
y6-NH3
y7
y7-H2O
b3
b4
b6
b6 2+
b7 2+
b8
759.9155
1128.654
1067.548
1371.755
1646.882
Peptide Sequence
Protein Sequence
- F K E D W G F Q K -

Raw File	Scan	Method	Score	m/z	Gene names
TMT15_epididymal_sperm_HPRP_F21_20230219	13784	FTMS; HCD	156.08	551.81	mt-Co3

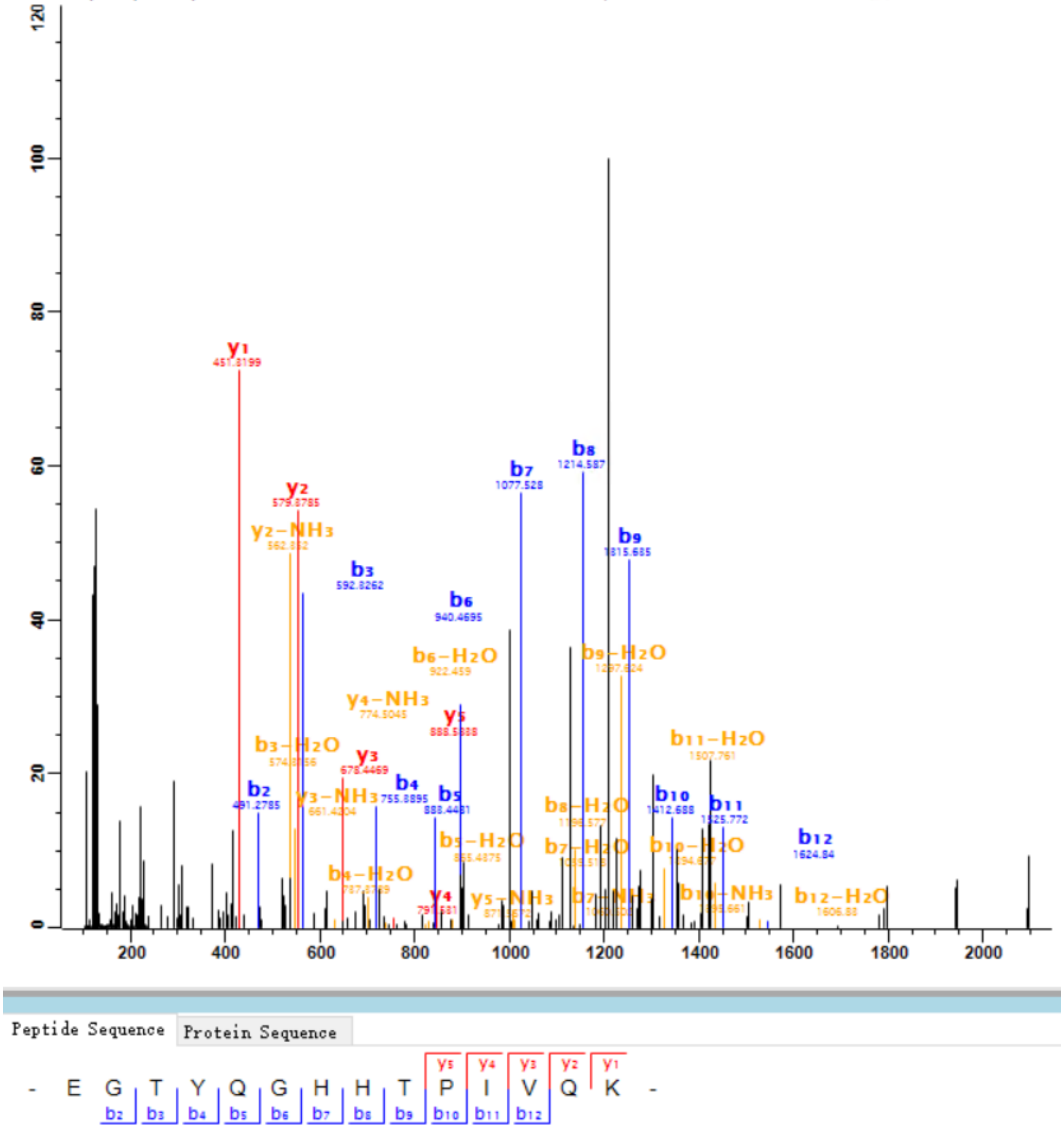

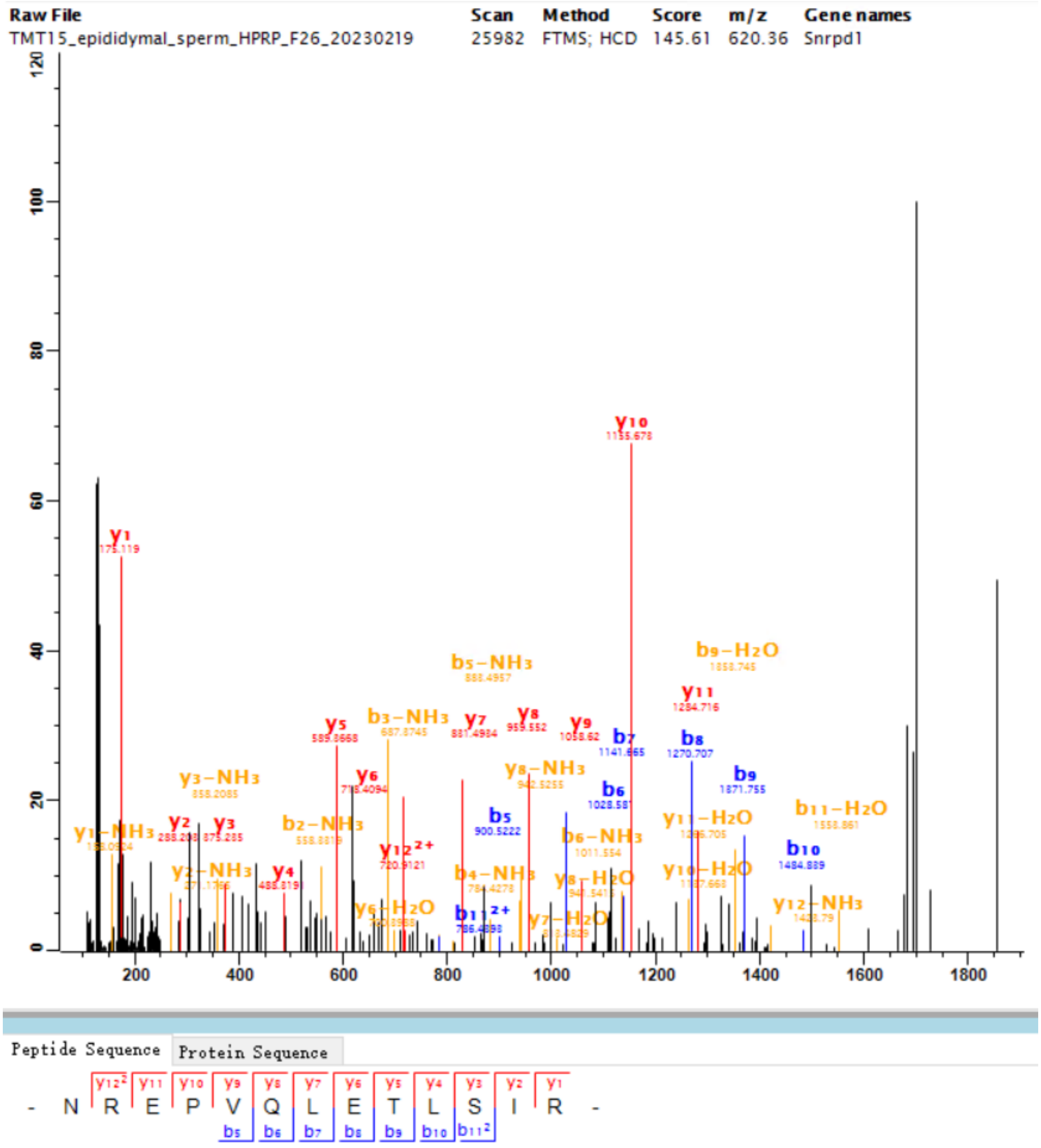
Raw File
TMT15_epididymal_sperm_HPRP_F26_20230219
Scan
25982
Method
FTMS; HCD
Score
145.61
m/z
620.36
Gene names
Snrpd1
Peptide Sequence
Protein Sequence
- N R E P V Q L E T L S I R -

Raw File	Scan	Method	Score	m/z	Gene names
TMT15_epididymal_sperm_HPRP_F27_20230219	30353	FTMS; HCD	83.25	763.78	Supt4h1b;Supt4h1a

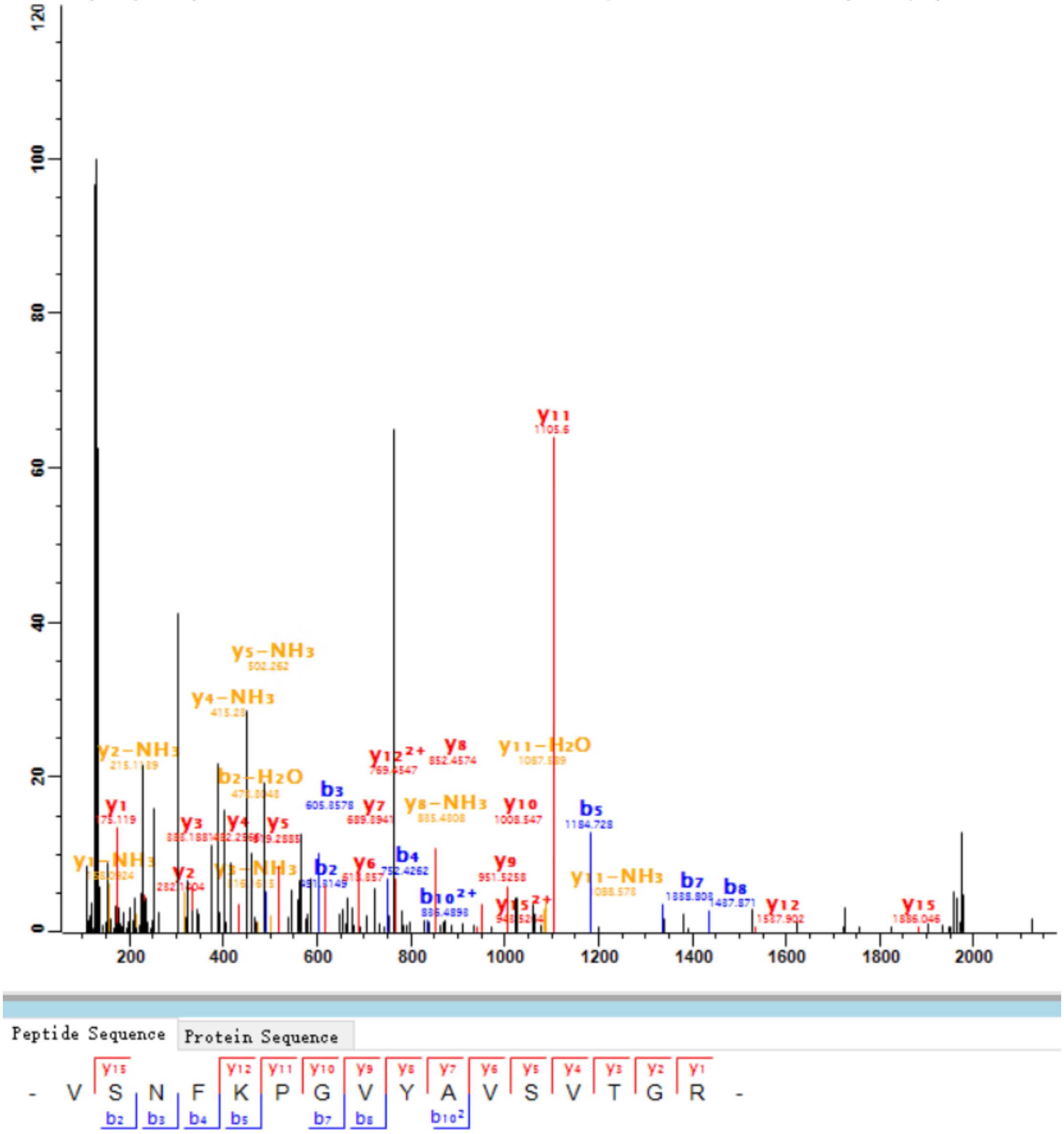

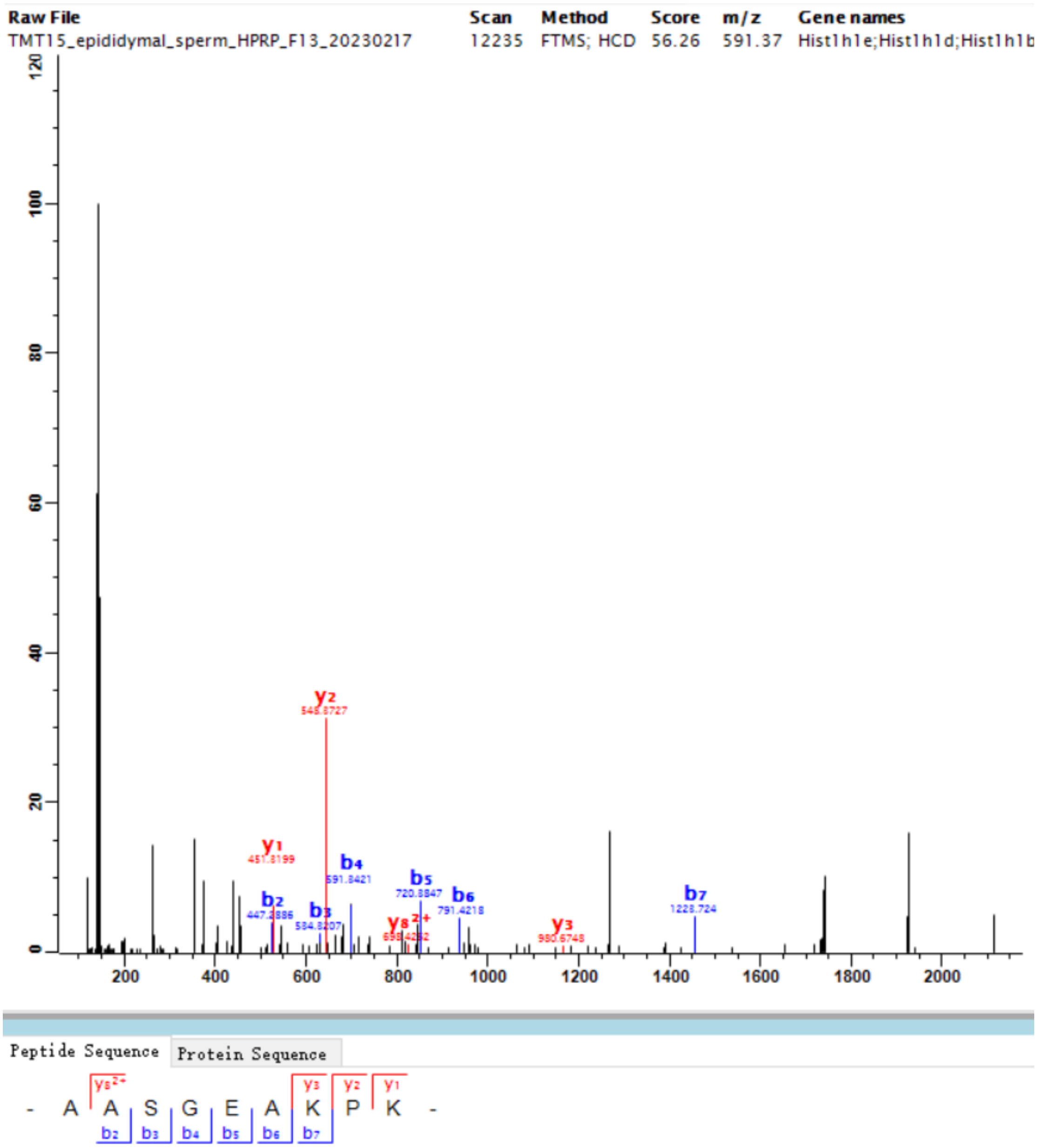
Raw File
TMT15_epididymal_sperm_HPRP_F13_20230217
Scan 12235
Method FTMS; HCD
Score 56.26
m/z 591.37
Gene names Hist1h1e;Hist1h1d;Hist1h1b
y2
y1
b2
b3
b4
b5
b6
y3
b7
Peptide Sequence
Protein Sequence
- A A S G E A K P K -

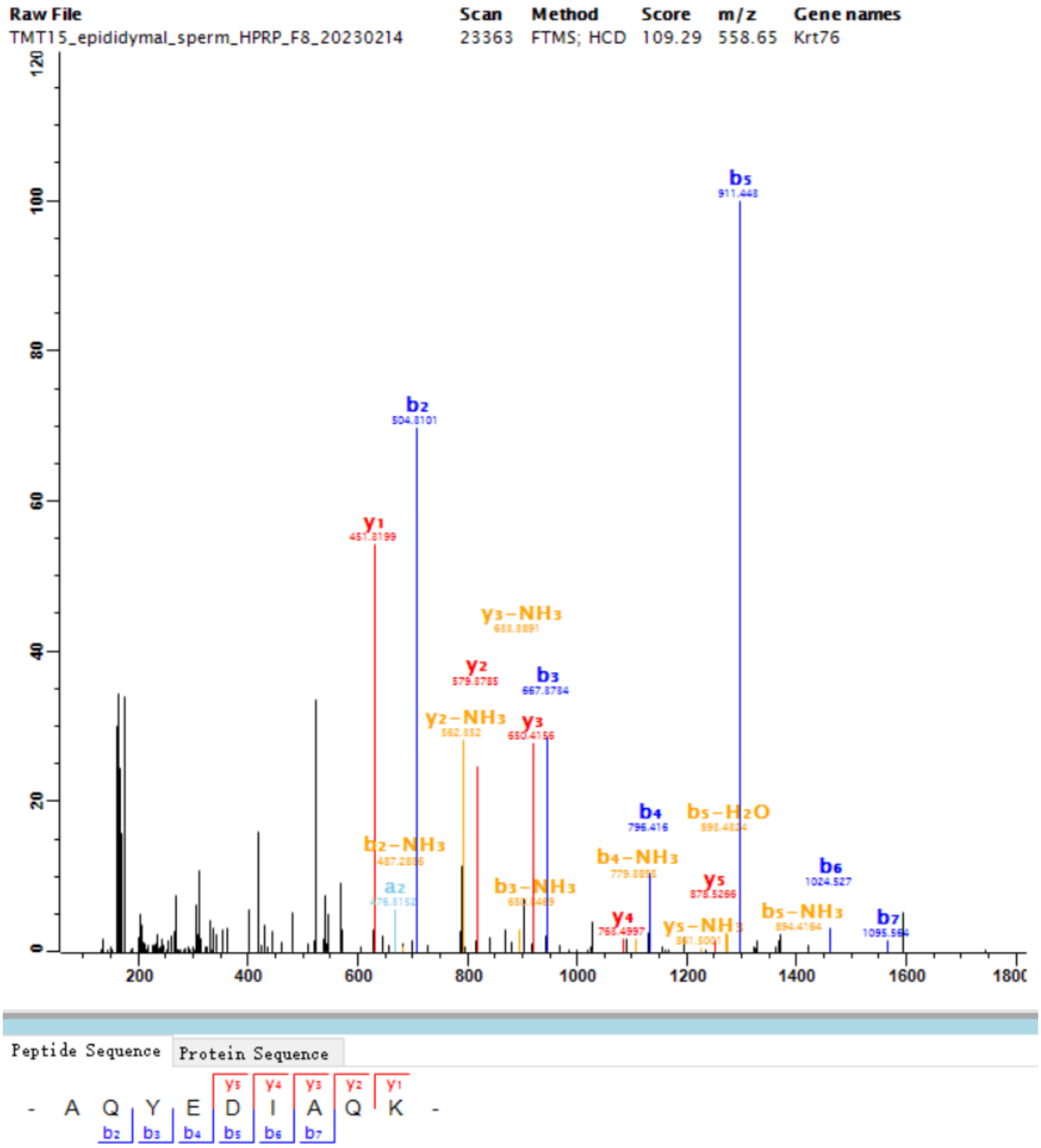

Raw File
TMT15_epididymal_sperm_HPRP_F8_20230214
Scan
23363
Method
FTMS; HCD
Score
109.29
m/z
558.65
Gene names
Krt76
b5
911.448
b2
504.8101
y1
y3-NH3
688.8891
y2
579.8785
b3
667.8784
y2-NH3
562.852
y3
b4
796.416
b5-H2O
b2-NH3
487.2886
a2
b4-NH3
779.8895
b3-NH3
y5
878.5266
b6
1024.527
y4
y5-NH3
b5-NH3
894.4164
b7
1095.564
0
20
40
60
80
100
120
200
400
600
800
1000
1200
1400
1600
Peptide Sequence
Protein Sequence
- A Q Y E D I A Q K -
y5 y4 y3 y2 y1
b2 b3 b4 b5 b6 b7

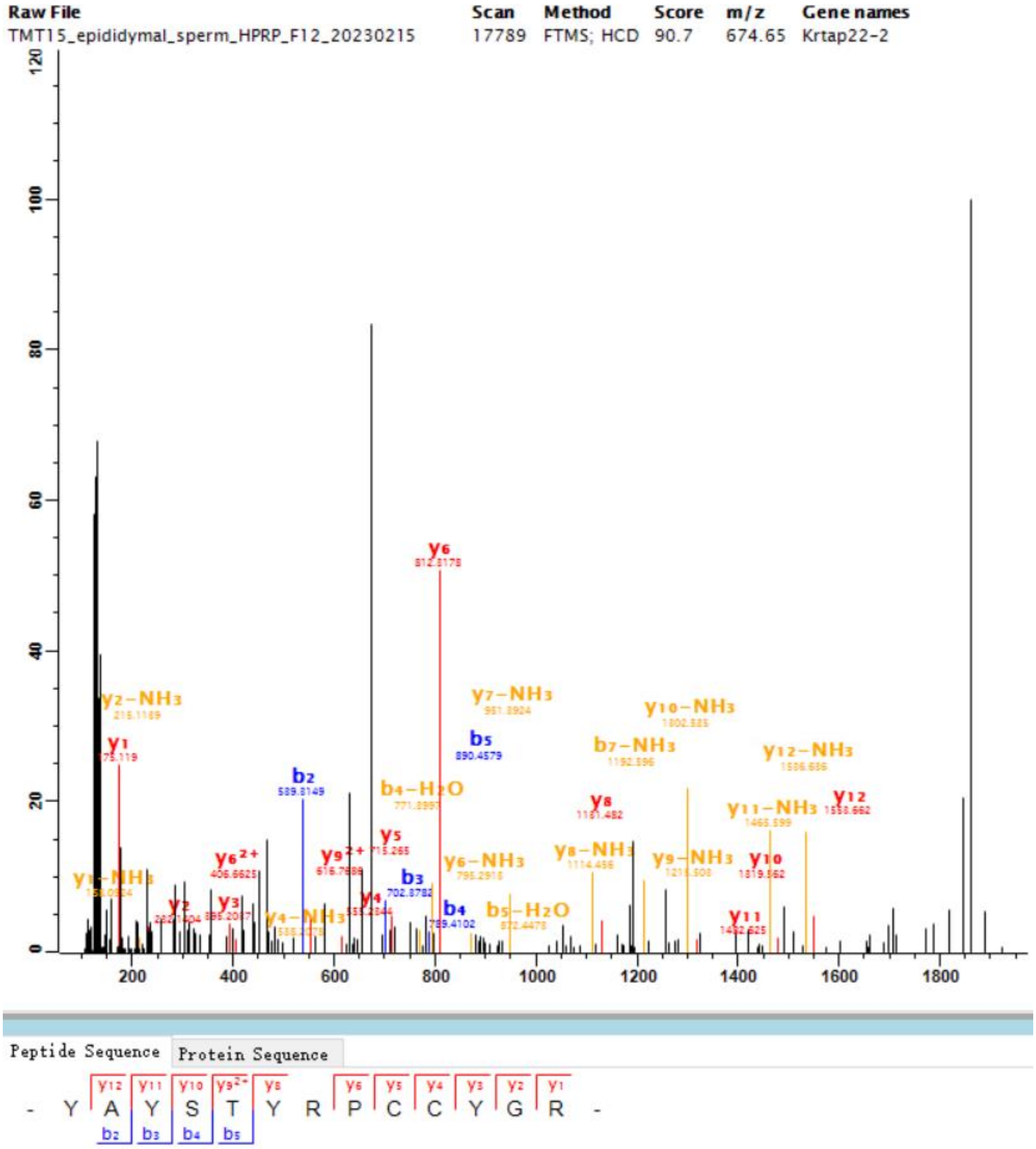
Raw File
TMT15_epididymal_sperm_HPRP_F12_20230215
Scan
17789
Method
FTMS; HCD
Score
90.7
m/z
674.65
Gene names
Krtap22-2
Peptide Sequence
Protein Sequence
- Y A Y S T Y R P C C Y G R -

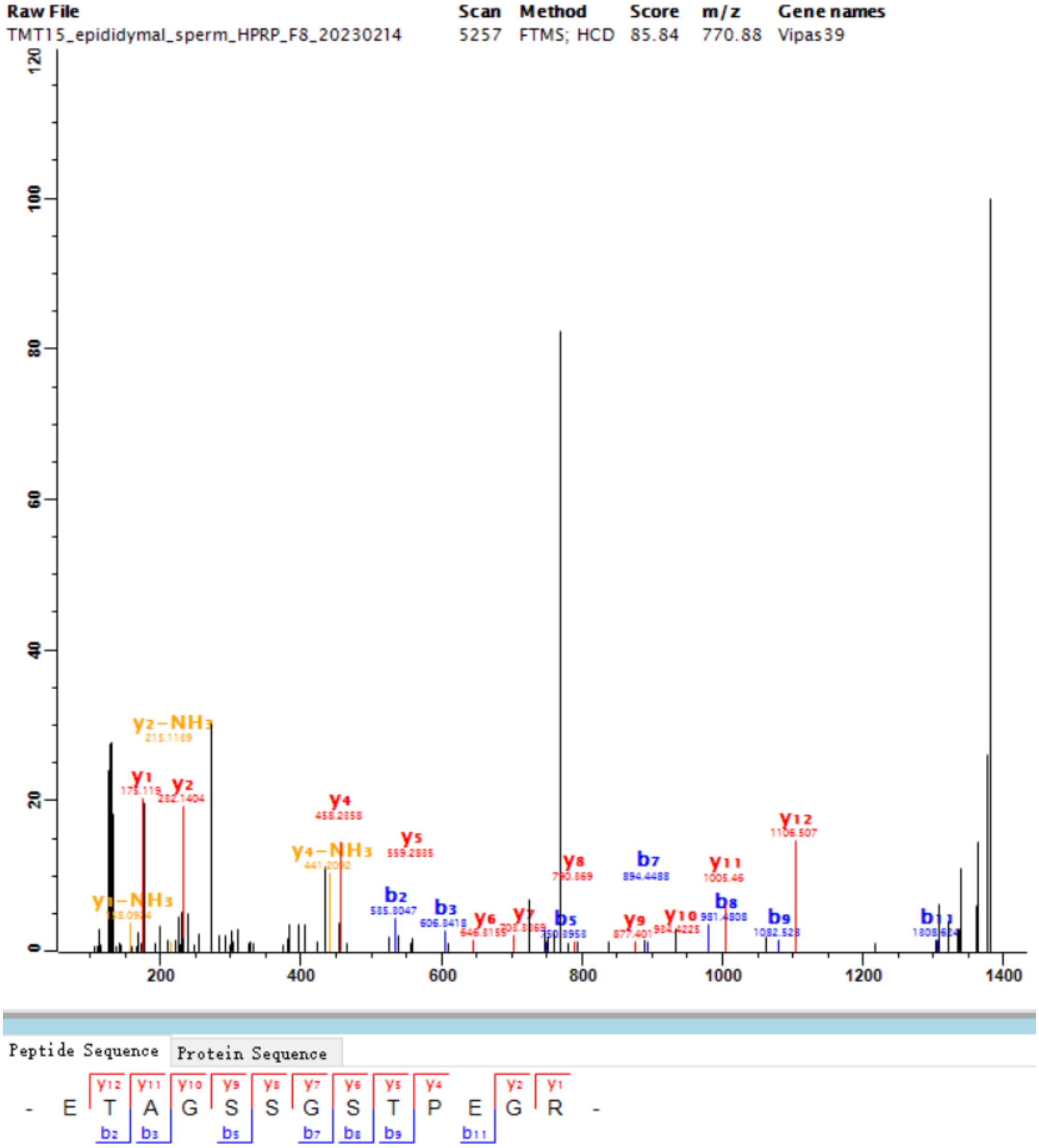
Raw File
TMT15_epididymal_sperm_HPRP_F8_20230214
Scan
5257
Method
FTMS; HCD
Score
85.84
m/z
770.88
Gene names
Vipas39
y2-NH3
215.1189
y1
175.119
y2
232.1404
y4
458.2858
y4-NH3
441.2092
y5
559.2885
b2
535.8047
b3
606.8418
y6
646.8155
y7
y8
790.869
b5
b7
894.4488
y9
877.401
y10
934.4225
y11
1005.46
b8
981.4808
b9
1082.528
y12
1106.507
b11
Peptide Sequence
Protein Sequence
- E T A G S S G S T P E G R -

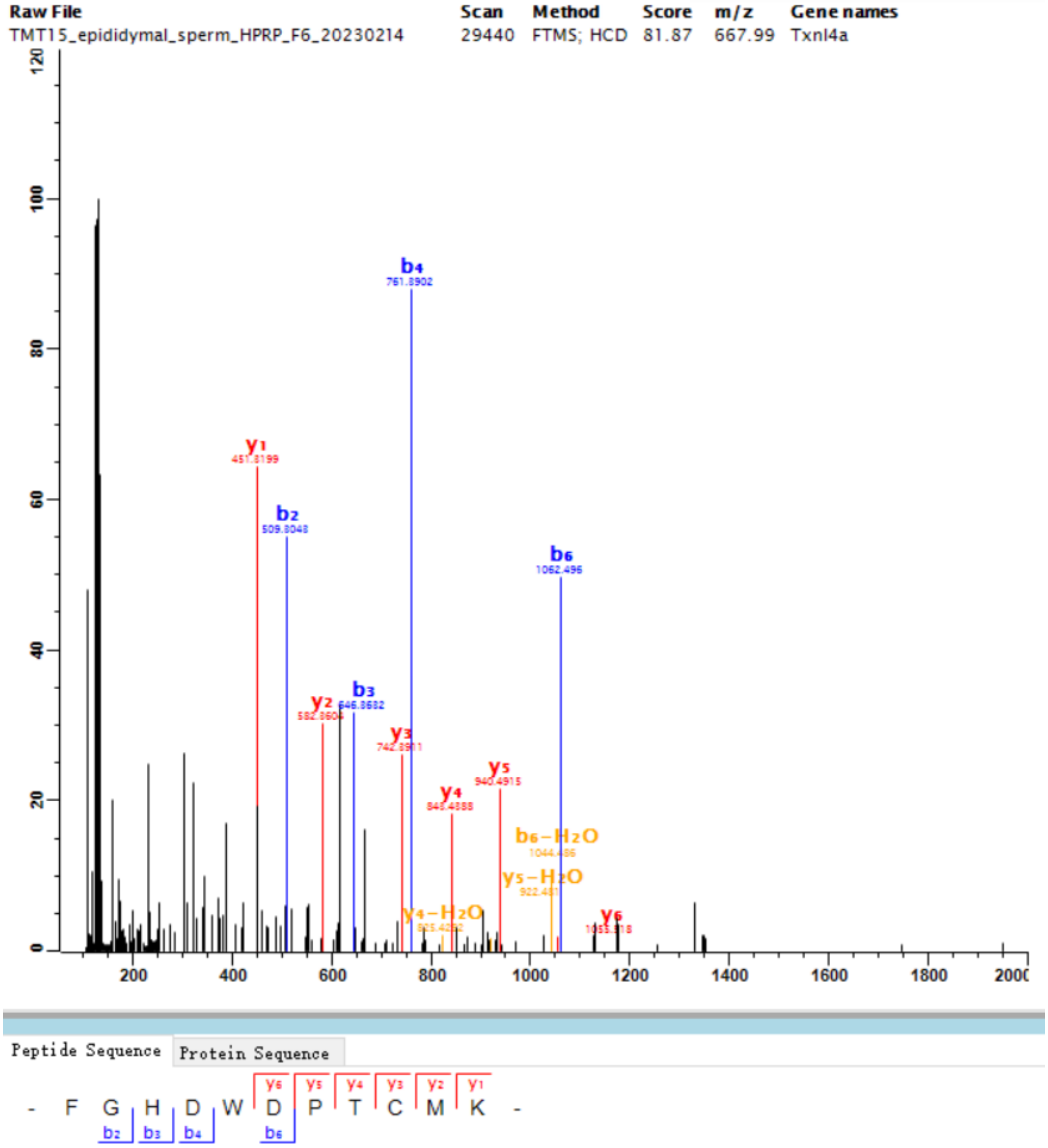

Raw File
TMT15_epididymal_sperm_HPRP_F6_20230214
Scan
29440
Method
FTMS; HCD
Score
81.87
m/z
667.99
Gene names
Txnl4a
b4
761.8902
y1
451.8199
b2
509.8048
b6
1062.496
y2
b3
646.8682
y3
y5
940.4915
y4
b6-H2O
1044.486
y5-H2O
y4-H2O
y6
Peptide Sequence
Protein Sequence
- F G H D W D P T C M K -

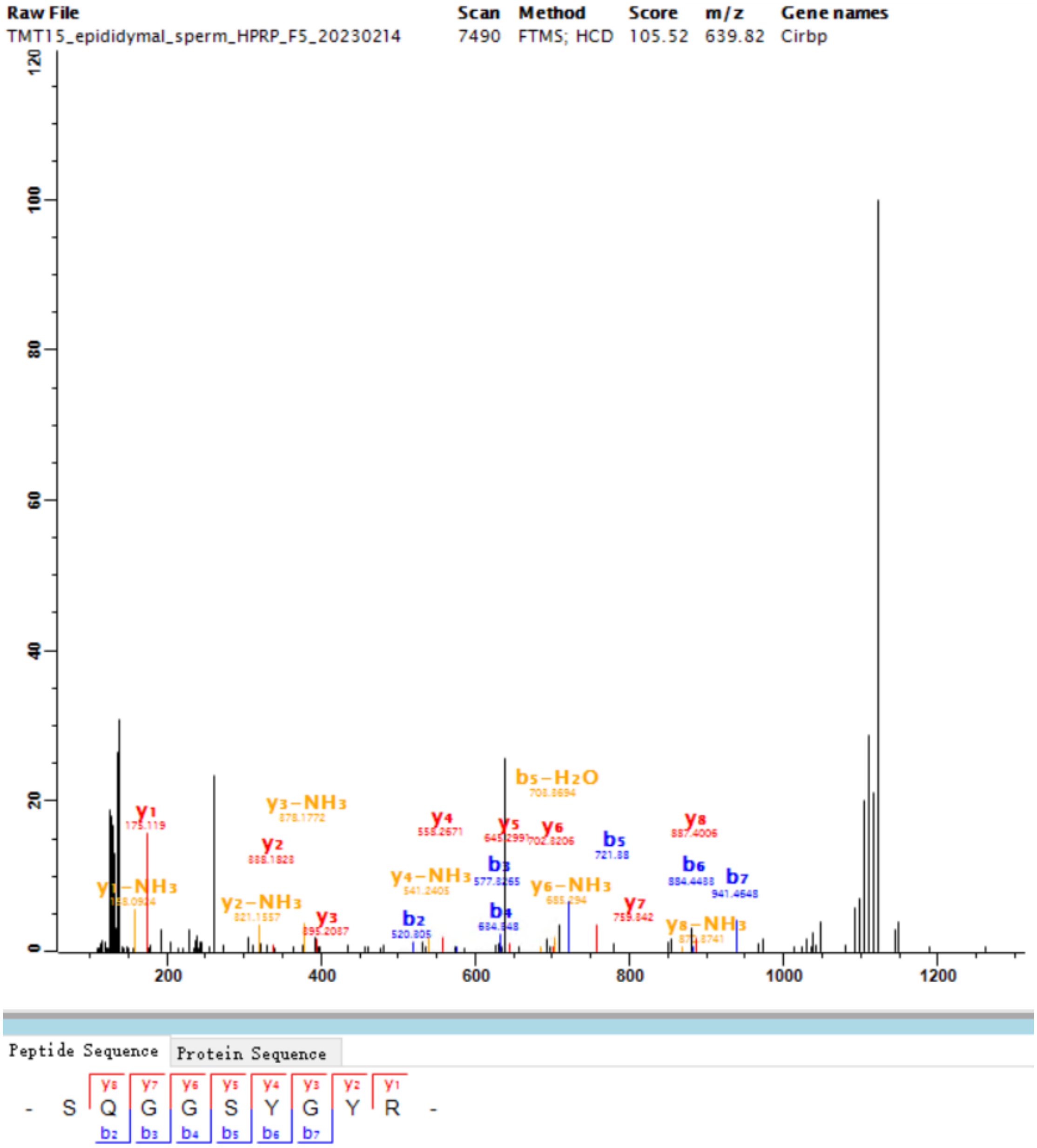
Raw File
TMT15_epididymal_sperm_HPRP_F5_20230214
Scan 7490
Method FTMS; HCD
Score 105.52
m/z 639.82
Gene names Cirbp
Peptide Sequence
Protein Sequence
- S Q G G S Y G Y R -
y1 y2 y3 y4 y5 y6 y7 y8
b2 b3 b4 b5 b6 b7
y1-NH3 y2-NH3 y3-NH3 y4-NH3 y6-NH3 y8-NH3
b5-H2O

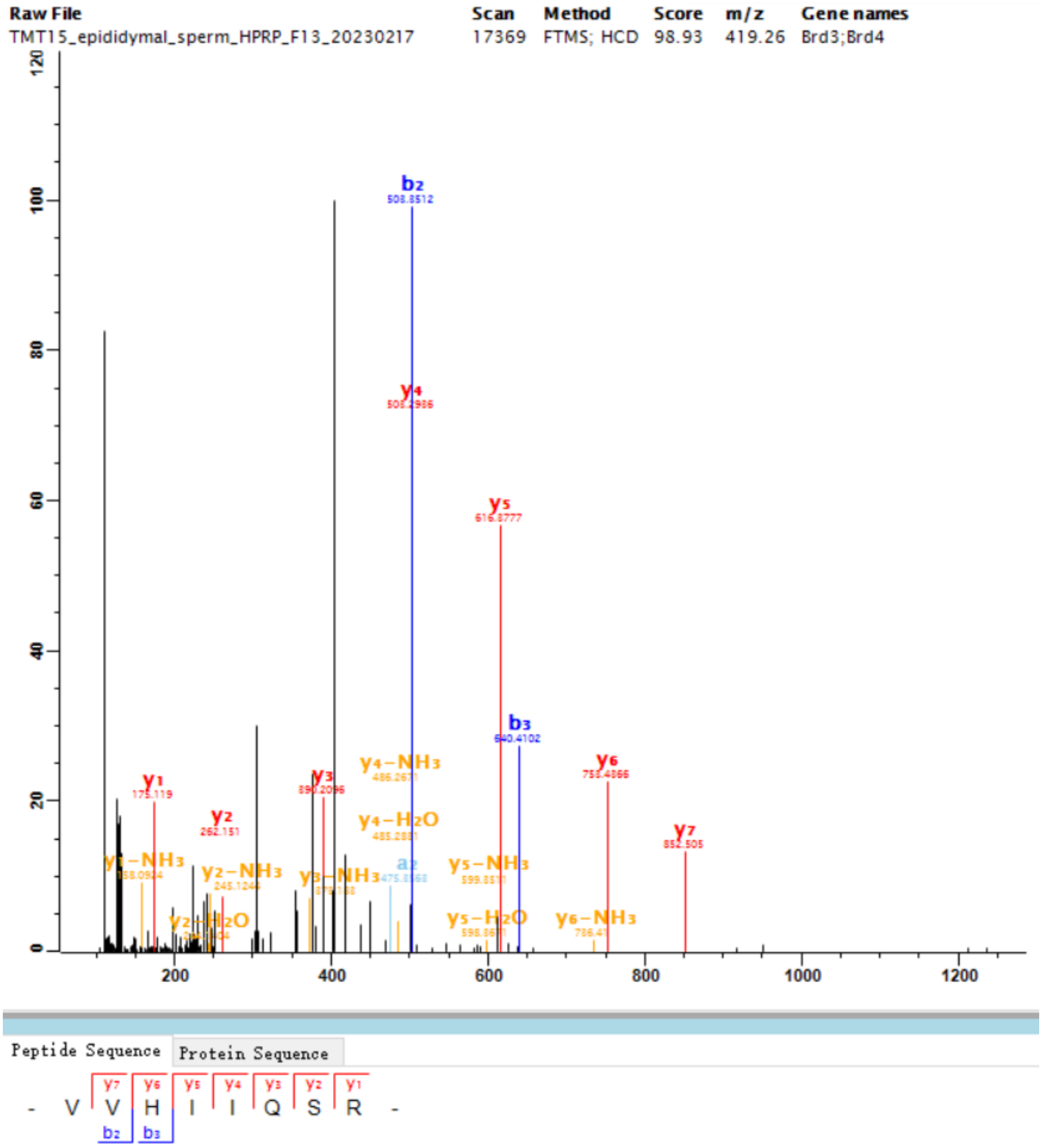

Raw File
TMT15_epididymal_sperm_HPRP_F13_20230217
Scan
17369
Method
FTMS; HCD
Score
98.93
m/z
419.26
Gene names
Brd3;Brd4
b2
y4
y5
616.8777
b3
640.4102
y6
y1
y2
262.151
y3
y4−NH3
y4−H2O
y7
852.505
y2−NH3
y5−NH3
y5−H2O
y6−NH3
0
20
40
60
80
100
120
200
400
600
800
1000
1200
Peptide Sequence
Protein Sequence
- V V H I I Q S R -

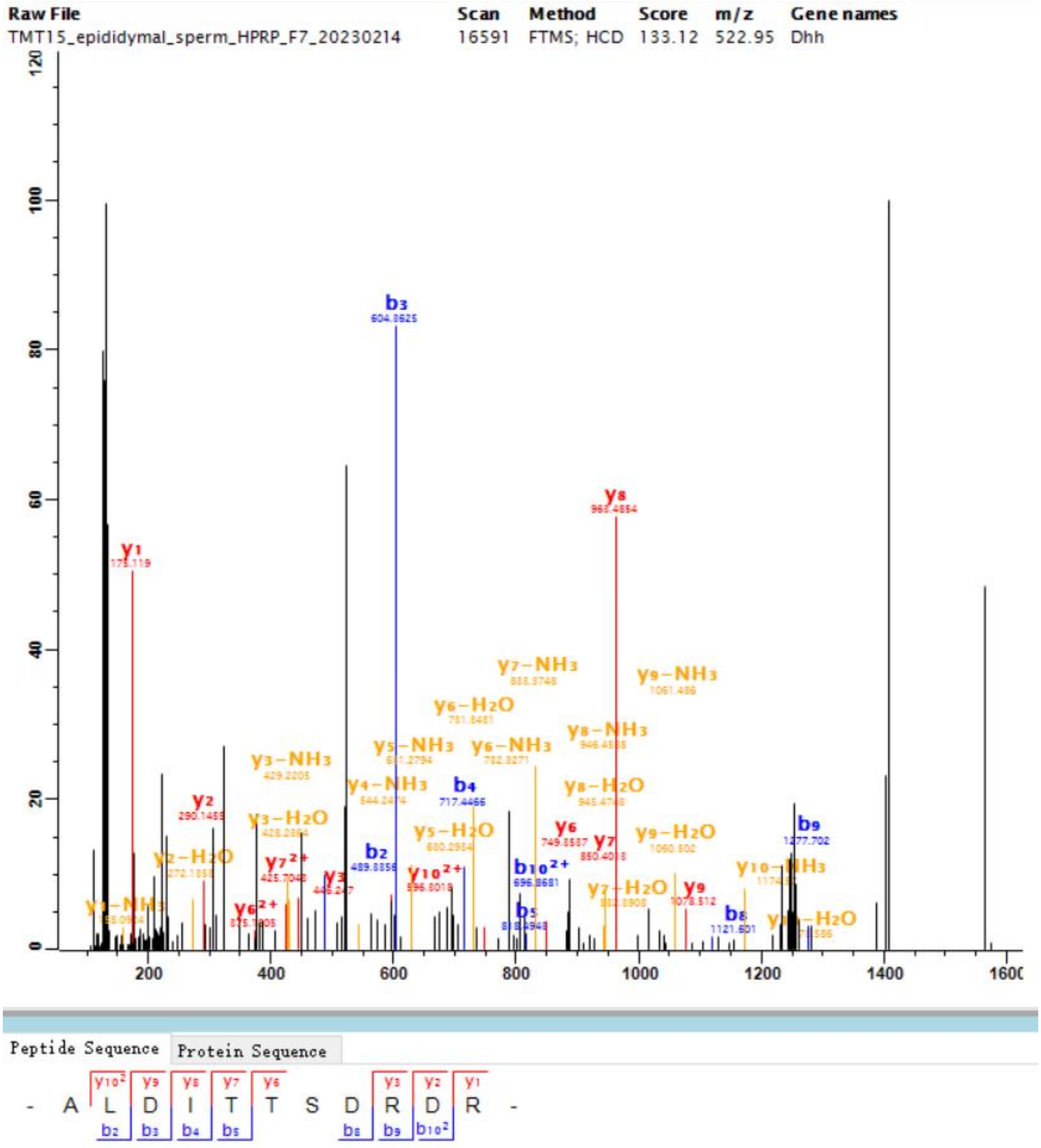

Raw File
TMT15_epididymal_sperm_HPRP_F7_20230214
Scan
16591
Method
FTMS; HCD
Score
133.12
m/z
522.95
Gene names
Dhh
b3
604.8625
y8
y1
y2
b2
b4
717.4466
b9
y6
y7
y9
y7−NH3
y9−NH3
y6−H2O
y8−NH3
y3−NH3
y5−NH3
y6−NH3
y4−NH3
y8−H2O
y3−H2O
y5−H2O
y9−H2O
y2−H2O
y10−NH3
y7−H2O
Peptide Sequence
Protein Sequence
- A L D I T T S D R D R -

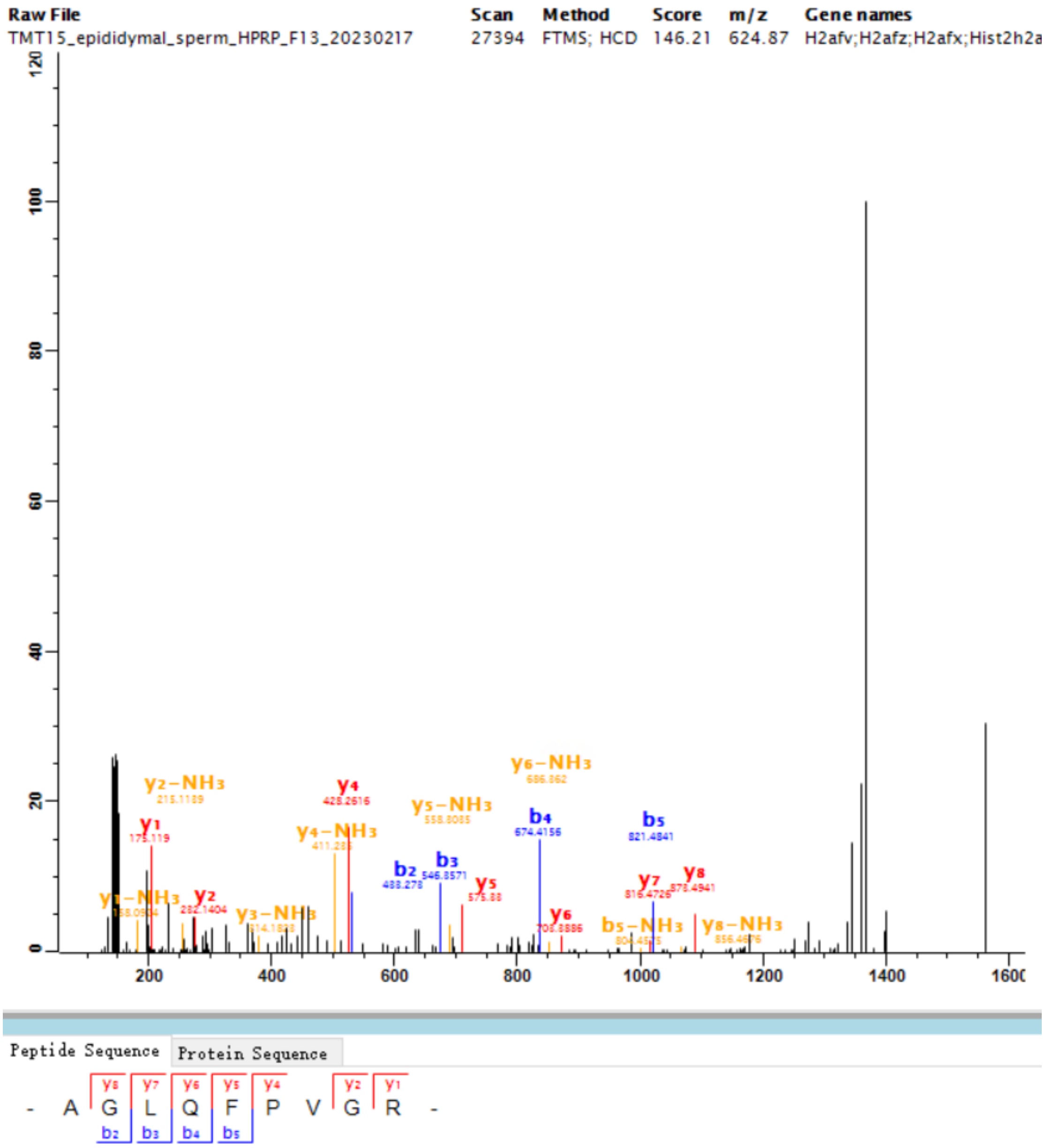

Raw File
TMT15_epididymal_sperm_HPRP_F13_20230217
Scan
27394
Method
FTMS; HCD
Score
146.21
m/z
624.87
Gene names
H2afv;H2afz;H2afx;Hist2h2a
y2-NH3
215.1189
y1
175.119
y4
428.2616
y4-NH3
y5-NH3
558.8085
y6-NH3
b4
674.4156
b5
821.4841
b2
488.278
b3
546.3571
y5
y7
y8
878.4941
y6
y2
282.1404
y3-NH3
b5-NH3
y8-NH3
Peptide Sequence
Protein Sequence
- A G L Q F P V G R -

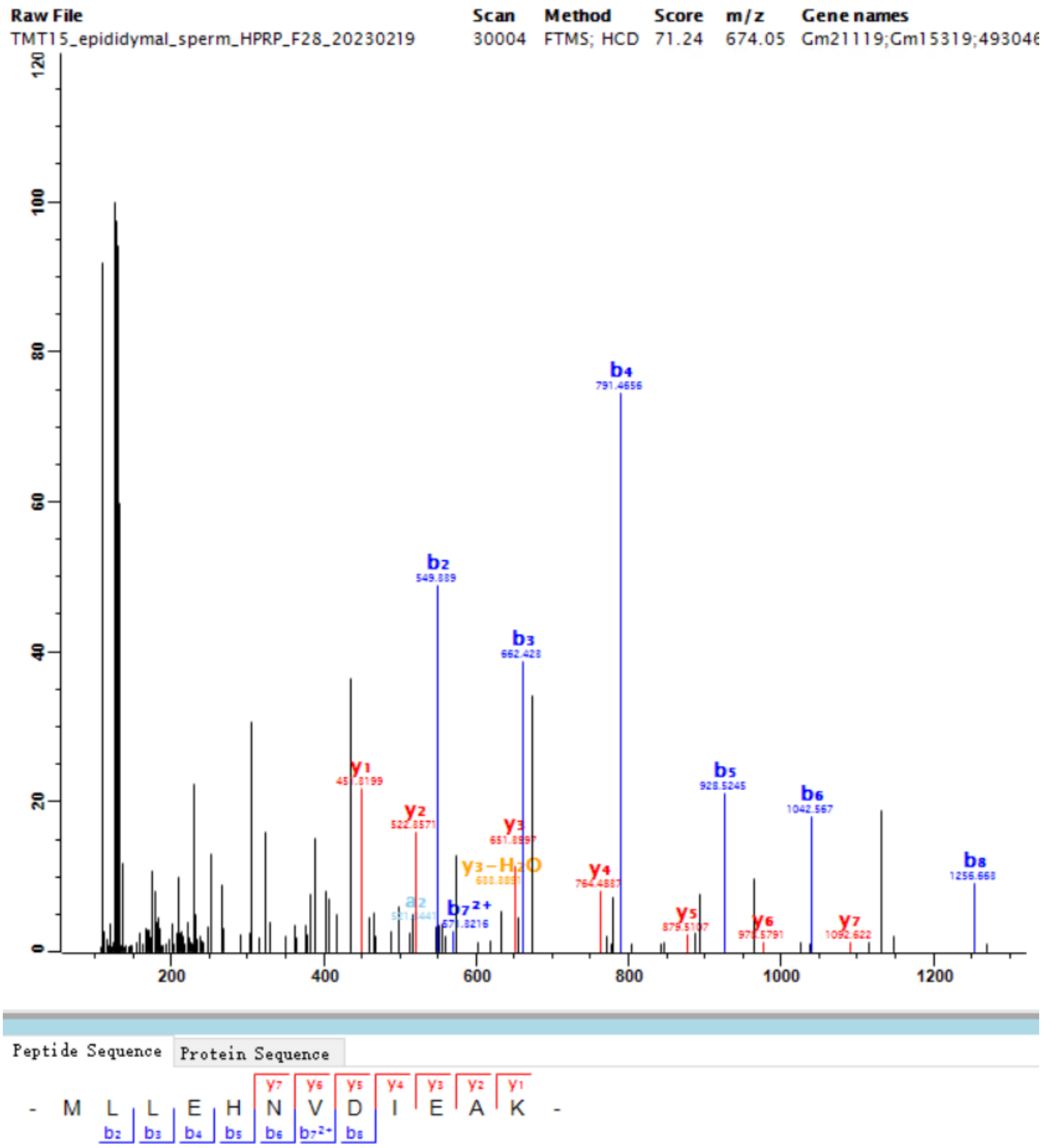

Raw File
TMT15_epididymal_sperm_HPRP_F28_20230219
Scan
30004
Method
FTMS; HCD
Score
71.24
m/z
674.05
Gene names
Gm21119;Gm15319;49304
b4
791.4656
b2
549.889
b3
662.428
b5
928.5245
b6
1042.567
b8
1256.668
y1
y2
522.8571
y3
y4
764.4887
y5
y6
y7
1092.622
y3-H2O
a2
b7 2+
Peptide Sequence
Protein Sequence
- M L L E H N V D I E A K -

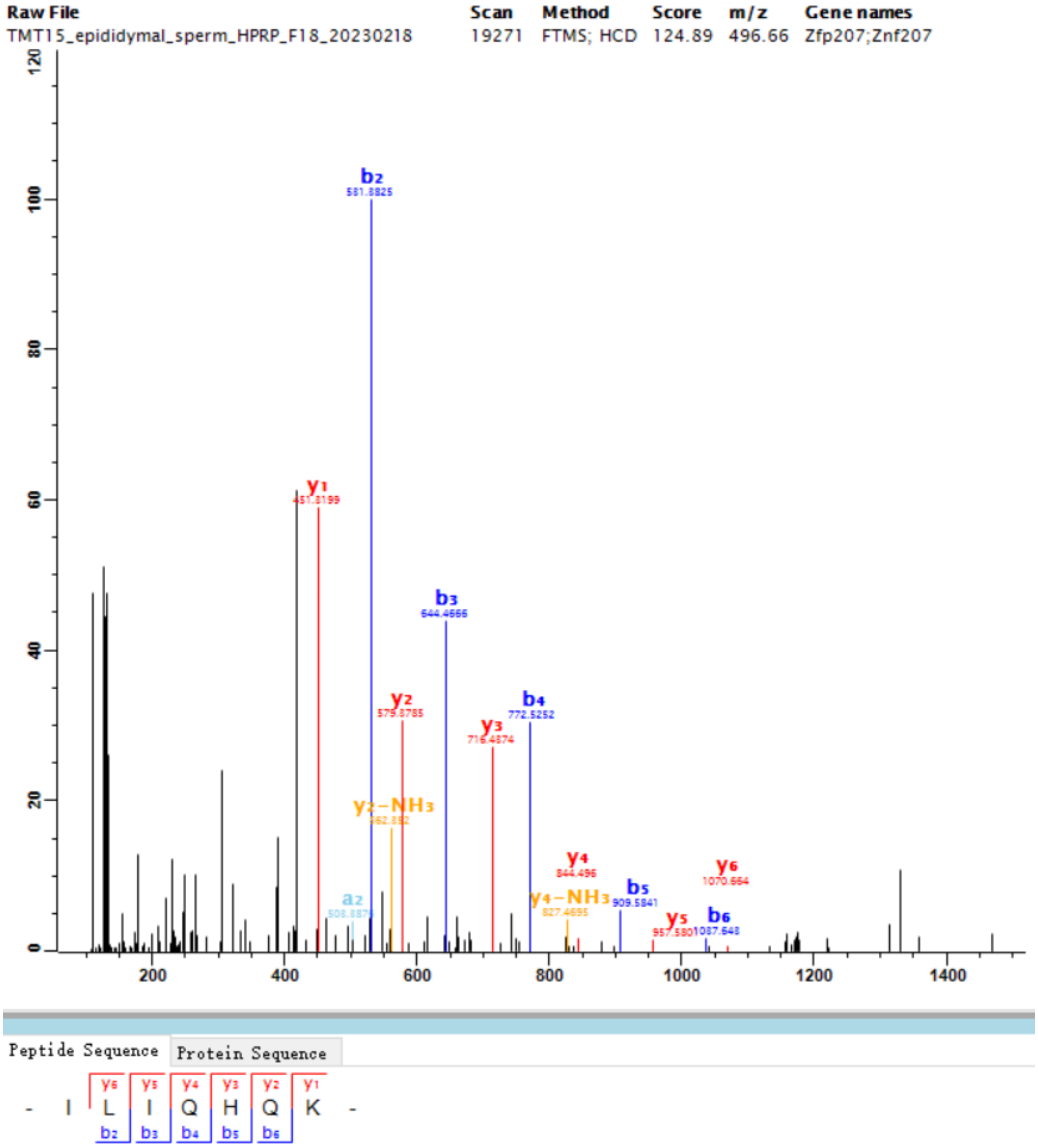
Raw File
TMT15_epididymal_sperm_HPRP_F18_20230218
Scan
19271
Method
FTMS; HCD
Score
124.89
m/z
496.66
Gene names
Zfp207;Znf207
b2
581.8825
y1
451.8199
b3
644.4666
y2
579.8785
b4
772.5252
y3
716.4874
y2-NH3
y4
844.496
y4-NH3
827.4695
b5
909.5841
y6
1070.664
y5
b6
a2
Peptide Sequence
Protein Sequence
- I L I Q H Q K -

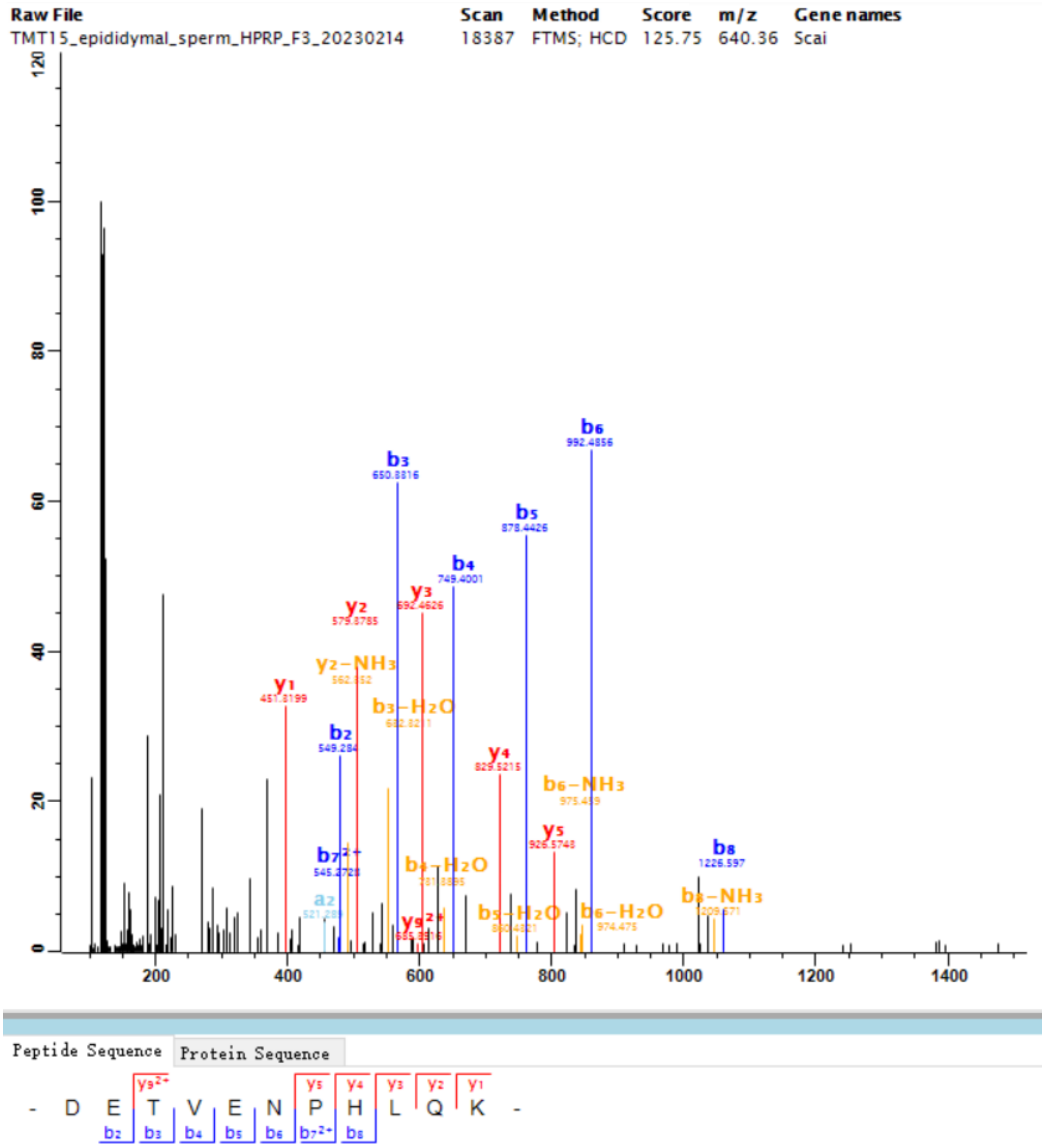

Raw File
TMT15_epididymal_sperm_HPRP_F3_20230214
Scan
18387
Method
FTMS; HCD
Score
125.75
m/z
640.36
Gene names
Scai
b6
992.4856
b3
650.8816
b5
878.4426
b4
749.4001
y3
y2
y2-NH3
y1
b3-H2O
b2
549.284
y4
b6-NH3
y5
b8
1226.597
b6-H2O
974.475
b5-H2O
a2
Peptide Sequence
Protein Sequence
- D E T V E N P H L Q K -

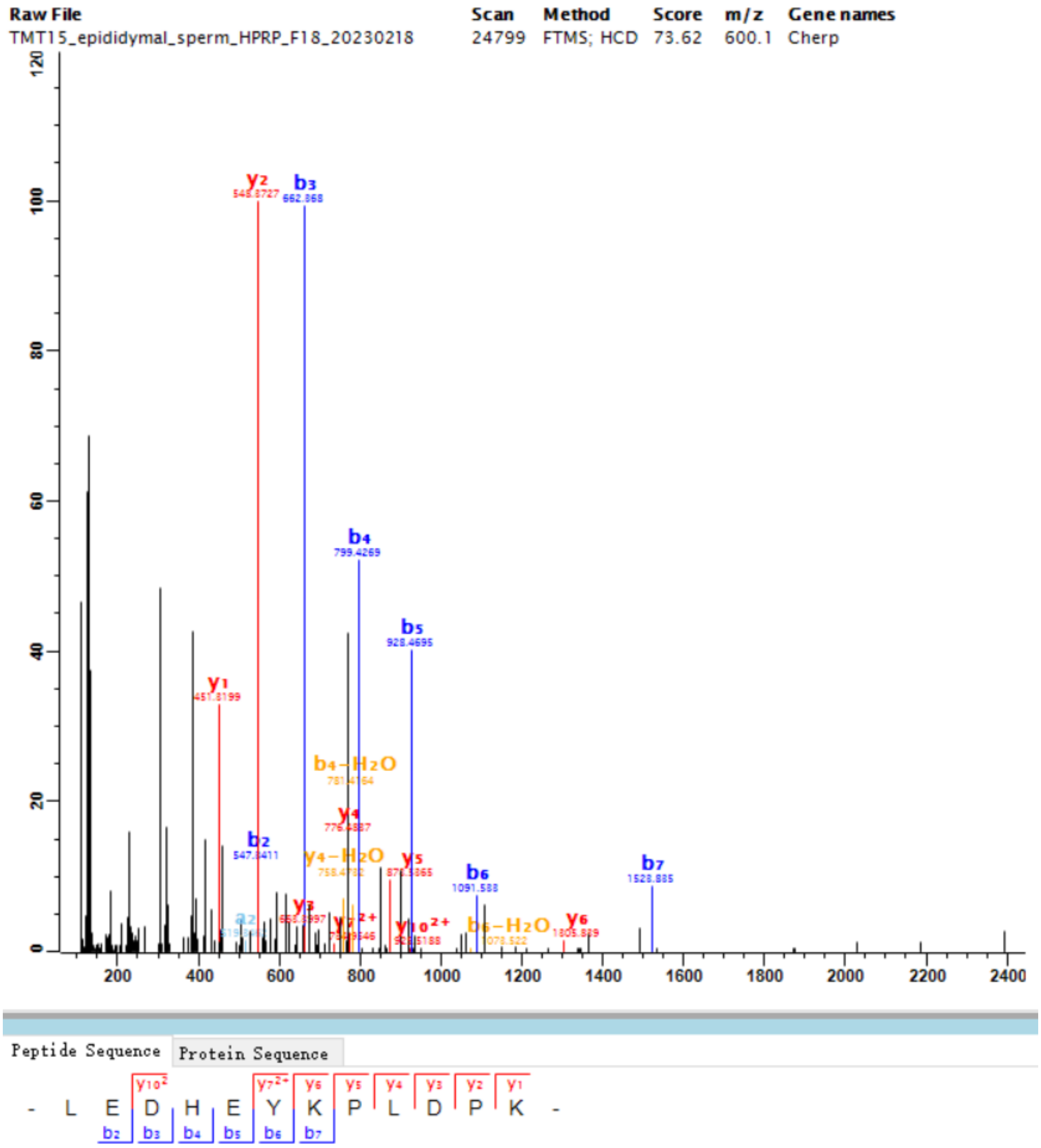
Raw File
TMT15_epididymal_sperm_HPRP_F18_20230218
Scan
24799
Method
FTMS; HCD
Score
73.62
m/z
600.1
Gene names
Cherp
y2 548.8727
b3 662.868
b4 799.4269
b5 928.4695
y1 451.8199
b4-H2O
y4
b2 547.3411
y4-H2O
y5
b6 1091.588
b7 1528.885
y3
y7 2+
y10 2+
b6-H2O
y6
Peptide Sequence
Protein Sequence
- L E D H E Y K P L D P K -

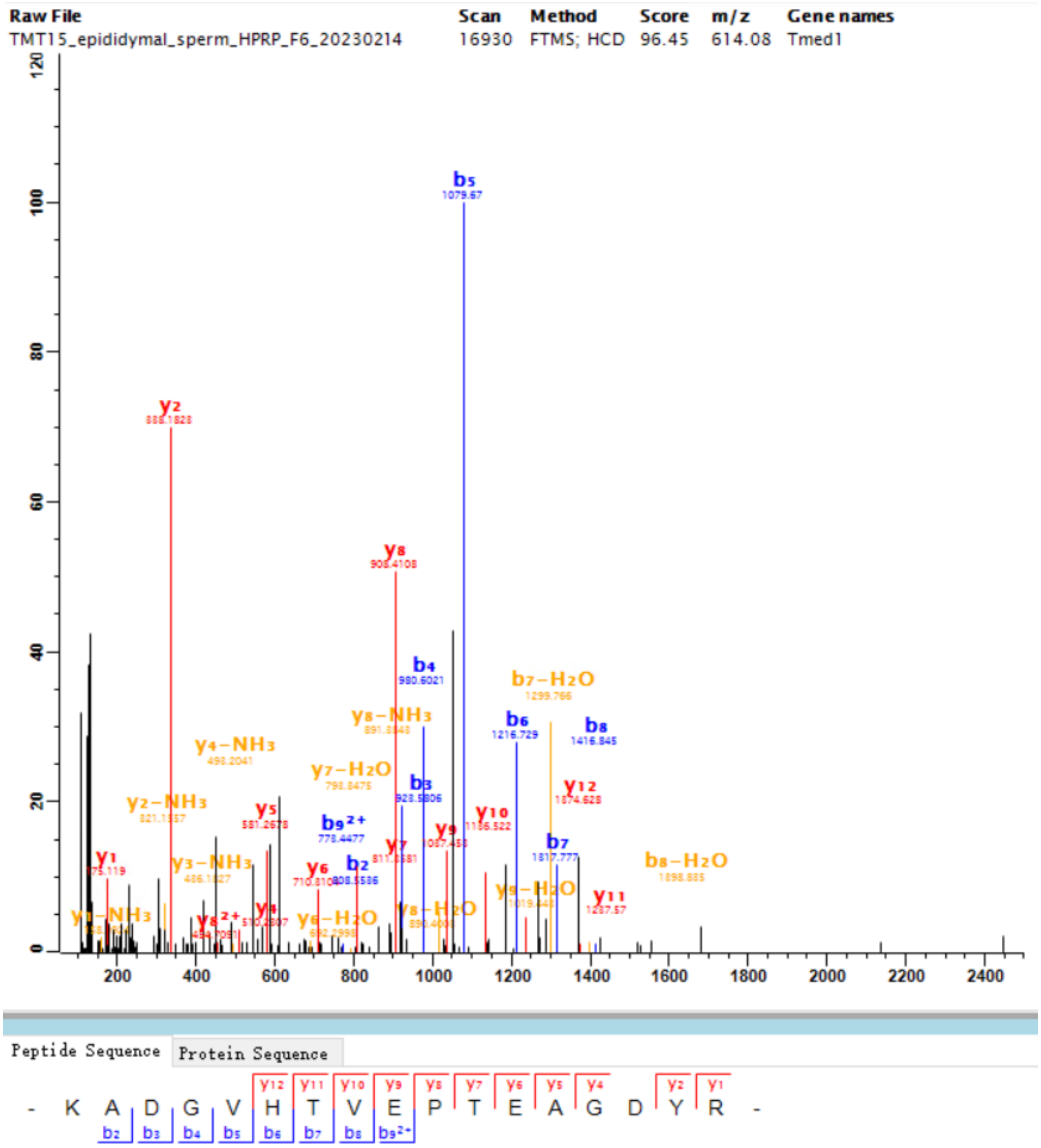
Raw File
TMT15_epididymal_sperm_HPRP_F6_20230214
Scan
16930
Method
FTMS; HCD
Score
96.45
m/z
614.08
Gene names
Tmed1
b5
y2
y8
b4
b7-H2O
y8-NH3
b6
b8
y4-NH3
y7-H2O
b3
y12
y2-NH3
y5
y10
b9 2+
y9
b7
y1
b2
y3-NH3
y6
y7
b8-H2O
y9-H2O
y11
y1-NH3
y6-H2O
y8-H2O
Peptide Sequence
Protein Sequence
- K A D G V H T V E P T E A G D Y R -

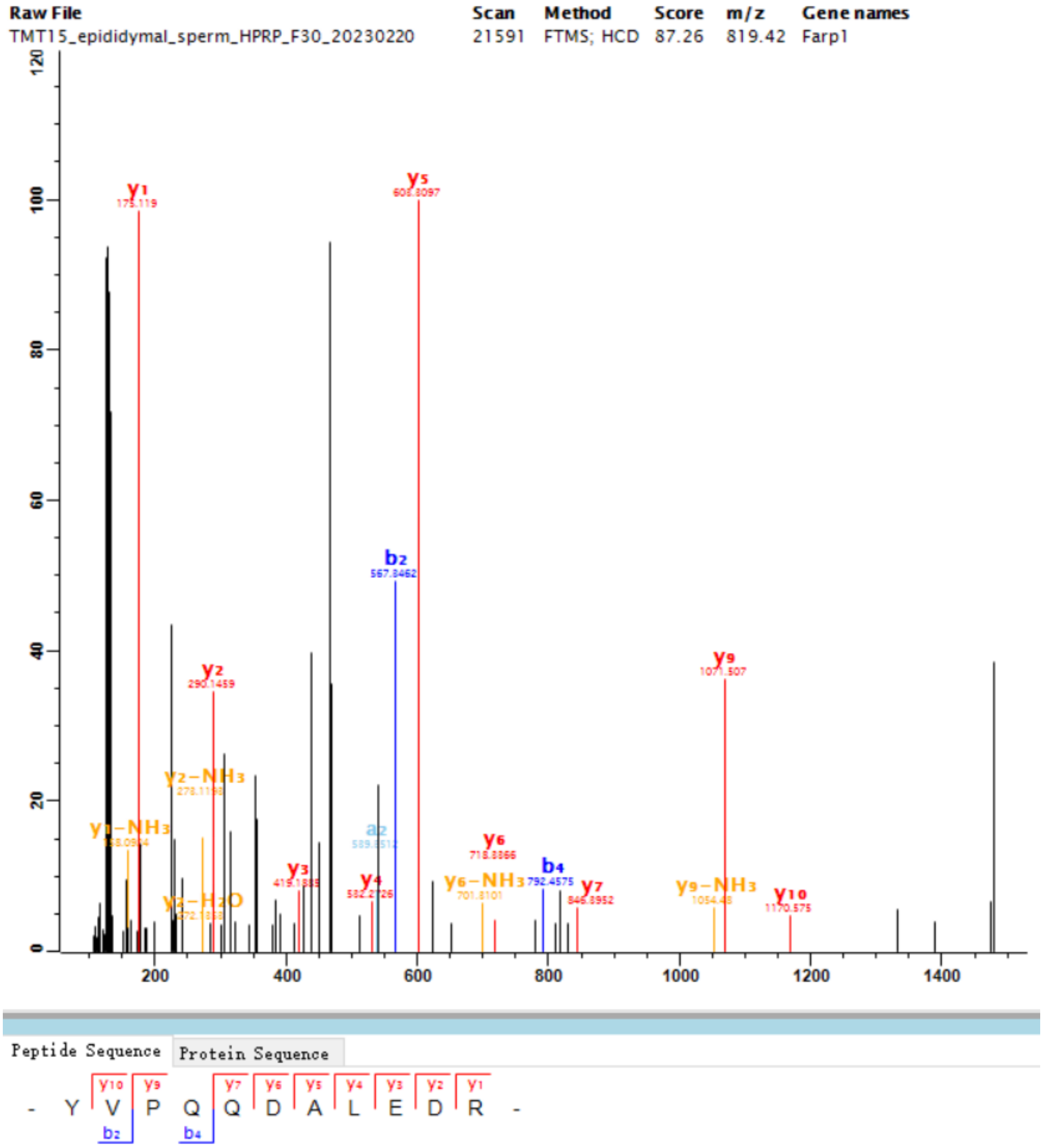
Raw File
TMT15_epididymal_sperm_HPRP_F30_20230220
Scan
21591
Method
FTMS; HCD
Score
87.26
m/z
819.42
Gene names
Farp1
y1
y5
b2
567.8462
y9
y2
290.1459
y2−NH3
y1−NH3
a2
y6
718.8866
b4
792.4575
y3
y4
y6−NH3
701.8101
y7
y9−NH3
y10
1170.575
y3−H2O
0
20
40
60
80
100
120
200
400
600
800
1000
1200
1400
Peptide Sequence
Protein Sequence
- Y V P Q Q D A L E D R -
y10 y9 y7 y6 y5 y4 y3 y2 y1
b2 b4

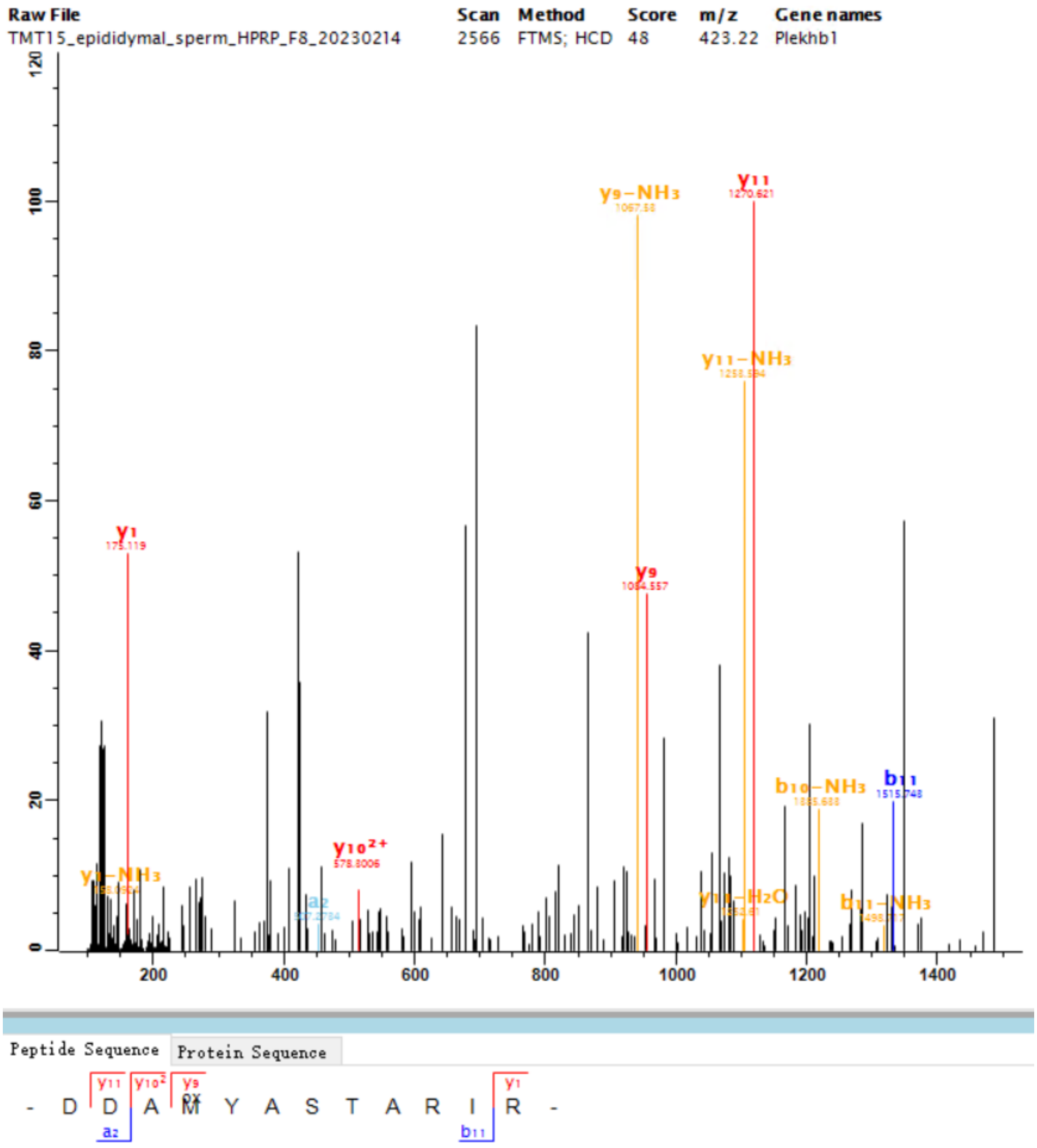
Raw File
TMT15_epididymal_sperm_HPRP_F8_20230214
Scan 2566
Method FTMS; HCD
Score 48
m/z 423.22
Gene names Plekhb1
y11
1270.621
y9-NH3
1067.58
y11-NH3
y1
175.119
y9
y10 2+
578.8006
b10-NH3
b11
1515.748
a2
b11-NH3
Peptide Sequence
Protein Sequence
- D D A M Y A S T A R I R -

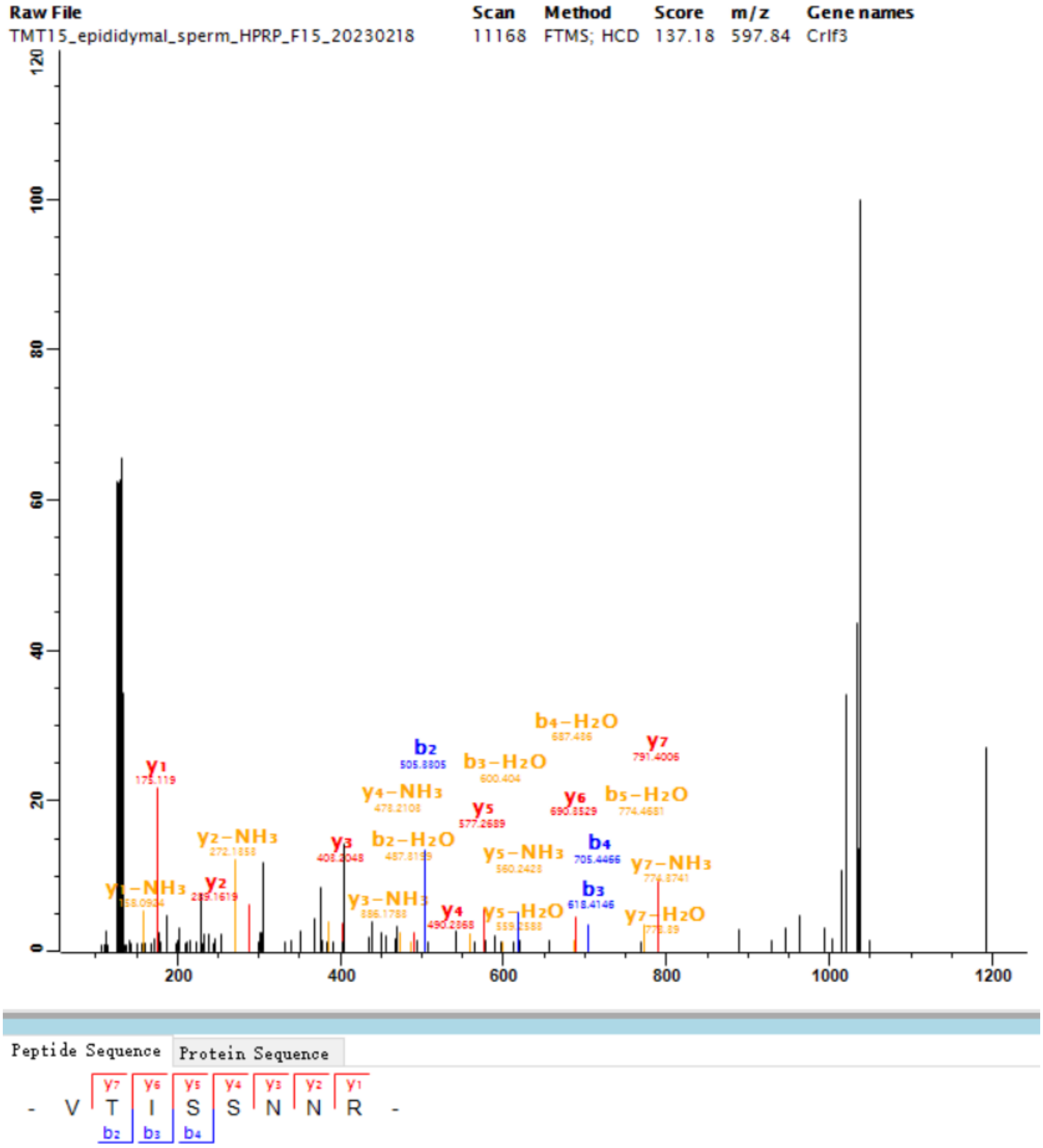

Raw File
TMT15_epididymal_sperm_HPRP_F15_20230218
Scan
11168
Method
FTMS; HCD
Score
137.18
m/z
597.84
Gene names
Crlf3
y1
y2
y3
y4
y5
y6
y7
b2
b3
b4
y1-NH3
y2-NH3
y3-NH3
y4-NH3
y5-NH3
y7-NH3
y5-H2O
y7-H2O
b2-H2O
b3-H2O
b4-H2O
b5-H2O
Peptide Sequence
Protein Sequence
- V T I S S N N R -

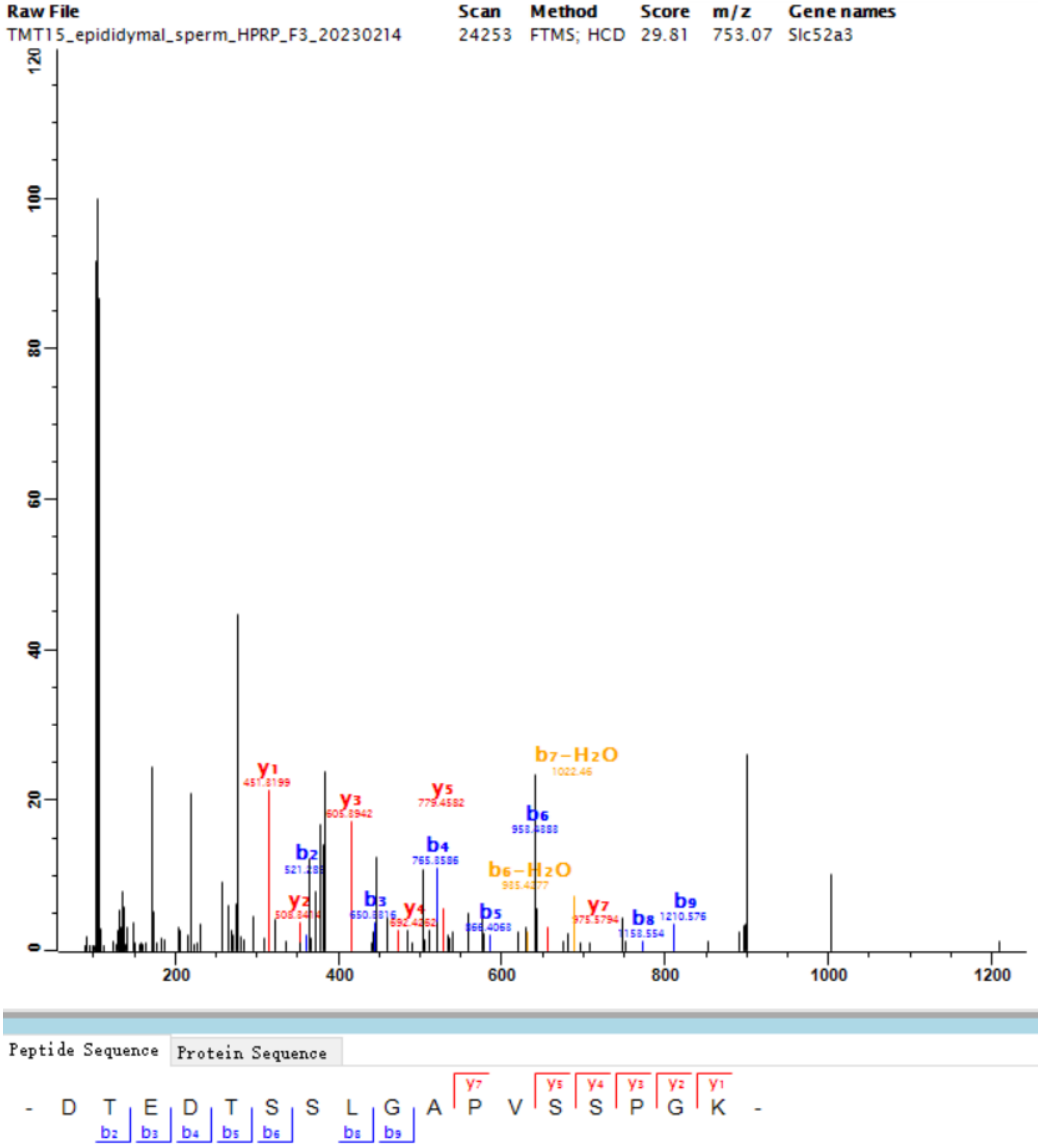

Raw File
TMT15_epididymal_sperm_HPRP_F3_20230214
Scan
24253
Method
FTMS; HCD
Score
29.81
m/z
753.07
Gene names
Slc52a3
0
20
40
60
80
100
120
200
400
600
800
1000
1200
y1
y2
y3
y4
y5
y7
b2
b3
b4
b5
b6
b8
b9
b7−H2O
1022.46
b6−H2O
Peptide Sequence
Protein Sequence
- D T E D T S S L G A P V S S P G K -

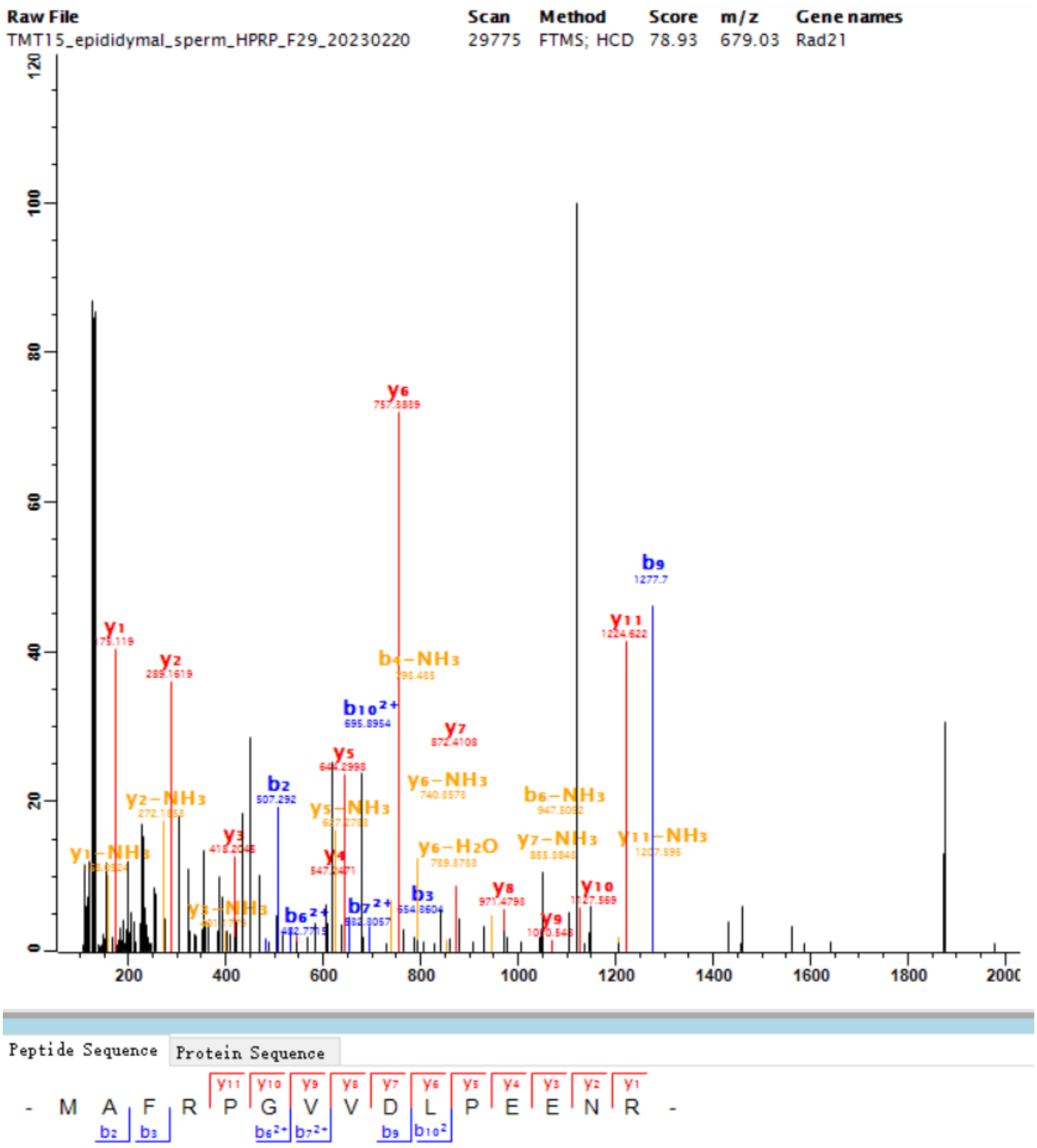

Raw File
TMT15_epididymal_sperm_HPRP_F29_20230220
Scan
29775
Method
FTMS; HCD
Score
78.93
m/z
679.03
Gene names
Rad21
y6
757.8889
b9
1277.7
y11
1224.622
y1
175.119
y2
289.1619
b10 2+
695.8954
y7
872.4108
y5
644.2998
b2
507.292
y3
418.2045
y4
547.2471
y8
971.4798
y10
1127.569
b3
654.3604
y2-NH3
y5-NH3
y6-NH3
740.8578
b6-NH3
947.5092
y6-H2O
789.8788
y7-NH3
855.8848
y11-NH3
1207.595
y1-NH3
y3-NH3
b6 2+
b7 2+
y9
b4-NH3
0
20
40
60
80
100
120
200
400
600
800
1000
1200
1400
1600
1800
Peptide Sequence
Protein Sequence
- M A F R P G V V D L P E E N R -

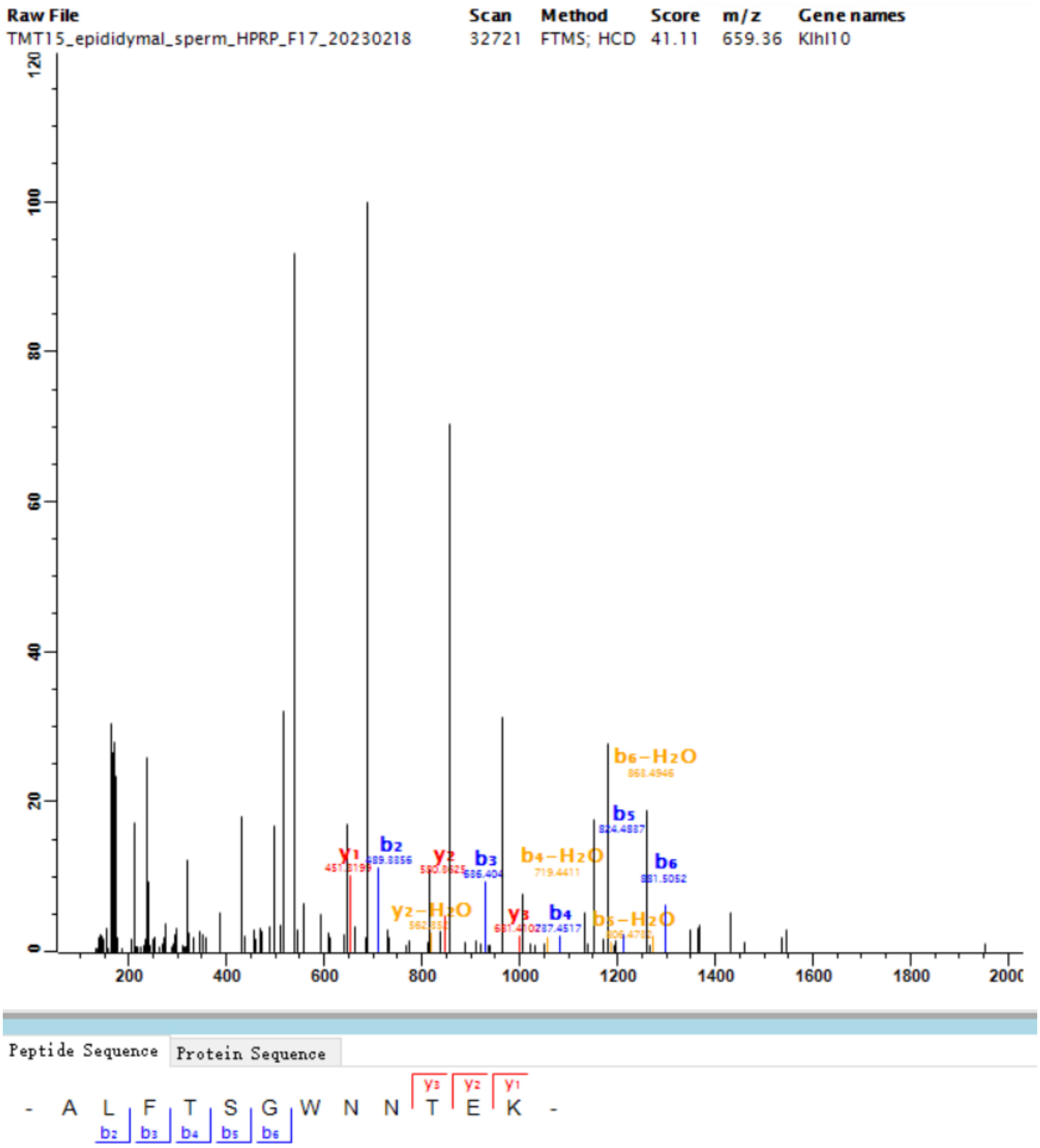
Raw File
TMT15_epididymal_sperm_HPRP_F17_20230218
Scan
32721
Method
FTMS; HCD
Score
41.11
m/z
659.36
Gene names
Klhl10
y1
b2
y2
b3
b4-H2O
719.4411
y2-H2O
y3
b4
b5-H2O
b5
824.4887
b6-H2O
b6
Peptide Sequence
Protein Sequence
- A L F T S G W N N T E K -
b2 b3 b4 b5 b6
y3 y2 y1

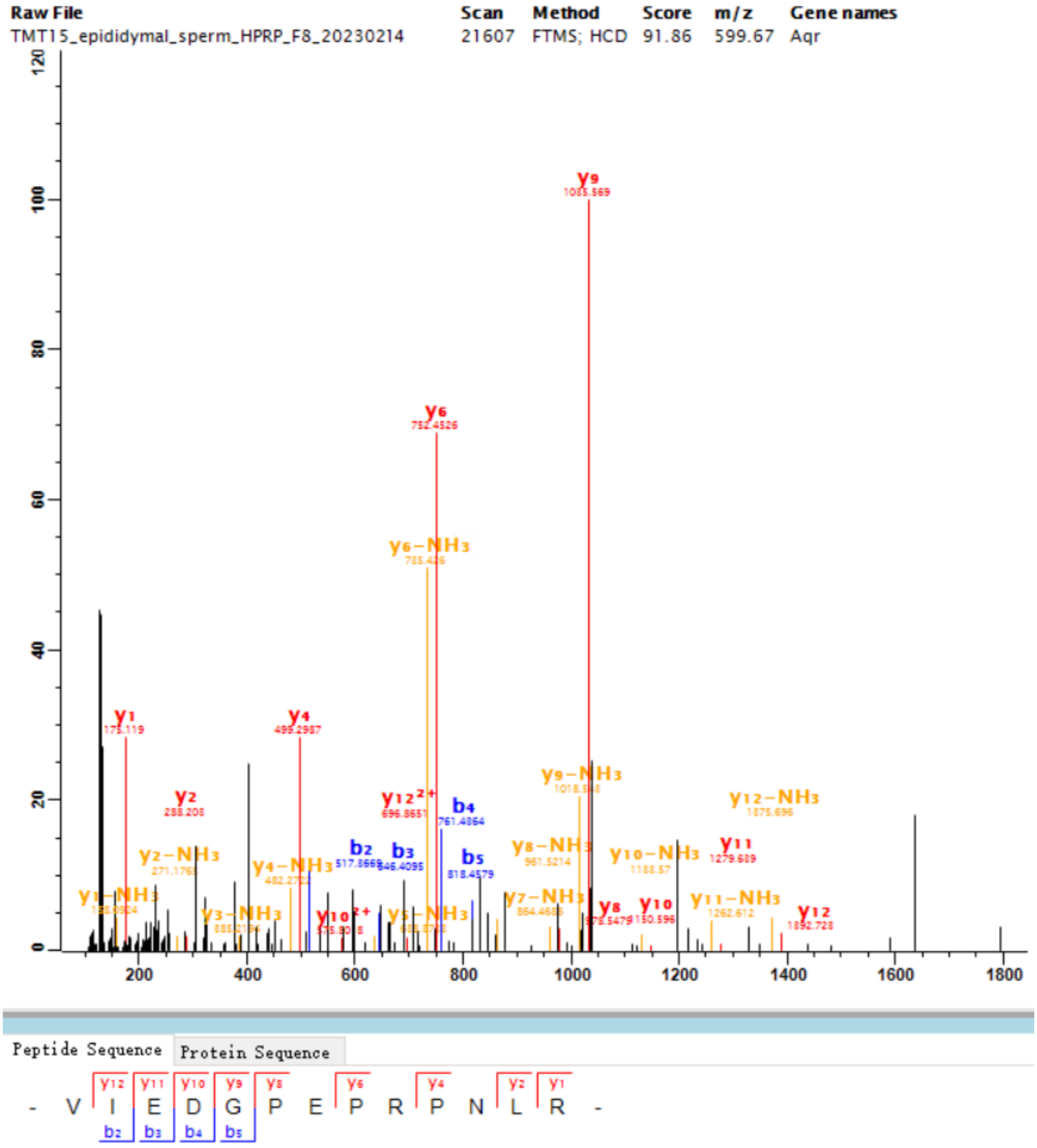
Raw File
TMT15_epididymal_sperm_HPRP_F8_20230214
Scan
21607
Method
FTMS; HCD
Score
91.86
m/z
599.67
Gene names
Aqr
y9
y6
752.4526
y6−NH3
y1
175.119
y4
y2
288.208
y2−NH3
y9−NH3
y12−NH3
1375.696
y12 2+
b4
761.4864
b2
b3
546.4095
b5
818.4579
y8−NH3
961.5214
y10−NH3
y11
1279.689
y7−NH3
y8
y10
1150.596
y11−NH3
1262.612
y12
y1−NH3
y3−NH3
y10 2+
y5−NH3
y4−NH3
0
20
40
60
80
100
120
200
400
600
800
1000
1200
1400
1600
1800
Peptide Sequence
Protein Sequence
- V I E D G P E P R P N L R -
y12 y11 y10 y9 y8 y6 y4 y2 y1
b2 b3 b4 b5

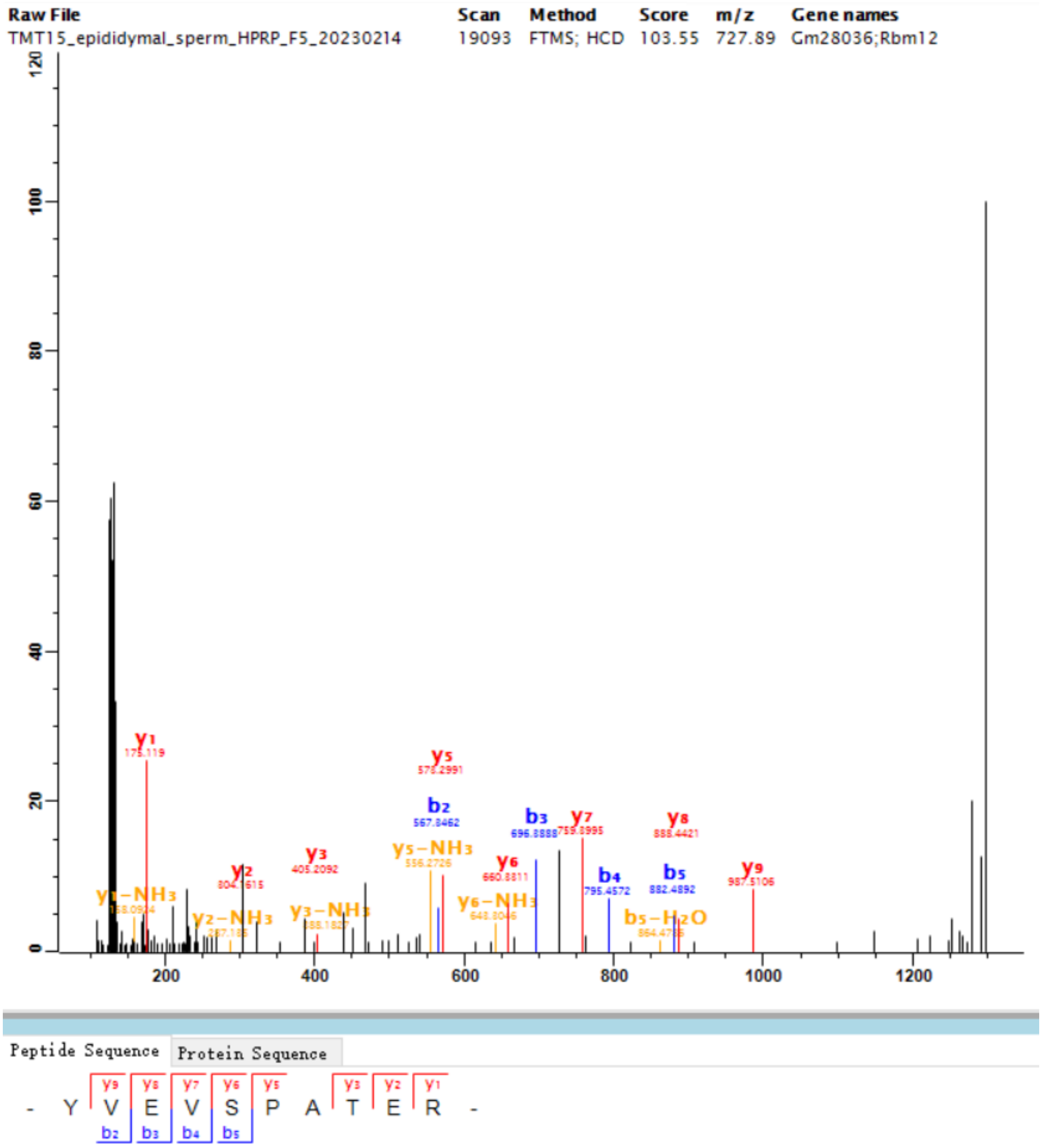

Raw File
TMT15_epididymal_sperm_HPRP_F5_20230214
Scan
19093
Method
FTMS; HCD
Score
103.55
m/z
727.89
Gene names
Gm28036;Rbm12
y1
175.119
y2
304.1615
y3
405.2092
y5
575.2991
b2
567.3462
y5-NH3
558.2726
b3
696.3888
y7
759.3995
y6
660.3811
y6-NH3
b4
795.4572
y8
888.4421
b5
882.4892
b5-H2O
y9
987.5106
y1-NH3
y2-NH3
y3-NH3
Peptide Sequence
Protein Sequence
- Y V E V S P A T E R -

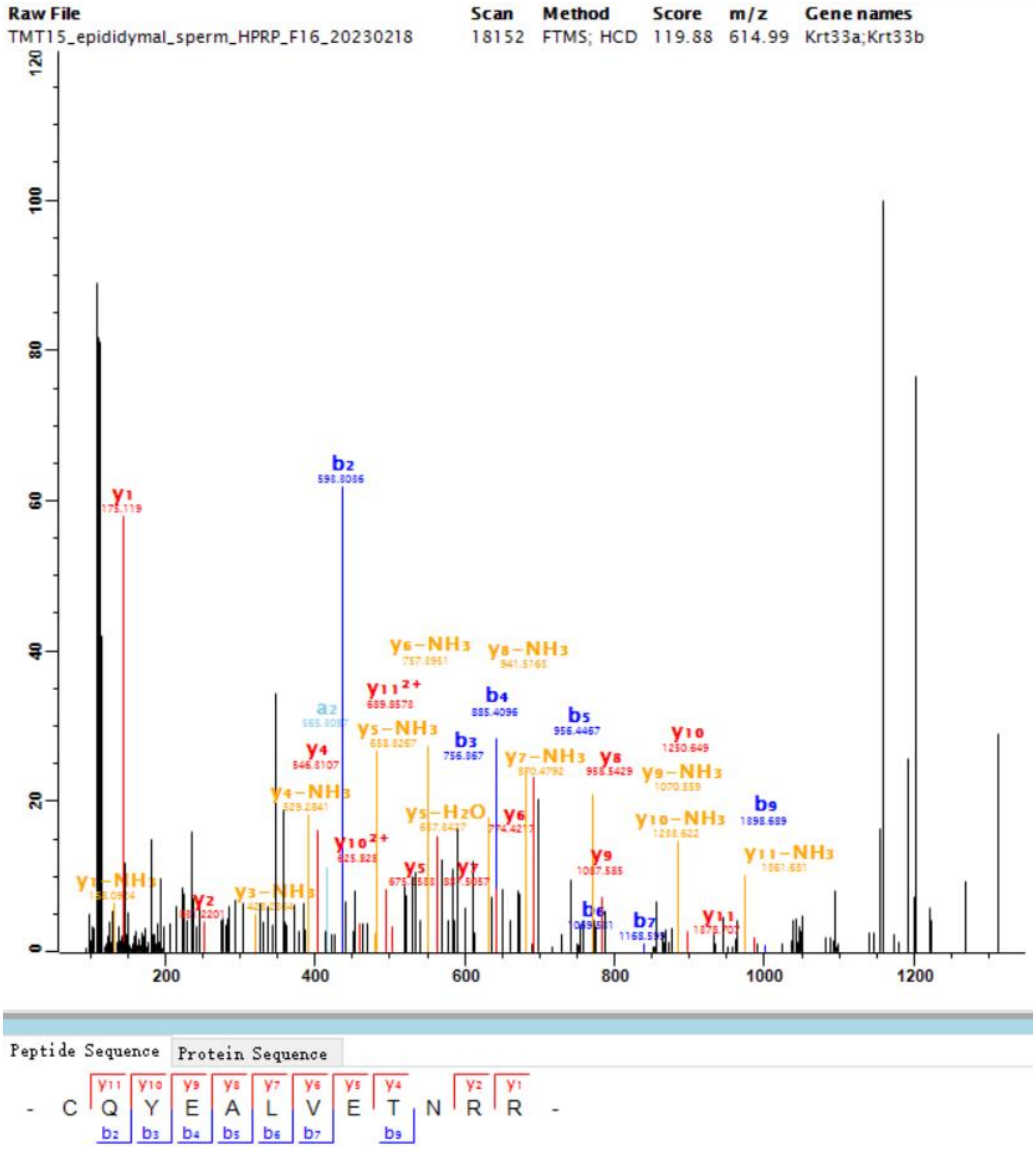
Raw File
TMT15_epididymal_sperm_HPRP_F16_20230218
Scan
18152
Method
FTMS; HCD
Score
119.88
m/z
614.99
Gene names
Krt33a;Krt33b
Peptide Sequence
Protein Sequence
- C Q Y E A L V E T N R R -

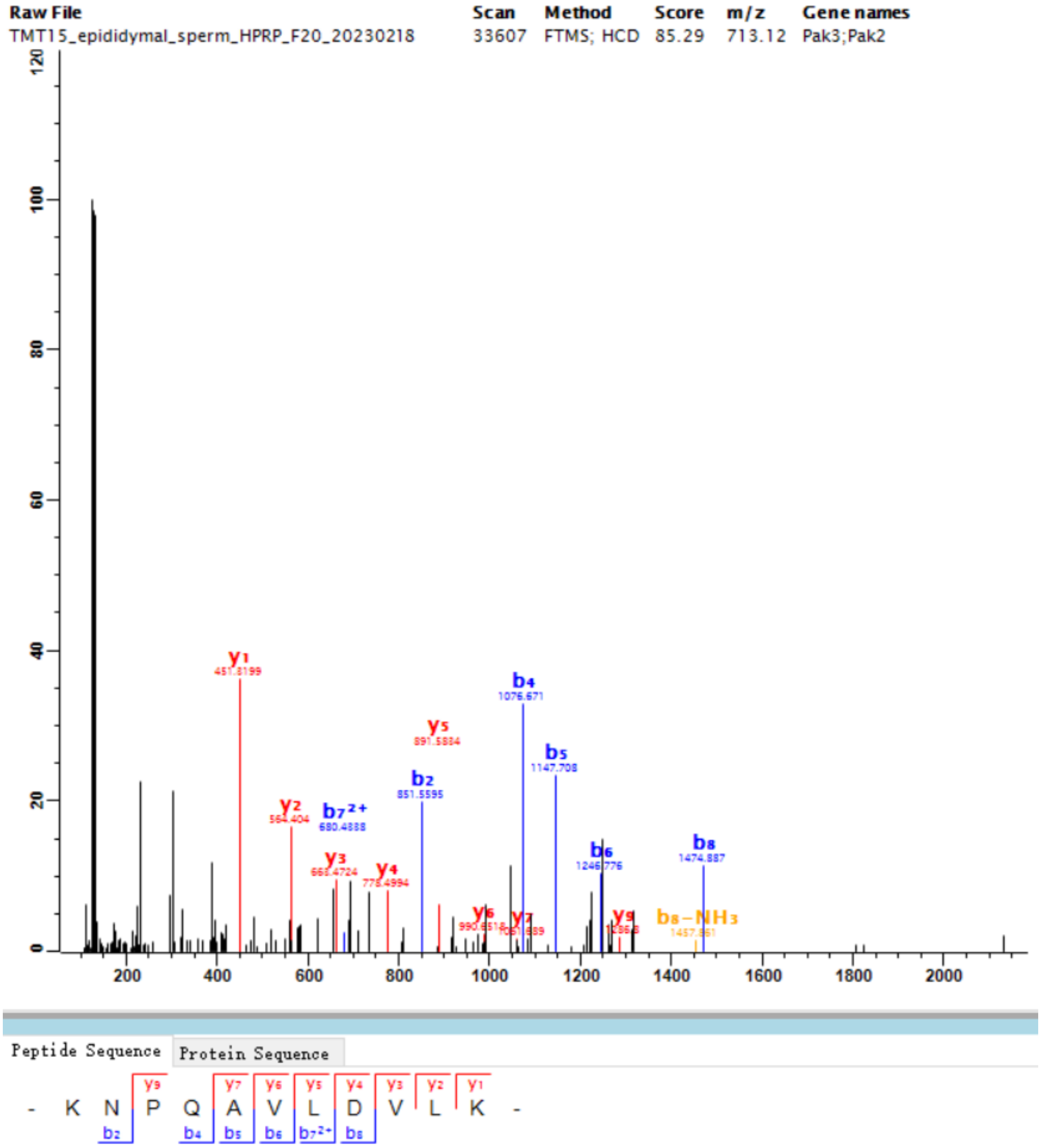
Raw File
TMT15_epididymal_sperm_HPRP_F20_20230218
Scan
33607
Method
FTMS; HCD
Score
85.29
m/z
713.12
Gene names
Pak3;Pak2
y1
y2
564.404
b7 2+
680.4888
y3
y4
y5
b2
851.5595
b4
1076.671
b5
1147.708
b6
b8
1474.887
y6
y7
y9
b8-NH3
Peptide Sequence
Protein Sequence
- K N P Q A V L D V L K -

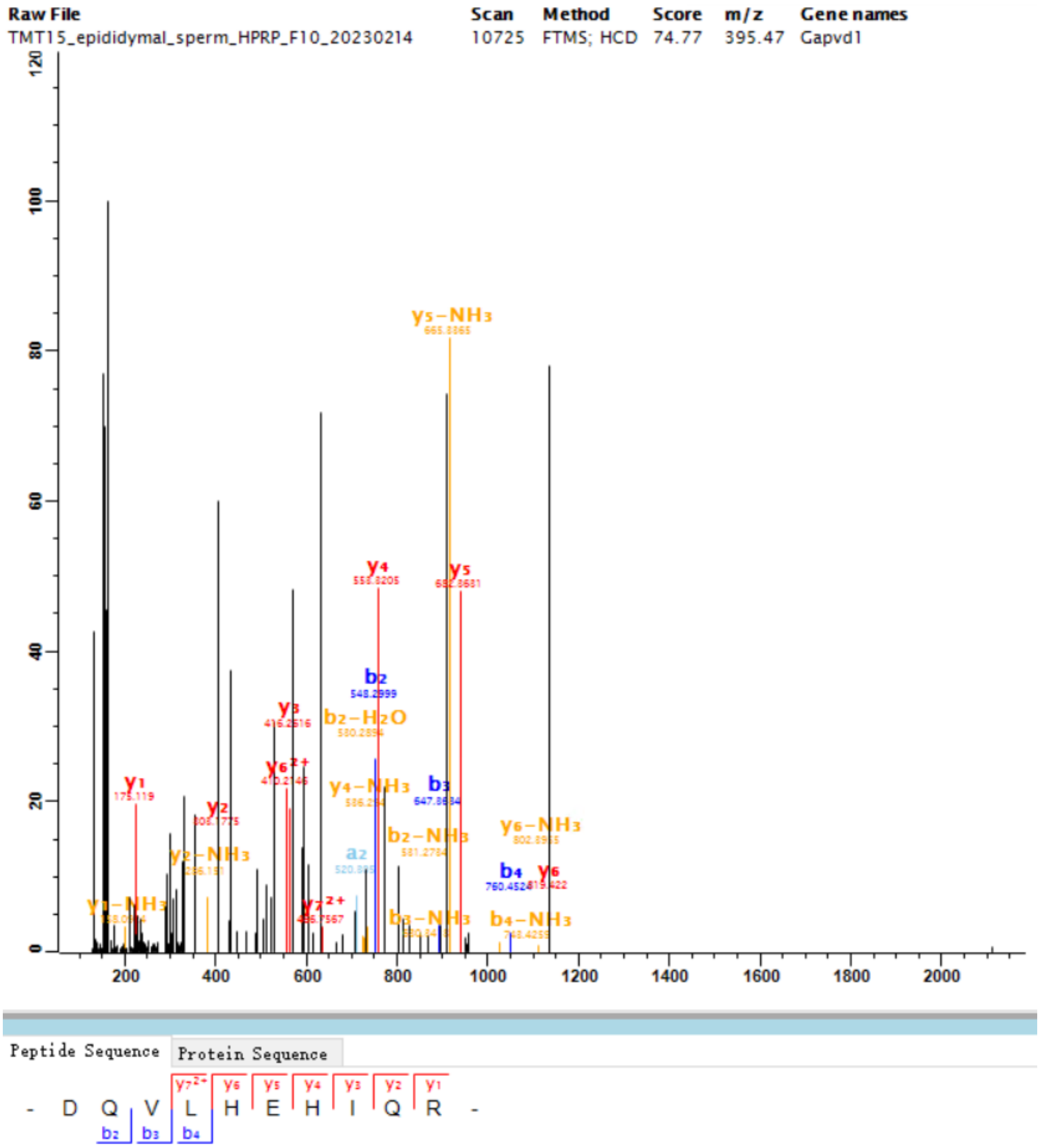

Raw File
TMT15_epididymal_sperm_HPRP_F10_20230214
Scan
10725
Method
FTMS; HCD
Score
74.77
m/z
395.47
Gene names
Gapvd1
200
400
600
800
1000
1200
1400
1600
1800
2000
Peptide Sequence
Protein Sequence
- D Q V L H E H I Q R -

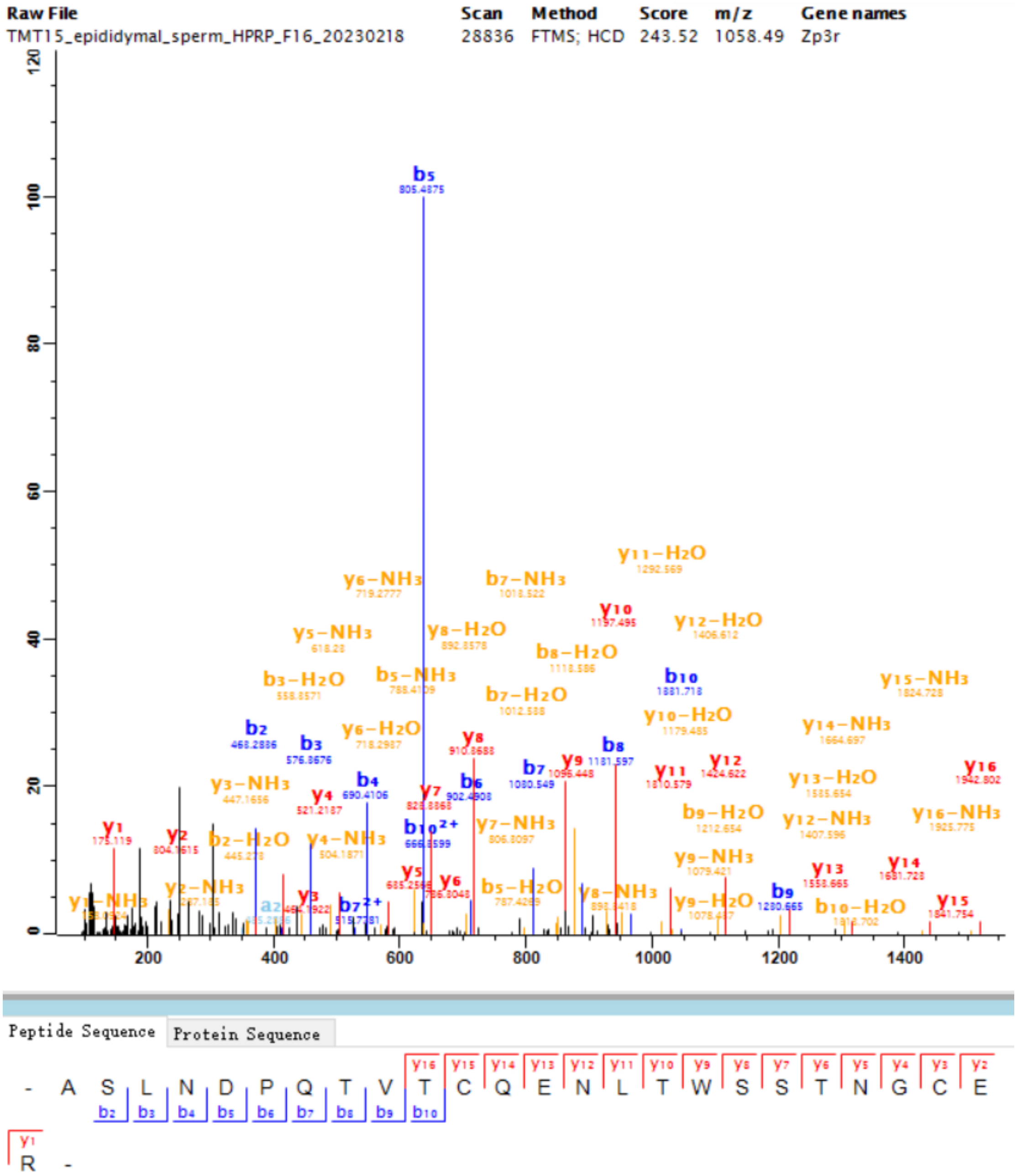

Raw File
TMT15_epididymal_sperm_HPRP_F16_20230218
Scan
28836
Method
FTMS; HCD
Score
243.52
m/z
1058.49
Gene names
Zp3r
b5
805.4875
200
400
600
800
1000
1200
1400
Peptide Sequence
Protein Sequence
- A S L N D P Q T V T C Q E N L T W S S T N G C E
R -

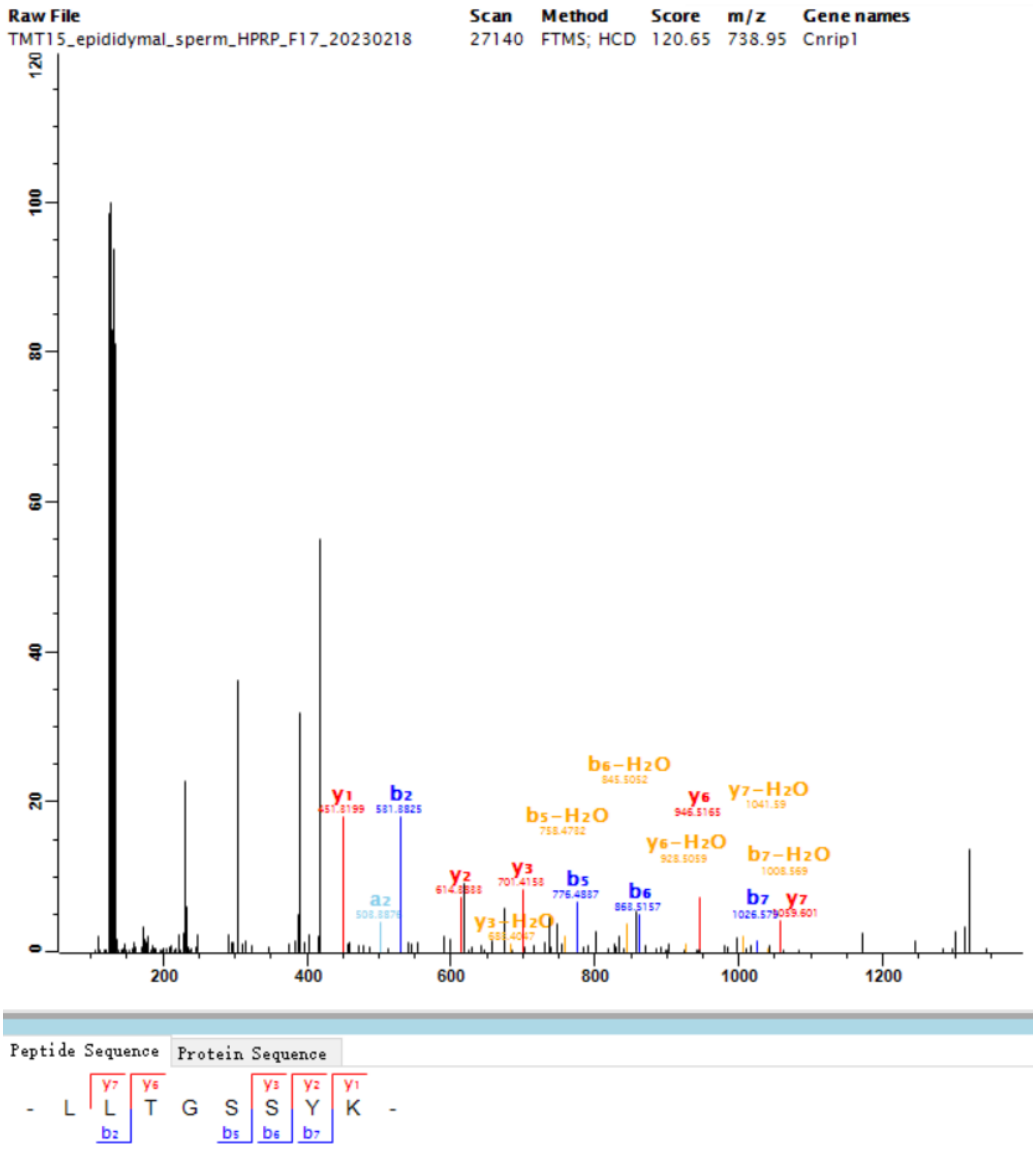
Raw File
TMT15_epididymal_sperm_HPRP_F17_20230218
Scan
27140
Method
FTMS; HCD
Score
120.65
m/z
738.95
Gene names
Cnrip1
y1
b2
581.8825
a2
y2
y3
701.4158
b5-H2O
758.4782
b5
776.4887
b6-H2O
845.5052
b6
868.5157
y6-H2O
928.5059
y6
946.5165
y7-H2O
1041.59
b7-H2O
1008.569
b7
y7
Peptide Sequence
Protein Sequence
- L L T G S S Y K -

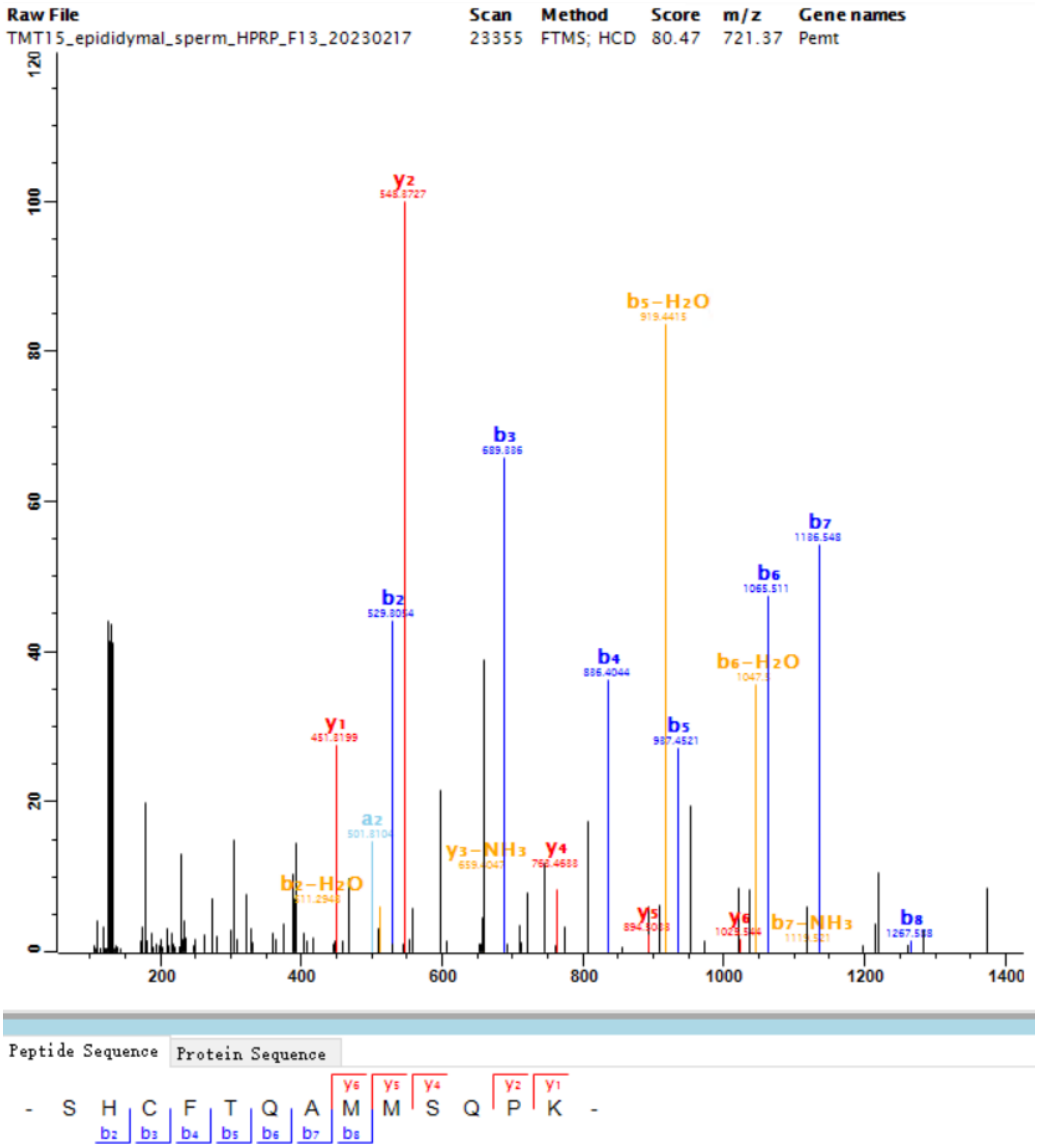

Raw File
TMT15_epididymal_sperm_HPRP_F13_20230217
Scan
23355
Method
FTMS; HCD
Score
80.47
m/z
721.37
Gene names
Pemt
y2
b5-H2O
919.4415
b3
689.886
b7
b6
1065.511
b2
b4
886.4044
b6-H2O
b5
987.4521
y1
451.8199
a2
501.8104
y3-NH3
y4
b2-H2O
y5
y6
b7-NH3
b8
Peptide Sequence
Protein Sequence
- S H C F T Q A M M S Q P K -

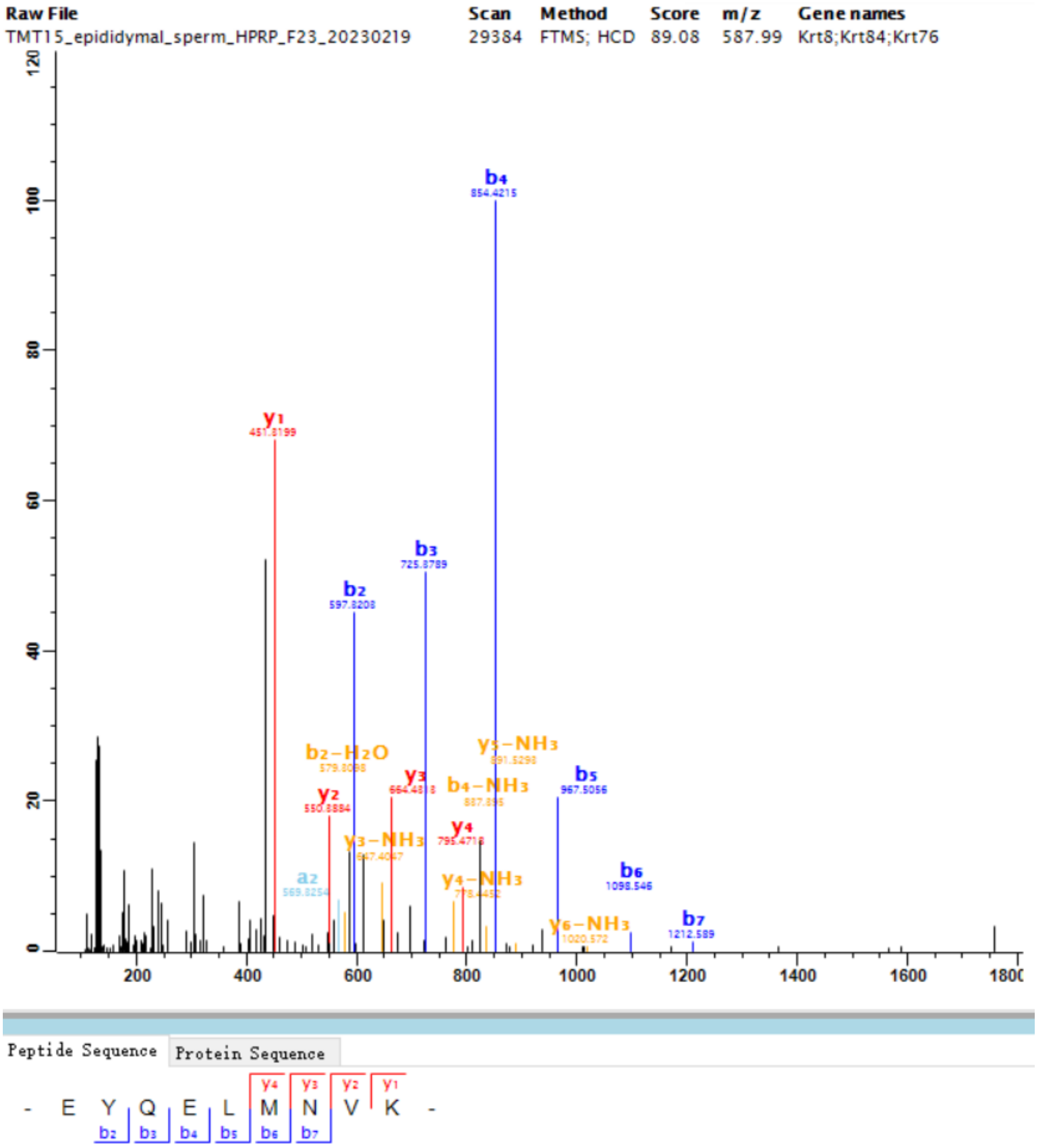
Raw File
TMT15_epididymal_sperm_HPRP_F23_20230219
Scan 29384
Method FTMS; HCD
Score 89.08
m/z 587.99
Gene names Krt8;Krt84;Krt76
b4 854.4215
y1 451.8199
b3 725.8789
b2 597.8208
b2-H2O 579.8098
y5-NH3 891.5298
b5 967.5056
y3 664.4818
b4-NH3 887.896
y2 550.8884
y4 795.4718
y3-NH3 647.4047
a2 569.8254
b6 1098.546
y4-NH3 778.4452
y6-NH3 1020.572
b7 1212.589
0 20 40 60 80 100 120
200 400 600 800 1000 1200 1400 1600 1800
Peptide Sequence
Protein Sequence
- E Y Q E L M N V K -
y4 y3 y2 y1
b2 b3 b4 b5 b6 b7

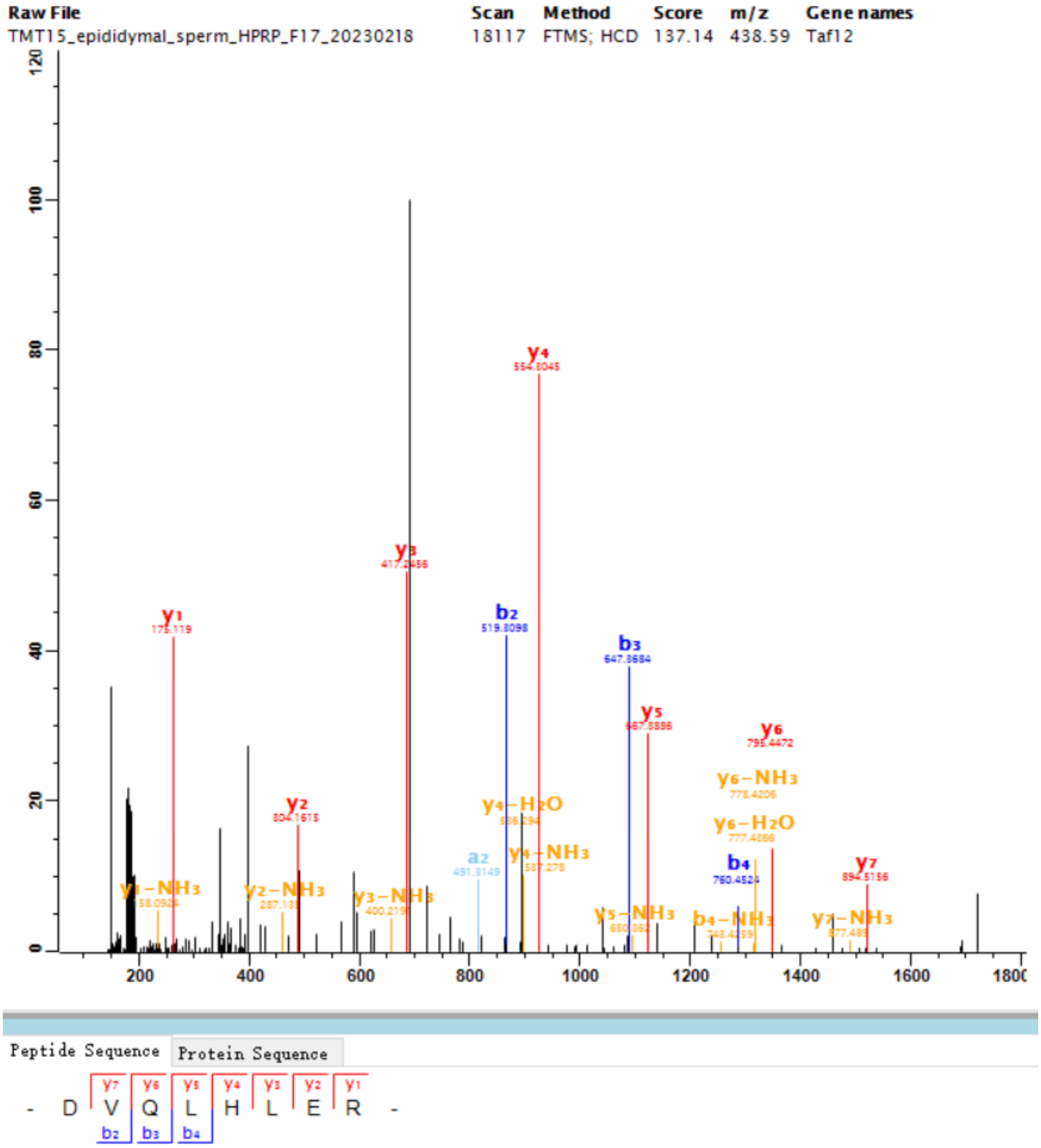

Raw File
TMT15_epididymal_sperm_HPRP_F17_20230218
Scan
18117
Method
FTMS; HCD
Score
137.14
m/z
438.59
Gene names
Taf12
y1
175.119
y2
y3
y4
554.8045
y5
y6
y7
b2
519.8098
b3
647.8684
b4
760.4524
a2
491.8149
y4−H2O
y6−NH3
778.4206
y6−H2O
777.4866
y4−NH3
y1−NH3
y2−NH3
y3−NH3
y5−NH3
b4−NH3
y7−NH3
Peptide Sequence
Protein Sequence
- D V Q L H L E R -

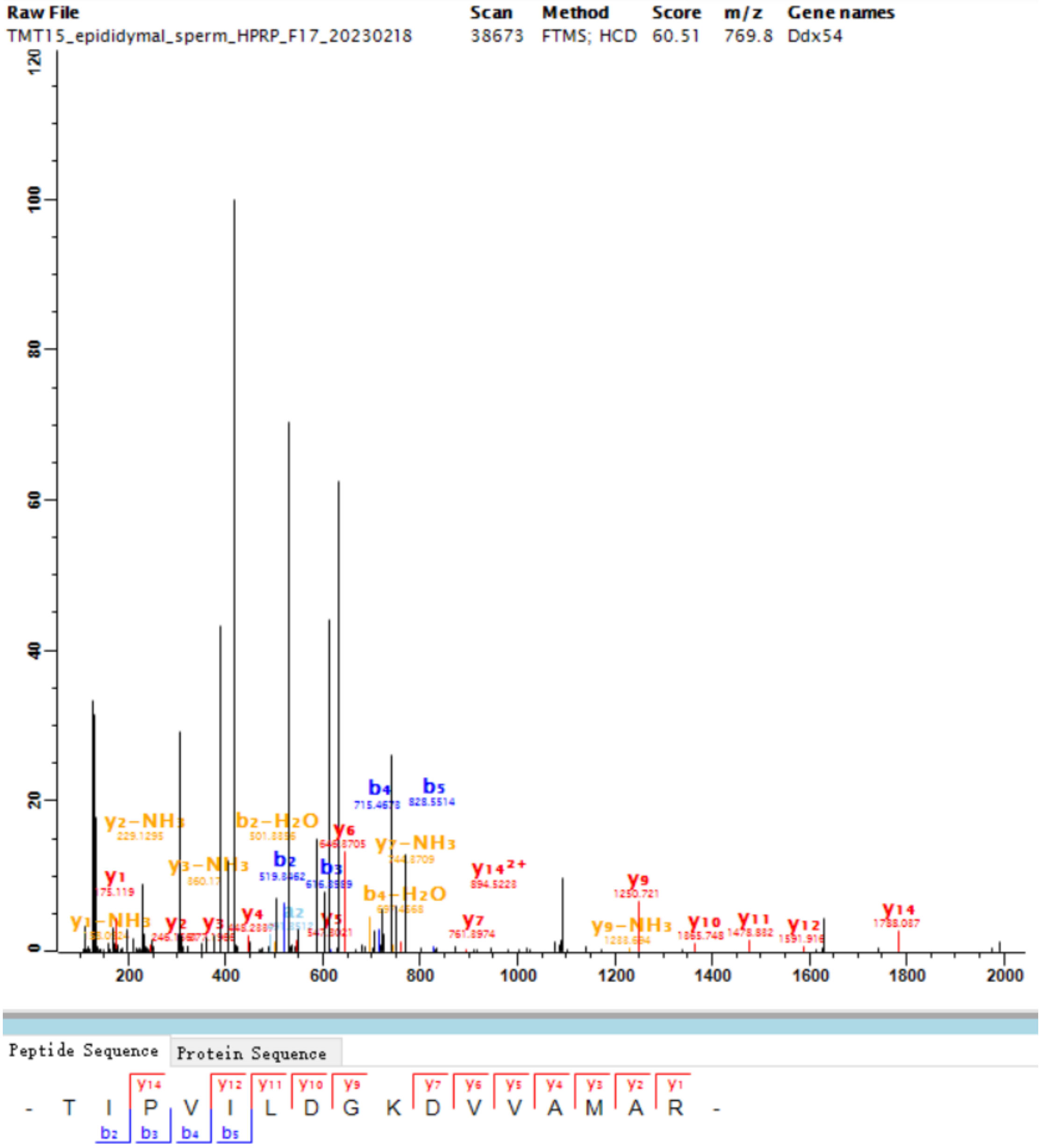
Raw File
TMT15_epididymal_sperm_HPRP_F17_20230218
Scan
38673
Method
FTMS; HCD
Score
60.51
m/z
769.8
Gene names
Ddx54
y2−NH3
229.1295
b2−H2O
501.8886
y6
y7−NH3
b4
715.4678
b5
828.5514
y3−NH3
b2
519.8462
b3
616.8889
y1
175.119
y14 2+
894.5228
y9
1250.721
b4−H2O
y1−NH3
y2
y3
y4
y5
y7
761.8974
y9−NH3
y10
y11
1478.882
y12
y14
0
20
40
60
80
100
120
200
400
600
800
1000
1200
1400
1600
1800
2000
Peptide Sequence
Protein Sequence
- T I P V I L D G K D V V A M A R -
y14 y12 y11 y10 y9 y7 y6 y5 y4 y3 y2 y1
b2 b3 b4 b5

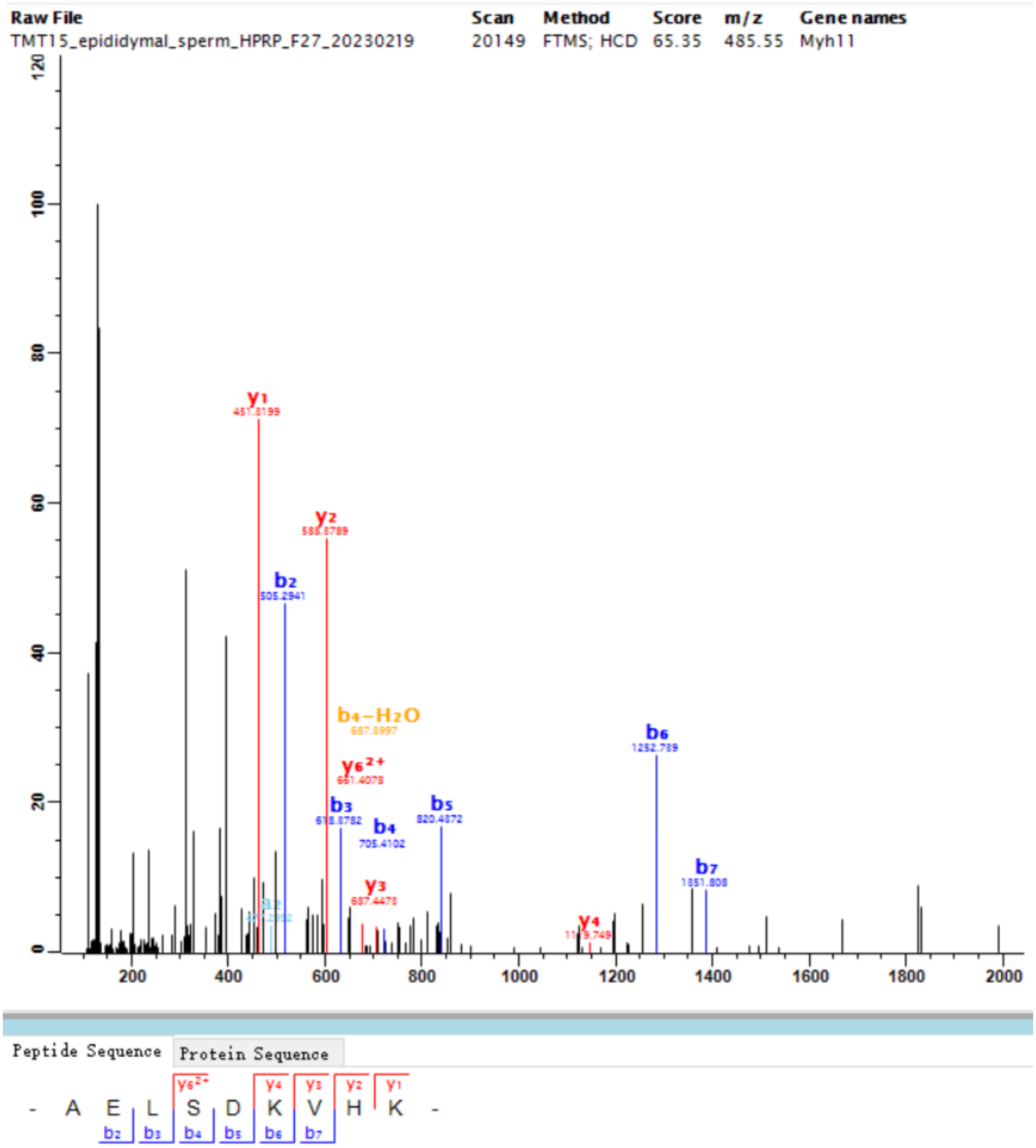
Raw File
TMT15_epididymal_sperm_HPRP_F27_20230219
Scan
20149
Method
FTMS; HCD
Score
65.35
m/z
485.55
Gene names
Myh11
y1
451.8199
y2
b2
505.2941
b4−H2O
687.8997
y6 2+
b3
b4
705.4102
b5
820.4872
b6
1252.789
b7
1351.808
y3
687.4478
y4
0
20
40
60
80
100
120
200
400
600
800
1000
1200
1400
1600
1800
2000
Peptide Sequence
Protein Sequence
- A E L S D K V H K -
y6 2+ y4 y3 y2 y1
b2 b3 b4 b5 b6 b7

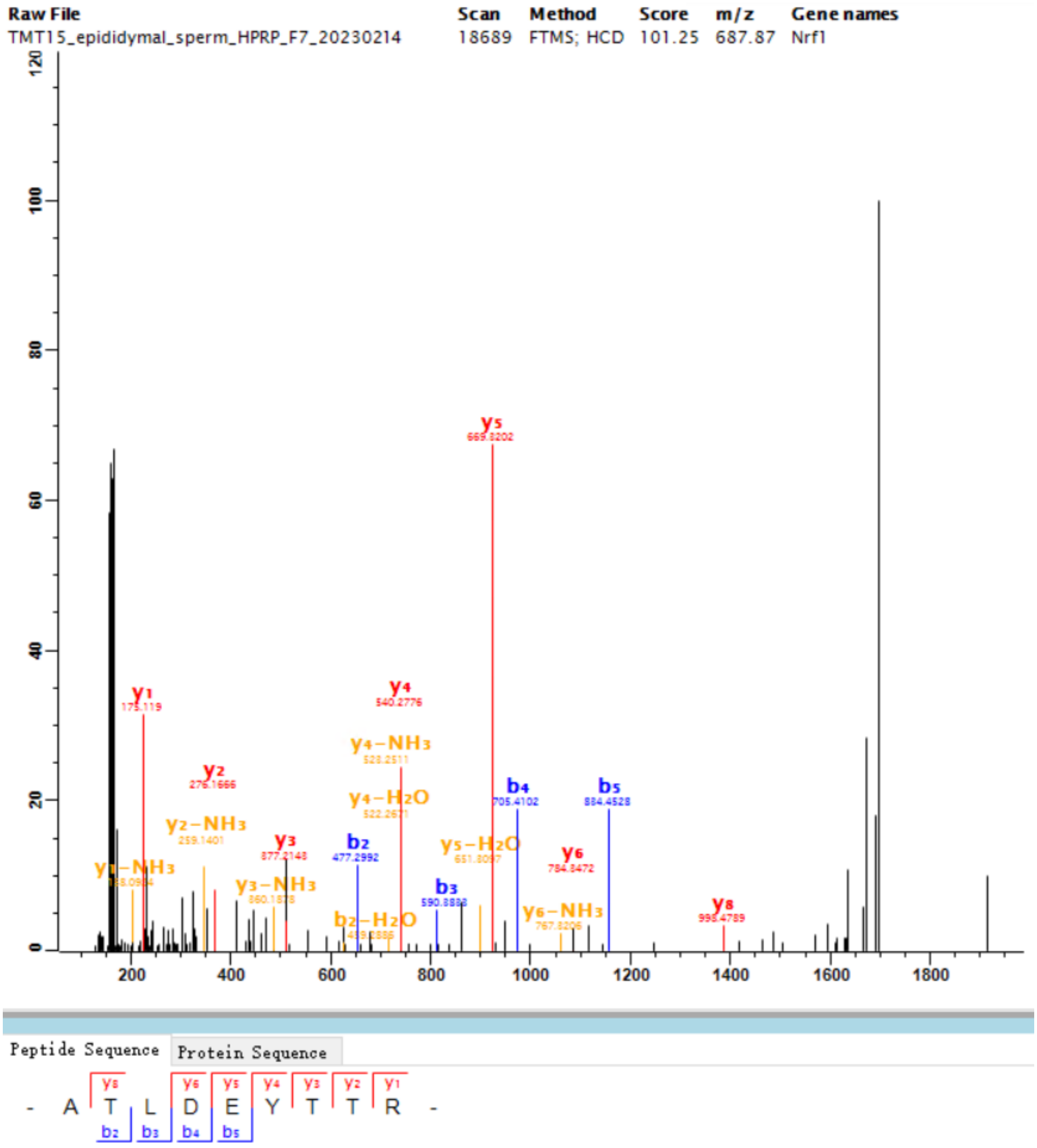

Raw File
TMT15_epididymal_sperm_HPRP_F7_20230214
Scan
18689
Method
FTMS; HCD
Score
101.25
m/z
687.87
Gene names
Nrf1
y1
175.119
y2
276.1666
y2-NH3
259.1401
y3
y3-NH3
y4
540.2776
y4-NH3
523.2511
y4-H2O
522.2671
y5
b2
477.2992
b2-H2O
b3
b4
705.4102
b5
884.4528
y5-H2O
y6
y6-NH3
y1-NH3
y8
0
20
40
60
80
100
120
200
400
600
800
1000
1200
1400
1600
1800
Peptide Sequence
Protein Sequence
- A T L D E Y T T R -
y8 y6 y5 y4 y3 y2 y1
b2 b3 b4 b5

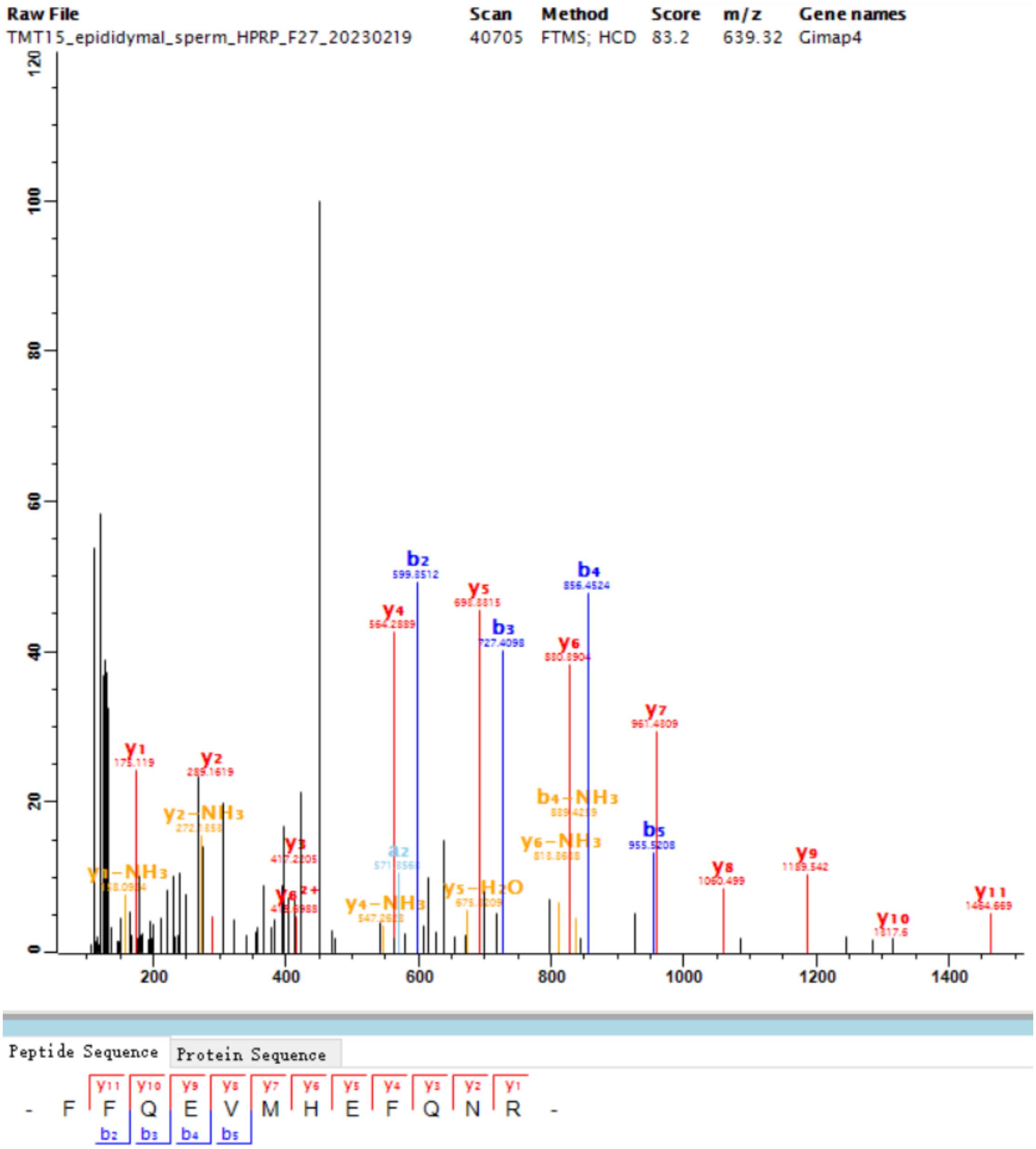

Raw File
TMT15_epididymal_sperm_HPRP_F27_20230219
Scan
40705
Method
FTMS; HCD
Score
83.2
m/z
639.32
Gene names
Gimap4
b2
599.8512
b4
856.4524
y5
y4
564.2889
b3
727.4098
y6
y7
961.4809
y1
175.119
y2
b4-NH3
y2-NH3
b5
955.5208
y6-NH3
y3
y8
y9
y10
y11
y1-NH3
y5-H2O
y4-NH3
Peptide Sequence
Protein Sequence
- F F Q E V M H E F Q N R -

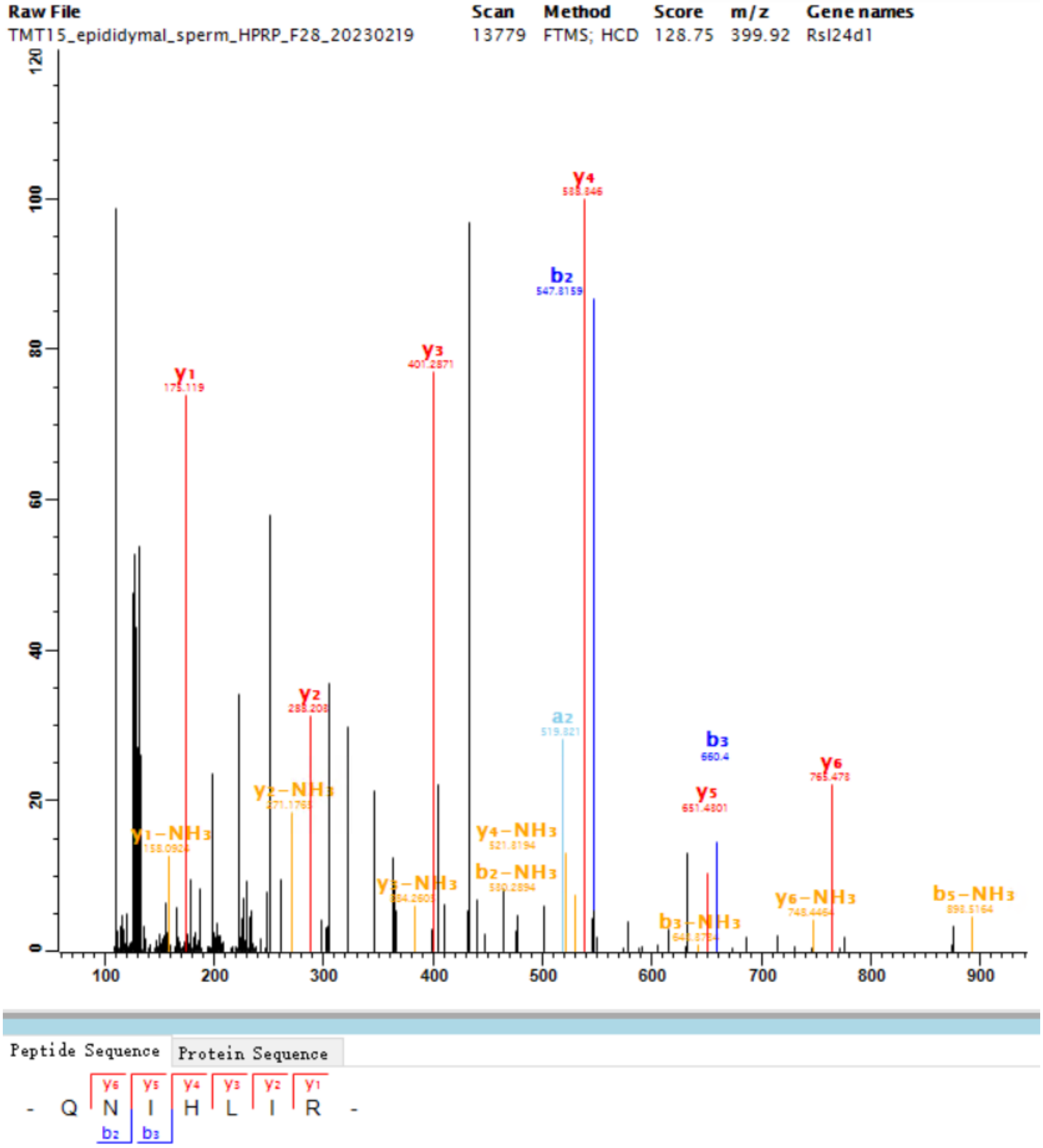

Raw File
TMT15_epididymal_sperm_HPRP_F28_20230219
Scan
13779
Method
FTMS; HCD
Score
128.75
m/z
399.92
Gene names
Rsl24d1
y4
b2
547.8159
y1
175.119
y3
401.2871
y2
a2
519.821
b3
660.4
y6
y5
651.4801
y1-NH3
y4-NH3
521.8194
b2-NH3
530.2894
y6-NH3
748.4464
b5-NH3
898.5164
Peptide Sequence
Protein Sequence
- Q N I H L I R -

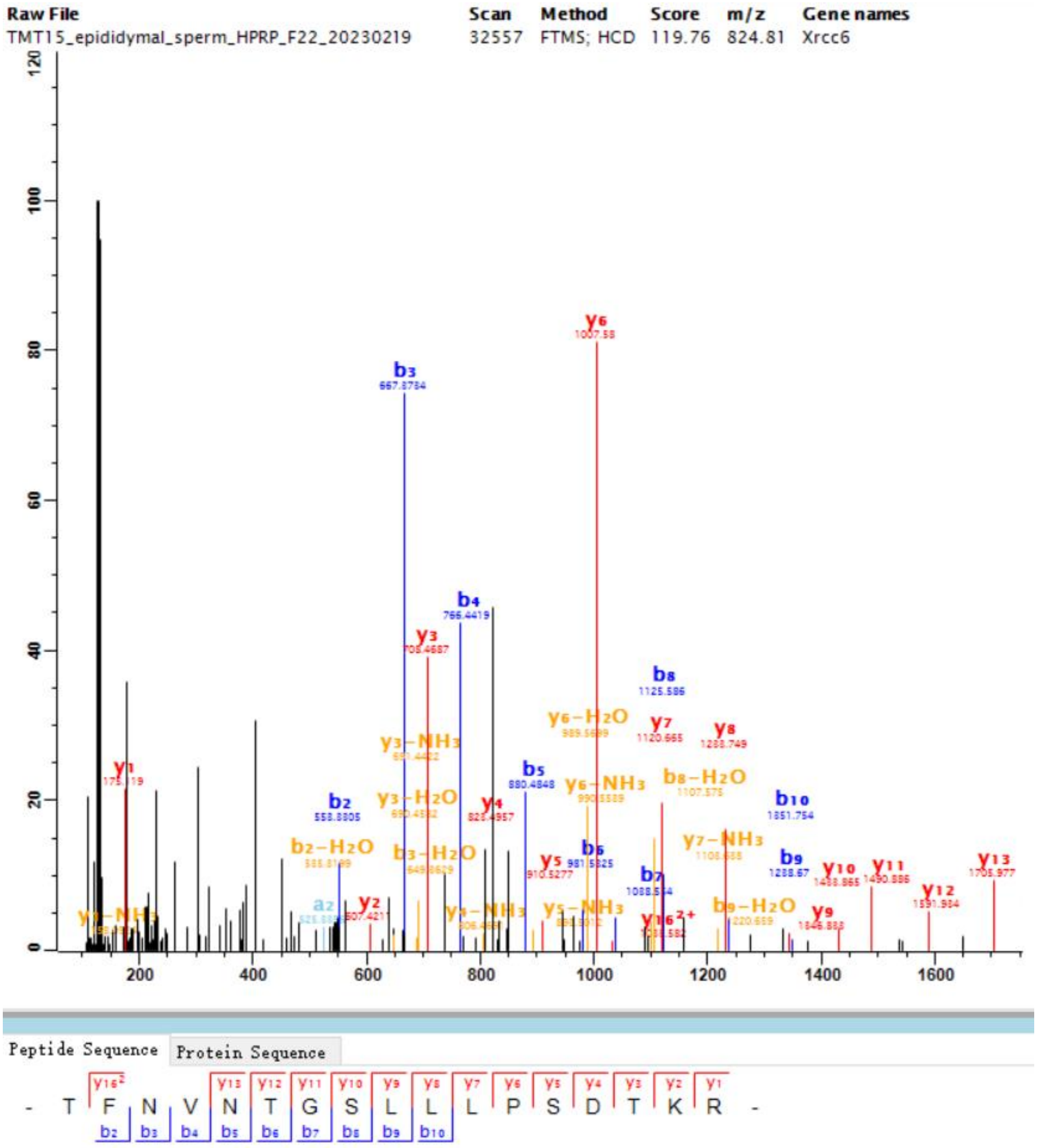

Raw File
TMT15_epididymal_sperm_HPRP_F22_20230219
Scan 32557
Method FTMS; HCD
Score 119.76
m/z 824.81
Gene names Xrcc6
Peptide Sequence
Protein Sequence
- T F N V N T G S L L L P S D T K R -

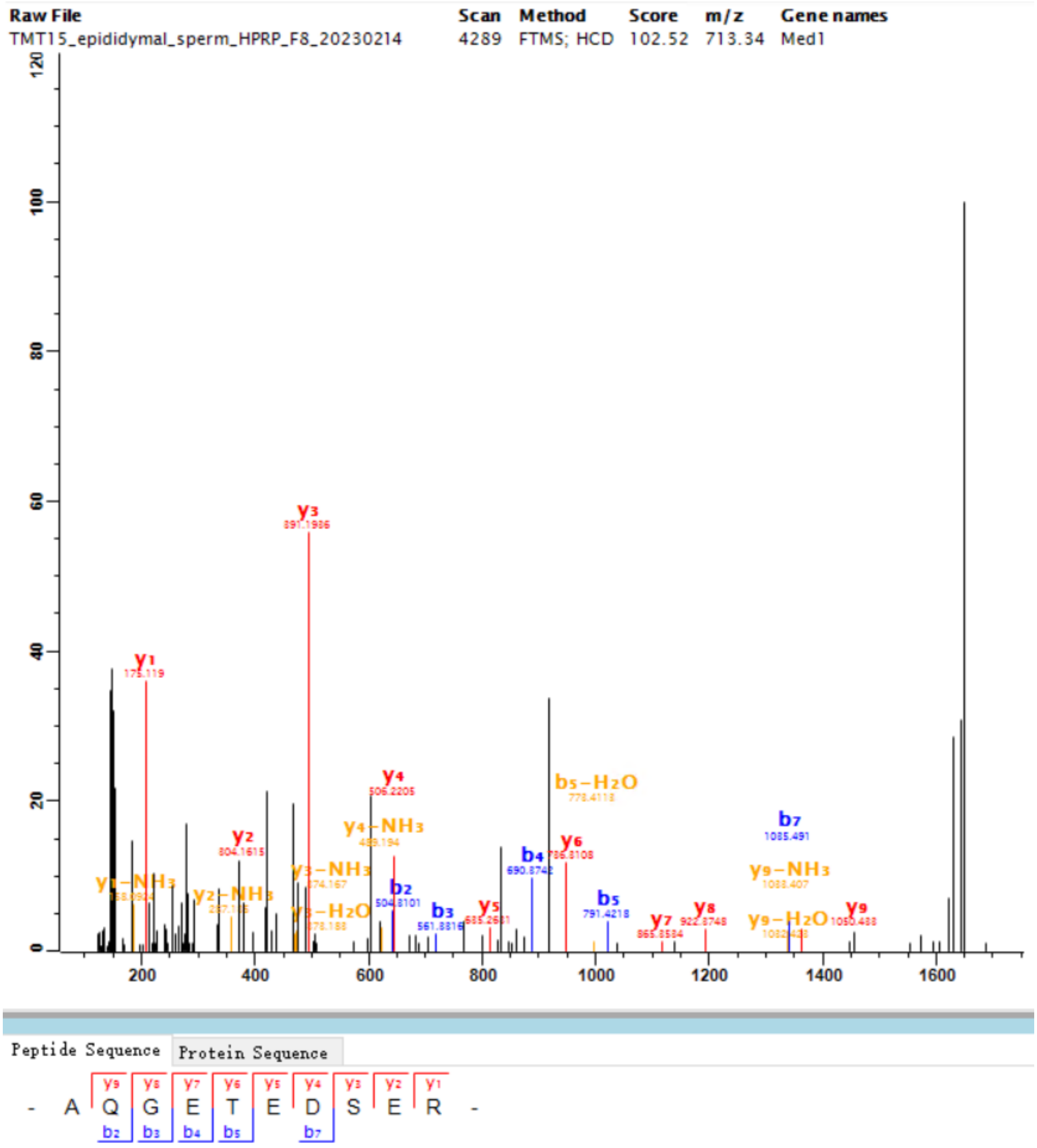
Raw File
TMT15_epididymal_sperm_HPRP_F8_20230214
Scan
4289
Method
FTMS; HCD
Score
102.52
m/z
713.34
Gene names
Med1
Peptide Sequence
Protein Sequence
- A Q G E T E D S E R -

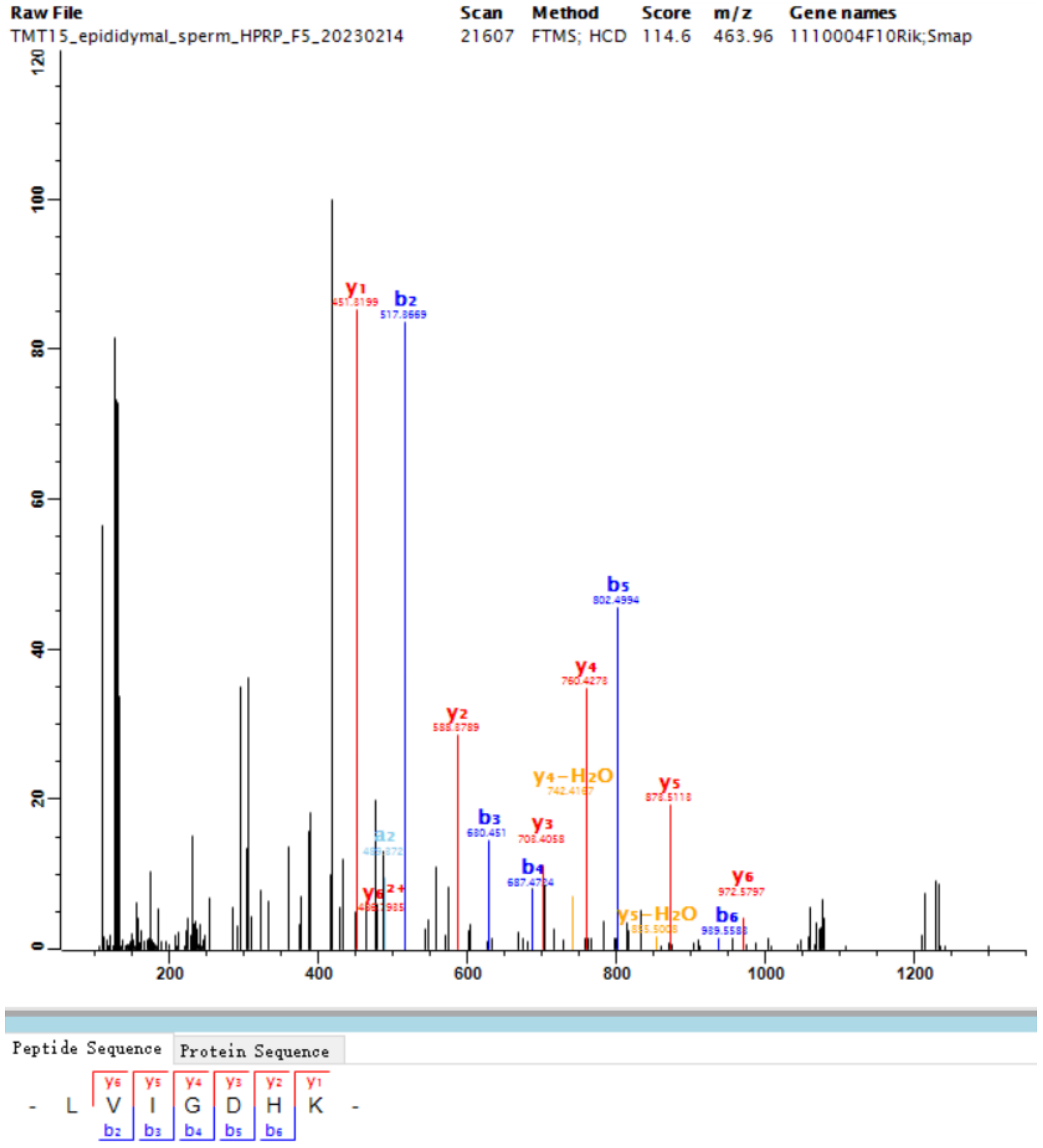

Raw File
TMT15_epididymal_sperm_HPRP_F5_20230214
Scan
21607
Method
FTMS; HCD
Score
114.6
m/z
463.96
Gene names
1110004F10Rik;Smap
y1
451.8199
b2
517.8669
b5
802.4994
y4
760.4278
y2
588.8789
y4−H2O
y5
b3
y3
b4
y6
b6
y5−H2O
Peptide Sequence
Protein Sequence
- L V I G D H K -

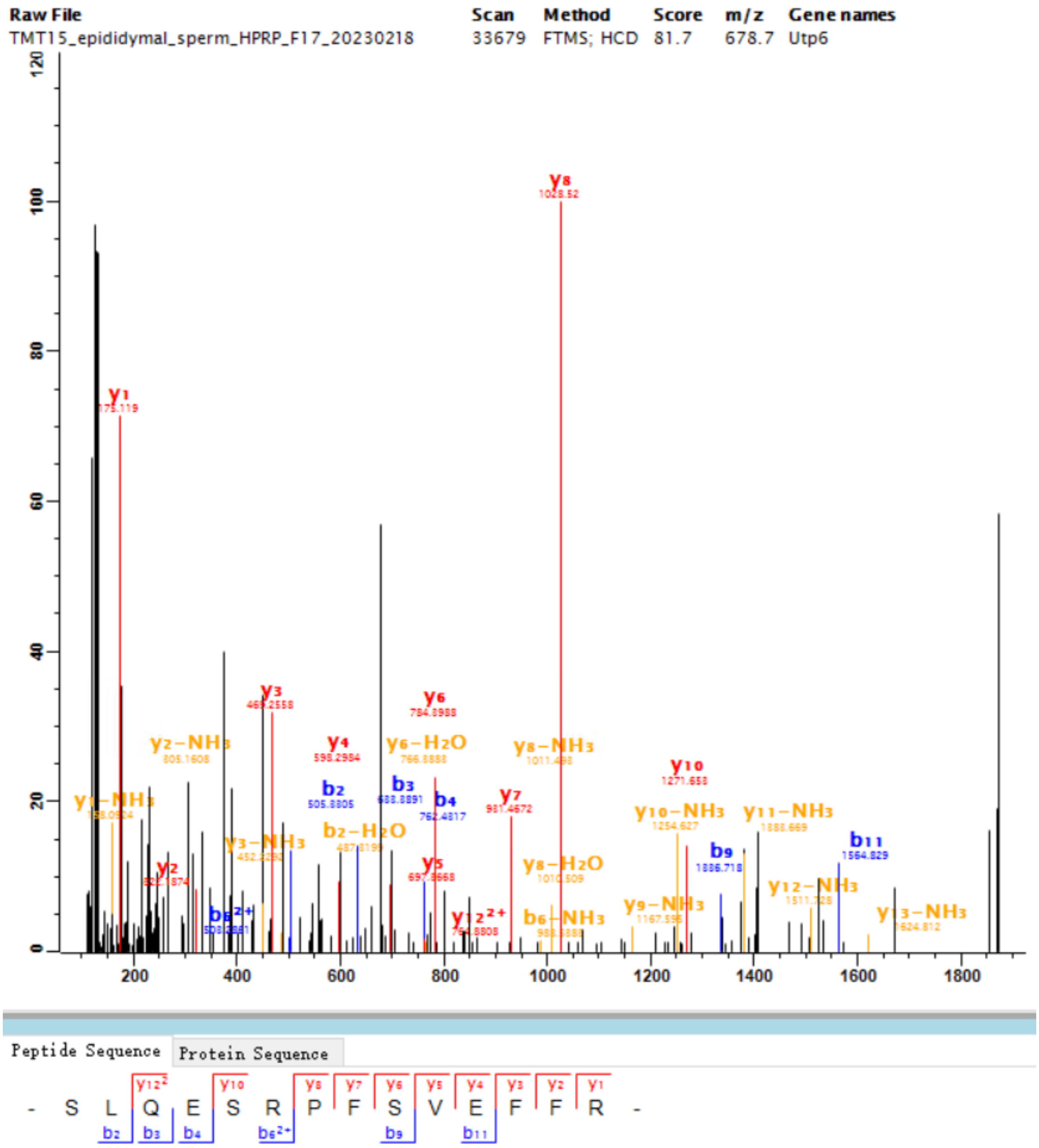

Raw File
TMT15_epididymal_sperm_HPRP_F17_20230218
Scan
33679
Method
FTMS; HCD
Score
81.7
m/z
678.7
Gene names
Utp6
Peptide Sequence
Protein Sequence
- S L Q E S R P F S V E F F R -

Raw File	Scan	Method	Score	m/z	Gene names
TMT15_epididymal_sperm_HPRP_F1_20230213	11349	FTMS; HCD	155.06	730.39	Pip4k2a

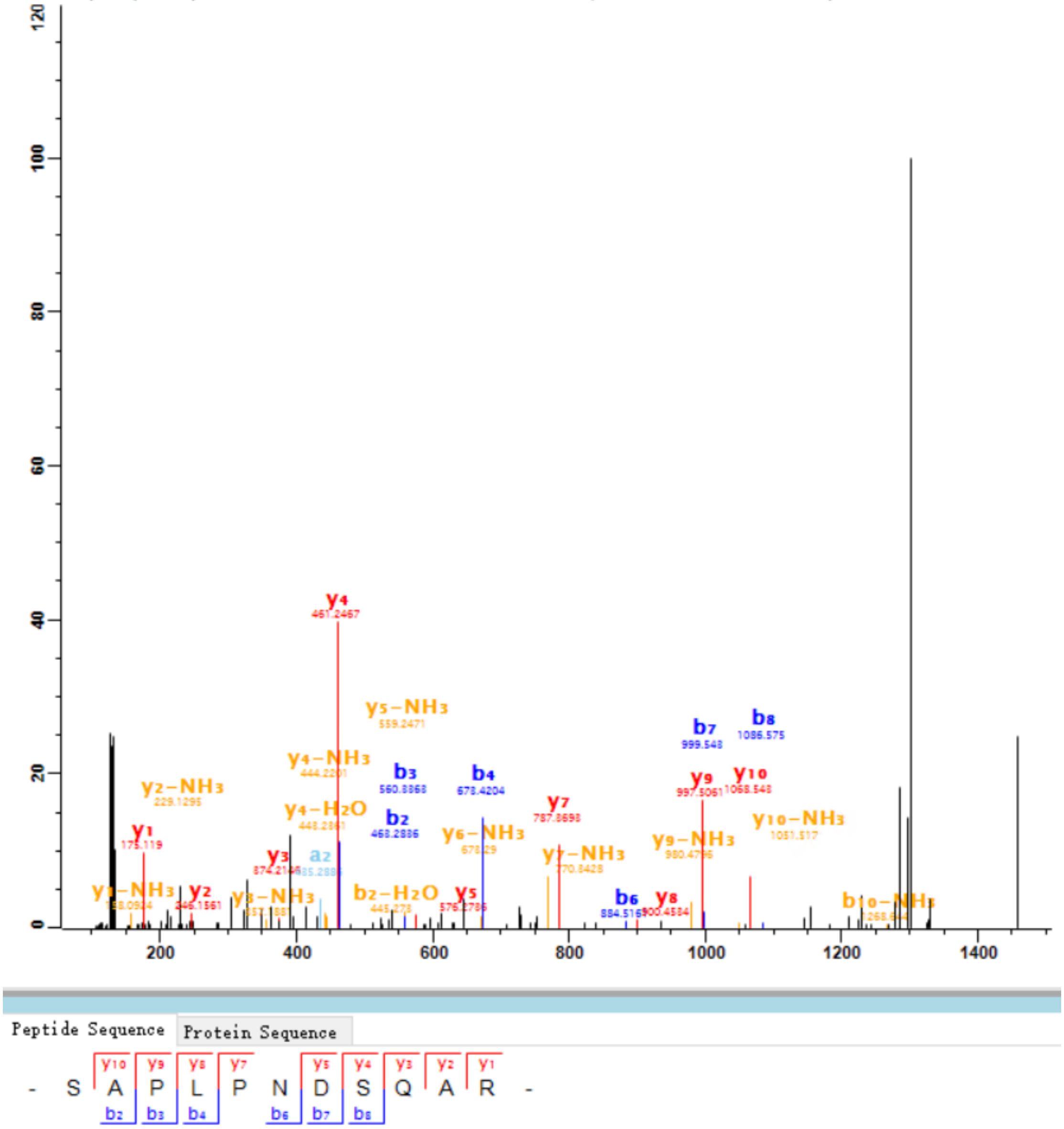

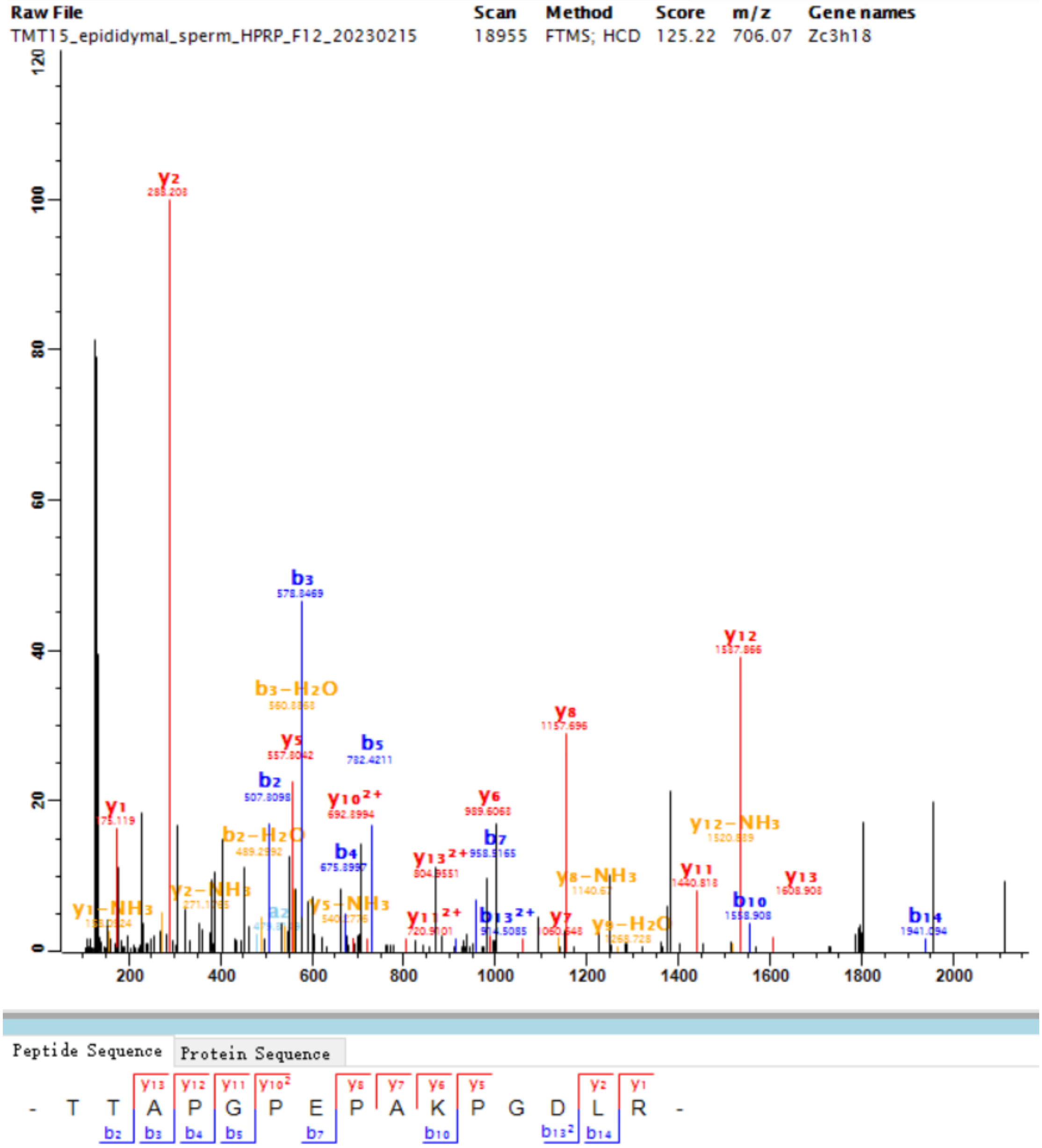

Raw File
TMT15_epididymal_sperm_HPRP_F12_20230215
Scan
18955
Method
FTMS; HCD
Score
125.22
m/z
706.07
Gene names
Zc3h18
y2
b3
578.8469
y12
b3-H2O
560.8868
y8
y5
557.8042
b5
782.4211
b2
507.8098
y10 2+
692.8994
y6
989.6068
y1
175.119
y12-NH3
b2-H2O
b7
958.5165
b4
675.8997
y13 2+
804.9551
y8-NH3
y11
y13
1608.908
y1-NH3
y2-NH3
y5-NH3
b10
1558.908
y11 2+
b13 2+
y7
y6-H2O
b14
1941.094
Peptide Sequence
Protein Sequence
- T T A P G P E P A K P G D L R -

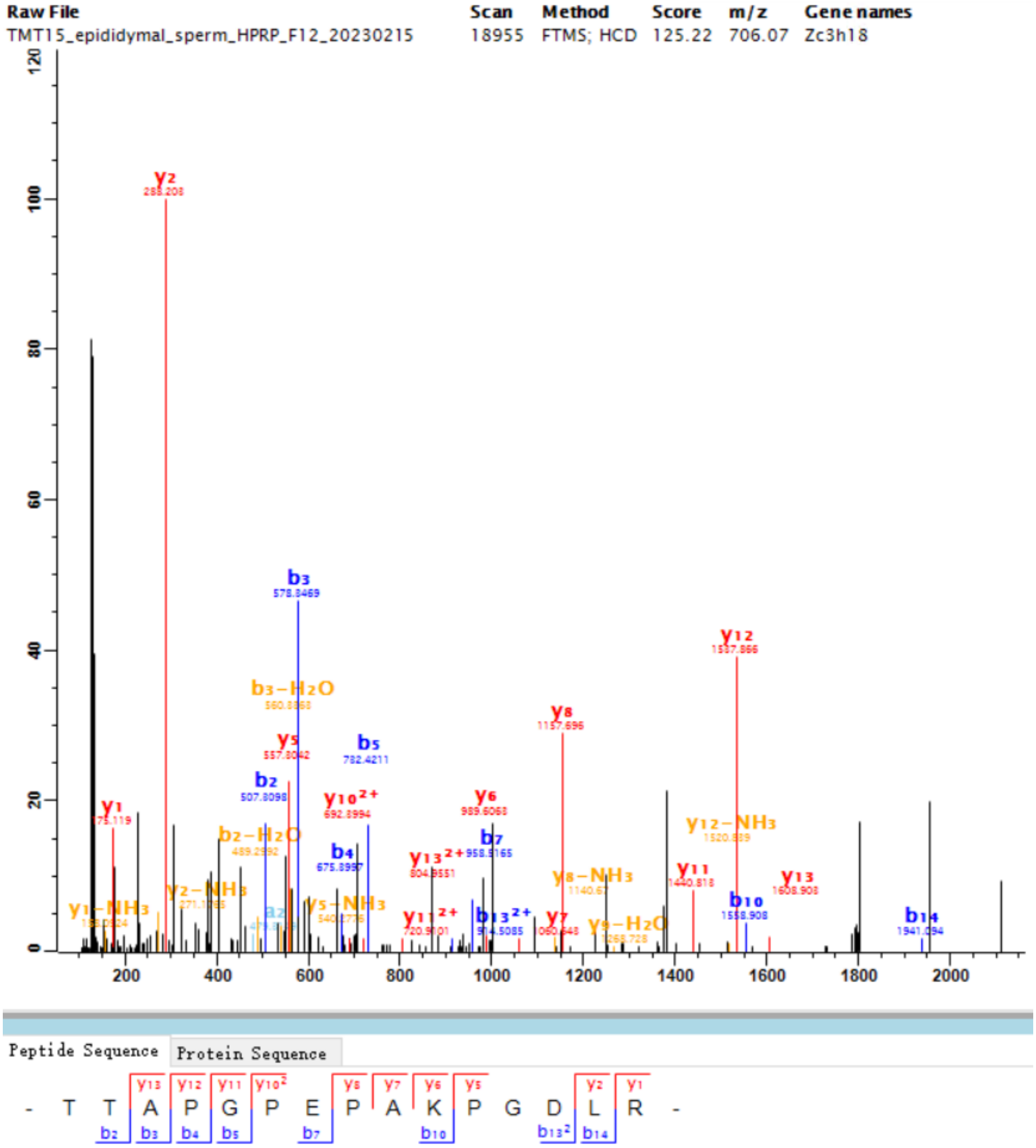
Raw File
TMT15_epididymal_sperm_HPRP_F12_20230215
Scan
18955
Method
FTMS; HCD
Score
125.22
m/z
706.07
Gene names
Zc3h18
y2
b3 578.8469
y12
b3-H2O 560.8363
y8 1157.696
y5 557.8042
b5 782.4211
b2 507.8098
y10 2+ 692.8994
y6 989.6063
y1 175.119
b2-H2O 489.2992
b7 958.5165
b4 675.8997
y13 2+ 804.9551
y12-NH3 1520.889
y8-NH3 1140.67
y11 1440.818
y13 1608.908
y1-NH3
y2-NH3 271.1785
y5-NH3
b10 1558.908
b14 1941.094
y11 2+ 720.9101
b13 2+ 974.5085
y7
200
400
600
800
1000
1200
1400
1600
1800
2000
0
20
40
60
80
100
120
Peptide Sequence
Protein Sequence
- T T A P G P E P A K P G D L R -

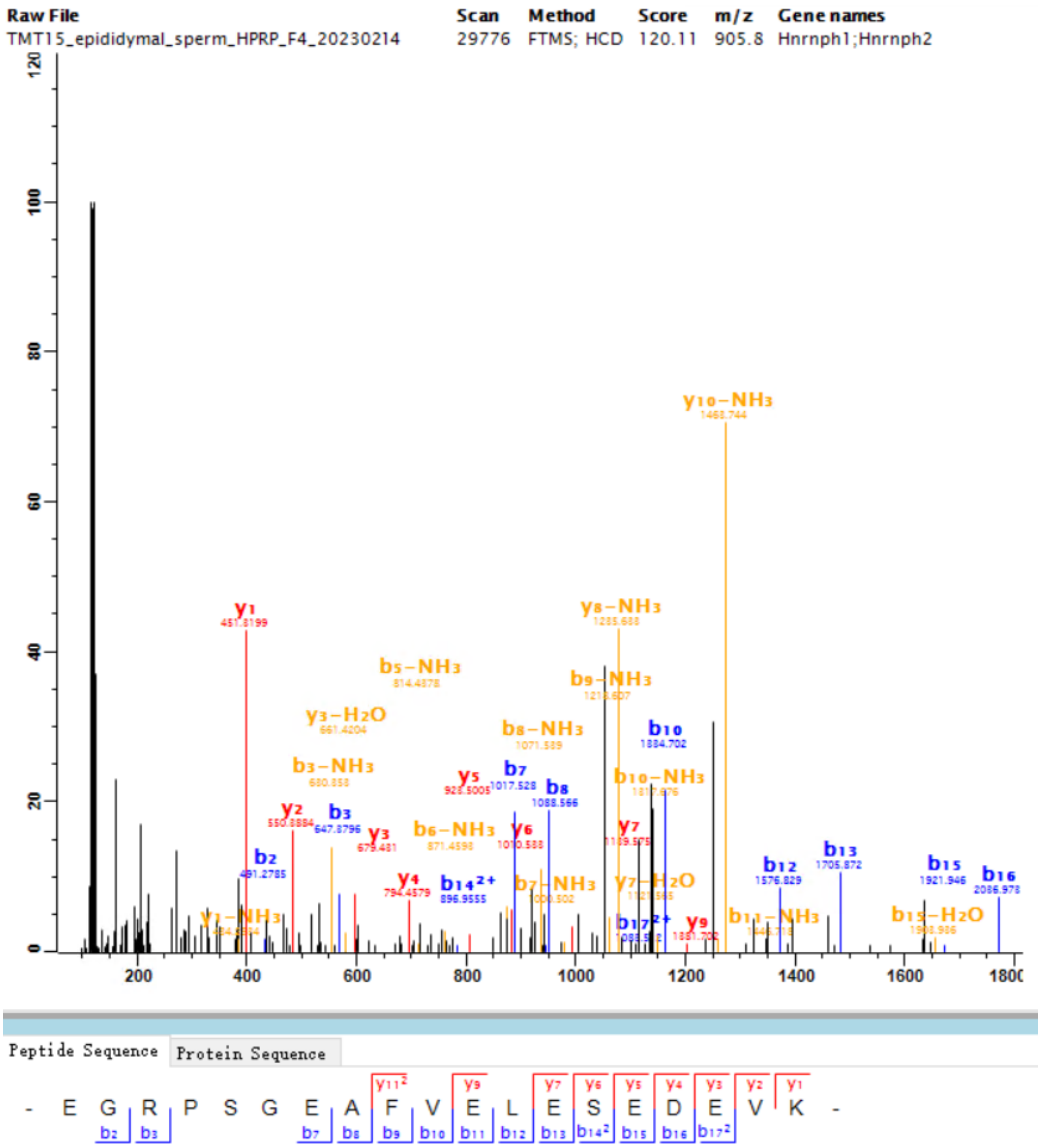

Raw File
TMT15_epididymal_sperm_HPRP_F4_20230214
Scan Method Score m/z Gene names
29776 FTMS; HCD 120.11 905.8 Hnrnph1;Hnrnph2
y1 451.8199
y10-NH3 1468.744
y8-NH3 1285.686
b5-NH3 814.4878
b9-NH3
y3-H2O 661.4204
b8-NH3 1071.589
b10 1384.702
b3-NH3 680.858
b7 1017.528
b8 1088.566
b10-NH3
y5
y2 550.8884
b3 647.8796
y3 679.481
b6-NH3 871.4598
y6 1010.588
y7
b2 491.2785
b12 1576.829
b13 1705.872
b15 1921.946
b16 2036.978
y4 794.4579
b14 2+ 896.9555
b7-NH3
y7-H2O
b17 2+
y9
b11-NH3
b15-H2O
200 400 600 800 1000 1200 1400 1600
0 20 40 60 80 100 120
Peptide Sequence
Protein Sequence
- E G R P S G E A F V E L E S E D E V K -

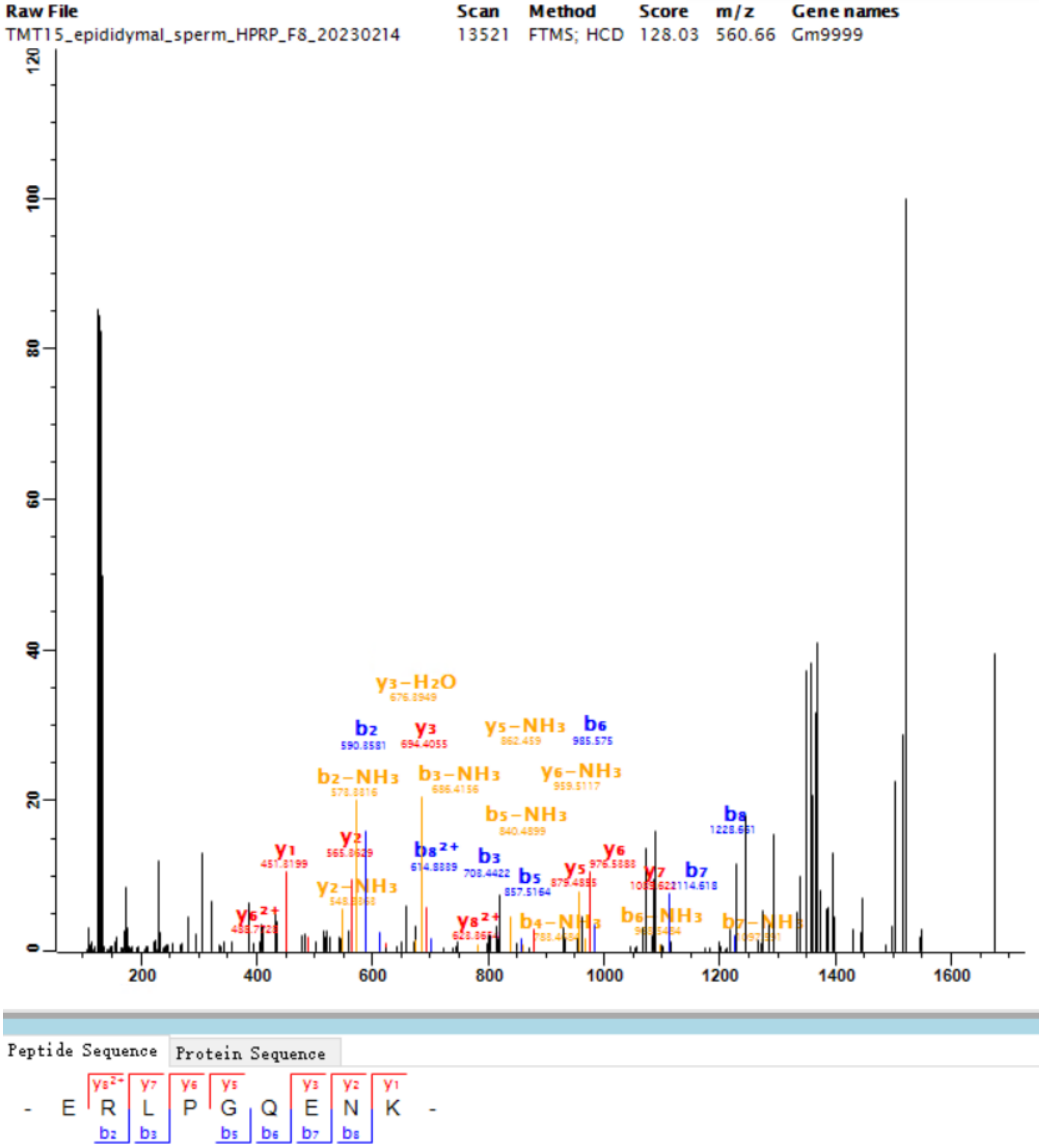
Raw File
TMT15_epididymal_sperm_HPRP_F8_20230214
Scan
13521
Method
FTMS; HCD
Score
128.03
m/z
560.66
Gene names
Gm9999
Peptide Sequence
Protein Sequence
- E R L P G Q E N K -

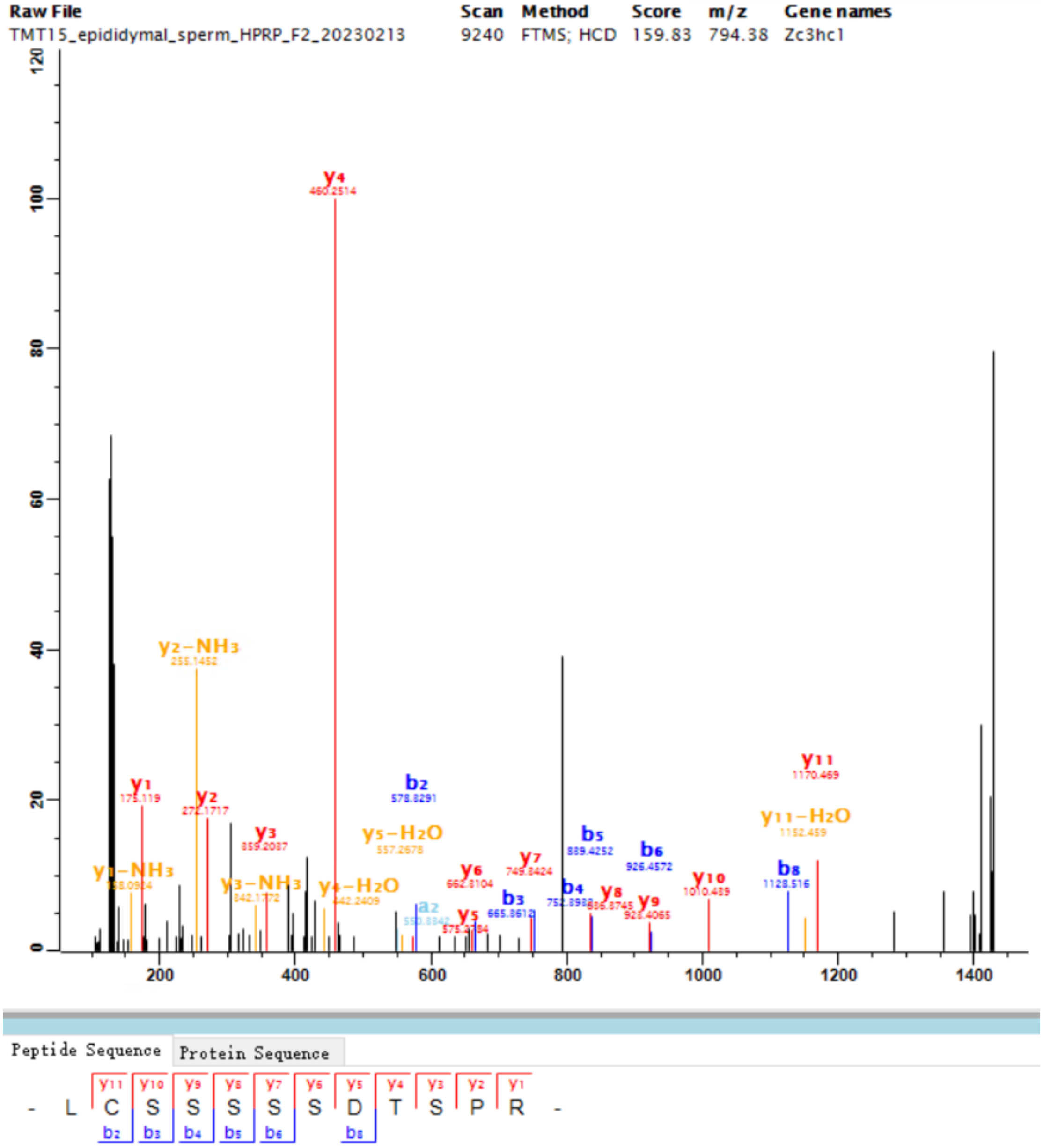
Raw File
TMT15_epididymal_sperm_HPRP_F2_20230213
Scan Method Score m/z Gene names
9240 FTMS; HCD 159.83 794.38 Zc3hc1
y4 460.2514
y2-NH3 255.1452
y1 175.119
y2 272.1717
y3 359.2037
b2 578.8291
y5-H2O 557.2678
y11 1170.489
y11-H2O 1152.459
b5 889.4252
b6 926.4572
y7 749.3424
y6 662.3104
y10 1010.489
b8 1128.516
y3-NH3 342.1772
y4-H2O 442.2409
b3 665.8612
b4 752.8932
y8
y9
y5
a2
Peptide Sequence
Protein Sequence
y11 y10 y9 y8 y7 y6 y5 y4 y3 y2 y1
- L C S S S S S D T S P R -
b2 b3 b4 b5 b6 b8

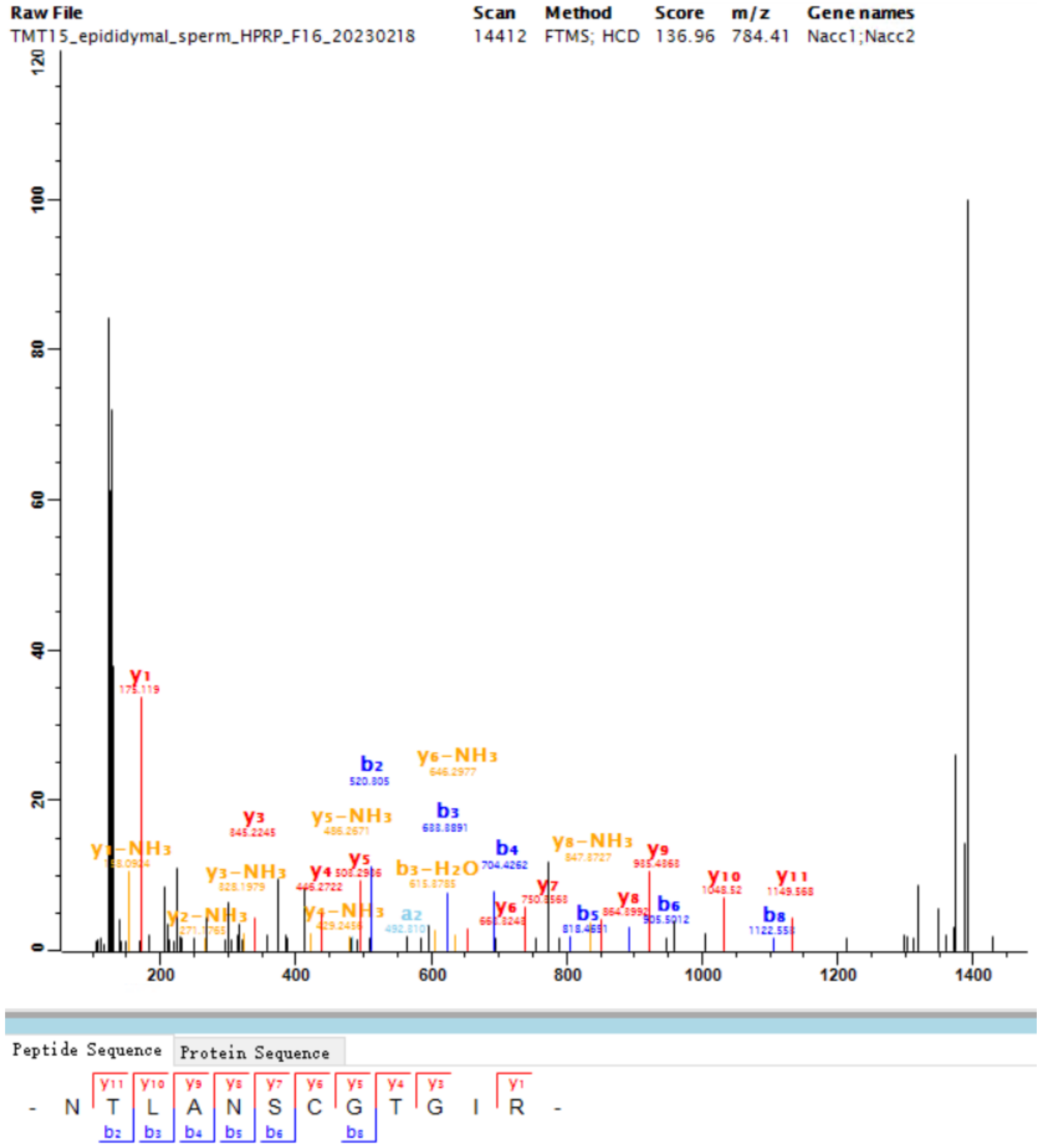
Raw File
TMT15_epididymal_sperm_HPRP_F16_20230218
Scan 14412
Method FTMS; HCD
Score 136.96
m/z 784.41
Gene names Nacc1;Nacc2
y1 175.119
y2-NH3
y3 345.2245
y3-NH3 328.1979
y4 446.2722
y4-NH3 429.2459
y5 503.2936
y5-NH3 486.2671
y6-NH3 646.2977
y6
y7 750.3568
y8 864.3990
y8-NH3 847.3727
y9 935.4368
y10 1048.52
y11 1149.568
b2 520.805
b3 633.8891
b3-H2O 615.8785
b4 704.4262
b5 818.4651
b6 905.5012
b8 1122.558
a2 492.810
Peptide Sequence
Protein Sequence
- N T L A N S C G T G I R -

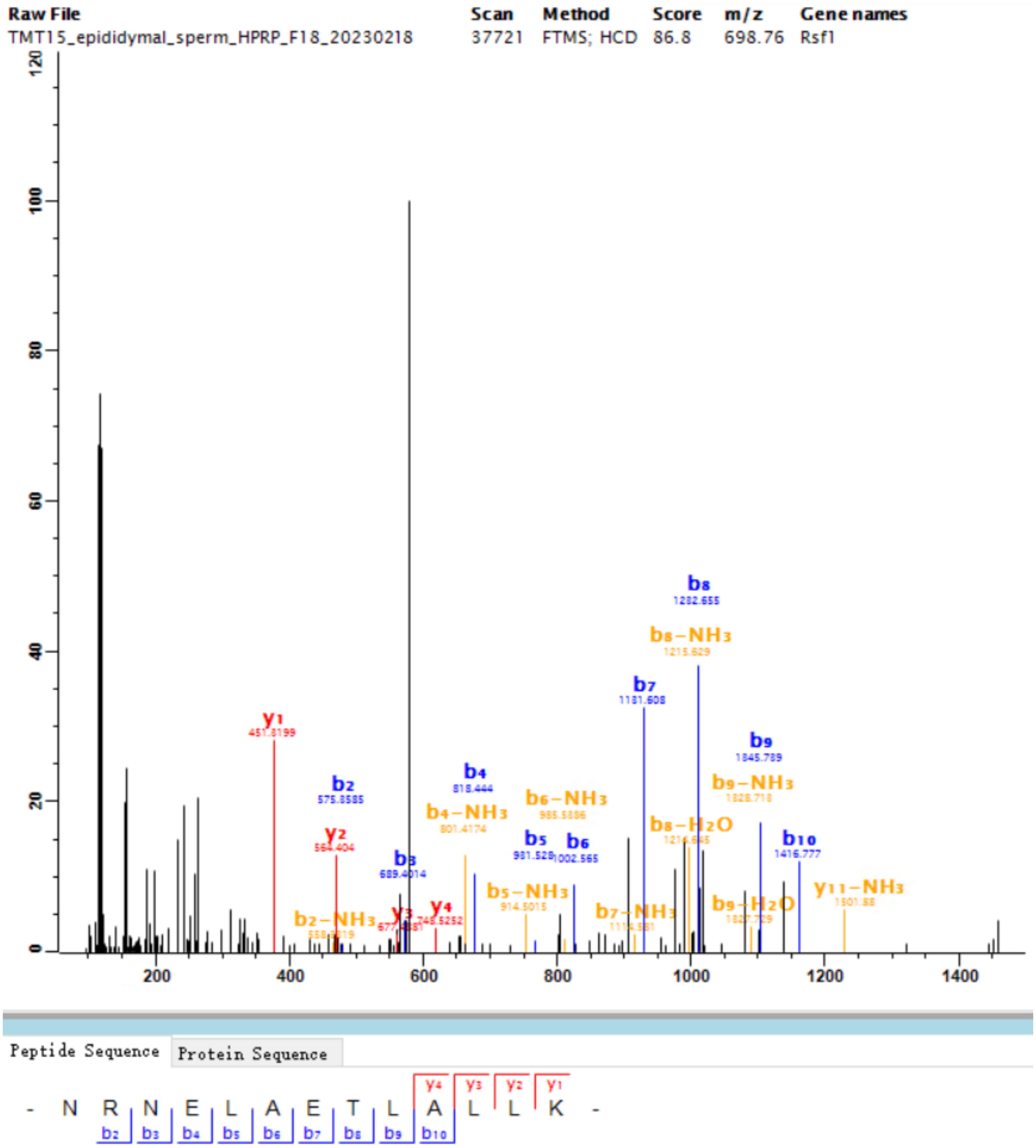
Raw File
TMT15_epididymal_sperm_HPRP_F18_20230218
Scan
37721
Method
FTMS; HCD
Score
86.8
m/z
698.76
Gene names
Rsf1
y1
451.8199
b2
575.8585
y2
564.404
b3
689.4014
b4
818.444
b4-NH3
801.4174
b5
931.529
b6
1002.565
b6-NH3
985.5886
b5-NH3
914.5015
b7
1131.608
b8
1232.655
b8-NH3
1215.629
b8-H2O
b9
1345.789
b9-NH3
1328.718
b9-H2O
b10
1416.777
y11-NH3
1501.88
b2-NH3
y3
y4
748.5252
b7-NH3
Peptide Sequence
Protein Sequence
- N R N E L A E T L A L L K -

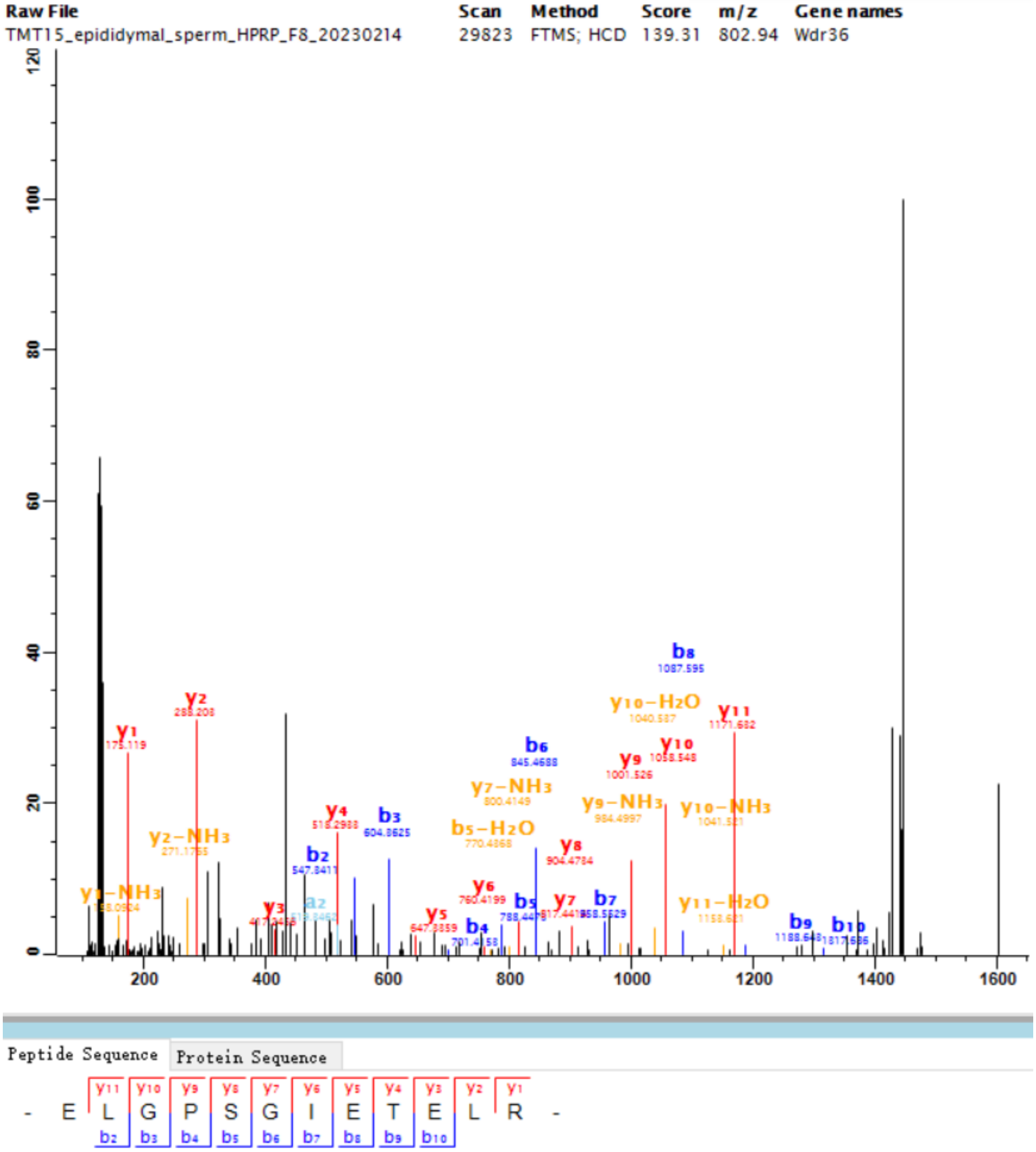
Raw File
TMT15_epididymal_sperm_HPRP_F8_20230214
Scan
29823
Method
FTMS; HCD
Score
139.31
m/z
802.94
Gene names
Wdr36
y1 175.119
y2 288.203
y2-NH3 271.1755
y4 518.2988
b2 547.8411
b3 604.8625
b5-H2O 770.4868
y7-NH3 800.4149
b6 845.4688
y8 904.4784
y9 1001.526
y9-NH3 984.4997
y10 1058.548
y10-H2O 1040.537
b8 1087.595
y11 1171.632
y6 760.4199
b7 958.5529
Peptide Sequence
Protein Sequence
- E L G P S G I E T E L R -

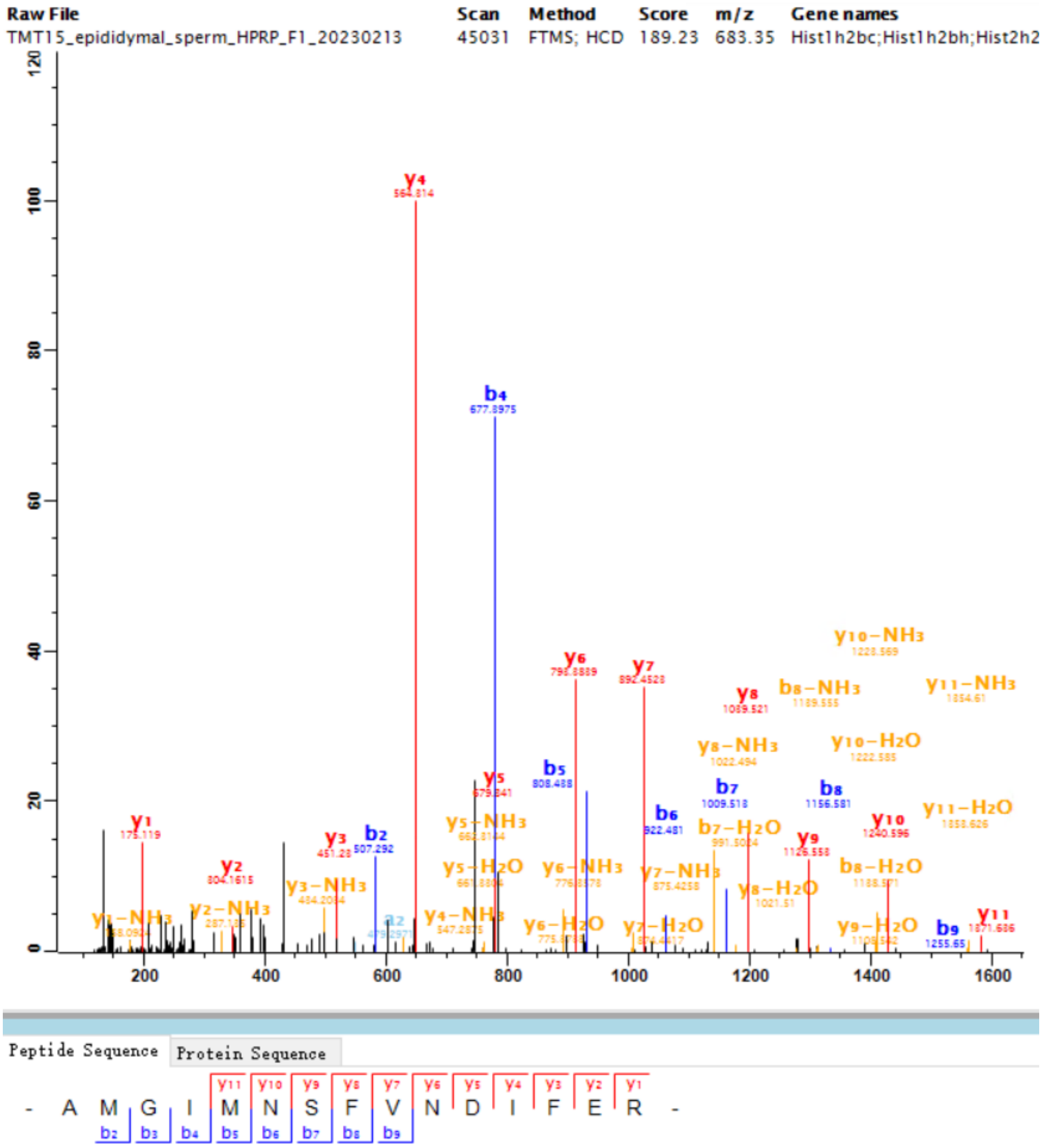
Raw File
TMT15_epididymal_sperm_HPRP_F1_20230213
Scan
45031
Method
FTMS; HCD
Score
189.23
m/z
683.35
Gene names
Hist1h2bc;Hist1h2bh;Hist2h2
y4
b4
677.8975
y6
y7
y8
y5
b5
b6
b7
b8
y9
y10
y11
b2
b9
y1
y2
y3
y10−NH3
y11−NH3
b8−NH3
y8−NH3
y10−H2O
y11−H2O
b7−H2O
b8−H2O
y5−NH3
y5−H2O
y6−NH3
y7−NH3
y8−H2O
y3−NH3
y2−NH3
y1−NH3
y4−NH3
y6−H2O
y7−H2O
y9−H2O
a2
200
400
600
800
1000
1200
1400
1600
Peptide Sequence
Protein Sequence
- A M G I M N S F V N D I F E R -

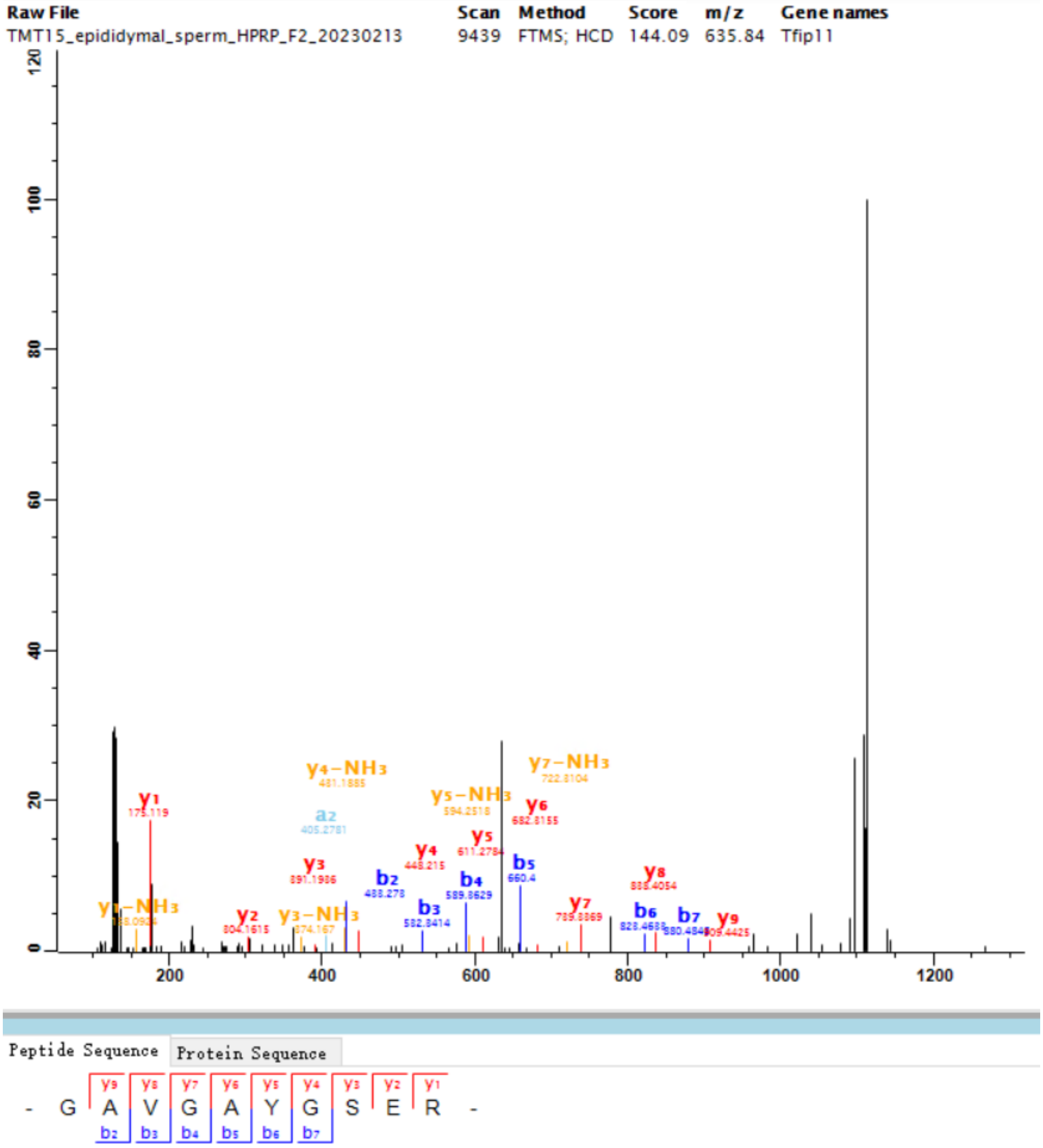
Raw File
TMT15_epididymal_sperm_HPRP_F2_20230213
Scan
9439
Method
FTMS; HCD
Score
144.09
m/z
635.84
Gene names
Tfip11
y1
y2
y3
y4
y5
y6
y7
y8
y9
b2
b3
b4
b5
b6
b7
a2
y3−NH3
y4−NH3
y5−NH3
y7−NH3
0
20
40
60
80
100
120
200
400
600
800
1000
1200
Peptide Sequence
Protein Sequence
- G A V G A Y G S E R -

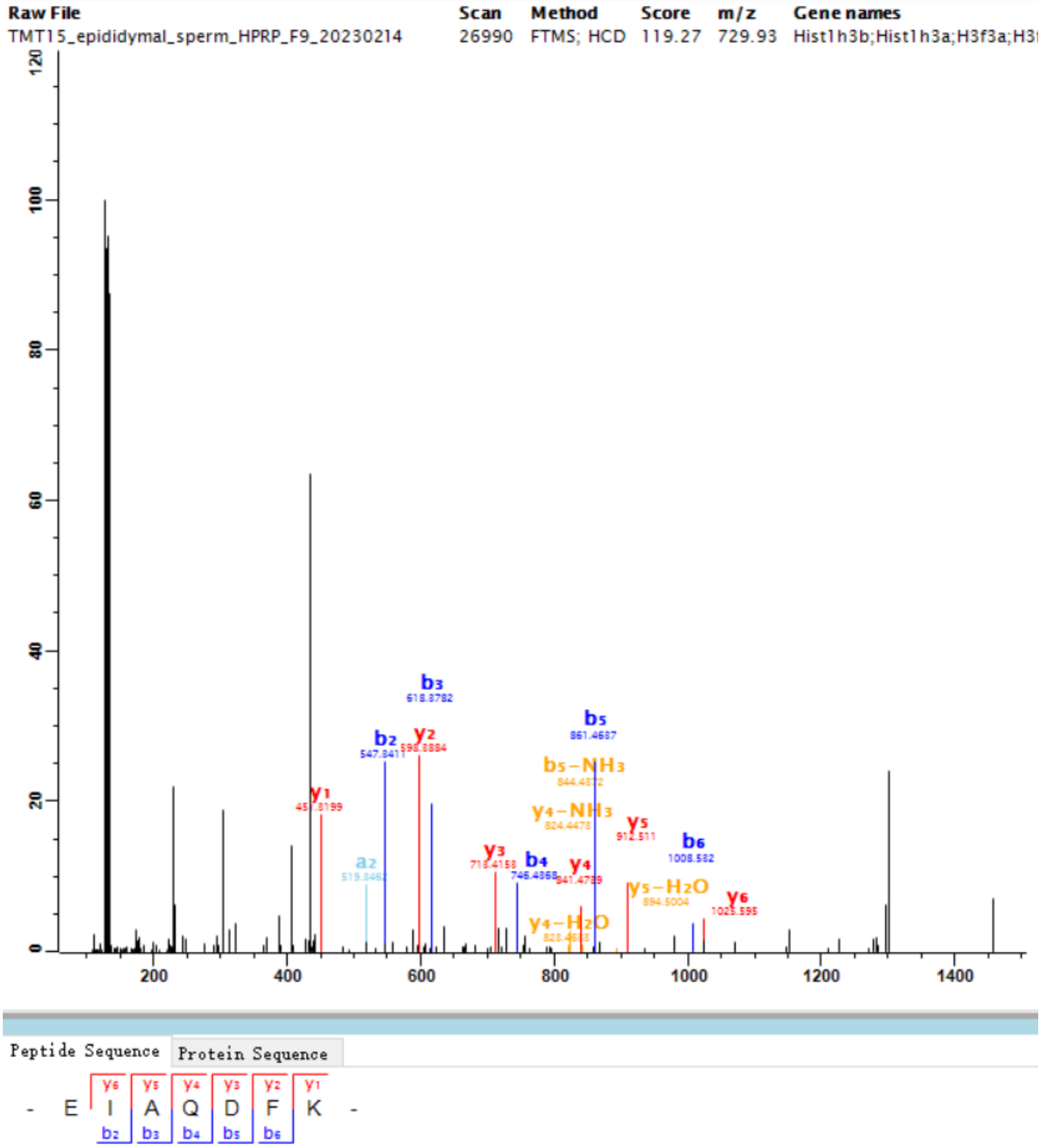

Raw File
TMT15_epididymal_sperm_HPRP_F9_20230214
Scan
26990
Method
FTMS; HCD
Score
119.27
m/z
729.93
Gene names
Hist1h3b;Hist1h3a;H3f3a;H3
0
20
40
60
80
100
120
200
400
600
800
1000
1200
1400
b3
618.8782
b2
547.8411
y2
598.8884
b5
861.4687
b5-NH3
844.4872
y1
y4-NH3
824.4478
y5
912.511
a2
y3
b4
746.4868
y4
841.4789
b6
1008.582
y5-H2O
894.5004
y6
1025.595
y4-H2O
Peptide Sequence
Protein Sequence
- E I A Q D F K -
y6 y5 y4 y3 y2 y1
b2 b3 b4 b5 b6

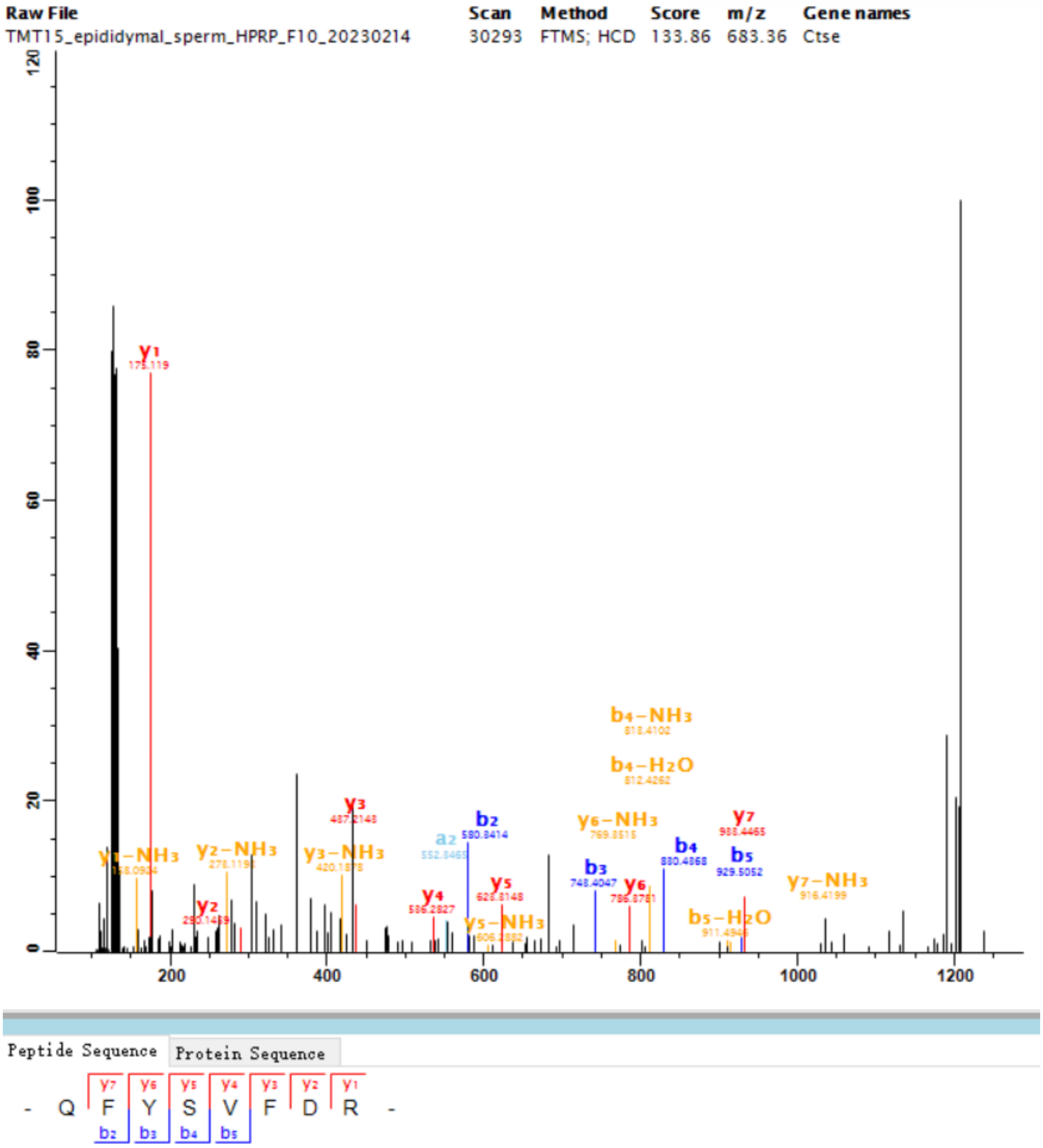
Raw File
TMT15_epididymal_sperm_HPRP_F10_20230214
Scan
30293
Method
FTMS; HCD
Score
133.86
m/z
683.36
Gene names
Ctse
y1
y2
y3
y4
y5
y6
y7
b2
b3
b4
b5
a2
y1-NH3
y2-NH3
y3-NH3
y5-NH3
y6-NH3
y7-NH3
b4-NH3
b4-H2O
b5-H2O
Peptide Sequence
Protein Sequence
- Q F Y S V F D R -

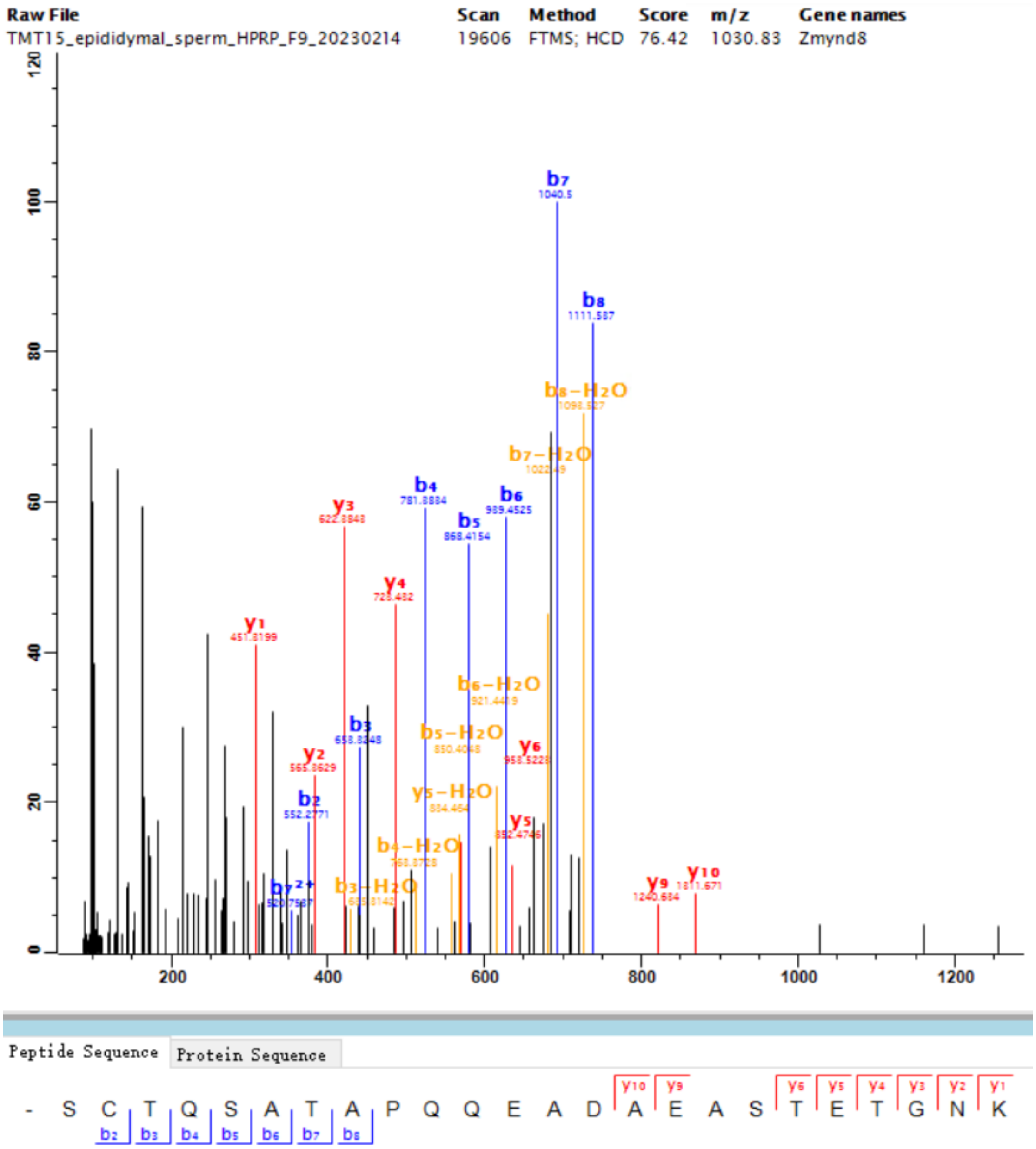
Raw File
TMT15_epididymal_sperm_HPRP_F9_20230214
Scan
19606
Method
FTMS; HCD
Score
76.42
m/z
1030.83
Gene names
Zmynd8
b7
1040.5
b8
1111.587
b8-H2O
b7-H2O
b4
781.8884
b6
989.4525
b5
868.4154
y3
622.8848
y4
y1
b6-H2O
b3
b5-H2O
y6
y2
b2
552.2771
y5-H2O
y5
b4-H2O
b7²⁺
b3-H2O
y9
y10
Peptide Sequence
Protein Sequence
- S C T Q S A T A P Q Q E A D A E A S T E T G N K

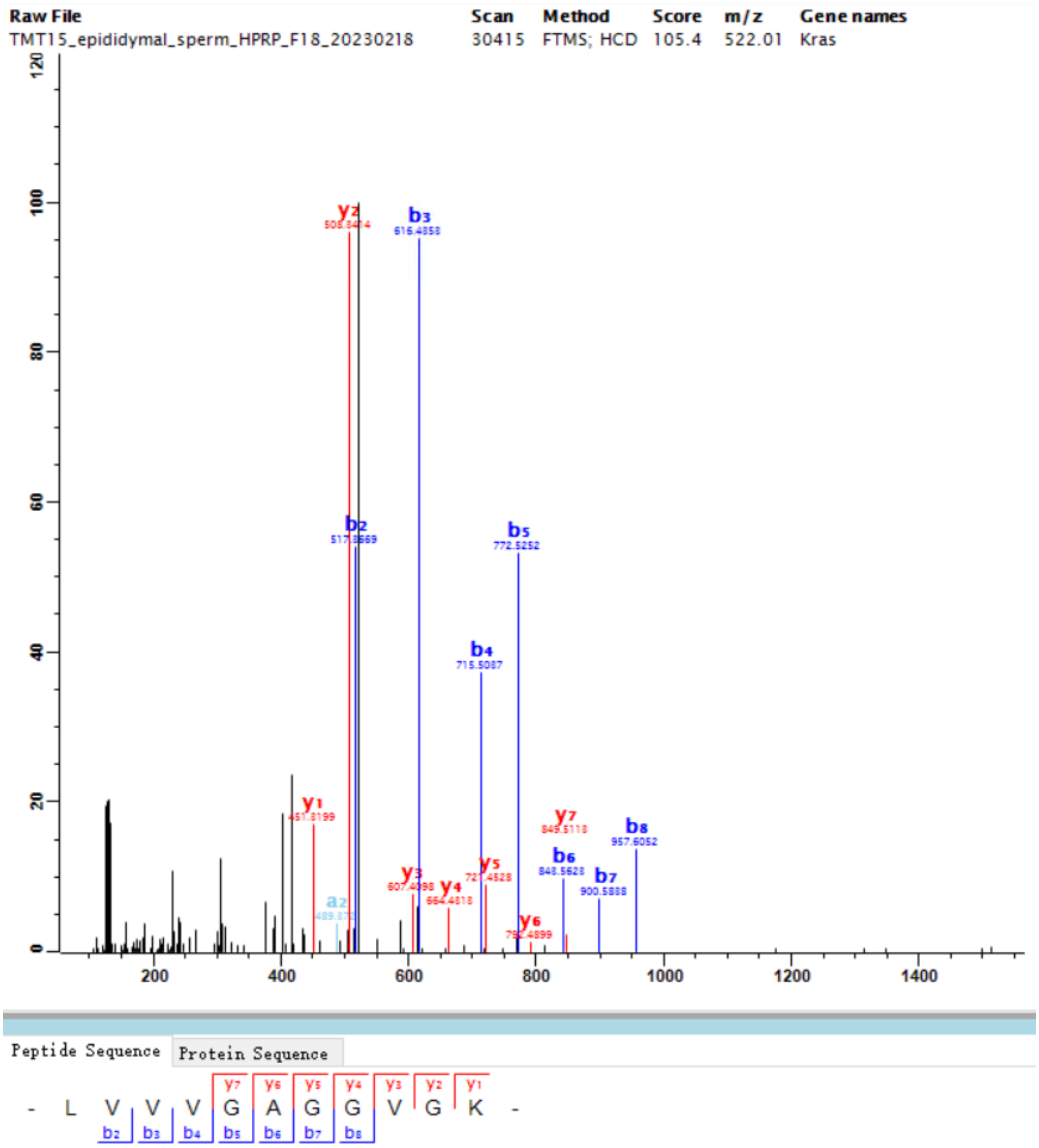

Raw File
TMT15_epididymal_sperm_HPRP_F18_20230218
Scan
30415
Method
FTMS; HCD
Score
105.4
m/z
522.01
Gene names
Kras
y2
b3
616.4358
b2
b5
772.5252
b4
715.5087
y1
y7
b8
957.6052
b6
848.5628
b7
900.5888
y3
y4
y5
a2
y6
Peptide Sequence
Protein Sequence
- L V V V G A G G V G K -

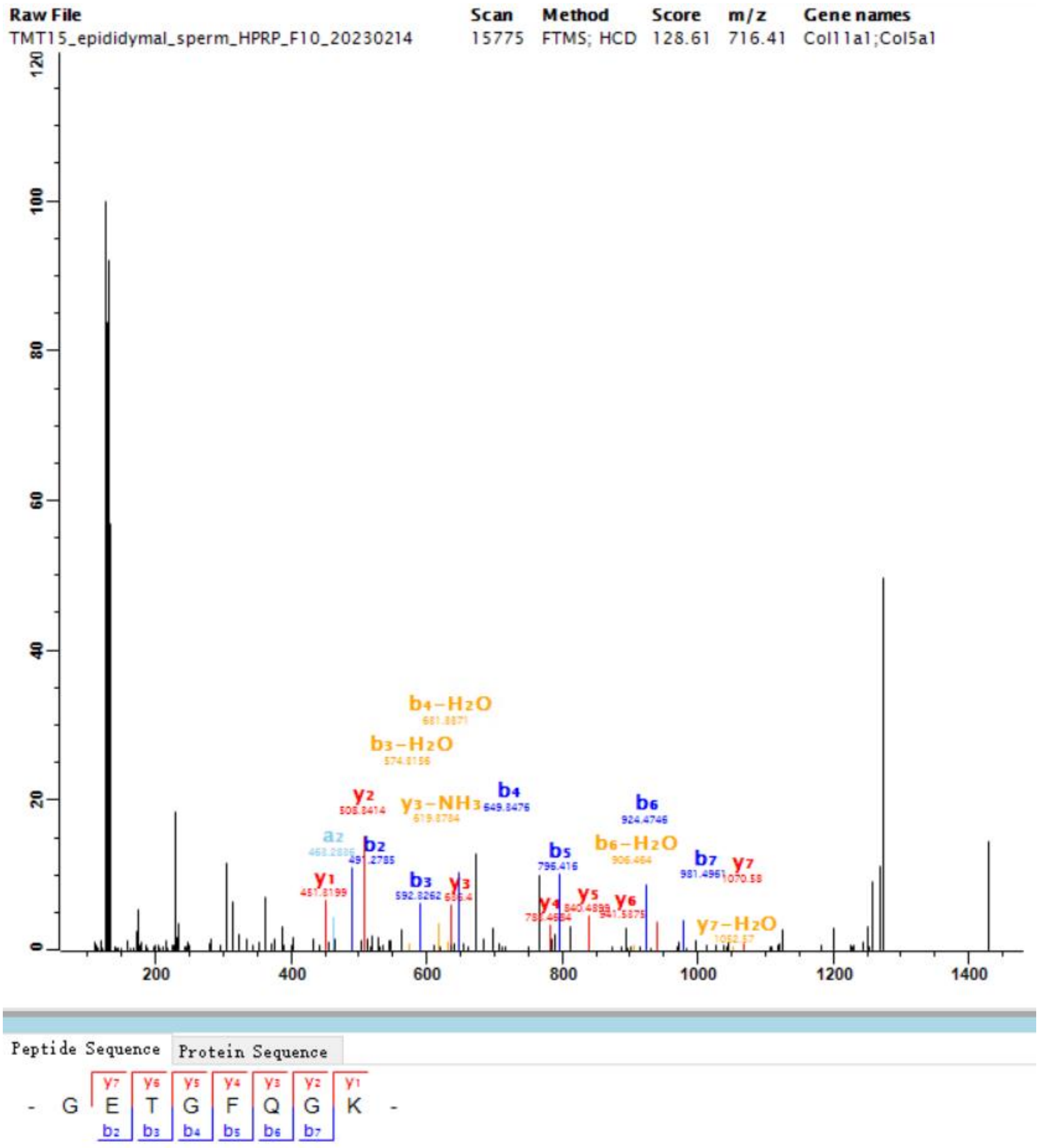
Raw File
TMT15_epididymal_sperm_HPRP_F10_20230214
Scan
15775
Method
FTMS; HCD
Score
128.61
m/z
716.41
Gene names
Col11a1;Col5a1
b4-H2O
b3-H2O
y2
y3-NH3
b4
b6
a2
b2
b5
b6-H2O
b7
y7
y1
b3
y3
y4
y5
y6
y7-H2O
Peptide Sequence
Protein Sequence
- G E T G F Q G K -

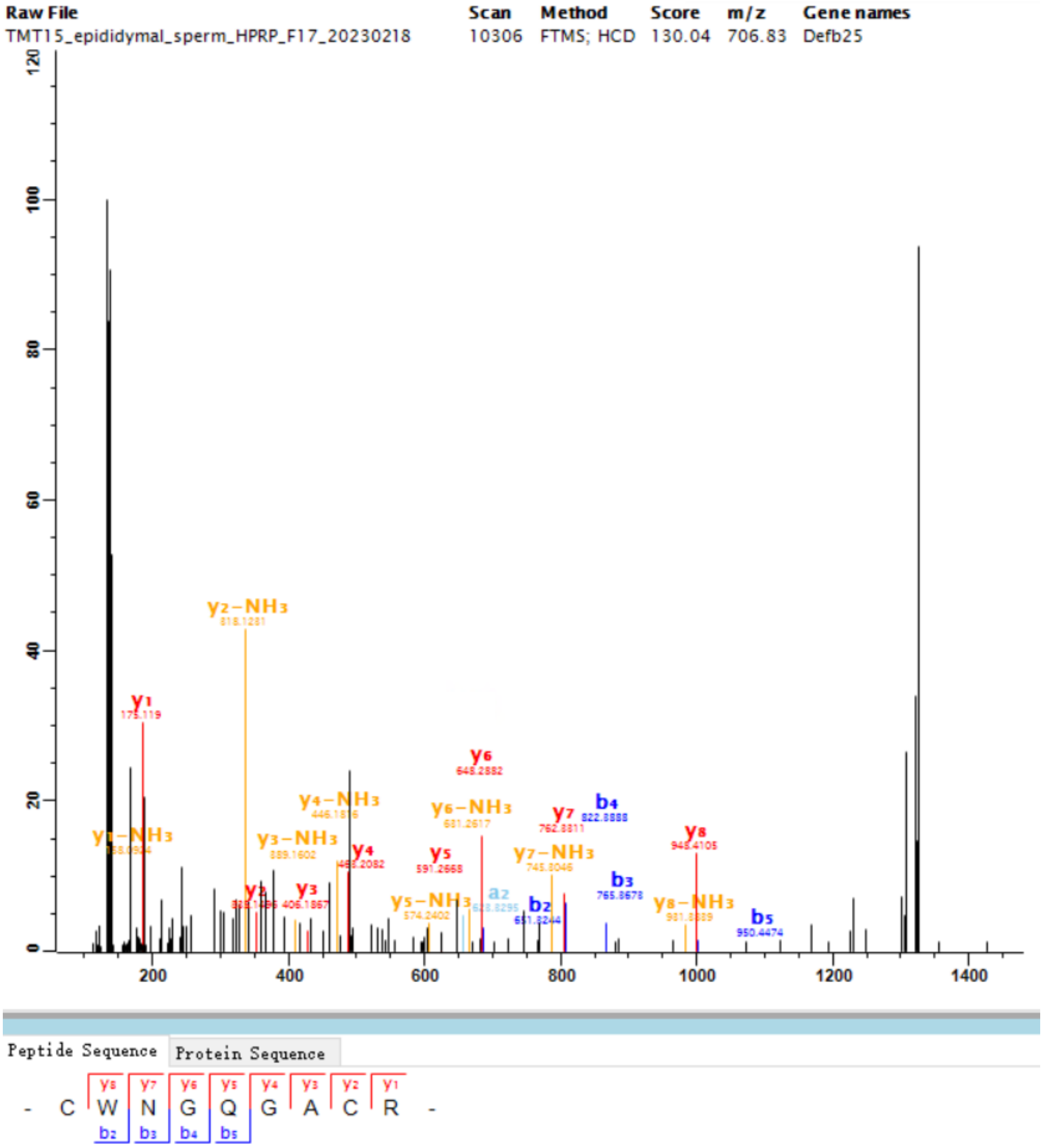

Raw File
TMT15_epididymal_sperm_HPRP_F17_20230218
Scan
10306
Method
FTMS; HCD
Score
130.04
m/z
706.83
Gene names
Defb25
y1
175.119
y2-NH3
y3-NH3
389.1602
y4-NH3
446.1816
y4
y3
406.1867
y5
591.2668
y5-NH3
574.2402
y6
648.2882
y6-NH3
631.2617
y7
762.3311
y7-NH3
745.3046
y8
948.4105
y8-NH3
931.3839
a2
b2
b3
765.3678
b4
822.3888
b5
950.4474
Peptide Sequence
Protein Sequence
- C W N G Q G A C R -

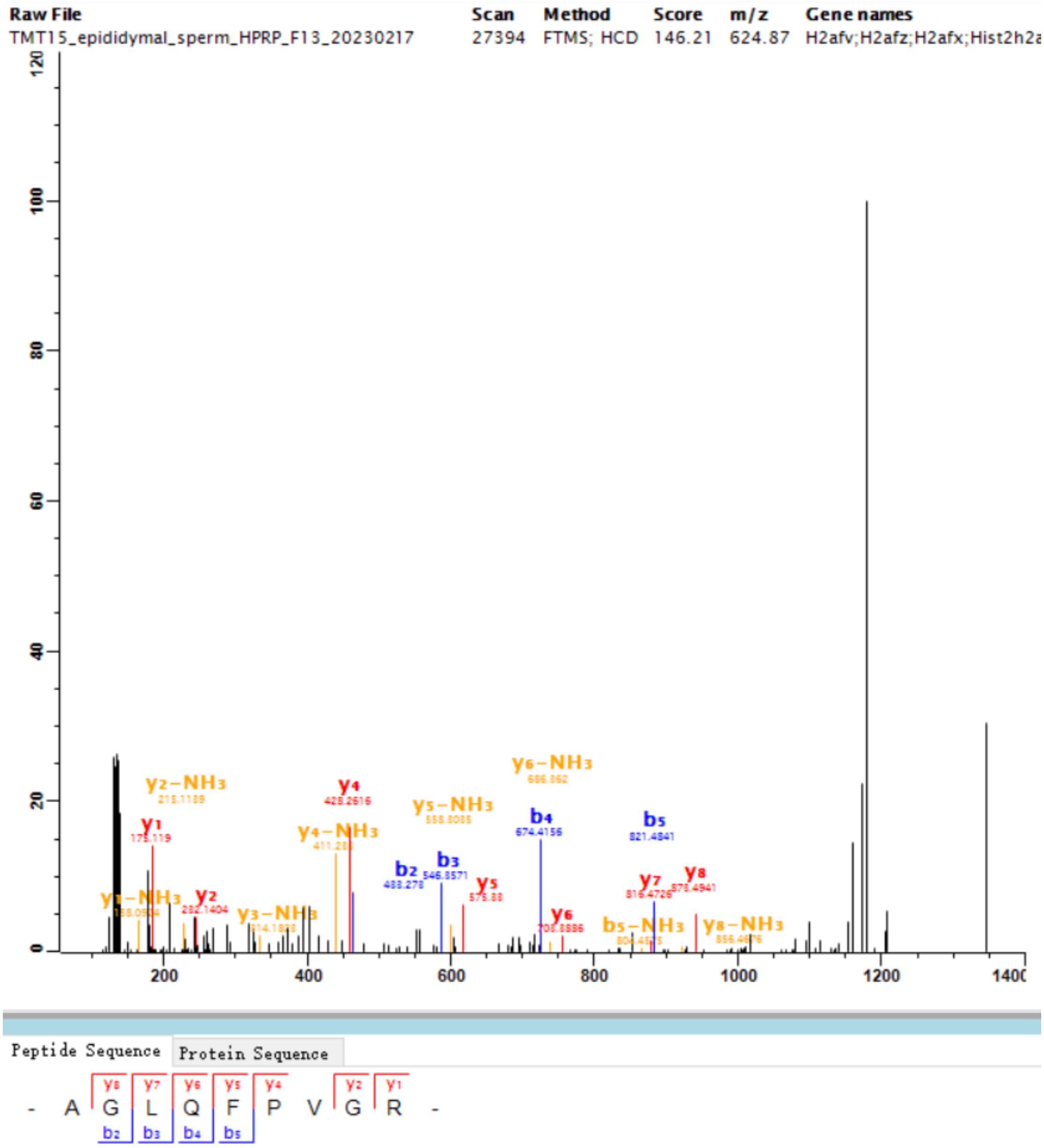
Raw File
TMT15_epididymal_sperm_HPRP_F13_20230217
Scan
27394
Method
FTMS; HCD
Score
146.21
m/z
624.87
Gene names
H2afv;H2afz;H2afx;Hist2h2a
y2−NH3
y1
y4
y4−NH3
y5−NH3
y6−NH3
b2
b3
b4
b5
y5
y6
y7
y8
y2
y3−NH3
b5−NH3
y8−NH3
0
20
40
60
80
100
120
200
400
600
800
1000
1200
Peptide Sequence
Protein Sequence
- A G L Q F P V G R -

Raw File	Scan	Method	Score	m/z	Gene names
TMT15_epididymal_sperm_HPRP_F12_20230215	19258	FTMS; HCD	101.43	447.29	Gzmb;Gzmc

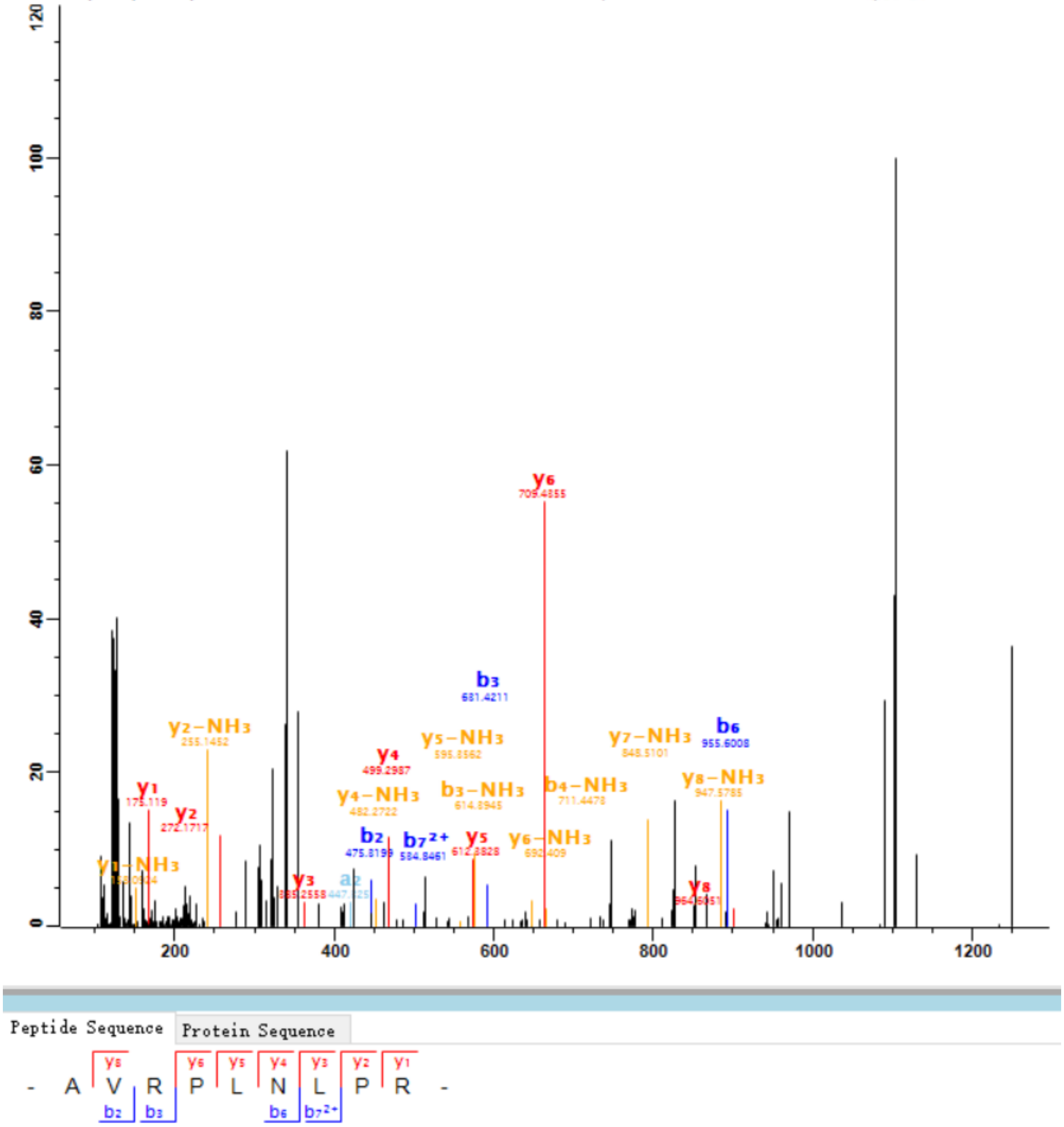

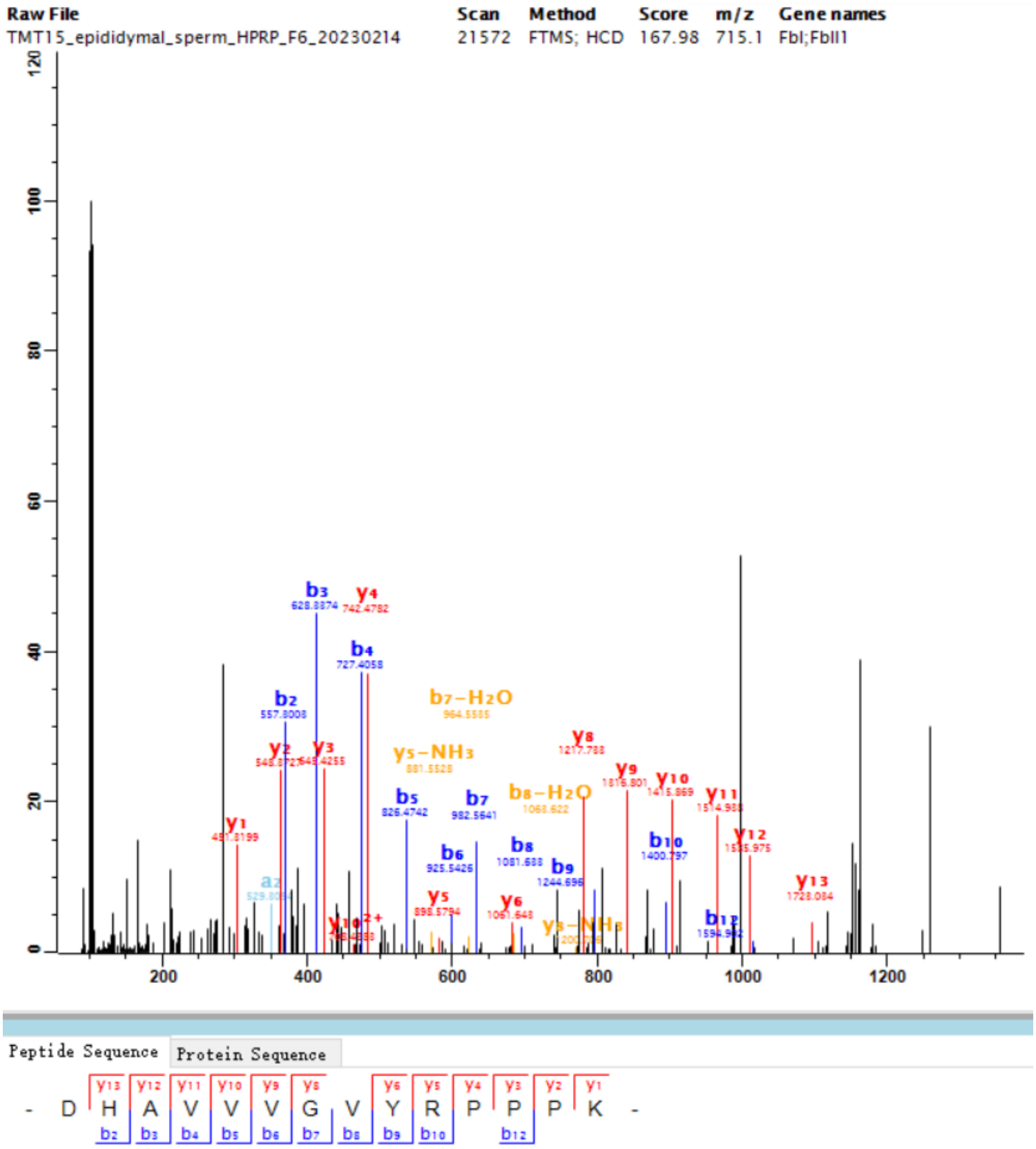

Raw File
TMT15_epididymal_sperm_HPRP_F6_20230214
Scan
21572
Method
FTMS; HCD
Score
167.98
m/z
715.1
Gene names
Fbl;Fbll1
Peptide Sequence
Protein Sequence
- D H A V V V G V Y R P P P K -

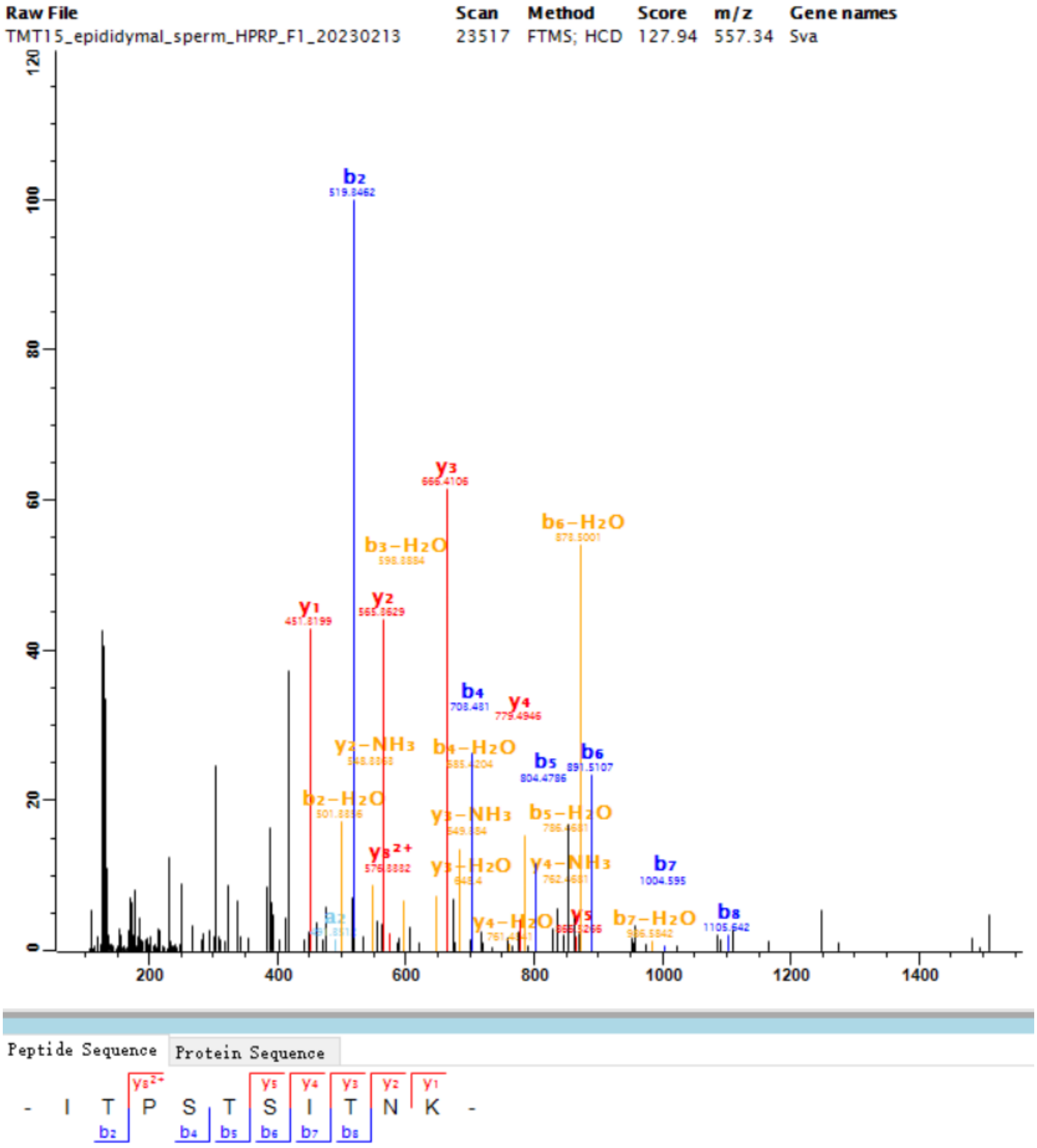
Raw File
TMT15_epididymal_sperm_HPRP_F1_20230213
Scan
23517
Method
FTMS; HCD
Score
127.94
m/z
557.34
Gene names
Sva
b2
519.8462
y3
666.4106
b6−H2O
873.5001
b3−H2O
598.8884
y1
451.8199
y2
565.8629
b4
708.481
y4
779.4946
y2−NH3
548.8368
b4−H2O
685.4204
b5
804.4786
b6
891.5107
b2−H2O
501.8856
y3−NH3
649.384
b5−H2O
786.4681
y5 2+
576.8882
y3−H2O
648.4
y4−NH3
762.4681
b7
1004.595
y4−H2O
761.4841
y5
866.5266
b7−H2O
986.5842
b8
1105.642
200
400
600
800
1000
1200
1400
0
20
40
60
80
100
120
Peptide Sequence
Protein Sequence
- I T P S T S I T N K -
y5 2+
y5 y4 y3 y2 y1
b2 b4 b5 b6 b7 b8

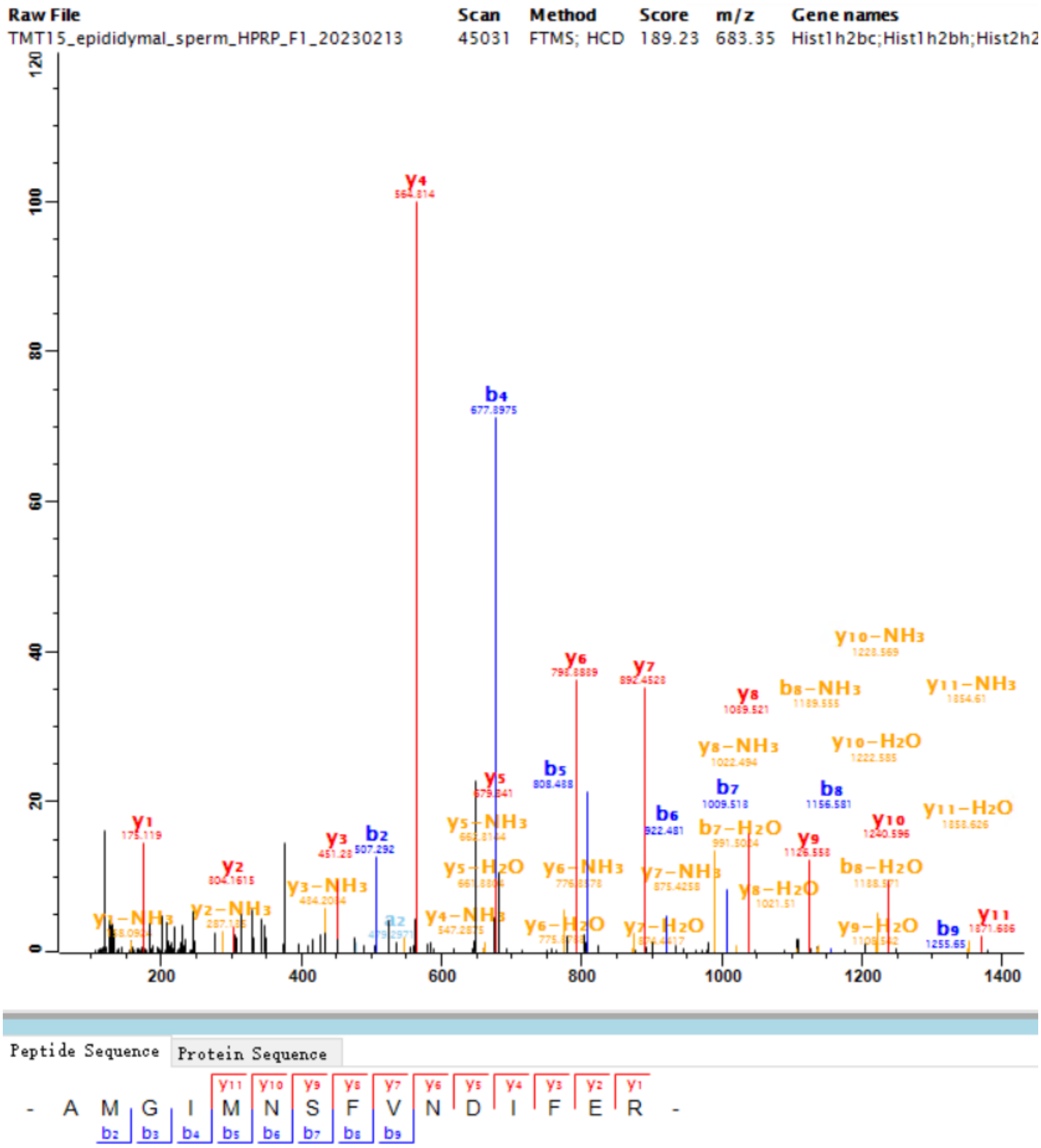
Raw File
TMT15_epididymal_sperm_HPRP_F1_20230213
Scan
45031
Method
FTMS; HCD
Score
189.23
m/z
683.35
Gene names
Hist1h2bc;Hist1h2bh;Hist2h2
y4
564.814
b4
677.8975
y6
y7
892.4528
y8
1089.521
b8-NH3
1189.555
y10-NH3
1223.569
y11-NH3
1354.61
y8-NH3
1022.494
y10-H2O
1222.585
b5
808.488
b7
1009.518
b8
1156.581
y5
b6
922.481
y11-H2O
y10
1240.596
y1
175.119
y3
b2
507.292
y5-NH3
b7-H2O
y9
y2
y5-H2O
y6-NH3
y7-NH3
875.4258
b8-H2O
y3-NH3
y8-H2O
y4-NH3
y6-H2O
y7-H2O
y9-H2O
b9
1255.65
y11
y1-NH3
y2-NH3
a2
Peptide Sequence
Protein Sequence
- A M G I M N S F V N D I F E R -

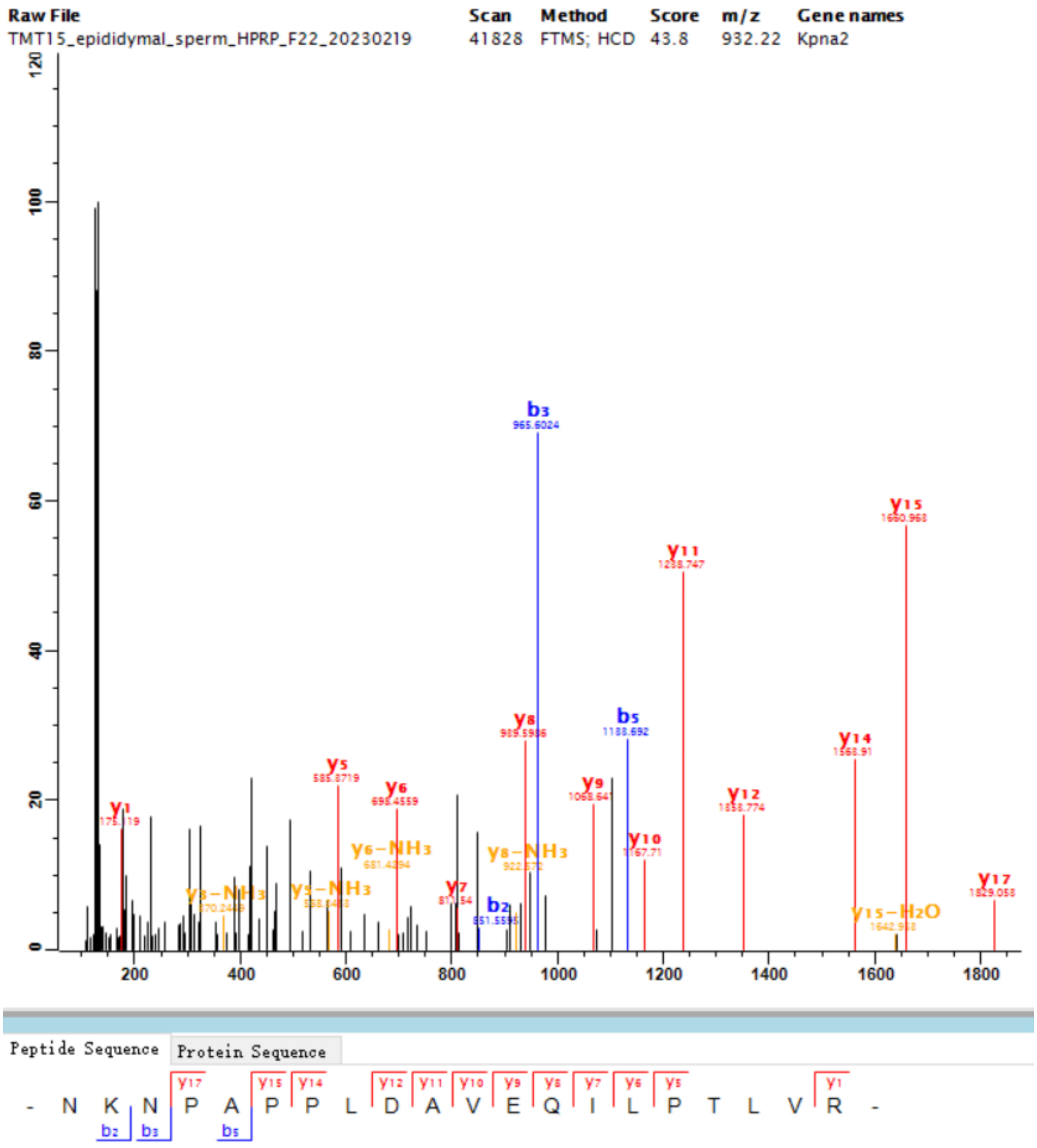
Raw File
TMT15_epididymal_sperm_HPRP_F22_20230219
Scan
41828
Method
FTMS; HCD
Score
43.8
m/z
932.22
Gene names
Kpna2
b3
965.6024
y15
1660.968
y11
1238.747
b5
1188.692
y8
989.5986
y14
1568.91
y5
585.3719
y6
698.4559
y9
1068.641
y12
1358.774
y1
y10
1167.71
y6-NH3
681.4294
y8-NH3
922.572
y7
b2
851.5595
y17
1829.058
y15-H2O
1642.958
200
400
600
800
1000
1200
1400
1600
1800
0
20
40
60
80
100
120
Peptide Sequence
Protein Sequence
- N K N P A P P L D A V E Q I L P T L V R -

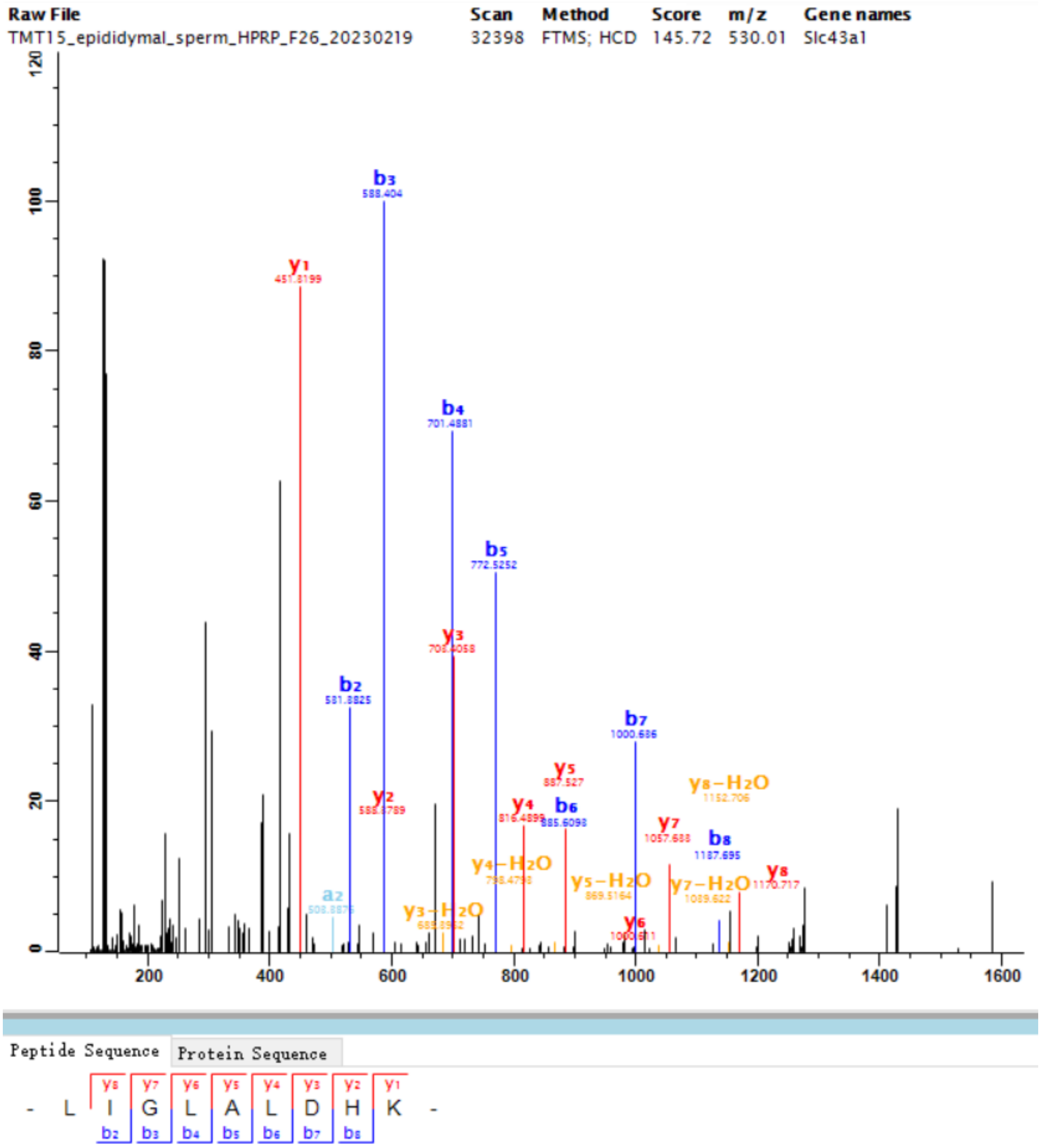

Raw File
TMT15_epididymal_sperm_HPRP_F26_20230219
Scan
32398
Method
FTMS; HCD
Score
145.72
m/z
530.01
Gene names
Slc43a1
b3 588.404
y1 451.8199
b4 701.4881
b5 772.5252
y3
b2 581.8825
b7 1000.686
y5 887.527
y8-H2O 1152.706
y2
y4
b6 885.6098
y7 1057.688
b8 1187.695
y4-H2O
y8 1170.717
a2
y5-H2O 869.5164
y7-H2O 1039.622
y3-H2O
y6
Peptide Sequence
Protein Sequence
- L I G L A L D H K -

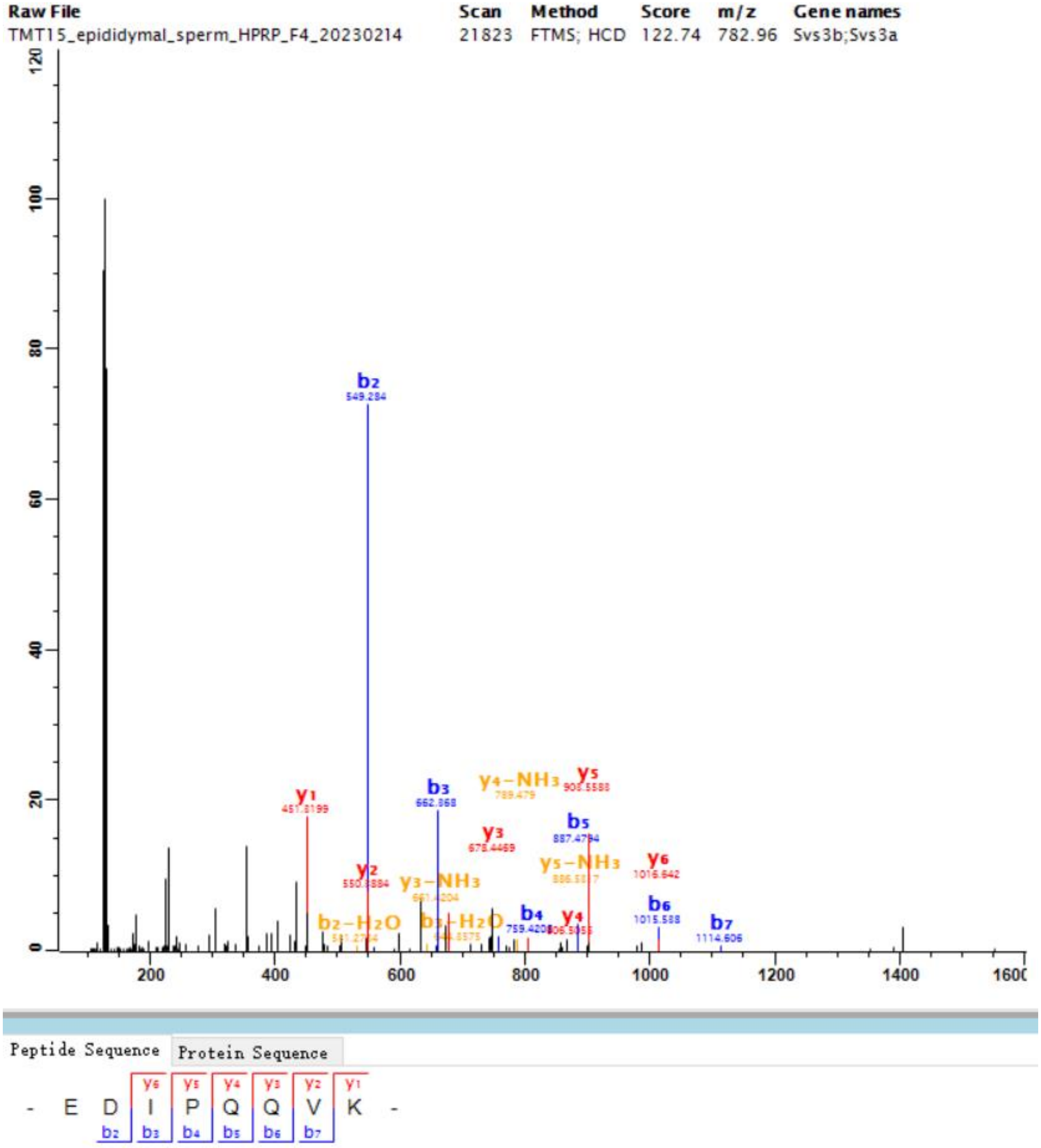

Raw File
TMT15_epididymal_sperm_HPRP_F4_20230214
Scan Method Score m/z Gene names
21823 FTMS; HCD 122.74 782.96 Svs3b;Svs3a
b2
549.284
b3
662.868
y1
y2
y3
y4
y5
y6
1016.642
b4
b5
b6
1015.588
b7
1114.606
y4-NH3
789.479
y3-NH3
y5-NH3
b2-H2O
b3-H2O
Peptide Sequence
Protein Sequence
- E D I P Q Q V K -
