# Supplementary material for: Quantitative Phosphoproteomic Profiling of Mouse Sperm Maturation in Epididymis Revealed Kinases Important for Sperm Motility
Source: Mol Cell Proteomics. 2024 Jul 6;23(8):100810. doi: 10.1016/j.mcpro.2024.100810 (PMC11338950; doi:10.1016/j.mcpro.2024.100810)
Supplement: Supplemental Figures [file mmc11.pdf]

Supplemental Information For

Quantitative phosphoproteomic profiling of mouse sperm maturation in epididymis revealed kinases important for sperm motility

Xiangzheng Zhang[1,#], Haixia Tu[1,2, #], Xin Zhou[1, #], Bing Wang[1,3], Yueshuai Guo[1], Chenghao Situ[1], Yaling Qi[1], Yan Li[1,2,*], Xuejiang Guo[1,*]

[1] State Key Laboratory of Reproductive Medicine and Offspring Health, Department of Histology and Embryology, Nanjing Medical University, Nanjing, 211166 China

[2] Department of Clinical Laboratory, Sir Run Run Hospital, Nanjing Medical University, Nanjing, 211100 China

[3] School of Medicine, Southeast University, Nanjing, 210009 China

*For corresponding author: Xuejiang Guo, State Key Laboratory of Reproductive Medicine and Offspring Health, Nanjing Medical University, 101 Longmian Avenue, Nanjing, Jiangsu, 211166, China; Yan Li, Department of Clinical Laboratory, Sir Run Run Hospital, Nanjing Medical University, 109 Longmian Avenue, Nanjing, Jiangsu, 211100 China

E-mail: guo_xuejiang@njmu.edu.cn (XG); yanli@njmu.edu.cn (YL)

Tel (lab): 86-25-86869383 (XG); 86-25-87115615 (YL)

[#]These authors contributed equally to this work.

This Supplemental Information include:

Fig.S1 to S3

Figure S1

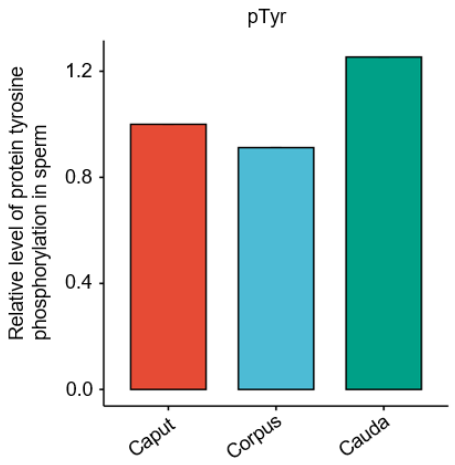

Figure S1. Quantification of immunoblot analysis of relative level of protein tyrosine phosphorylation in sperm from different epididymal regions

Figure S2

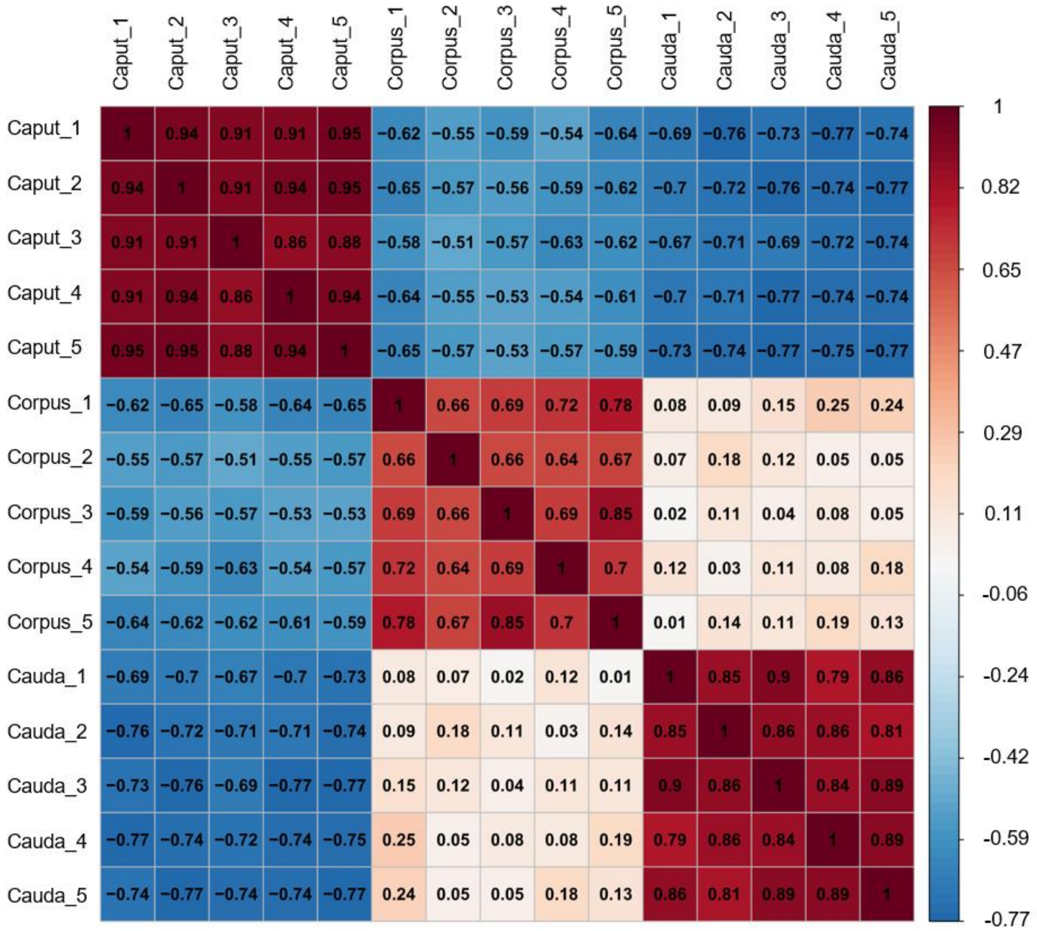

Figure S2. Correlation analysis of protein expression levels in sperm from different epididymal regions

Figure S3

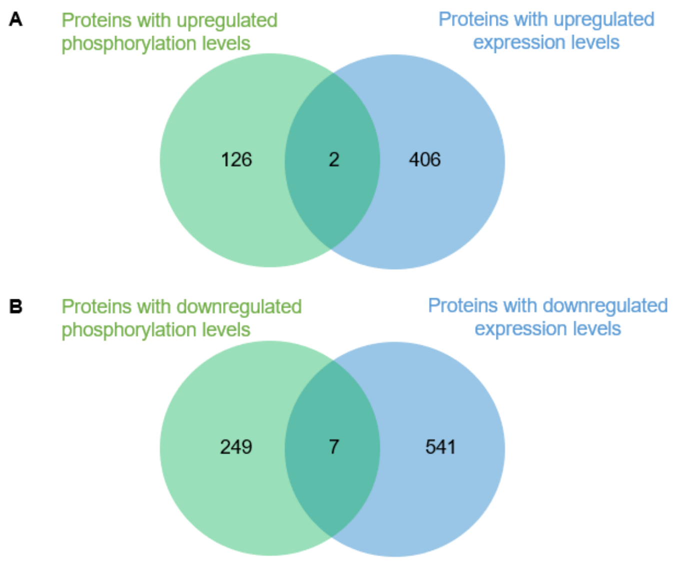

Figure S3. Overlap between proteins with altered phosphorylation levels and proteins with altered expression levels during sperm epididymal maturation.
